# Dynamic gene expressions of peripheral blood mononuclear cells in patients with acute exacerbation of chronic obstructive pulmonary disease: a preliminary study

**DOI:** 10.1186/s13054-014-0508-y

**Published:** 2014-11-19

**Authors:** Xiaodan Wu, Xiaoru Sun, Chengshui Chen, Chunxue Bai, Xiangdong Wang

**Affiliations:** Department of Respiratory Medicine, Zhongshan Hospital, Fudan University, Fenglin Rd. No180, 200032 Shanghai, China; Department of Respiratory Medicine, Wenzhou Medical University and The First Hospital, Nanbaixiang, 325000 Wenzhou, China; Shanghai Institute of Clinical Bioinformatics, Fudan University Center for Clinical Bioinformatics, Shanghai Respiratory Research Medicine, Fenglin Rd. No 180, 200032 Shanghai, China

## Abstract

**Introduction:**

Acute exacerbation of chronic obstructive pulmonary disease (AECOPD) is a serious event that is responsible for the progress of the disease, increases in medical costs and high mortality.

**Methods:**

The aim of the present study was to identify AECOPD-specific biomarkers by evaluating the dynamic gene expression profiling of peripheral blood mononuclear cells (PBMCs) from patients with AECOPD on days 1, 3 and 10 after hospital admission and to compare the derived data with data from healthy controls or patients with stable COPD.

**Results:**

We found that 14 genes were co–differentially upregulated and 2 downregulated greater than 10-fold in patients with COPD or AECOPD compared with the healthy individuals. Eight co–differentially upregulated genes and six downregulated genes were identified as a panel of AECOPD-specific genes. Downregulation of *TCF7* in PBMCs was found to be associated with the severity of COPD. Dynamic changes of Aminolevulinate-delta-synthase 2 and carbonic anhydrase I had similar patterns of Digital Evaluation Score System scores and may serve as potential genes of interest during the course of AECOPD.

**Conclusion:**

Thus, our findings indicate a panel of altered gene expression patterns in PBMCs that can be used as AECOPD-specific dynamic biomarkers to monitor the course of AECOPD.

**Electronic supplementary material:**

The online version of this article (doi:10.1186/s13054-014-0508-y) contains supplementary material, which is available to authorized users.

## Introduction

Chronic obstructive pulmonary disease (COPD) is an inflammation-based syndrome characterized by progressive deterioration of pulmonary function and increasing airway obstruction [1]. COPD is a major and growing public health burden, ranking as the fourth leading cause of death in the world [2]. In China, it is the fourth leading cause of mortality in urban areas and the third leading cause in rural areas [3]. Patients with COPD often experience a sudden deterioration, termed *acute exacerbations* of chronic obstructive pulmonary disease (AECOPD), along with a progressive decline in lung function; AECOPD becomes more frequent and severe when the severity of disease increases [4,5]. There is a great need for early and sensitive diagnosis and novel therapeutic targets for the disease, especially for patients with AECOPD in whom COPD is diagnosed in the late phase of disease, when they have significant or irreversible impairment [6].

The progress of COPD is accelerated by the occurrence of the exacerbation induced by multiple factors, including infection. AECOPD is a serious event that is related to decreased health status, increased medical and social costs and increased mortality [7]. Inflammatory cells (for example, lymphocytes, monocytes or macrophages, and their products) could interact with each other or with structural cells in the airways and the lung parenchymal and pulmonary vasculature, leading to the worsening of COPD [8]. Increased numbers of CD8+ lymphocytes were suggested as one of COPD’s characteristics, being present only in smokers who develop the disease [9]. Increased pulmonary inflammatory mediators in patients with COPD could attract inflammatory cells from the circulation, amplify the inflammatory process and induce structural changes [9].

Peripheral blood mononuclear cells (PBMCs) act as a critical component in the immune system to fight infection and adapt to intruders and play an important role in the development of AECOPD. Gene expression profiles of PBMCs were found to be disease-specific and associated with severity [10]. PBMC samples were suggested as easy to gather and important to the discovery of biomarkers for diagnosis and therapeutic management of COPD [11,12], although gene expression changes in lung tissues were noted to be associated with COPD [13-15]. The aim of the present study was to determine AECOPD-specific biomarkers of PBMCs using the concept of clinical bioinformatics and integrating genomics, bioinformatics, clinical informatics and systems biology [16-18]. We translated all clinical measures, including patient complaints, history, therapies, clinical symptoms and signs, physician’s examinations, biochemical analyses, imaging profiles, pathologies and other measurements, into digital format using a digital evaluation scoring system. PBMCs were isolated from healthy volunteers and patients with stable COPD or AECOPD, and we investigated the disease specificity that we inferred from clinical informatics analysis to search for COPD- or AECOPD-specific genes and dynamic biomarkers for AECOPD.

## Material and methods

### Patient population

**Figure 1:**
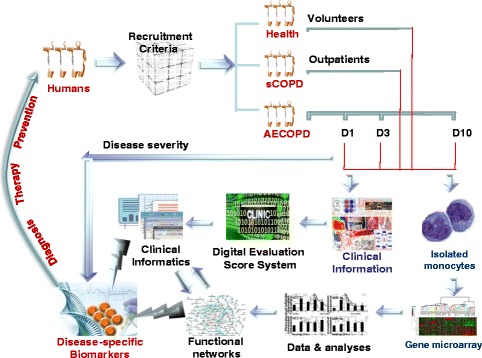
**Details of the study design.** Healthy volunteers and patients with stable chronic obstructive pulmonary disease (sCOPD) or acute exacerbation of COPD (AECOPD) at day 1 (D1), day 3 (D3) or day 10 (D10) of hospital admission of hospital were recruited into the present study according to the criteria stated in the text. All clinical information was collected and transferred into the clinical informatics database using the Digital Evaluation Score System. mRNAs of peripheral blood monocytes were harvested, and gene expression profiles were measured by human gene expression array and subjected to bioinformatics analysis. AECOPD-specific biomarkers were selected by integrating gene functional networks and profiles with clinical informatics data.

The present study was approved by the Ethical Evaluation Committee of Zhongshan Hospital and designed using a case–control approach. From among 220 candidates comprising blood donors (60 healthy controls), inpatients (80 patients with AECOPD) and outpatients (80 patients with stable COPD) in Zhongshan Hospital, patients with AECOPD, patients with stable COPD and healthy controls matched for age and sex were recruited into the study between October 2011 and March 2012. The inclusion criteria for patients with COPD were as follows: (1) forced expiratory volume in 1 second (FEV_1_) <80% of predicted value adjusted for age, weight and height, and (2) an improvement in FEV_1_ following bronchodilator inhalation <12% of baseline FEV_1_. Patients with asthma who had a persistent airflow obstruction were excluded. Stable COPD was defined according to American Thoracic Society/European Respiratory Society consensus criteria as no requirement for increased treatment above maintenance therapy, other than bronchodilators, for 30 days [1]. AECOPD was the reason for hospital admission and was characterized as a worsening of the patient’s respiratory symptoms that was beyond normal day-to-day variations and led to a change in medication [4,19]. Healthy controls enrolled were blood donors at Zhongshan Hospital. Subjects with respiratory diseases, or any family history of lung disease, were excluded. PBMCs were harvested once from healthy controls and patients with stable COPD, as well as from patients with AECOPD, on the admission day and 3 and 10 days after the admission. Informed consent was given by the subjects themselves before they underwent lung function tests, high-resolution computed tomography and blood collection. The time points used in the present study were selected on the basis of our previous study for collecting plasma samples from healthy controls and from patients with stable COPD or AECOPD. The details of the study design are explained in Figure 1.

### Digital evaluation score system

The Digital Evaluation Score System (DESS) is a score index used to translate clinical descriptions and information into clinical informatics, as described previously [20]. Using this instrument, we took into account patient symptoms and signs, biochemical analyses and clinical imaging for patients with stable COPD or AECOPD. Briefly, for the assessment of severity, each component was assigned a score of 0, 1, 2 or 4. The score of 4 as the maximum value indicates far above normal range or much severer condition, and 0 as the minimum value indicates within normal physiological range. After compiling patient data, we added the points for each variable. The DESS scores ranged from 0 to 256 points, with a higher score indicating a severer condition. Patients were scored on the day when their blood samples were collected.

### Isolation of PBMC RNA

PBMCs were isolated by using BD Vacutainer CPT cell preparation tubes (Becton Dickinson, Franklin Lakes, NJ, USA) according to the manufacturer’s instructions. Approximately 4 ml of whole blood was collected from each subject. Following centrifugation, cells were lysed for RNA isolation. DNase-free total RNA preparation was performed using TRIzol reagent (Life Technologies, Carlsbad, CA, USA) and the RNeasy kit (QIAGEN, Valencia, CA, USA) according to the manufacturers’ recommendations. RNA concentrations were determined by using a NanoDrop ND-1000 spectrophotometer (NanoDrop, Wilmington, DE, USA). RNA quality was assessed on an Agilent 2100 Bioanalyzer (Agilent Technologies, Santa Clara, CA, USA), and samples with an RNA integrity number >6.0 were used.

### Microarray analysis

The Human 12×135K Gene Expression Array (Roche NimbleGen Systems, Madison, WI, USA), with about 45,000+ human genes and transcripts represented with public domain annotations, was applied for this study. Sample labeling and array hybridization were performed according to the one-color microarray-based gene expression analysis protocol (Roche NimbleGen Systems). Double-stranded cDNA (ds-cDNA) was synthesized from 5 μg of total RNA using an Invitrogen SuperScript reverse transcriptase ds-cDNA synthesis kit (Life Technologies) in the presence of 100 pmol oligo(dT) primers. ds-cDNA was cleaned and labeled in accordance with the NimbleGen gene expression analysis protocol. Briefly, ds-cDNA was incubated with 4 μg of RNase A at 37°C for 10 minutes and cleaned using phenol:chloroform:isoamyl alcohol, followed by ice-cold absolute ethanol precipitation. The purified cDNA was quantified using the NanoDrop ND-1000 spectrophotometer. For Cy3 labeling of cDNA, the NimbleGen one-color DNA labeling kit was used according to the manufacturer’s guidelines as detailed in its gene expression analysis protocol. One microgram of ds-cDNA was incubated for 10 minutes at 98°C with 1 optical density of Cy3-9mer primer. Next, 100 pmol of deoxynucleoside triphosphates and 100 U of the Klenow fragment (New England Biolabs, Ipswich, MA, USA) were added, and the mix was incubated at 37°C for 2 hours. The reaction was stopped by adding 0.1 vol of 0.5 M ethylenediaminetetraacetic acid, and the labeled ds-cDNA was purified by isopropanol/ethanol precipitation. Microarrays were hybridized at 42°C for 16 to 20 hours with 4 μg of Cy3-labeled ds-cDNA in NimbleGen hybridization buffer/hybridization component A in a hybridization chamber. Following hybridization, washing was performed using the NimbleGen wash buffer kit. After being washed in an ozone-free environment, the slides were scanned using an Axon GenePix 4000B microarray scanner (Molecular Devices, Sunnyvale, CA, USA).

### Data analysis

For clinical data, all values were expressed as mean ± SE. Analyses were performed using SPSS software (SPSS 18.0; SPSS, Chicago, IL, USA). For microarray analysis, slides were scanned at 5 μm/pixel resolution using the Axon GenePix 4000B microarray scanner piloted by GenePix Pro 6.0 software (Molecular Devices). Scanned images (in TIFF file format) were then imported into NimbleScan software (version 2.5) files for grid alignment and expression data analysis. Expression data were normalized through quantile normalization and the Robust Multi-array Average (RMA) algorithm included in the NimbleScan software. The probe-level (*_norm_RMA.pair) files and gene-level (*_RMA.calls) files were generated after normalization. All gene-level files were imported into GeneSpring GX software (version 11.5.1; Agilent Technologies) for further analysis. Differentially expressed genes between two samples were identified by fold change filtering. Hierarchical clustering was performed using the GeneSpring GX software. Gene Ontology (GO) database analysis and pathway analysis were performed using the standard enrichment computation method. The GO database covers three domains: biological process, cellular component and molecular function. Fisher’s exact test was used to find more overlaps between the descriptive list and the GO annotation list than would be expected by chance. The *P*-value denoted the significance of GO term enrichment in the descriptive genes. The gene expression data are publicly available in the Gene Expression Omnibus database [GEO:GSE60399] [21].

## Results

### Clinical informatics analysis

Clinical phenotypes are described in Table 1, including age, sex, smoking status, lung function test results and emphysema scores of the subjects. Control subjects were nonsmokers, and patients with stable COPD or AECOPD were ex-smokers. Because of the severity of disease, lung function tests were not performed at the onset of AECOPD; however, the baseline FEV_1_/forced vital capacity (FVC%) and FEV_1_/predicted percentage of patients with AECOPD were similar to those of patients with stable COPD. In addition, there was no significant difference in the extent of emphysema between patients with stable COPD and those with AECOPD (*P* = 0.47). DESS scores of subjects from each group are shown in Additional file 1. DESS values of patients with stable COPD or AECOPD were significantly higher than those of control subjects (*P <* 0.01), as shown in Table 2. DESS scores represented the severity of COPD and declined as the patient’s condition improved. DESS values of patients with AECOPD on day 1 of hospital admission (AE-1) were significantly higher than those on day 3 (AE-3) and day 10 (AE-10) (*P <* 0.05 and *P <* 0.01, respectively) (Table 2).

**Table 1:** **Clinical phenotypes of healthy controls, patients with stable chronic obstructive pulmonary disease and patients with acute exacerbation of chronic obstructive pulmonary disease** ^**a**^

**Groups**	**Subject no.**	**Age (yr)**	**Smoking status**	**FEV**_**1**_ **/FVC%**	**FEV**_**1**_ **/pred%**	**Goddard emphysema score**
Control	1	56	Nonsmoker	75	85	0
	2	53	Nonsmoker	80	87	0
	3	62	Nonsmoker	77	91	0
	4	68	Nonsmoker	81	83	0
	5	58	Nonsmoker	79	81	0
	6	67	Nonsmoker	76	90	0
Mean ± SE		60.7 ± 2.5		78.0 ± 1.0	86.2 ± 1.6	0.0 ± 0.0
Stable COPD	1	71	Ex-smoker	57	47	10
	2	75	Ex-smoker	46	66	6
	3	61	Ex-smoker	46	47	8
	4	57	Ex-smoker	38	29	12
	5	59	Ex-smoker	67	66	7
	6	53	Ex-smoker	29	36	11
Mean ± SE		62.7 ± 3.5		47.2 ± 5.5	48.5 ± 6.2	9.0 ± 1.0
AECOPD	1	77	Ex-smoker	40	42	10
	2	72	Ex-smoker	36	27	11
	3	65	Ex-smoker	28	33	16
	4	56	Ex-smoker	48	61	6
	5	61	Ex-smoker	69	55	4
	6	67	Ex-smoker	56	60	8
Mean ± SE		66.3 ± 3.1		46.2 ± 6.0	46.3 ± 5.9	9.2 ± 1.7

^a^AECOPD, Acute exacerbation of chronic obstructive pulmonary disease; COPD, Chronic obstructive pulmonary disease; FEV_1_, Forced expiratory volume in 1 second; FVC, Forced vital capacity; pred, Prediction. Data represent information gathered on days 1, 3 and 10 of hospital admission.

**Table 2:** **Digital evaluation score system scores** ^**a**^

	**DESS scores**				
**Patient no.**	**Control**	**Stable COPD**	**AE-1**	**AE-3**	**AE-10**
1	0	30	100	78	43
2	4	27	81	66	46
3	8	35	86	76	36
4	4	55	70	51	30
5	3	38	80	71	35
6	0	47	97	81	30
Mean ± SE	3.2 ± 1.2	38.7 ± 4.3	85.7 ± 4.6	70.5 ± 4.5	36.7 ± 2.7

^a^AE-1, Day 1 of hospital admission; AE-3, Day 3 of hospital admission; AE-10, Day 10 of hospital admission; COPD, Chronic obstructive pulmonary disease; DESS, Digital evaluation score system.

### Gene expression profiles

**Figure 2:**
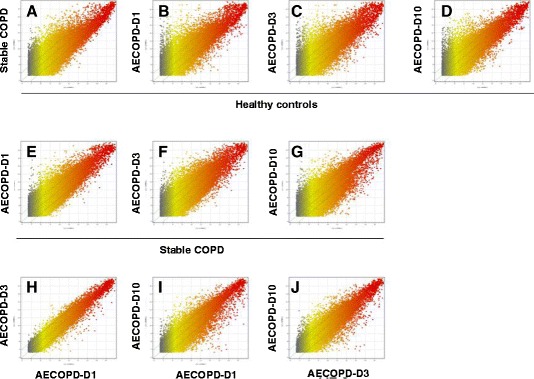
**Scatterplots showing variations in gene expression profiles.** Scatterplots of peripheral blood monocytes between patients with stable chronic obstructive pulmonary disease (Stable COPD) **(A)**, acute exacerbation of chronic obstructive pulmonary disease at day 1 of hospital admission (AECOPD-D1) **(B)**, AECOPD at day 3 of hospital admission (AECOPD-D3) **(C)** or AECOPD at day 10 of hospital admission (AECOPD-D10) **(D)** compared with healthy controls. Scatterplots also illustrate variations between AECOPD-D1 **(E)**, AECOPD-D3 **(F)** or AECOPD-D10 **(G)** and stable COPD; between AECOPD-D3 **(H)** or AECOPD-D10 **(I)** with AECOPD-D1; and between AECOPD-D3 and AECOPD-D10 **(J)**.

The quality of the genetic data obtained after filtering and the distribution of data sets were assessed and visualized by creating box plots, which showed that there were no significant differences in the distributions of log_2_ ratios among the groups (see Additional file 2: Figure S1). The variation or reproducibility of gene expression between arrays of different groups was visualized and assessed by creating scatterplots, which are shown in Figure 2. There was a significant variation in gene arrays between healthy controls and patients with stable COPD or AECOPD (Figures 2A to 2D) and between patients with stable COPD and AECOPD (Figures 2E to 2G). The variation in gene array data at AE-1 and AE-3 was significantly different from that at AE-10 (Figures 2I and 2J), whereas there was no difference between AE-1 and AE-3 (Figure 2H). The results of hierarchical clustering showed gene expression profiles similar to those revealed by the scatterplots shown in Figure S2 of Additional file 2.

To identify differentially expressed genes, a fold change filtering between each group pair was performed with a threshold fold change ≥2.0. There were ten comparison pairs with information for fold changes and regulation (that is, SEQ-ID, log fold change, log or absolute fold change, or regulation), normalized intensities or annotations (that is, GENE_NAME, synonyms, description, NCBI_GENE_ID, chromosome, GO, UniGene ID, The Institute of Genomic Research Database-TDB (TIGRID) or Ensembl ID), as shown in Additional file 3. Table 3 shows the number of genes overexpressed more than twofold, (for example, 4,508, 3,899, 4,167 and 3,488 genes of stable, AE-1, AE-3 and AE-10, respectively, above controls; 4,067, 5,063 or 5,451 genes of AE-1, AE-3 and AE-10, respectively, above stable COPD; 586 genes of AE-3 above AE-1; and 1,735 and 1,706 genes of AE-10, respectively, above AE-1 and AE-3). Tables 4, 5 and 6, respectively, list the genes overexpressed (above controls) in PBMCs from patients with stable COPD, AE-1, AE-3 or AE-10 by more than 30-fold (Table 4), between 20- and 30-fold (Table 5) and between 15- and 20-fold (Table 6). Tables 7, 8 and 9 list the genes overexpressed (above patients with stable COPD) in PBMCs from patients with AE-1, AE-3 or AE-10 by more than 30-fold (Table 7), between 20- and 30-fold (Table 8) and between 15- and 20-fold. Table 10 presents upregulated genes in PBMCs of patients at AE-1, AE-3 or AE-10.

**Table 3:** **Genes upregulated in peripheral blood mononuclear cells** ^**a**^

	**Fold changes in upregulated genes (** ***n*** **)**								
**Comparisons**	**>2**	**>5**	**>8**	**>10**	**>15**	**>20**	**>30**	**>50**	**>100**
Stable vs Con	4,508	671	217	145	49	27	9	1	0
AE-1 vs Con	3,899	734	334	221	136	86	40	18	3
AE-3 vs Con	4,167	742	358	259	149	97	51	17	5
AE-10 vs Con	3,488	677	331	238	116	74	35	10	1
AE-1 vs Stable	4,067	389	135	80	36	21	9	3	1
AE-3 vs Stable	5,063	620	221	146	56	24	10	1	0
AE-10 vs Stable	5,451	534	178	117	56	33	14	1	0
AE-3 vs AE-1	586	8	2	2	0	0	0	0	0
AE-10 vs AE-1	1,735	164	55	26	10	4	1	0	0
AE-10 vs AE-3	1,706	156	49	29	2	2	1	0	0

^a^Data are number of upregulated genes expressed in peripheral blood mononuclear cells of healthy controls (Con) or of patients with stable chronic obstructive pulmonary disease (Stable) or acute exacerbation of chronic obstructive pulmonary disease on hospital admission day 1 (AE-1), day 3 (AE-3) and day 10 (AE-10).

**Table 4:** **Genes upregulated >30-fold in peripheral blood mononuclear cells** ^**a**^

**Stable vs control**		**AE-1 vs control**		**AE-3 vs control**		**AE-10 vs control**	
**Fold changes**	**Genes**	**Fold changes**	**Genes**	**Fold changes**	**Genes**	**Fold changes**	**Genes**
31.7	*REXO1L2P*	30.3	*HP*	30.1	*FOS*	30.8	*EMP2*
33.0	*DEFA1*	30.5	*LOC152573*	30.6	*BPIL1*	31.0	*SEPP1*
33.3	*DUB3*	31.2	*INHBA*	31.0	*ARG1*	31.0	*FOLR1*
37.2	*LOC402207*	31.4	*COL6A3*	31.6	*N/A*	31.1	*GPX3*
37.3	*DUB3*	32.4	*MPO*	31.9	*LOC152573*	31.2	*SFTPB*
40.5	*LOC402110*	32.6	*ELF3*	32.5	*COL6A3*	31.4	*S100A14*
43.1	*LOC653600*	34.4	*CLDN4*	32.9	*TIMP3*	33.1	*FOLR1*
43.5	*N/A*	34.9	*DCN*	33.5	*FOS*	33.4	*CDH5*
50.7	*MGC45438*	35.7	*CTGF*	34.4	*KRT19*	34.9	*CAV1*
		35.7	*MMP2*	34.7	*INHBA*	35.4	*DLC1*
		36.2	*MFAP4*	35.2	*HP*	35.6	*FOSB*
		37.1	*EPB42*	35.6	*CD177*	36.1	*KRT19*
		37.2	*H19*	36.5	*LCN2*	36.4	*SUSD2*
		37.3	*ATP1B1*	36.9	*CTGF*	36.9	*FN1*
		37.5	*INHBA*	37.9	*MMP8*	37.2	*ADH1C*
		38.0	*AZU1*	38.3	*ORM1*	37.2	*RNASE1*
		38.5	*LCN2*	38.8	*ELF3*	37.3	*IL1RL1*
		39.6	*CEACAM8*	38.9	*DCN*	41.1	*FOLR1*
		40.3	*CALCA*	39.0	*CTSG*	41.3	*DHCR24*
		41.4	*LOC387763*	39.1	*CLDN4*	41.3	*LOC387763*
		42.2	*CEACAM3*	39.3	*CALCA*	42.0	*ADH1B*
		45.9	*UNQ473*	40.0	*DCN*	43.6	*LAMA3*
		54.0	*BPIL1*	40.1	*FOSB*	45.0	*GPX3*
		56.2	*FN1*	41.1	*ATP1B1*	47.9	*DCN*
		56.7	*CEACAM5*	41.6	*MFAP4*	49.1	*EPAS1*
		58.4	*MMP8*	41.8	*FN1*	50.9	*CNN3*
		65.0	*CALCA*	42.0	*MMP2*	51.5	*DCN*
		66.3	*BPI*	42.0	*GPR97*	54.5	*LOC653509*
		68.7	*DEFA1*	42.2	*INHBA*	56.2	*CXCL2*
		72.3	*COL1A2*	45.5	*AZU1*	58.2	*MGC45438*
		77.2	*CA1*	46.0	*BPI*	58.5	*CYP4B1*
		80.2	*PLUNC*	46.4	*LOC387763*	59.3	*CTGF*
		83.0	*CEACAM1*	46.6	*MPO*	75.8	*GPRC5A*
		83.9	*DEFA4*	50.0	*HP*	88.9	*TIMP3*
		85.0	*COL3A1*	50.7	*ORM2*	149.5	*MFAP4*
		96.1	*DEFA1*	53.1	*UNQ473*		
		99.4	*CEACAM5*	57.8	*AQP9*		
		101.2	*CEACAM1*	59.6	*CEACAM5*		
		115.8	*LOC653600*	59.6	*BPIL1*		
		140.3	*DEFA4*	61.0	*CEACAM1*		
				62.8	*DEFA1*		
				66.5	*CEACAM1*		
				72.6	*DEFA4*		
				82.5	*PLUNC*		
				86.7	*DEFA1*		
				92.9	*COL1A2*		
				100.8	*CEACAM5*		
				101.1	*CALCA*		
				109.4	*LOC653600*		
				111.5	*COL3A1*		
				165.7	*DEFA4*		

^a^Data are from patients with stable chronic obstructive pulmonary disease (Stable) or acute exacerbation of chronic obstructive pulmonary disease on day 1 (AE-1), day 3 (AE-3) and day 10 (AE-10) of hospital admission, as compared to healthy controls.

**Table 5:** **Genes upregulated between 20- and 30-fold in peripheral blood mononuclear cells of patients with stable COPD or AECOPD compared with healthy control subjects** ^**a**^

**Stable vs control**		**AE-1 vs control**		**AE-3 vs control**		**AE-10 vs control**	
**Fold changes**	**Genes**	**Fold changes**	**Gene**	**Fold changes**	**Genes**	**Fold changes**	**Genes**
20.1	*P8*	20.3	*PLAU*	20.0	*ALPL*	20.3	*SCNN1A*
20.1	*REXO1L5P*	21.0	*COL6A3*	20.1	*MUC1*	20.4	*MGC45438*
20.2	*UNQ473*	21.1	*SLC25A37*	20.2	*SPDEF*	20.7	*FBLN1*
20.2	*DEFA1*	21.1	*HIG2*	20.3	*HIG2*	20.7	*CLDN4*
20.5	*LOC440015*	21.2	*GPRC5A*	20.4	*KLK11*	20.9	*SFTPA2*
21.1	*LOC391749*	21.2	*CFB*	20.4	*MGP*	21.0	*FKBP9*
21.3	*MGC45438*	21.3	*LTF*	20.4	*GPR109A*	21.1	*FAM107A*
21.7	*RP11-146D12.2*	21.4	*VSIG4*	21.0	*LOC653342*	21.3	*N/A*
22.0	*LOC399839*	21.7	*FOSB*	21.1	*CFB*	21.4	*C10orf10*
22.9	*SPDEF*	21.9	*SLC25A37*	21.3	*P8*	21.5	*SELENBP1*
23.0	*CLDN4*	22.0	*ARG1*	21.8	*PBEF1*	21.6	*ANXA3*
24.7	*LOC349196*	22.0	*SPDEF*	21.9	*S100P*	21.6	*IFI27*
25.3	*STAC2*	22.2	*LTF*	21.9	*MS4A3*	21.8	*C1QC*
25.8	*REXO1L3P*	22.3	*FOS*	22.4	*COL6A3*	21.9	*SEPP1*
26.3	*SCGB3A1*	22.6	*FAM46C*	23.1	*MANSC1*	22.0	*KLK11*
26.9	*RNASE1*	22.6	*ISLR*	23.2	*COL1A2*	22.1	*P8*
27.0	*AZGP1*	22.6	*COL1A2*	23.2	*GCA*	22.1	*LOC653723*
29.5	*H19*	22.8	*ATP1B1*	23.3	*LTBP2*	22.5	*LOC391359*
		23.8	*SCNN1A*	23.9	*CHI3L1*	22.7	*LAMB2*
		23.8	*SERPINE1*	24.0	*TMC5*	22.8	*AQP1*
		23.8	*EPB42*	24.2	*CD24*	24.0	*C9orf61*
		23.8	*C1QC*	24.2	*HP*	24.1	*C4BPA*
		23.9	*RGS1*	24.3	*ISLR*	24.2	*LTBP2*
		23.9	*ORM2*	24.3	*SIX1*	24.3	*UNQ473*
		24.1	*COL5A1*	24.5	*APOE*	24.5	*TMEM139*
		24.5	*MS4A3*	24.6	*COL3A1*	24.6	*N/A*
		25.6	*CD177*	24.6	*LOC646309*	25.7	*OLFML3*
		25.6	*APOE*	24.7	*CEACAM3*	25.9	*SNF1LK*
		26.4	*C20orf114*	24.9	*AATK*	25.9	*A2M*
		26.6	*BPIL1*	25.3	*LTF*	26.4	*FXYD3*
		27.1	*CTSG*	25.4	*ALPL*	27.0	*HP*
		27.4	*FOS*	25.6	*ACSL1*	27.1	*N/A*
		27.6	*ALAS2*	26.2	*CEACAM6*	27.4	*LOC653509*
		28.0	*INHBA*	26.3	*COL5A1*	28.0	*LDB2*
		28.0	*TIMP3*	26.4	*KLK11*	28.0	*OLFML3*
		28.1	*COL3A1*	26.7	*PRTN3*	28.5	*SFTPA1*
		28.1	*SLC4A1*	26.9	*RGS1*	28.6	*MUC1*
		28.2	*KLK11*	27.3	*KCNJ15*	29.6	*HSPA12B*
		28.2	*LOC653492*	27.4	*CAMP*	29.8	*MFAP4*
		28.5	*LOC203510*	27.6	*PLAU*		
		28.7	*CEACAM3*	27.8	*LTF*		
		28.8	*DCN*	27.9	*ANXA3*		
		28.9	*CEACAM1*	28.0	*H19*		
		29.0	*CEACAM6*	28.0	*SERPINE1*		
		29.3	*SELENBP1*	28.1	*LTF*		
		29.7	*KRT19*	28.3	*INHBA*		

^a^Data are from patients with stable chronic obstructive pulmonary disease (Stable) or acute exacerbation of chronic obstructive pulmonary disease on day 1 (AE-1), day 3 (AE-3) and day 10 (AE-10) of hospital admission, as compared to healthy controls.

**Table 6:** **Genes upregulated between 15- and 20-fold in peripheral blood mononuclear cells of patients with stable COPD or AECOPD compared with healthy control subjects** ^**a**^

**Stable vs control**		**AE-1 vs control**		**AE-3 vs control**		**AE-10 vs control**	
**Fold changes**	**Genes**	**Fold changes**	**Genes**	**Fold changes**	**Genes**	**Fold changes**	**Genes**
15.2	*LOC645558*	15.0	*CNN3*	15.0	*GPR109B*	15.0	*USP54*
15.7	*N/A*	15.0	*GPT2*	15.0	*LOC653492*	15.0	*MGC45438*
15.9	*LOC653455*	15.1	*ORM1*	15.2	*RNASE1*	15.2	*SLCO2A1*
15.9	*DUX4*	15.1	*LOC402110*	15.3	*FN1*	15.3	*AGER*
16.1	*LOC653768*	15.1	*MDK*	15.5	*ACSL1*	15.3	*FLJ11259*
16.5	*RAB17*	15.2	*ELF3*	15.5	*CDH5*	15.5	*CLEC3B*
16.6	*LOC653541*	15.2	*PSG8*	15.6	*FOLR3*	15.8	*ADCY4*
16.6	*LOC391763*	15.3	*SLC25A37*	15.8	*PVRL2*	16.0	*FN1*
16.7	*LOC642286*	15.4	*FKBP9*	15.9	*KRT19*	16.1	*HP*
16.7	*S100A14*	15.5	*C1QB*	15.9	*MDK*	16.1	*CKB*
16.7	*NBPF9*	15.6	*BPGM*	16.0	*APOC1*	16.1	*CYP4B1*
16.9	*PSG8*	15.7	*AQP9*	16.3	*NOL3*	16.2	*RARRES2*
17.0	*REXO1L6P*	15.7	*LOC402207*	16.3	*ATP1B1*	16.3	*TSPAN1*
17.0	*MLPH*	15.7	*PSG11*	16.4	*TMC4*	16.6	*SDC4*
17.1	*FAM90A7*	16.0	*KLK11*	16.4	*VEGF*	16.7	*ERG*
17.4	*LOC401650*	16.2	*KIAA0703*	16.6	*SPAG4*	16.8	*LOC653107*
17.8	*DUB3*	16.2	*IGFBP5*	16.8	*LIF*	17.2	*RAB25*
17.9	*MGC45438*	16.2	*IGFBP3*	16.8	*CCDC80*	17.2	*COL1A2*
18.9	*COL3A1*	16.2	*N/A*	16.9	*CEACAM3*	17.3	*DCN*
19.1	*LOC645732*	16.2	*SLC25A37*	16.9	*IGFBP3*	17.5	*TSPAN13*
19.8	*LOC392188*	16.3	*SIX1*	17.1	*CXCL2*	17.6	*HSD17B6*
20.0	*MUC1*	16.3	*LOC645009*	17.2	*FKBP9*	17.8	*RHOB*
		16.4	*C1QA*	17.2	*CEACAM1*	17.9	*KRT19*
		16.5	*UBD*	17.7	*ELF3*	18.0	*AQP9*
		16.6	*LOC653342*	17.7	*CNN3*	18.2	*FOLR1*
		17.0	*GPR97*	17.8	*PGLYRP1*	18.2	*IL1RL1*
		17.1	*COL1A1*	17.9	*KRT23*	18.2	*SERPING1*
		17.3	*ALPL*	18.1	*SLC44A4*	18.3	*MGC35295*
		17.4	*FBLN1*	18.1	*SCNN1A*	18.4	*FLJ43663*
		17.5	*HIG2*	18.4	*FBLN1*	18.6	*TGM2*
		17.7	*COL8A1*	18.5	*HPR*	18.6	*ADH1C*
		17.9	*TMC5*	18.6	*SYT7*	18.7	*KIAA1026*
		18.1	*LTBP2*	18.6	*CEACAM8*	19.1	*DKFZP686A01247*
		18.4	*SLC25A37*	18.8	*C1R*	19.2	*CCDC48*
		18.7	*CEACAM3*	18.8	*COL1A1*	19.2	*ANKRD25*
		18.9	*MPO*	18.9	*COL8A1*	19.3	*DMBT1*
		19.0	*CD24*	18.9	*C1QC*	19.4	*MALL*
		19.0	*CHI3L1*	18.9	*SFRP2*	19.5	*ANXA8*
		19.0	*DCN*	19.0	*HIG2*	19.5	*SPRY4*
		19.1	*P8*	19.2	*C1QB*	19.7	*ELF3*
		19.1	*CEACAM6*	19.2	*GPRC5A*	19.9	*EHD2*
		19.1	*ACSL1*	19.3	*MMP25*	20.0	*DCN*
		19.5	*PRTN3*	19.3	*UBD*		
		19.5	*LIF*	19.3	*GADD45A*		
		19.6	*LTF*	19.4	*ISLR*		
		19.7	*ANXA3*	19.5	*ORM1*		
		19.7	*C1R*	19.5	*C20orf114*		
		19.7	*MUC1*	19.5	*LOC203510*		
		19.8	*PSG4*	19.6	*DCN*		
		19.9	*HP*	19.7	*FN1*		
				19.8	*DAAM2*		
				19.9	*FOLR3*		

^a^Data are from patients with stable chronic obstructive pulmonary disease (Stable) or acute exacerbation of chronic obstructive pulmonary disease on day 1 (AE-1), day 3 (AE-3) and day 10 (AE-10) of hospital admission, as compared to healthy controls.

**Table 7:** **Genes upregulated >30-fold in peripheral blood mononuclear cells of patients with AECOPD compared to patients with stable COPD** ^**a**^

**AE-1 vs stable**		**AE-3 vs stable**		**AE-10 vs stable**	
**Fold changes**	**Genes**	**Fold changes**	**Genes**	**Fold changes**	**Genes**
37.3	*MMP8*	33.2	*LOC646309*	30.0	*CCDC48*
37.6	*CEACAM5*	34.7	*SERPINE1*	31.9	*LOC653509*
38.6	*PLUNC*	34.9	*FOS*	32.0	*EPAS1*
39.4	*BPIL1*	37.6	*CYR61*	32.2	*CDH5*
40.3	*CYR61*	39.5	*CEACAM5*	34.4	*CLDN5*
45.4	*CEACAM5*	39.6	*PLUNC*	36.3	*SEPP1*
55.2	*CALCA*	40.1	*ARG1*	38.7	*CAV1*
56.0	*VSIG4*	43.5	*BPIL1*	39.2	*CYR61*
103.9	*CA1*	46.0	*CEACAM5*	42.1	*ADH1B*
		85.9	*CALCA*	44.2	*CTGF*
				44.9	*CAV1*
				45.1	*GPRC5A*
				49.8	*SEPP1*
				81.4	*GPX3*

^a^Data are from patients with patients with acute exacerbation of chronic obstructive pulmonary disease on day 1 (AE-1), day 3 (AE-3) and day 10 (AE-10) of hospital admission, as compared to patients with stable chronic obstructive pulmonary disease (Stable).

**Table 8:** **Genes upregulated between 20- and 30-fold in peripheral blood mononuclear cells of patients with AECOPD compared to patients with stable COPD** ^**a**^

**AE-1 vs stable**		**AE-3 vs stable**		**AE-10 vs stable**	
**Fold changes**	**Genes**	**Fold changes**	**Genes**	**Fold changes**	**Genes**
20.1	*MS4A3*	20.5	*GPR97*	20.3	*TIMP3*
21.0	*CEACAM6*	20.6	*ALPL*	20.3	*SLC6A4*
21.1	*SLC25A37*	20.7	*MTHFS*	20.4	*SFTPA2*
21.2	*DCN*	21.0	*FLJ32028*	20.6	*AKAP2*
22.4	*SPP1*	21.4	*ADM*	20.7	*DST*
24.0	*TCN1*	23.3	*ACSL1*	21.2	*TCF21*
24.7	*BPIL1*	23.3	*DCN*	21.5	*ADH1C*
26.4	*SLC25A37*	24.3	*MMP8*	21.6	*SLIT3*
26.6	*CTGF*	24.5	*TCN1*	21.7	*C9orf61*
28.5	*ARG1*	25.3	*FOS*	22.5	*FOSB*
28.6	*FOS*	25.3	*FOSB*	25.5	*MFAP4*
29.5	*SERPINE1*	27.5	*CTGF*	26.0	*GPX3*
		28.3	*BPIL1*	26.5	*DCN*
		28.4	*VSIG4*	26.9	*SFTPB*
				27.6	*FBLN5*
				28.1	*LOC653509*
				28.5	*ADH1C*
				28.7	*SFTPA1*
				28.7	*TIMP3*

^a^Data are from patients with patients with acute exacerbation of chronic obstructive pulmonary disease on day 1 (AE-1), day 3 (AE-3) and day 10 (AE-10) of hospital admission, as compared to patients with stable chronic obstructive pulmonary disease (Stable).

**Table 9:** **Genes upregulated between 15- and 20-fold in peripheral blood mononuclear cells of patients with AECOPD compared to patients with stable COPD** ^**a**^

**AE-1 vs stable**		**AE-3 vs stable**		**AE-10 vs stable**	
**Fold changes**	**Genes**	**Fold changes**	**Genes**	**Fold changes**	**Genes**
15.6	*ADM*	15.2	*LOC387763*	15.0	*VSIG4*
15.8	*DEFA4*	15.2	*MMP25*	15.6	*IL1RL1*
16.2	*DEFA4*	15.3	*USP15*	15.8	*PZP*
16.2	*C1R*	15.5	*C1R*	16.0	*LDB2*
16.9	*GPNMB*	15.8	*KCNJ15*	16.2	*FLJ43663*
17.2	*DCN*	15.9	*GADD45A*	16.5	*N/A*
17.3	*FAM46C*	15.9	*LRRC4*	16.6	*CD55*
17.6	*ALAS2*	16.3	*GLT1D1*	16.8	*CXCL2*
17.6	*CALCA*	16.4	*CD55*	16.9	*IL1RL1*
17.9	*GPNMB*	16.5	*CEACAM6*	17.0	*RHOB*
18.2	*DUSP1*	16.6	*SPP1*	17.1	*DLC1*
18.2	*CEACAM6*	16.7	*SLC25A37*	17.2	*VIPR1*
18.2	*SLC25A37*	17.1	*ORM1*	17.2	*CRYAB*
18.7	*FOS*	17.2	*CALCA*	17.8	*CNN3*
18.9	*SLC25A37*	17.3	*DUSP1*	18.1	*DCN*
		17.5	*CD177*	18.1	*IFI27*
		17.6	*GPNMB*	18.2	*SLIT2*
		17.7	*MS4A3*	18.3	*RASIP1*
		17.8	*DCN*	18.8	*MFAP4*
		17.8	*GPR109A*	19.0	*CAMK2N1*
		17.9	*BASP1*	19.0	*CD55*
		17.9	*IL8RB*	19.5	*AGER*
		18.4	*AQP9*	19.9	*DKFZP686A01247*
		18.7	*DEFA4*		
		18.8	*QPCT*		
		19.0	*PBEF1*		
		19.0	*BASP1*		
		19.0	*CEACAM6*		
		19.2	*GNG10*		
		19.7	*GPNMB*		
		19.7	*GCA*		
		20.0	*RNASE3*		

^a^Data are from patients with patients with acute exacerbation of chronic obstructive pulmonary disease on day 1 (AE-1), day 3 (AE-3) and day 10 (AE-10) of hospital admission, as compared to patients with stable chronic obstructive pulmonary disease (Stable).

**Table 10:** **Genes upregulated more than fivefold in peripheral blood mononuclear cells of patients with AECOPD** ^**a**^

**AE-3 vs AE-1**		**AE-10 vs AE-1**		**AE-10 vs AE-3**	
**Fold changes**	**Genes**	**Fold changes**	**Genes**	**Fold changes**	**Genes**
5.1	*TMEM50A*	10.3	*SUSD2*	10.1	*SLCO2A1*
5.2	*BCL2A1*	10.6	*TCF21*	10.1	*OAS3*
5.3	*C6orf32*	10.6	*FOLR1*	10.1	*C4BPA*
6.0	*PI3*	10.7	*C9orf61*	10.2	*DMBT1*
7.0	*KCNJ15*	10.9	*LOC653107*	10.4	*VSIG2*
7.6	*CISH*	11.3	*AGER*	10.4	*LOC653107*
10.4	*CISH*	12.0	*SLIT2*	10.5	*ITLN2*
10.7	*CISH*	12.7	*ITLN2*	10.7	*CX3CR1*
		12.9	*FLRT3*	10.7	*MSLN*
		13.1	*VIPR1*	10.8	*SOCS2*
		13.2	*SOCS2*	10.9	*LOC653107*
		13.3	*IL1RL1*	11.7	*FOLR1*
		13.4	*LOC653107*	11.7	*GPX3*
		13.8	*C4BPA*	11.8	*CLIC5*
		14.4	*CYP4B1*	11.8	*SLIT2*
		14.4	*LAMA3*	11.9	*LOC653107*
		15.1	*CYP4B1*	12.1	*AQP1*
		15.2	*ADH1C*	12.6	*LOC653509*
		15.7	*MGC35295*	12.6	*ADH1C*
		15.8	*GPX3*	12.7	*ADH1C*
		17.0	*IL1RL1*	12.8	*ADH1B*
		17.9	*MSLN*	12.9	*LAMA3*
		20.0	*ADH1C*	13.6	*IL1RL1*
		22.4	*ADH1B*	13.6	*CYP4B1*
		24.5	*SLC6A4*	13.9	*FAM107A*
		35.3	*FOLR1*	14.2	*LOC653107*
				14.9	*CYP4B1*
				22.0	*MGC35295*
				31.2	*SLC6A4*

^a^Data are from day 1 (AE-1), day 3 (AE-3) and day 10 (AE-10) of hospital admission.

Table 11 lists the number of genes downregulated more than twofold, including 4,516, 2,975, 3,426 and 2,798 genes of PBMCs from patients with stable COPD on AE-1, AE-3 and AE-10, respectively, below controls; 3,207, 4,510 and 5288 genes on AE-1, AE-3 and AE-10, respectively, below stable COPD; 598 genes from AE-3 below AE-1; and 2,162 and 1,918 genes from AE-10 below those from AE-1 and AE-3, respectively. Downregulated genes of PBMCs from patients with stable COPD, AE-1, AE-3 or AE-10 greater than tenfold, between 10- and 8-fold or between 8- and 6-fold below healthy control subjects are listed in Tables 12, 13 and 14, respectively. Downregulated genes of PBMCs from patients at AE-1, AE-3 or AE-10 compared to stable COPD, or among patients with AECOPD, are shown in Tables 15 and 16.

**Table 11:** **Number of downregulated genes in peripheral blood mononuclear cells of healthy control subjects, patients with stable COPD and patients with AECOPD** ^**a**^

	**Fold changes in upregulated genes (** ***n*** **)**									
**Compared pairs**	**>2**	**>5**	**>6**	**>8**	**>10**	**>15**	**>20**	**>30**	**>50**	**>100**
Stable vs Con	4,516	135	55	9	4	2	1	0	0	0
AE-1 vs Con	2,975	182	107	47	22	7	4	1	0	0
AE-3 vs Con	3,426	225	149	65	35	11	5	2	0	0
AE-10 vs Con	2,798	124	73	31	16	2	1	1	0	0
AE-1 vs Stable	3,207	33	16	4	4	2	0	0	0	0
AE-3 vs Stable	4,510	125	71	21	8	3	1	0	0	0
AE-10 vs Stable	5,288	445	236	97	49	20	8	3	0	0
AE-3 vs AE-1	598	32	23	17	5	3	2	0	0	0
AE-10 vs AE-1	2,162	261	168	82	43	21	14	10	5	1
AE-10 vs AE-3	1,918	192	130	66	36	15	9	6	4	0

^a^Data are from controls (Con) or patients with stable chronic obstructive pulmonary disease (Stable) or acute exacerbation of chronic obstructive pulmonary disease on day 1 (AE-1), day 3 (AE-3) and day 10 (AE-10) of the hospital admission.

**Table 12:** **Genes downregulated more than tenfold in peripheral blood mononuclear cells of patients with stable COPD or AECOPD compared to healthy control subjects** ^**a**^

**Stable vs Con**		**AE-1 vs Con**		**AE-3 vs Con**		**AE-10 vs Con**	
**Fold changes**	**Genes**	**Fold changes**	**Genes**	**Fold changes**	**Genes**	**Fold changes**	**Genes**
10.7	*EIF3S6*	10.3	*HAND1*	10.2	*GZMK*	10.0	*C21orf7*
10.7	*YLPM1*	10.3	*CD8B*	10.5	*CXCR3*	10.0	*NELL2*
16.1	*TFCP2L1*	10.4	*UBASH3A*	10.6	*AK5*	10.4	*C21orf7*
21.0	*SCP2*	10.8	*TRA@*	10.7	*TRA@*	10.4	*GFI1B*
		10.9	*TRBV3-1*	10.7	*IL24*	10.5	*LOC129293*
		11.2	*CD8B*	10.9	*CD6*	10.5	*LOC123876*
		11.4	*MAL*	10.9	*N/A*	10.7	*HIST1H3H*
		11.4	*LOC643514*	11.2	*KIAA0748*	11.1	*IL24*
		11.5	*NELL2*	11.4	*LCK*	11.4	*GFI1B*
		11.7	*TTC24*	11.5	*CD8B*	11.9	*CRTAC1*
		12.7	*CD8B*	12.3	*APBB1*	11.9	*OR10A4*
		13.1	*LEF1*	12.3	*IL12RB1*	11.9	*SAA3P*
		13.8	*TCF7*	12.5	*TTC24*	12.7	*TTC24*
		14.2	*LOC129293*	12.5	*GFI1B*	14.9	*TFCP2L1*
		14.5	*LOC129293*	12.5	*CRTAC1*	18.6	*SCP2*
		15.6	*TCF7*	12.6	*TRBV3-1*	32.3	*UNQ470*
		16.1	*TCF7*	12.6	*ATG9B*		
		16.8	*CD8B*	12.9	*ABLIM1*		
		21.8	*TFCP2L1*	12.9	*LOC129293*		
		25.4	*CRTAC1*	13.0	*CD8B*		
		27.9	*SCP2*	13.1	*CD28*		
		44.1	*UNQ470*	13.1	*GRAP2*		
				14.3	*UBASH3A*		
				14.4	*CCR7*		
				15.0	*LOC129293*		
				16.0	*CD8B*		
				18.1	*UNQ470*		
				18.7	*SCP2*		
				18.8	*LEF1*		
				19.3	*LEF1*		
				23.5	*CD8B*		
				24.3	*TCF7*		
				25.1	*TCF7*		
				30.4	*TCF7*		
				32.0	*TFCP2L1*		

^a^Data are from patients with stable chronic obstructive pulmonary disease (Stable) or acute exacerbation of chronic obstructive pulmonary disease on day 1 (AE-1), day 3 (AE-3) and day 10 (AE-10) of the hospital admission, as compared to healthy controls (Con).

**Table 13:** **Genes downregulated between eight- and tenfold in peripheral blood mononuclear cells of patients with stable COPD or AECOPD compared to healthy control subjects** ^**a**^

**Stable vs Con**		**AE-1 vs Con**		**AE-3 vs Con**		**AE-10 vs Con**	
**Fold changes**	**Genes**	**Fold changes**	**Genes**	**Fold changes**	**Genes**	**Fold changes**	**Genes**
8.2	*AK5*	8.1	*CD3G*	8.0	*TRBV19*	8.1	*HFE2*
8.6	*TRA@*	8.2	*LY9*	8.0	*OTOA*	8.2	*TRA@*
9.1	*ZC3HAV1*	8.2	*AK5*	8.1	*CD7*	8.6	*UNQ470*
9.3	*MAL*	8.2	*C21orf7*	8.1	*GRAP2*	8.6	*CD248*
9.7	*TMEM50B*	8.2	*TRBC1*	8.1	*TNFRSF25*	8.6	*XG*
		8.3	*ANKDD1A*	8.2	*C21orf7*	8.7	*ATG9B*
		8.4	*CD6*	8.2	*EPHA6*	8.8	*LOC339778*
		8.4	*RPS6KB1*	8.2	*GIMAP5*	8.9	*TCF7*
		8.5	*TMEM50B*	8.3	*1-Sep*	8.9	*CCR7*
		8.7	*YLPM1*	8.3	*UBASH3A*	9.2	*LOC644663*
		8.7	*TRBV19*	8.4	*GIMAP7*	9.4	*LOC129293*
		8.8	*FLT3LG*	8.5	*MGC23244*	9.5	*MGC39606*
		8.9	*N/A*	8.6	*LOC645852*	9.7	*GZMK*
		9.1	*LEF1*	8.7	*SCAP1*	9.9	*AK5*
		9.1	*GZMK*	9.0	*HIST1H3H*	9.9	*TCF7*
		9.1	*KIAA0748*	9.0	*HFE2*		
		9.2	*ABLIM1*	9.2	*GFI1B*		
		9.5	*C21orf7*	9.2	*TMEM50B*		
		9.5	*ATG9B*	9.5	*N/A*		
		9.6	*LCK*	9.5	*C21orf7*		
		9.6	*LOC647353*	9.6	*GATA3*		
		9.8	*CCR7*	9.7	*C21orf7*		
		9.8	*UNQ470*	9.7	*CD247*		
		9.9	*OR10A4*	9.8	*LCK*		
		9.9	*IL12RB1*	9.8	*KSP37*		
				9.9	*FAIM3*		
				9.9	*SPOCK2*		
				9.9	*TRA@*		
				9.9	*SH2D1B*		
				10.0	*GRAP2*		

^a^Data are from patients with stable chronic obstructive pulmonary disease (Stable) or acute exacerbation of chronic obstructive pulmonary disease on day 1 (AE-1), day 3 (AE-3) and day 10 (AE-10) of the hospital admission, as compared to healthy controls (Con).

**Table 14:** **Genes downregulated between six- and eightfold in peripheral blood mononuclear cells of patients with stable COPD or AECOPD compared to healthy control subjects** ^**a**^

**Stable vs Con**		**AE-1 vs Con**		**AE-3 vs Con**		**AE-10 vs Con**	
**Fold changes**	**Genes**	**Fold changes**	**Genes**	**Fold changes**	**Genes**	**Fold changes**	**Genes**
6.0	*NDUFV3*	6.0	*MAL*	6.0	*IL7R*	6.0	*HKDC1*
6.0	*C17orf45*	6.0	*ARHGAP12*	6.0	*CD28*	6.1	*TANC2*
6.1	*MAL*	6.0	*TRAPPC4*	6.0	*KIR2DS1*	6.1	*FAM5B*
6.1	*CXCR6*	6.0	*GNLY*	6.0	*FLJ20647*	6.2	*KIAA0748*
6.1	*SUCLA2*	6.0	*N/A*	6.1	*N/A*	6.3	*CD40LG*
6.1	*C21orf7*	6.0	*LOC642376*	6.1	*CLDN1*	6.3	*PCDH10*
6.2	*TNPO1*	6.0	*MYOZ3*	6.1	*TRBV5-4*	6.3	*LOC644273*
6.2	*LOC643514*	6.1	*FLJ20647*	6.1	*CARD11*	6.3	*CD96*
6.2	*ALS2CR13*	6.1	*CD96*	6.1	*LOC441320*	6.3	*TRA@*
6.2	*CREB1*	6.2	*MAL*	6.1	*ACADSB*	6.3	*TRBV3-1*
6.2	*C17orf45*	6.2	*GIMAP5*	6.1	*NXPH4*	6.4	*TRA@*
6.3	*NELL2*	6.2	*CLDN1*	6.2	*SCNN1D*	6.4	*LOC642483*
6.3	*C6orf32*	6.2	*CD3D*	6.2	*MTMR1*	6.5	*ANKDD1A*
6.3	*LOC642455*	6.2	*LY9*	6.2	*MAL*	6.5	*N/A*
6.4	*GMDS*	6.3	*LOC123876*	6.2	*ZAP70*	6.5	*N/A*
6.4	*ABHD6*	6.3	*TNFRSF25*	6.3	*MAL*	6.5	*LY9*
6.4	*DAPP1*	6.3	*C21orf7*	6.3	*IL2RB*	6.6	*CD8B*
6.4	*SH3BGRL*	6.3	*LOC645885*	6.3	*EDG8*	6.6	*MGC26597*
6.5	*IL7R*	6.3	*BLOC1S3*	6.3	*HKDC1*	6.7	*TRBV19*
6.6	*LOC441601*	6.3	*LOC644727*	6.3	*SCAP1*	6.7	*LOC145783*
6.6	*GPR18*	6.4	*CCDC45*	6.3	*LOC440455*	6.8	*CD8B*
6.7	*P2RX5*	6.4	*C21orf7*	6.3	*CD300E*	6.9	*C21orf7*
6.7	*LY9*	6.5	*CD28*	6.4	*LY9*	6.9	*UBASH3A*
6.8	*GGPS1*	6.5	*LOC440455*	6.4	*KIR2DS2*	7.0	*LOC400768*
6.8	*EIF3S6*	6.5	*IL24*	6.4	*SLAMF6*	7.1	*CD8B*
6.8	*ARHGAP15*	6.5	*GHRL*	6.4	*SAA3P*	7.1	*HAND1*
6.8	*SF3B1*	6.5	*FAM113B*	6.4	*SF3A2*	7.2	*LOC126075*
6.8	*GPR89A*	6.5	*LOC644663*	6.5	*UNQ470*	7.2	*TNFRSF7*
6.9	*LOC129293*	6.5	*C15orf37*	6.5	*C6orf21*	7.3	*LEF1*
6.9	*CPNE3*	6.5	*MAL*	6.6	*CD96*	7.3	*HLA-DOA*
6.9	*LY9*	6.5	*LOC644445*	6.6	*CD244*	7.4	*LOC646279*
7.0	*PIP3-E*	6.6	*LOC126075*	6.6	*N/A*	7.4	*YLPM1*
7.0	*TAF9*	6.6	*1-Sep*	6.6	*KLRK1*	7.4	*LOC643514*
7.0	*N/A*	6.6	*UBASH3A*	6.6	*C16orf5*	7.5	*MTMR1*
7.0	*KIAA0748*	6.7	*SAA3P*	6.6	*TRBC1*	7.6	*NOG*
7.1	*CD55*	6.8	*CD6*	6.6	*LOC339778*	7.7	*TCF7*
7.2	*EIF3S6*	6.8	*TRBV5-4*	6.7	*GNLY*	7.7	*KIAA0748*
7.2	*PGRMC2*	6.9	*1-Sep*	6.7	*LDLRAP1*	7.7	*C21orf7*
7.3	*C21orf7*	6.9	*LOC129293*	6.8	*HAND1*	7.7	*PRDM9*
7.4	*PSMD6*	7.0	*SCNN1D*	6.8	*CD3D*	7.7	*FCER2*
7.5	*ABLIM1*	7.0	*SIT1*	6.8	*FLJ45825*	7.9	*CD8B*
7.6	*STAG2*	7.1	*GATA3*	6.8	*SF3A2*	8.0	*LEF1*
7.8	*CCDC45*	7.1	*CD7*	6.8	*CXCR3*		
7.8	*UNQ470*	7.1	*CDKN3*	6.8	*KIR3DL3*		
7.9	*LY9*	7.2	*SCAP1*	6.8	*LAT*		
8.0	*CD40LG*	7.3	*TRA@*	6.9	*CD52*		
		7.3	*LY9*	6.9	*TNFRSF7*		
		7.3	*DDAH1*	6.9	*LOC442726*		
		7.3	*TRA@*	6.9	*3-Sep*		
		7.5	*TNFRSF7*	6.9	*KIAA0748*		
		7.5	*KIAA0748*	6.9	*XG*		
		7.6	*ITM2A*	6.9	*KIAA1549*		
		7.6	*CD5*	7.0	*RNF157*		
		7.6	*D4S234E*	7.0	*SIT1*		
		7.6	*CD300E*	7.0	*CD1C*		
		7.7	*APBB1*	7.0	*SLC16A10*		
		7.8	*CD3D*	7.0	*CD3G*		
		7.8	*LCK*	7.1	*CD6*		
		7.8	*UBASH3A*	7.1	*LY9*		
		7.9	*XG*	7.1	*FLT3LG*		
				7.1	*LOC647353*		
				7.2	*LOC123876*		
				7.2	*CX3CR1*		
				7.2	*LOC126075*		
				7.3	*NELL2*		
				7.4	*LY9*		
				7.4	*MAL*		
				7.4	*KIR2DS2*		
				7.4	*CHIA*		
				7.4	*BIN1*		
				7.5	*CCDC78*		
				7.5	*MAL*		
				7.5	*C21orf7*		
				7.5	*KIR2DL4*		
				7.6	*CD6*		
				7.6	*CD3D*		
				7.7	*1-Sep*		
				7.7	*LCK*		
				7.8	*ITM2A*		
				7.8	*TRA@*		
				7.9	*SIT1*		
				7.9	*CD5*		
				8.0	*CD8A*		
				8.0	*LOC129293*		

^a^Data are from patients with stable chronic obstructive pulmonary disease (Stable) or acute exacerbation of chronic obstructive pulmonary disease on day 1 (AE-1), day 3 (AE-3) and day 10 (AE-10) after the hospital admission, as compared to healthy controls (Con).

**Table 15:** **Genes downregulated more than fivefold in peripheral blood mononuclear cells of patients with AECOPD compared to patients with stable COPD** ^**a**^

**AE-1 vs Stable**		**AE-3 vs Stable**		**AE-10 vs Stable**	
**Fold changes**	**Genes**	**Fold changes**	**Genes**	**Fold changes**	**Genes**
5.0	*PRODH*	10.2	*KSP37*	10.1	*LOC646781*
5.1	*MT1F*	10.3	*DUB3*	10.1	*LOC389634*
5.1	*OR2A7*	10.6	*DUB3*	10.1	*LOC441056*
5.3	*CD8B*	10.8	*TCF7*	10.1	*LOC340243*
5.4	*CGI-38*	11.2	*CX3CR1*	10.2	*C1QL2*
5.4	*DMBT1*	17.6	*MGC35295*	10.2	*LOC653541*
5.4	*N/A*	19.9	*STAC2*	10.2	*LOC158318*
5.4	*GNLY*	25.0	*AZGP1*	10.3	*N/A*
5.5	*LCK*			10.4	*LOC644373*
5.5	*DZIP1*			10.6	*SPDEF*
5.6	*TCF7*			10.7	*DUX1*
5.6	*MGC45438*			10.9	*LOC643001*
5.6	*UNQ470*			11.1	*LOC391767*
5.8	*MGLL*			11.2	*LOC645509*
5.8	*B4GALNT3*			11.7	*FLJ36131*
5.9	*CGI-38*			11.8	*LOC441323*
5.9	*CGI-38*			11.9	*LOC440015*
6.1	*LOC388886*			11.9	*LOC441812*
6.1	*GNLY*			12.0	*TCEB3C*
6.2	*N/A*			12.1	*SPDEF*
6.4	*CD8B*			12.3	*DUX4*
6.4	*AEBP2*			12.5	*LOC285697*
6.4	*EDG8*			12.9	*LOC646066*
6.5	*PRDM16*			13.3	*LOC441873*
6.8	*CX3CR1*			13.6	*LOC645402*
7.0	*MGC45438*			13.7	*LOC285563*
7.3	*MST1*			13.9	*LOC391763*
7.4	*LOC644088*			14.4	*DUB3*
7.5	*EDG8*			14.7	*LOC391766*
10.1	*MGC45438*			15.0	*LOC392197*
12.6	*MGC35295*			15.0	*REXO1L2P*
15.5	*STAC2*			15.2	*DUB3*
19.1	*AZGP1*			15.2	*LOC402199*
				15.7	*LOC653442*
				15.8	*LOC653455*
				16.0	*LOC402207*
				16.5	*LOC391745*
				16.7	*LOC392188*
				18.1	*REXO1L6P*
				19.1	*LOC391764*
				19.4	*DUB3*
				20.6	*LOC645836*
				21.0	*LOC391749*
				23.8	*LOC402110*
				24.2	*REXO1L7P*
				29.6	*REXO1L1*
				30.0	*STAC2*
				33.5	*REXO1L3P*
				39.7	*REXO1L5P*

^a^Data are from patients acute exacerbation of chronic obstructive pulmonary disease on day 1 (AE-1), day 3 (AE-3) and day 10 (AE-10) after the hospital admission, as compared to patients with stable chronic obstructive pulmonary disease (Stable).

**Table 16:** **Genes downregulated more than fivefold in peripheral blood mononuclear cells of patients with AECOPD** ^**a**^

**AE-3 vs AE-1**		**AE-10 vs AE-1**		**AE-10 vs AE-3**	
**Fold changes**	**Genes**	**Fold changes**	**Genes**	**Fold changes**	**Genes**
5.0	*ITGB3*	10.2	*MPO*	10.3	*MOXD1*
5.1	*CGI-69*	10.4	*LOC653492*	10.3	*LOC152573*
5.2	*SPTB*	10.5	*SPP1*	10.7	*SPDEF*
5.2	*BCL2L1*	10.6	*ANK1*	10.8	*CCDC80*
5.2	*GATA1*	11.0	*DEFA4*	11.0	*CTSG*
5.3	*FBXO7*	11.0	*MOXD1*	11.0	*CAMP*
5.6	*SELENBP1*	11.0	*HIG2*	11.3	*PLA2G2D*
5.8	*OSBP2*	11.1	*OSBP2*	11.4	*SPP1*
5.9	*LOC643855*	11.2	*REXO1L3P*	11.6	*S100P*
6.1	*ERAF*	11.6	*SPDEF*	11.7	*SLC4A11*
6.2	*EPB49*	12.0	*COL1A1*	11.8	*COL3A1*
6.2	*MYH9*	12.2	*BPI*	11.8	*SPAG4*
6.4	*ALAS2*	12.3	*SNCA*	12.5	*THBS2*
7.4	*LOC644462*	12.3	*SLC4A11*	12.7	*MPO*
7.8	*GMPR*	12.5	*COL1A1*	13.0	*PRTN3*
8.1	*ANK1*	12.6	*AZU1*	13.2	*COL1A1*
8.9	*BPGM*	12.6	*ARG1*	13.3	*ELA2*
9.1	*FAM46C*	13.2	*GREM1*	14.3	*LIF*
9.2	*LOC643497*	13.5	*DEFA4*	14.4	*CEACAM5*
9.4	*TRIM58*	13.5	*ELA2*	14.6	*RNF183*
9.4	*MBNL3*	14.2	*CEACAM5*	14.9	*B3Gn-T6*
9.5	*EPB49*	14.5	*ITGA11*	15.1	*AZU1*
9.6	*EPB49*	15.0	*CEACAM8*	15.4	*ITGA11*
9.6	*EPB42*	15.3	*SPTB*	15.9	*DEFA4*
9.7	*EPB41*	15.6	*CEACAM5*	16.4	*CEACAM5*
9.7	*SLC14A1*	16.6	*LIF*	17.5	*MS4A3*
9.9	*EPB42*	17.1	*TRIM58*	17.8	*ARG1*
10.1	*SNCA*	19.2	*THY1*	20.4	*THY1*
13.5	*TRIM58*	19.5	*MS4A3*	21.1	*MS4A3*
19.7	*SLC4A1*	23.1	*TRIM58*	22.5	*SPP1*
20.7	*EPB41*	24.1	*MS4A3*	34.4	*SFRP2*
21.6	*CA1*	27.1	*SFRP2*	49.9	*PLUNC*
		29.9	*EPB42*	57.1	*CALCA*
		30.4	*SPP1*	68.9	*CALCA*
		41.9	*ALAS2*	80.4	*BPIL1*
		43.8	*EPB42*	93.1	*BPIL1*
		44.2	*CALCA*		
		48.5	*PLUNC*		
		55.5	*SLC4A1*		
		58.6	*CALCA*		
		70.0	*BPIL1*		
		84.3	*BPIL1*		
		109.9	*CA1*		

^a^Data are from patients with acute exacerbation of chronic obstructive pulmonary disease on day 1 (AE-1), day 3 (AE-3) and day 10 (AE-10) after the hospital admission.

### COPD-specific genes

To search for COPD-specific genes, co–differentially expressed genes of PBMCs from patients with stable COPD or AECOPD were compared with those from control subjects (listed in Additional file 4). There were five groups and four comparison pairs with information regarding fold changes and regulation (that is, SEQ-ID, fold change, log or absolute fold change, or regulation), normalized intensities or annotations (that is, GENE_NAME, synonyms, description, NCBI_GENE_ID, chromosome, GO, UniGene ID, TIGRID or Ensembl ID). Seventy-nine genes were upregulated and 23 genes downregulated in PBMCs from patients with COPD, including both stable COPD and AECOPD, as compared to the healthy control subjects, as shown in Table 17. Of them, 14 genes were upregulated and 2 were downregulated more than tenfold, as compared to control subjects, including carcinoembryonic antigen–related cell adhesion molecule 1, collagen type VIα3(VI), collagen type I(α)2(I), nucleolar protein 3 (apoptosis repressor with CARD domain), melanophilin, cell surface–associated mucin 1, nuclear protein 1, chemokine (C-X-C motif) ligand 17, claudin 4, ribonuclease 1, imprinted maternally expressed transcript, defensin α1, transcription factor CP2-like 1 and sterol carrier protein 2 (*SCP2*).

**Table 17:** **Number and details of co–differentially up- or downregulated genes in peripheral blood mononuclear cells of patients with stable COPD or AECOPD compared to healthy control subjects** ^**a**^

Fold change	>5		>10			
Upregulated	79		14			
Downregulated	23		2			
Unexpressed genes (>10)						
**SEQ-ID**	**Gene name**	**Full name of gene**	**Stable vs Con**	**AE-1 vs Con**	**AE-3 vs Con**	**AE-10 vs Con**
D12502	*CEACAM1*	Carcinoembryonic antigen-related cell adhesion molecule 1	10.1	83.0	66.5	10.5
NM_004369	*COL6A3*	Collagen, type VI, α3	10.4	21.0	22.4	10.8
AF064599	*NOL3*	Nucleolar protein 3 (apoptosis repressor with CARD domain)	12.1	13.6	16.3	11.5
BC042586	*COL1A2*	Collagen, type I, α2	13.1	72.3	92.9	17.2
BC014473	*CEACAM1*	Carcinoembryonic antigen-related cell adhesion molecule 1	14.7	101.2	61.0	11.8
AY358857	*MLPH*	Melanophilin	17.0	10.3	12.8	12.2
AF348143	*MUC1*	Mucin 1, cell surface-associated	20.0	19.7	20.1	28.6
NM_012385	*P8*	p8 protein (candidate of metastasis 1)	20.1	19.1	21.3	22.1
BC093946	*UNQ473*	DMC	20.2	45.9	53.1	24.3
NM_001305	*CLDN4*	Claudin 4	23.0	34.4	39.1	20.7
NM_002933	*RNASE1*	Ribonuclease, RNase A family, 1 (pancreatic)	26.9	12.5	15.2	37.2
BC053636	*H19*	H19, imprinted maternally expressed untranslated mRNA	29.5	37.2	28.0	11.8
BC069423	*DEFA1*	Defensin, α1	33.0	96.1	86.7	10.2
XM_928349	*LOC653600*	Similar to neutrophil defensin 1 precursor (HNP-1) (HP-1) (HP1) (defensin, α1)	43.1	115.8	109.4	12.8
Downregulated genes (>5)						
**SEQ-ID**	**Gene name**	**Full name of genes**	**Stable vs Con**	**AE-1 vs Con**	**AE-3 vs Con**	**AE-10 vs Con**
M38056	*HLA-DOA*	Major histocompatibility complex, class II, DOα	5.3	5.9	5.6	7.3
AY209188	*SAA3P*	Serum amyloid A3 pseudogene	5.3	6.7	6.4	11.9
BC069511	*UBASH3A*	Ubiquitin-associated and SH3 domain-containing, A	5.5	10.4	14.3	6.9
AJ421515	*CRTAC1*	Cartilage acidic protein 1	5.6	25.4	12.5	11.9
AL133666	*EPHA6*	EPH receptor A6	5.6	5.8	8.2	5.3
NM_020152	*C21orf7*	Chromosome 21 open reading frame 7	5.7	8.2	9.7	10.4
XM_089384	*TTC24*	Tetratricopeptide repeat domain 24	5.8	11.7	12.5	12.7
NM_006850	*IL24*	Interleukin 24	6.0	6.5	10.7	11.1
AL713701	*C21orf7*	Chromosome 21 open reading frame 7	6.1	9.5	9.5	10.0
XM_931594	*LOC643514*	Hypothetical protein LOC643514	6.2	11.4	5.7	7.4
NM_006159	*NELL2*	NEL-like 2 (chicken)	6.3	11.5	7.3	10.0
NM_002348	*LY9*	Lymphocyte antigen 9	6.7	8.2	7.4	6.5
XM_934852	*LOC129293*	Hypothetical protein LOC129293	6.9	14.5	12.9	9.4
BC062589	*LY9*	Lymphocyte antigen 9	6.9	7.3	7.1	5.5
XM_934149	*KIAA0748*	KIAA0748	7.0	7.5	11.2	6.2
BC008567	*C21orf7*	Chromosome 21 open reading frame 7	7.3	6.3	7.5	7.7
NM_138363	*CCDC45*	Coiled-coil domain containing 45	7.8	6.4	5.9	5.2
BC022101	*UNQ470*	GAAI470	7.8	44.1	18.1	32.3
BC027920	*LY9*	Lymphocyte antigen 9	7.9	6.2	5.8	5.3
BC033896	*AK5*	Adenylate kinase 5	8.2	8.2	10.6	9.9
XM_085151	*YLPM1*	YLP motif containing 1	10.7	8.7	5.1	7.4
NM_014553	*TFCP2L1*	Transcription factor CP2-like 1	16.1	21.8	32.0	14.9
NM_001007098	*SCP2*	Sterol carrier protein 2	21.0	27.9	18.7	18.6

^a^Data are from patients with stable chronic obstructive pulmonary disease (stable) or acute exacerbation of chronic obstructive pulmonary disease on day 1 (AE-1), day 3 (AE-3) and day 10 (AE-10) of the hospital admission, as compared to healthy controls (Con).

### AECOPD-specific genes

To search for AECOPD-specific genes, co–differentially expressed genes of PBMCs from patients with AECOPD on days 1, 3 and 10 were compared to those from either patients with stable COPD or healthy control subjects (listed in Additional file 4). There were five groups and six comparison pairs with information regarding fold changes and regulation (that is, SEQ-ID, fold change, log or absolute fold change, or regulation), normalized intensities or annotations (that is, GENE_NAME, synonyms, description, NCBI_GENE_ID, chromosome, GO, UniGene ID, TIGRID or Ensembl ID). As compared with both patients with stable COPD and healthy control subjects, 58 genes were upregulated more than fivefold and 238 downregulated more than twofold in patients with AECOPD. Of them, eight upregulated (more than tenfold) and eight downregulated (more than threefold) genes are listed in Table 18. These genes include FBJ murine osteosarcoma viral oncogene homologue (*FOS*); interferon α-inducible protein 27 (*IFI27*); cysteine-rich angiogenic inducer 61 (*CYR61*), connective tissue growth factor (*CTGF*); G protein–coupled receptor family C group 5 member A (*GPRC5A*); FBJ murine osteosarcoma viral oncogene homologue B (*FOSB*); decorin (*DCN*); hypothetical LOC387763 (*LOC387763*); killer cell immunoglobulin-like receptor, two domains, short cytoplasmic tail, 2 (*KIR2DS2*); SH2 domain containing 1B (*SH2D1B*); CD8b molecule (*CD8B*); olfactory receptor family 2, subfamily W, member 5 (*OR2W5*); fibroblast growth factor binding protein 2 (*FGF2*); and transcription factor 7 (*TCF7*).

**Table 18:** **Number of co–differentially up- or downregulated genes in peripheral blood mononuclear cells of patients with AECOPD compared to patients with stable COPD and healthy control subjects** ^**a**^

Fold change		>5			>10		
Upregulated		58			8		
Fold change		>2			>3		
Downregulated		238			8		
Selected co–differentially upregulated genes (>10-fold)							
**SEQ_ID**	**Gene name**	**AE-1**		**AE-3**		**AE-10**	
		**AE-1 vs Con**	**AE-1 vs Stable**	**AE-3 vs Con**	**AE-3 vs Stable**	**AE-10 vs Con**	**AE-10 vs Stable**
BC004490	*FOS*	27.4	28.6	33.5	34.9	13.2	13.7
BC015492	*IFI27*	12.3	10.3	13.1	11.0	21.6	18.1
NM_001554	*CYR61*	12.0	40.3	11.2	37.6	11.7	39.2
NM_001901	*CTGF*	35.7	26.6	36.9	27.5	59.3	44.2
NM_003979	*GPRC5A*	21.2	12.6	19.2	11.4	75.8	45.1
NM_006732	*FOSB*	21.7	13.7	40.1	25.3	35.6	22.5
NM_133504	*DCN*	19.0	17.2	19.6	17.8	20.0	18.1
XM_373497	*LOC387763*	41.4	13.5	46.4	15.2	41.3	13.5
Selected co–differentially downregulated genes (>3-fold)							
**SEQ_ID**	**Gene names**	**AE-1**		**AE-3**		**AE-10**	
		**AE-1 vs Con**	**AE-1 vs Stable**	**AE-3 vs Con**	**AE-3 vs Stable**	**AE-10 vs Con**	**AE-10 vs Stable**
AJ002102	*KIR2DS2*	3.7	3.8	7.4	7.6	4.2	4.4
BC022407	*SH2D1B*	3.0	3.7	4.8	5.9	3.1	3.8
BC066595	*SH2D1B*	3.6	3.2	9.9	8.9	3.6	3.2
BC100911	*CD8B*	11.2	4.4	16.0	6.3	7.9	3.1
NM_001004698	*OR2W5*	3.7	3.1	4.7	4.0	3.7	3.1
NM_004931	*CD8B*	10.3	5.3	11.5	5.9	6.6	3.4
NM_031950	*KSP37*	4.8	5.0	9.8	10.2	3.0	3.1
NM_201633	*TCF7*	15.6	5.6	30.4	10.8	8.9	3.2

^a^Data are from acute exacerbation of chronic obstructive pulmonary disease on day 1 (AE-1), day 3 (AE-3) and day 10 (AE-10) of the hospital admission, as compared to patients with stable COPD (Stable) or healthy controls (Con).

### Dynamic change in gene expression in patients with AECOPD

**Figure 3:**
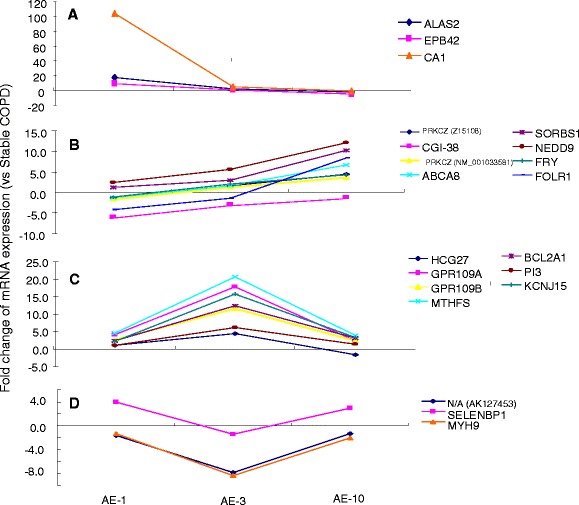
**Dynamic patterns of changes of gene expression of peripheral blood monocytes.** Consistent decrease **(A)** or consistent increase **(B)**, followed by a decrease **(C)**, or a decrease followed by a recovery **(D)**, in patients with acute exacerbation of chronic obstructive pulmonary disease (AECOPD) at day 1 (AE-1), day 3 (AE-3) and day 10 (AE-10) of hospital admission as compared with changes seen in patients with stable COPD.

Dynamic changes (down–down, down–up, up–down and up–up) of co–differentially expressed genes of PBMCs from patients with AECOPD are listed in Additional file 4, including fold changes and regulation (that is, SEQ-ID, fold change, log or absolute fold change, or regulation), normalized intensities or annotations (that is, GENE_NAME, synonyms, description, NCBI_GENE_ID, chromosome, GO, UniGene ID, TIGRID or Ensembl ID). Table 19 shows the dynamic changes in the patterns of down–down (52 genes), down–up (131 genes), up–down (238 genes) and up–up (8 genes) more than twofold, as compared with the gene expression on the previous day. The major genes of PBMCs from patients with AECOPD were aminolevulinate, delta-, synthase 2 (*ALAS2*), erythrocyte membrane protein band 4.2 (*EPB42*) and carbonic anhydrase I (*CA1*) in a down–down pattern; selenium-binding protein 1 (*SELENBP1*) and myosin heavy chain 9, non-muscle (*MYH9*), in a down–up pattern; HLA complex group 27 (*HCG27*), BCL2-related protein A1 (*BCL2A1*), G protein–coupled receptors 109A and 109B (GPR109A and GPR109B) in an up–down pattern; and zeta protein kinase C (*PRKCZ*), ATP-binding cassette, subfamily A, member 8 (*ABCA8*), and folate receptor 1 (adult) (*FOLR1*) in an up–up pattern (Table 19). Levels of genes from patients with AECOPD were also compared with those from patients with stable COPD, as shown in Figure 3, where positive or negative values indicate up- or downregulation as compared with those from patients with stable COPD. When correlated with DESS, *ALAS2* and *CA1* had similar patterns of change with DESS.

**Table 19:** **Number of genes in peripheral blood mononuclear cells of patients with AECOPD** ^**a**^

	**Down–down**		**Down–up**	**Up–down**	**Up–up**
Total	353		784	1,005	127
>2-fold	52		131	238	8
>4-fold	3		3	7	0
>5-fold	2		0	0	0
Selected co–differentially expressed genes at the down–down pattern (>4-fold)					
SEQ-ID	Gene name		Full name of gene	AE-3 vs AE-1	AE-10 vs AE-3
NM_000032	*ALAS2*		Aminolevulinate, delta-, synthase 2	6.4	6.5
BC099627	*EPB42*		Erythrocyte membrane protein band 4.2	9.9	4.4
BC027890	*CA1*		Carbonic anhydrase I	21.6	5.1
Selected co–differentially expressed genes at the down–up pattern (>4-fold)					
SEQ-ID	Gene name		Full name of gene	AE-3 vs AE-1	AE-10 vs AE-3
AK127453	N/A		Homo sapiens cDNA FLJ45545 fis, clone BRTHA2034281.	4.7	5.7
NM_003944	*SELENBP1*		Selenium-binding protein 1	5.6	4.1
BC090921	*MYH9*		Myosin, heavy chain 9, non-muscle	6.2	4.1
Selected co–differentially expressed genes at the up–down pattern (>4-fold)					
SEQ-ID	Gene name		Full name of gene	AE-3 vs AE-1	AE-10 vs AE-3
NM_181717	*HCG27*		HLA complex group 27	4.1	7.3
NM_177551	*GPR109A*		G protein-coupled receptor 109A	4.3	7.5
NM_006018	*GPR109B*		G protein-coupled receptor 109B	4.4	5.1
AF249277	*MTHFS*		5,10-methenyltetrahydrofolate synthetase (5-formyltetrahydrofolate cyclo-ligase)	4.6	5.3
AY234180	*BCL2A1*		BCL2-related protein A1	5.2	4.0
BC010952	*PI3*		Peptidase inhibitor 3, skin-derived (SKALP)	6.0	4.4
NM_002243	*KCNJ15*		Potassium inwardly rectifying channel, subfamily J, member 15	7.0	4.8
Selected co–differentially expressed genes at the up–up pattern (>2-fold)					
SEQ-ID	Gene name		Full name of gene	AE-3 vs AE-1	AE-10 vs AE-3
Z15108	*PRKCZ*		Protein kinase C, zeta	2.0	2.8
BC037798	*CGI-38*		Brain-specific protein	2.0	2.4
NM_001033581	*PRKCZ*		Protein kinase C, zeta	2.1	2.8
NM_007168	*ABCA8*		ATP-binding cassette, subfamily A, member 8	2.1	4.0
AK022468	*SORBS1*		Sorbin and SH3 domain containing 1	2.3	3.5
NM_006403	*NEDD9*		Neural precursor cell expressed, developmentally downregulated 9	2.3	2.2
NM_023037	*FRY*		Furry homologue (*Drosophila*)	2.3	2.1
NM_016730	*FOLR1*		Folate receptor 1 (adult)	3.0	11.7
Down–down	GENE_NAME	SEQ_ID	AE-1 vs Stable	AE-3 vs Stable	AE-10 vs Stable
	*ALAS2*	NM_000032	17.64	2.76	−2.37
	*EPB42*	BC099627	10.02	1.01	−4.37
	*CA1*	BC027890	103.93	4.81	−1.06
Down–up	GENE_NAME	SEQ_ID	AE-1 vs Stable	AE-3 vs Stable	AE-10 vs Stable
	N/A	AK127453	−1.69	−7.90	−1.38
	*SELENBP1*	NM_003944	3.97	−1.41	2.92
	*MYH9*	BC090921	−1.36	−8.40	−2.04
Up–down	GENE_NAME	SEQ_ID	AE-1 vs Stable	AE-3 vs Stable	AE-10 vs Stable
	*HCG27*	NM_181717	1.09	4.47	−1.63
	*GPR109A*	NM_177551	4.12	17.79	2.36
	*GPR109B*	NM_006018	2.64	11.64	2.28
	*MTHFS*	AF249277	4.51	20.75	3.95
	*BCL2A1*	AY234180	2.38	12.45	3.11
	*PI3*	BC010952	1.03	6.20	1.42
	*KCNJ15*	NM_002243	2.25	15.78	3.26
Up–up	GENE_NAME	SEQ_ID	AE-1 vs Stable	AE-3 vs Stable	AE-10 vs Stable
	*PRKCZ*	Z15108	−1.25	1.61	4.46
	*CGI-38*	BC037798	−5.87	−2.86	−1.18
	*PRKCZ*	NM_001033581	−1.61	1.30	3.64
	*ABCA8*	NM_007168	−1.27	1.68	6.69
	*SORBS1*	AK022468	1.28	2.92	10.30
	*NEDD9*	NM_006403	2.43	5.57	12.15
	*FRY*	NM_023037	−1.11	2.08	4.34
	*FOLR1*	NM_016730	−4.20	−1.39	8.39

^a^Data are from acute exacerbation of chronic obstructive pulmonary disease on day 1 (AE-1), day 3 (AE-3) and day 10 (AE-10) of the hospital admission. Comparisons are between AE-1 and AE-3 or between AE-3 and AE-10.

### Gene ontology analysis and pathway analysis

Within ten comparison pairs, up- or downregulated genes mainly involved in the biological process are shown in Figures S3 and S4 of Additional file 2, those in cellular components are shown in Figures S5 and S6 of Additional file 2 and those in molecular functions are shown in Figures S7 and S8 of Additional file 2. Additional file 5 lists gene numbers for ten comparison pairs with certain GO terms and different ranges of enrichment scores.

**Figure 4:**
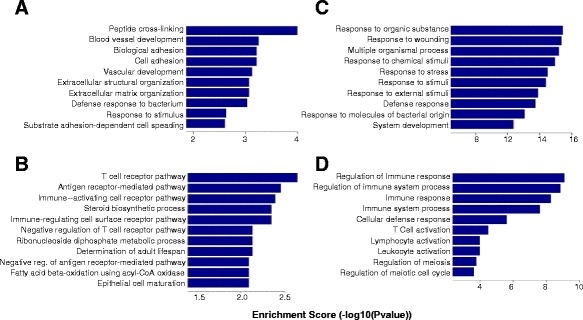
**Gene expression profile comparisons regarding the biological process.** Graphs describe co–differentially upregulated genes **(A)** and downregulated genes **(B)** in the biological process of peripheral blood mononuclear cells from patients with chronic obstructive pulmonary disease (COPD), including those with stable COPD and acute exacerbation of COPD (AECOPD), as compared to healthy control subjects. Also shown are co–differentially expressed upregulated genes **(C)** and downregulated genes **(D)** from patients with AECOPD, as compared to patients with stable COPD and healthy control subjects.

**Figure 5:**
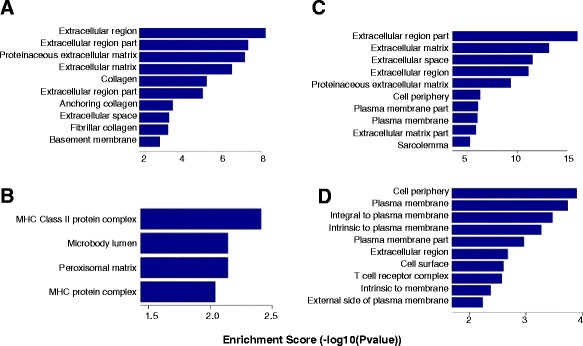
**Gene expression profile comparisons regarding the cellular component.** Graphs describe co–differentially upregulated genes **(A)** or downregulated genes **(B)** in the cellular component of peripheral blood mononuclear cells from patients with chronic obstructive pulmonary disease (COPD), including stable COPD and acute exacerbation of COPD (AECOPD), as compared to healthy control subjects. Also shown are co–differentially expressed upregulated genes **(C)** or downregulated genes **(D)** from patients with AECOPD, as compared to both patients with stable COPD and healthy control subjects. MHC, Major histocompatibility complex.

**Figure 6:**
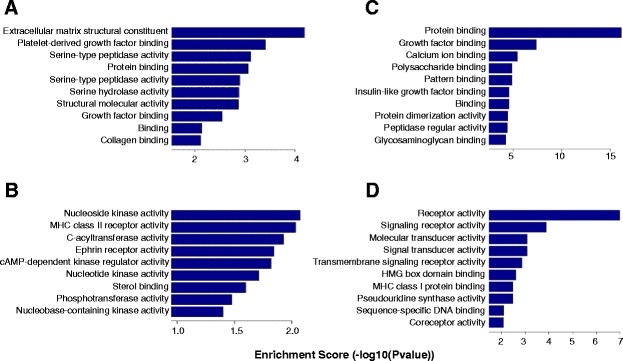
**Gene expression profile comparisons regarding molecular function.** Graphs describe co–differentially upregulated genes **(A)** or downregulated genes **(B)** in the molecular function of peripheral blood mononuclear cells from patients with chronic obstructive pulmonary disease (COPD), including stable COPD and acute exacerbation of COPD (AECOPD), as compared to healthy control subjects. Also shown are co–differentially expressed upregulated genes **(C)** or downregulated genes **(D)** from patients with AECOPD, as compared to both patients with stable COPD and healthy control subjects. MHC, Major histocompatibility complex.

In the biological process, COPD-specific upregulated genes were involved mainly in peptide cross-linking, blood vessel development, biological adhesion or cell adhesion (Figure 4A). COPD-specific downregulated genes were involved mainly in T cell receptor signaling pathways, antigen receptor–mediated signaling pathways, immune response–activating cell surface receptor signaling pathways or steroid biosynthetic process (Figure 4B). AECOPD-specific genes upregulated in response to organic substance, response to wounding, multicellular organismal process or response to chemical stimulus are shown in Figure 4C. AECOPD-specific downregulated genes were involved mainly in the regulation of immune response and the immune system process or in the immune response and immune system process themselves (Figure 4D). In the cellular component, COPD-specific upregulated genes were involved mainly in the extracellular region, the extracellular matrix part, the proteinaceous extracellular matrix or the extracellular matrix (Figure 5A). COPD-specific downregulated genes were involved mainly in the major histocompatibility complex class II (MHC II) protein complex, microbody lumen, peroxisomal matrix or MHC II protein complex (Figure 5B). AECOPD-specific upregulated genes were involved mainly in the extracellular region part, the extracellular matrix, the extracellular space or the extracellular region (Figure 5C). AECOPD-specific downregulated genes were involved mainly in the cell periphery and the plasma membrane and were integral to the plasma membrane and intrinsic to the plasma membrane (Figure 5D). In molecular function, COPD-specific upregulated genes participated mainly in extracellular matrix structural constituent, platelet-derived growth factor binding, serine-type endopeptidase activity and protein binding (Figure 6A). COPD-specific downregulated genes were involved mainly in nucleoside kinase activity, MHC class II receptor activity, C-acyltransferase activity and ephrin receptor activity (Figure 6B). AECOPD-specific upregulated genes were involved mainly in protein binding, growth factor binding, calcium ion binding and polysaccharide binding (Figure 6C). AECOPD-specific downregulated genes were involved mainly in receptor activity, signaling receptor activity, molecular transducer activity and signal transducer activity (Figure 6D).

**Figure 7:**
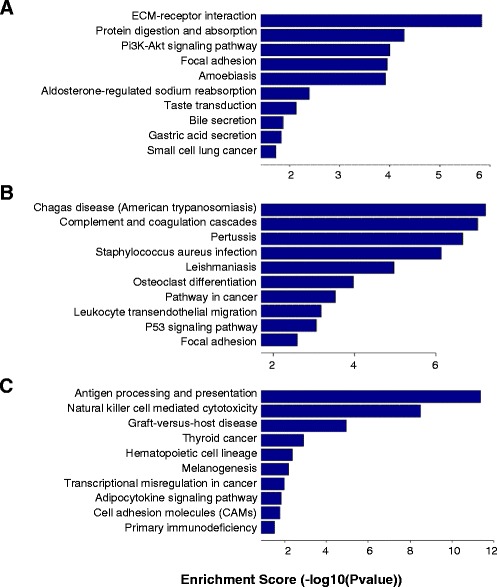
**Gene expression profile comparisons regarding signaling pathways.** Graphs describe co–differentially upregulated genes **(A)** in different pathways of peripheral blood mononuclear cells from patients with chronic obstructive pulmonary disease (COPD), including patients with stable COPD and acute exacerbation of COPD (AECOPD), as compared to healthy control subjects. Also shown are co–differentially expressed upregulated genes **(B)** or downregulated genes **(C)** from patients with AECOPD, as compared to patients with stable COPD and healthy control subjects. ECM, Extracellular matrix; MHC, Major histocompatibility complex; Pi3K, Phosphatidylinositol 3-kinase.

COPD-specific upregulated genes also participated in extracellular matrix receptor interaction, protein digestion and absorption, focal adhesion and the phosphatidylinositol 3-kinase-Akt signaling pathway (Figure 7A). AECOPD-specific upregulated genes participated in Chagas disease, complement and coagulation cascades, pertussis and *Staphylococcus aureus* infection (Figure 7B). AECOPD-specific downregulated genes participated in antigen processing and presentation, natural killer cell–mediated cytotoxicity, graft-versus-host disease and thyroid cancer (Figure 7C).

## Discussion

PBMCs play a critical and important role in the occurrence of AECOPD, owing to less capacity for balancing the proinflammatory immune response caused by infection and for secreting adequate amounts of anti-inflammatory cytokines [22]. The fact that patients with COPD are more susceptible to acute exacerbation has been suggested to be associated with PBMC dysfunction and failure of adaptation to infection, stimuli or hypoxia, although there have been not yet studies on the phenotypes of PBMCs in AECOPD. For example, PBMCs from patients with COPD could not induce hypoxia-inducible factor 1 and vascular endothelial growth factor, owing to a reduction in histone deacetylase 7 under hypoxic condition [23]. It was suggested that overproduction of proinflammatory cytokines (*CXCL6* and interleukin 6 (*IL-6*)) from human PBMCs could be stimulated by the infection through activation of Toll-like receptor 4, nicotinamide adenine dinucleotide phosphate oxidase phosphatidylinositol 3-kinase and nuclear factor κB [24], at least as partial mechanisms by which PBMCs may be involved in the occurrence of AECOPD. The present study provides initial evidence that dynamic alterations of PBMC genetic phenotypes occurred in patients with AECOPD after their hospital admission and during their hospital stay.

Gene expression profiles of PBMCs were investigated in patients with COPD, compared with healthy controls and correlated with lung function measurement [12]. Differential expression of 45 known genes was identified, of which 16 markers had significant correlation with quantitative traits and differential expression between cases and controls and 2 genes, *RP9* and *NAPE-PLD*, were identified as decreased in patients with COPD, as compared to controls, in both lung tissue and blood. Gene expression profiles of PBMCs were recently identified and validated in smokers with and without COPD and corrected with clinical phenotypes such as sex, age, body mass index, family history, smoking status and pack-years of smoking [25]. Of them, 16 candidate genes were found to be associated with airflow obstruction and secondary clinical phenotypes, 12 with emphysema, 13 with gas trapping and 8 with distance walked. Both previous studies demonstrated the gene expression profiles of PBMCs from patients with stable COPD and addressed the potential significance of smoking. In the present study, we selected healthy control subjects and patients who were not current smokers and demonstrated gene expression profiles of PBMCs from patients with COPD, including stable COPD and AECOPD. We addressed COPD-specific gene expression profiles that should appear in both stable COPD and COPD exacerbation conditions and found COPD-specific 79 genes were upregulated and 23 genes down-regulated more than fivefold as compared with gene expression in controls. In the present study, we selected consistent up- or downregulated gene expression on days 1, 3 and 10 of AECOPD-specific as compared with gene expression in both healthy controls and patients with stable COPD, as AECOPD-specific gene expression profiles. We found that 58 AECOPD-specific genes were upregulated more than fivefold and 238 genes were downregulated more than twofold, as compared to both control subjects and patients with stable COPD.

Variation of gene expression profiles is dependent upon multiple uncontrollable factors, such as study population, age, history, genetic background and treatment. In addition, gene expression profiles vary between harvested sample types, such as sputum, bronchoalveolar lavage fluid, blood or lung tissues. For example, 102 genes were identified to distinguish between non- or mild emphysema and severe emphysema in lung tissue [15] and to distinguish 70 microRNAs and 2,667 mRNAs between smoking patients with or without COPD [26]. In the present study, we investigated gene expression profiles of PBMCs from control subjects, patients with stable COPD, and patients with AECOPD on day 1, day 3 and day 10 of hospital admission, and we found about 3,000 overexpressed genes and 2,000 downregulated genes in patients with stable COPD or AECOPD, as compared with control subjects. These findings indicate that those COPD-specific genes exist in the stable COPD condition and during acute exacerbations of COPD.

Of the COPD-specific genes we studied, *CEACAM1*, *COL6A3*, *NOL3*, *COL1A2*, *MLPH*, *MUC1*, *P8*, *UNQ473*, *CLDN4*, *RNASE1*, *H19*, *DEFA1* and *LOC653600* were upregulated more than tenfold, mainly related to nuclear proteins, collagens or molecular structure. We noted that transcription factor CP2 (*TFCP2L1*) and *SCP2* were downregulated more than tenfold. In previous studies, these genes, including *CEACAM1*, *TFCP2L1* and *SCP2*, were not found to be associated with COPD. The *SCP2* gene is located within chromosome 1 and encodes the nonspecific lipid transfer protein *SCP2*, which is involved in organellar fatty acid metabolism [27,28] and the translocation of cytoplasmic free cholesterol to the mitochondria [29]. Our results indicate that PBMCs from patients with stable COPD or AECOPD had downregulated *SCP2*, which might point to severe metabolic disorder and thus that *SCP2* downregulation might contribute to one of the common comorbidities of COPD [30]. *TFCP2* is a member of a family of transcription factors that regulate genes involved in events from early development to terminal differentiation [31]. PBMCs with downregulated *TFCP2* of patients with COPD might have less capacity of the transcriptional switch of globin gene promoters, many other cellular and viral gene promoters, or interaction with certain inflammatory response factors, although the exact mechanism and pathological role remain unclear.

AECOPD-specific gene expression profiles were selected by comparing them with both healthy control subjects and patients with stable COPD, including 647 upregulated genes and 238 downregulated genes (greater than twofold upregulation). Of them, *FOS*, *IFI27*, *CYR61*, *CTGF*, *GPRC5A*, *FOSB*, *DCN* and *LOC387763* were upregulated more than tenfold and *KIR2DS2*, *SH2D1B*, *CD8B*, *OR2W5*, *KSP37* and *TCF7* were downregulated more than threefold.

We noticed that some genes, such as *FOS*, *CYR61* and *CTGF*, were upregulated in PBMCs from patients with either stable COPD or AECOPD, consistent with the lung tissue gene expression profiles of patients with COPD or smokers, in whom the genes were expressed mainly in alveolar epithelial cells, airway epithelial cells and stromal and inflammatory cells [14]. Other genes, including *GPRC5A*, *LOC387763* and *KIR2DS2*, were not found to be associated with AECOPD in previous publications. *CTGF* is a cysteine-rich peptide implicated in several biological processes, such as cell proliferation, survival and migration, and involved in pulmonary vascular remodeling and hypertension in COPD. It was evidenced by the experimental finding that CTGF short-hairpin RNA could significantly prevent CTGF and cyclin D1 expression, arrest cell cycle at the G_0_/G_1_ phase, suppress cell proliferation in smoking-exposed pulmonary smooth muscle cells and ameliorate pulmonary vascular remodeling [32]. Another study demonstrated that some inflammatory genes (*IL-1β*, *IL-6*, *IL-8*, *CCL2* and *CCL8*) were upregulated, whereas some growth factor receptor genes (*BMPR2*, *CTGF*, *FGF1*, *KDR* and *TEK*) were downregulated in lung tissue samples from patients who were current smokers or had moderate COPD [33].

Downregulation of *TCF7* was found in PBMCs of patients with COPD and current smoking and was correlated with some clinical phenotypes, such as emphysema, gas trapping and distance walked [25]. In the present study, we also found that *TCF7* was downregulated in ex-smokers with COPD by about an absolute threefold compared with control subjects, and, in patients with AECOPD, *TCF7* was downregulated by about an absolute tenfold compared with both control subjects and patients with stable COPD. These findings indicate that *TCF7* not only is a COPD-specific gene but also is associated with the severity of the disease. *TCF7* is a member of a family of HMG box containing factors associated with β-catenin to mediate Wnt signaling, controls the switch between cell self-renewal and differentiation and plays a role in B cell and T cell development. *TCF7* was found to be the most downregulated transcription factor when CD34+ cells switched into CD34− cells through a coordinated regulation of the binding between *TCF7* and the short isoforms of *RUNX1* [34]. It is possible the downregulation of *TCF7* and associated regulation may be one part of molecular mechanism of PBMC incapacity during AECOPD.

Dynamic alterations of gene expression profiles in patients with AECOPD were evaluated with dynamic DESS scores. *ALAS2*, *EPB42* and *CA1* were co–differentially expressed with a down–down type in patients with AECOPD. Among these three genes, the *CA1* gene encodes a protein which is important in respiratory function, fluid secretion and maintenance of cellular acid–base homeostasis [35]. The genes with a down–up type included *SELENBP1*, *MYH9* and an unnamed gene in chromosome 19, both of which are associated with psychotic disorders [36,37]. One limitation of the present study is the small sample size, which detracts from the generalizability of the results presented.

## Conclusions

Dynamic alterations of PBMC gene expression profiles were initially investigated in patients with AECOPD, as compared with healthy control subjects or patients with stable COPD. A panel of genes, including eight that were upregulated and eight that were downregulated, were recommended as AECOPD-specific dynamic biomarkers. AECOPD-specific up- or downregulated genes in the biological process, cellular components or molecular function were defined and participated in complement and coagulation cascades, infection, antigen processing and presentation, natural killer cell–mediated cytotoxicity, and/or cancer-causing potential. The integration of dynamic bioinformatics with clinical phenotypes helped us to identify and validate AECOPD-specific biomarkers to help define the severity, duration and response of the disease to therapies.

## Key messages

- Circulating dynamic biomarkers were identified for the specificity and severity of AECOPD.
- A panel of 16 genes were selected as AECOPD-specific biomarkers.
- This is an initial study designed to examine gene expression profiles of peripheral blood mononuclear cells and identify dynamic changes of AECOPD-specific biomarkers.

## Additional files

Additional file 1:
**DESS scores.** This file lists Digital Evaluation Score System (DESS) scores of subjects from each group. Additional file 2:
**Eight supplemental figures. Figure S1.** A box plot showing distributions of log_2_ ratios among groups. They reflect our assessment of the quality of genetic data after the filtering and distribution of data sets. **Figure S2.** Hierarchical clustering shows distinguishable gene expression profiles and relationships between different groups. **Figure S3.** Co–differentially upregulated genes within 10 comparison pairs mainly involved in the biological process. Stable vs Con **(A)**; AE-1 vs Con **(B)**; AE-3 vs Con **(C)**; AE-10 vs Con **(D)**; AE-1 vs Stable **(E)**; AE-3 vs Stable **(F)**; AE-10 vs Stable **(G)**; AE-3 vs AE-1 **(H)**; AE-10 vs AE-1 **(I)**; AE-10 vs AE-3 **(J)**. **Figure S4.** Co–differentially downregulated genes within 10 comparison pairs mainly involved in the biological process. **Figure S5.** Co–differentially upregulated genes within 10 comparison pairs mainly involved in the cellular component. **Figure S6.** Co–differentially downregulated genes within 10 comparison pairs mainly involved in the cellular component. **Figure S7.** Co–differentially upregulated genes within 10 comparison pairs mainly involved in the molecular function. **Figure S8.** Co–differentially downregulated genes within 10 comparison pairs mainly involved in the molecular function. Additional file 3:
**Differentially expressed genes.** This file lists 10 comparison pairs with information of fold changes and regulation, normalized intensities or annotations. Additional file 4:
**Co–differentially expressed genes.** This file lists COPD-specific and AECOPD-specific genes, as well as dynamically changed genes, in patients with AECOPD. Additional file 5:
**Gene Ontology database.** This file lists gene numbers for 10 comparison pairs with certain GO (Gene Ontology) terms and different enrichment score ranges.

## Abbreviations

AE-1: Acute exacerbations of chronic obstructive pulmonary disease on day 1
AE-3: Acute exacerbations of chronic obstructive pulmonary disease on day 3
AE-10: Acute exacerbations of chronic obstructive pulmonary disease on day 10
AECOPD: Acute exacerbation of chronic obstructive pulmonary disease
ALAS2: Aminolevulinate, delta-, synthase 2
CA1: Carbonic anhydrase I
COPD: Chronic obstructive pulmonary disease
CXCL8: Chemokine (C-X-C motif) ligand 8
DESS: Digital evaluation score system
EPB42: Erythrocyte membrane protein band 4.2
FEV_1_: Forced expiratory volume in 1 second
FVC: Forced vital capacity
GO: Gene Ontology
IL: Interleukin
MHC: Major histocompatibility complex
MYH9: Myosin, heavy polypeptide 9, non-muscle
PBMC: Peripheral blood mononuclear cell
SCP2: Sterol carrier protein 2
SELENBP1: Selenium-binding protein 1
TCF7: Transcription factor 7
TFCP2L1: Transcription factor CP2-like 1
