# Supplementary material for: Dynamic gene expressions of peripheral blood mononuclear cells in patients with acute exacerbation of chronic obstructive pulmonary disease: a preliminary study
Source: Crit Care. 2014 Nov 19;18(6):508. doi: 10.1186/s13054-014-0508-y (PMC4305227; doi:10.1186/s13054-014-0508-y)
Supplement: Additional file 2: — Eight supplemental figures. Figure S1. A box plot showing distributions of log2 ratios among groups. They reflect our assessment of the quality of genetic data after the filtering and distribution of data sets. Figure S2. Hierarchical clustering shows distinguishable gene expression profiles and relationships between different groups. Figure S3. Co–differentially upregulated genes within 10 comparison pairs mainly involved in the biological process. Stable vs Con (A); AE-1 vs Con (B); AE-3 vs Con (C); AE-10 vs Con (D); AE-1 vs Stable (E); AE-3 vs Stable (F); AE-10 vs Stable (G); AE-3 vs AE-1 (H); AE-10 vs AE-1 (I); AE-10 vs AE-3 (J). Figure S4. Co–differentially downregulated genes within 10 comparison pairs mainly involved in the biological process. Figure S5. Co–differentially upregulated genes within 10 comparison pairs mainly involved in the cellular component. Figure S6. Co–differentially downregulated genes within 10 comparison pairs mainly involved in the cellular component. Figure S7. Co–differentially upregulated genes within 10 comparison pairs mainly involved in the molecular function. Figure S8. Co–differentially downregulated genes within 10 comparison pairs mainly involved in the molecular function. [file 13054_2014_508_MOESM2_ESM.pdf]

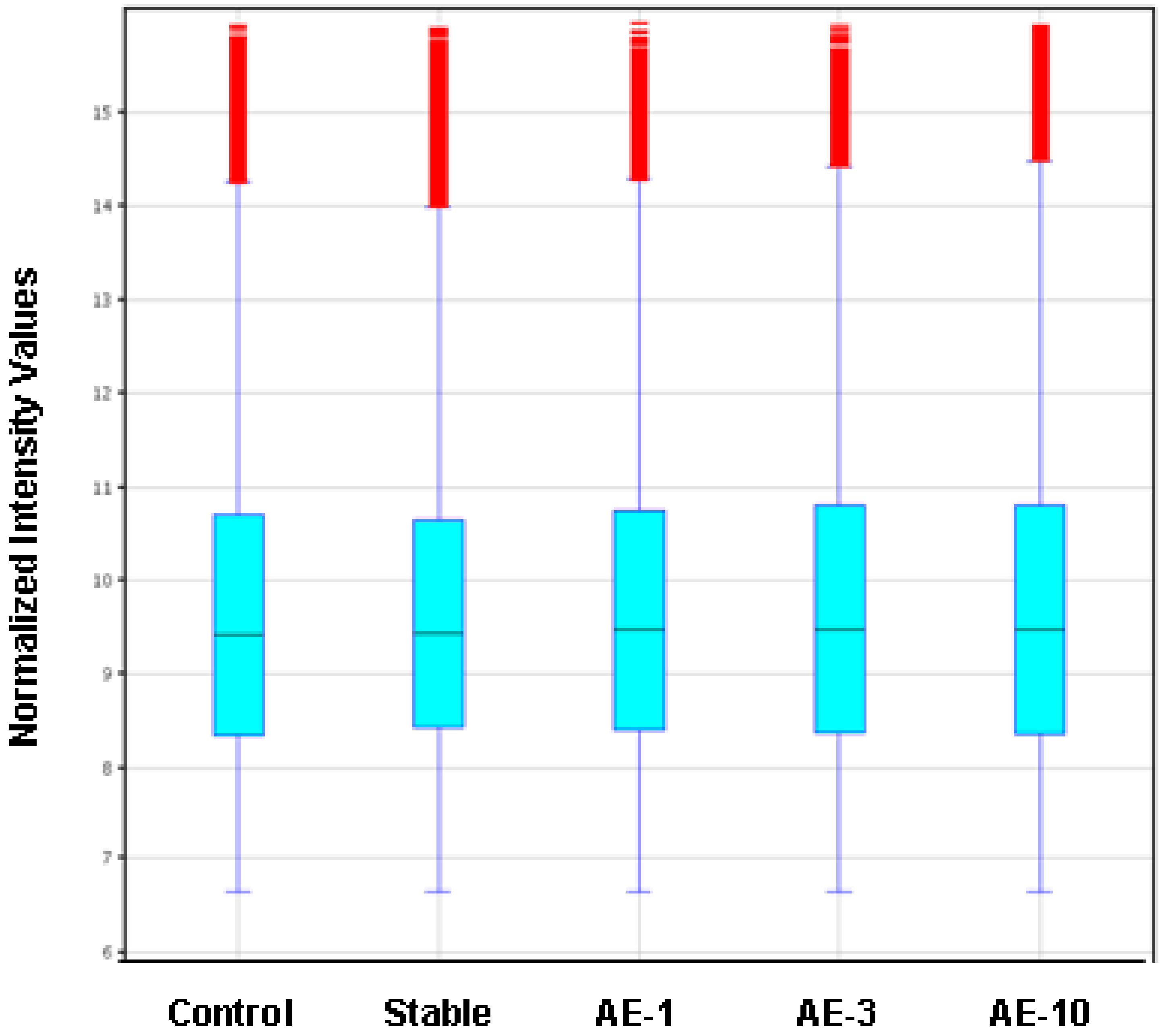


Suppl. Figure 1

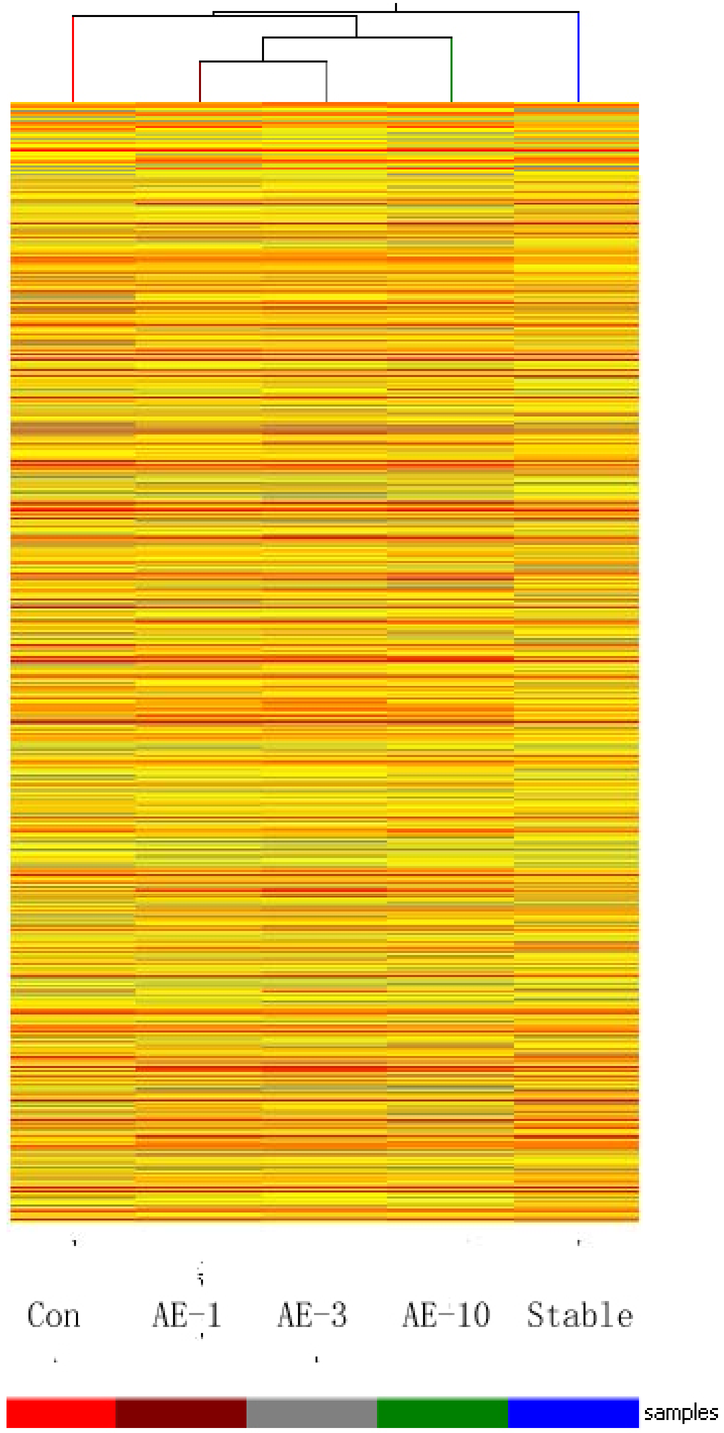


**Suppl. Figure 2**

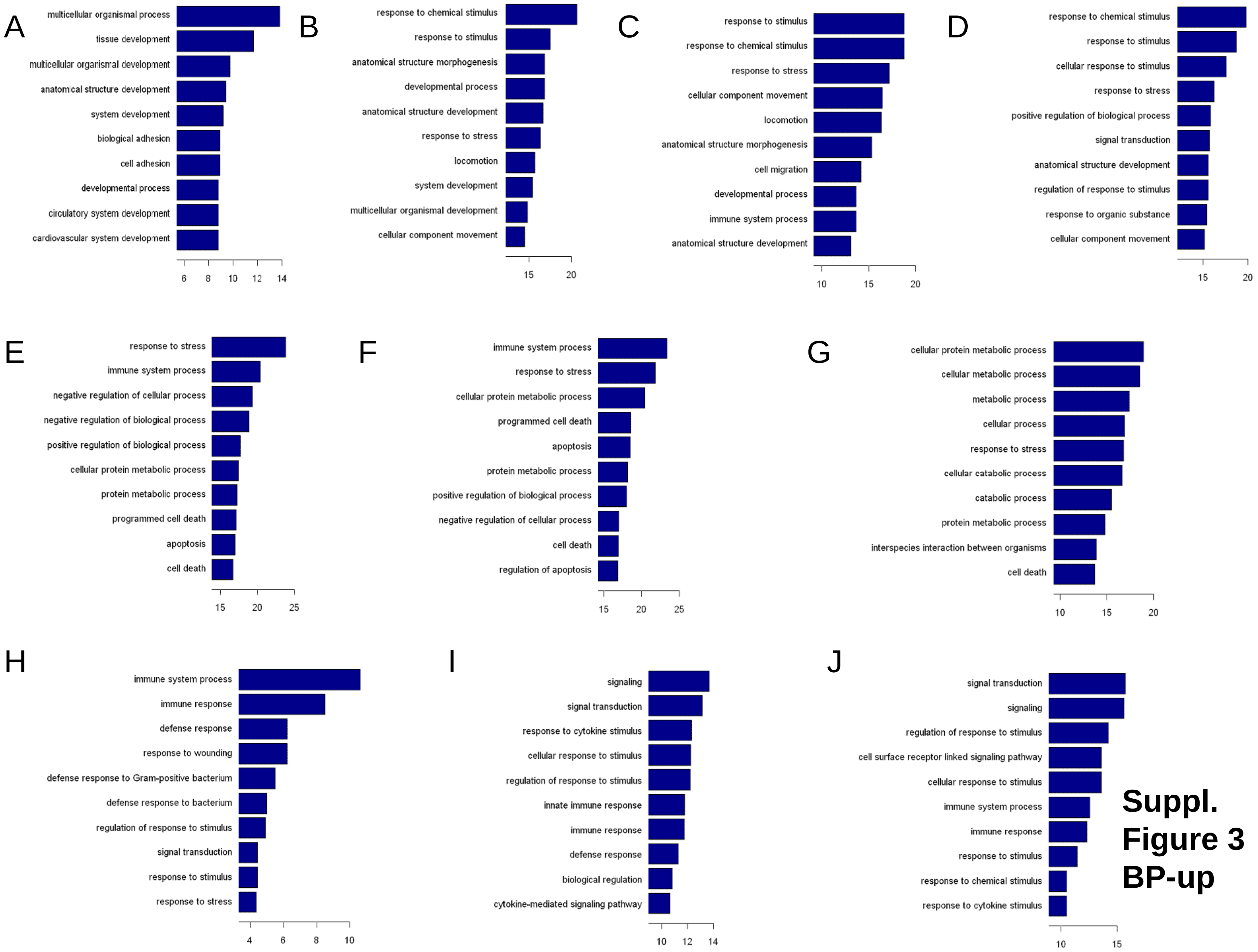


**Suppl. Figure 3 BP-up**

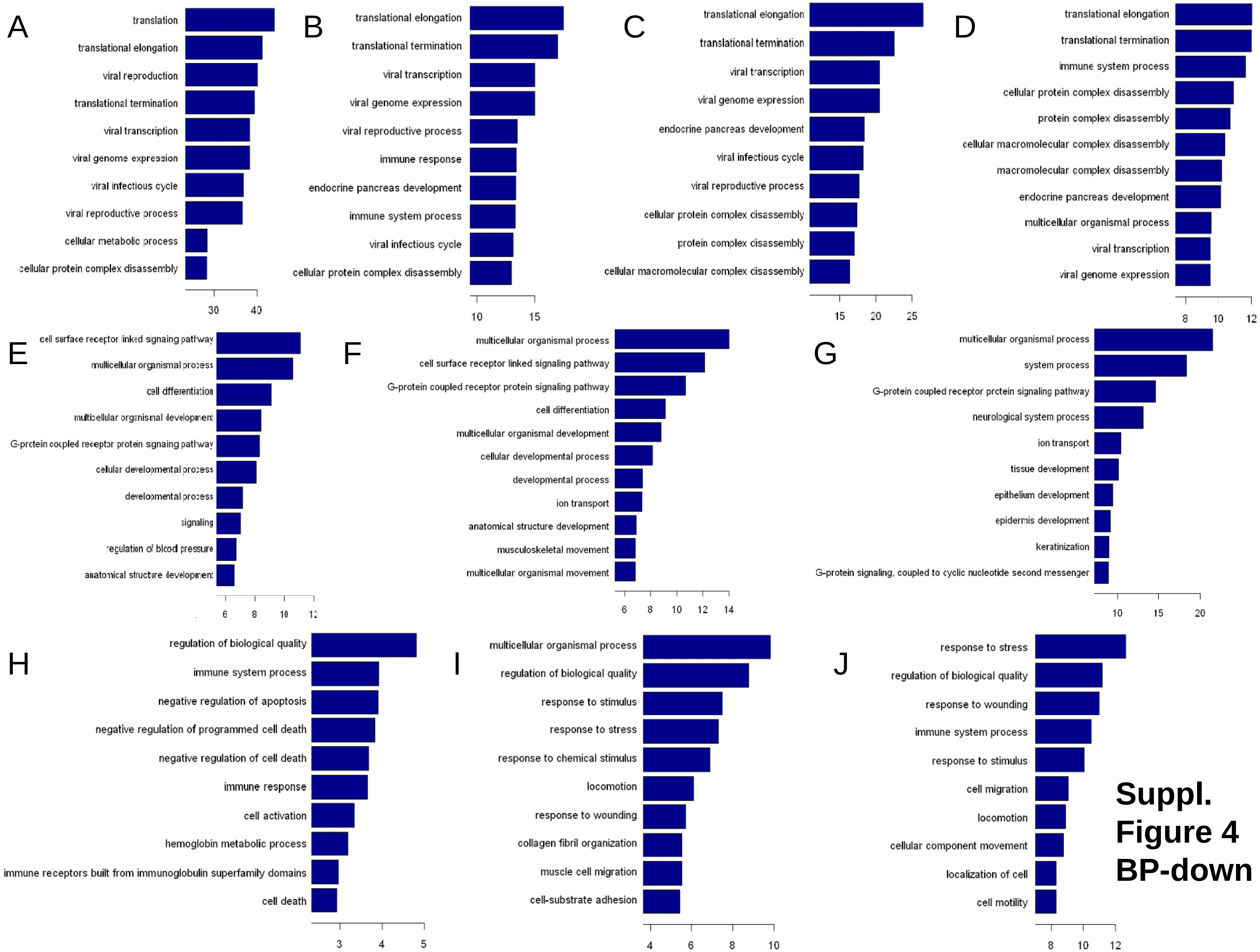


**Suppl. Figure 4 BP-down**

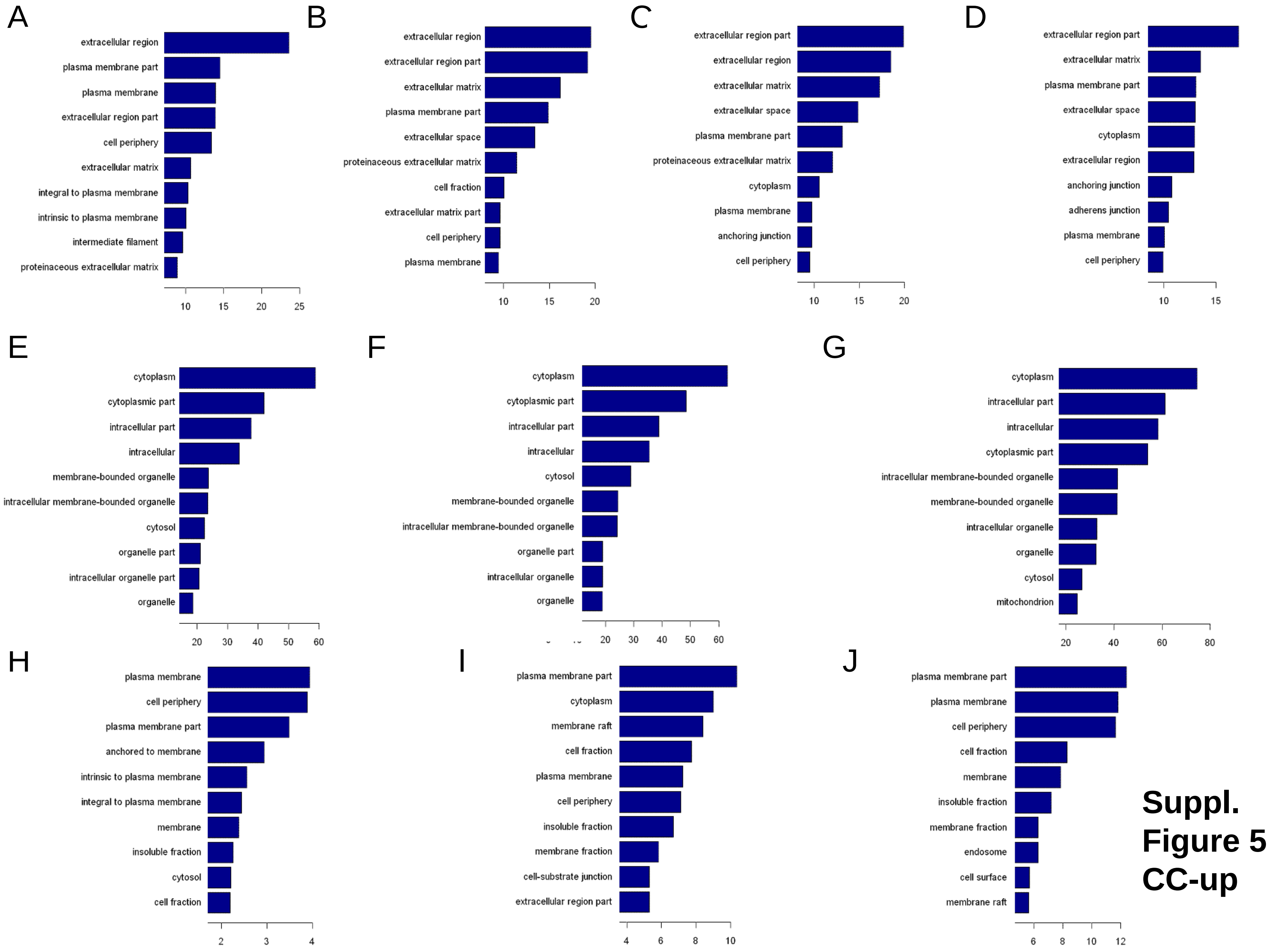


**Suppl. Figure 5 CC-up**

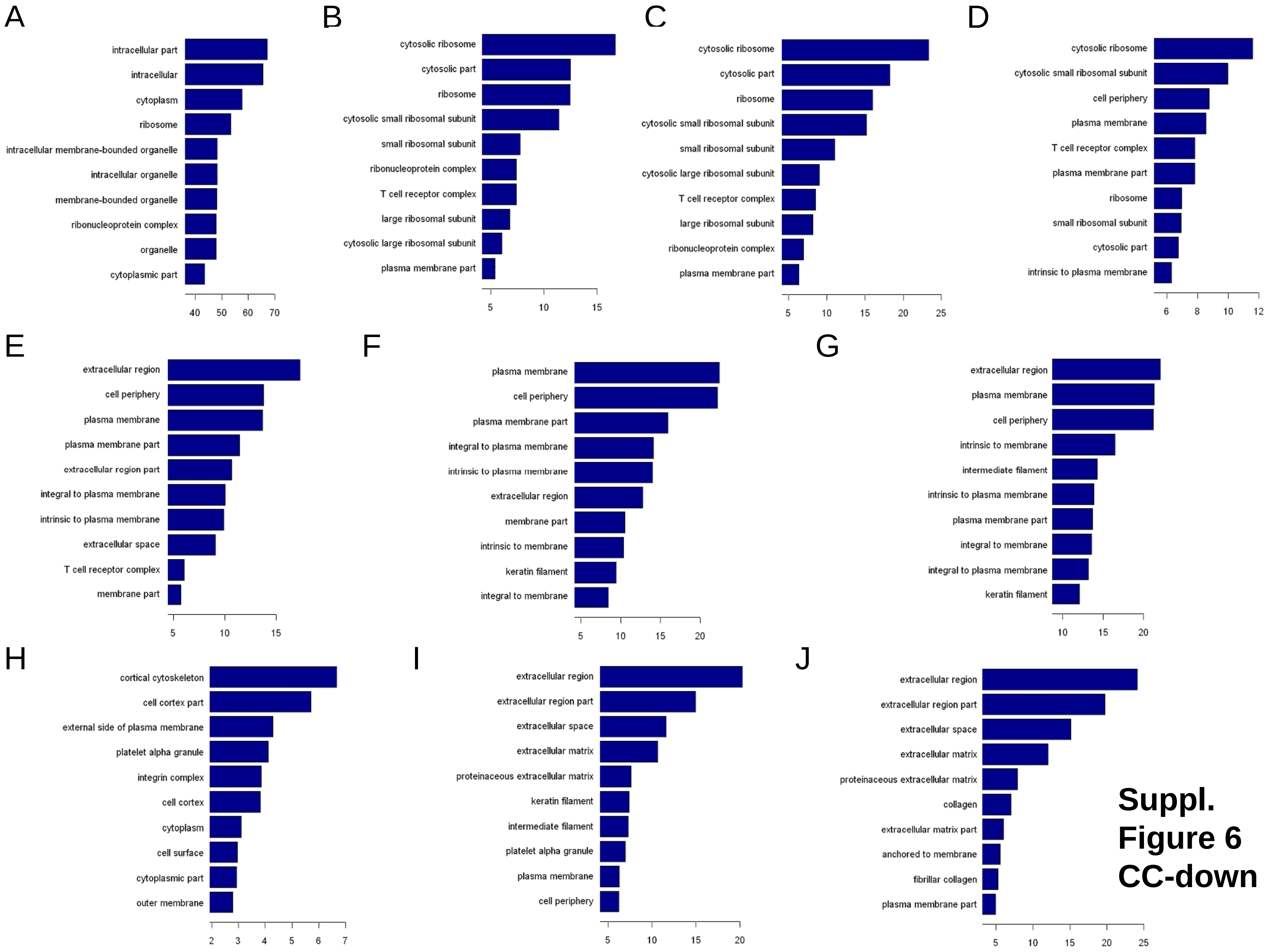


**Suppl. Figure 6 CC-down**

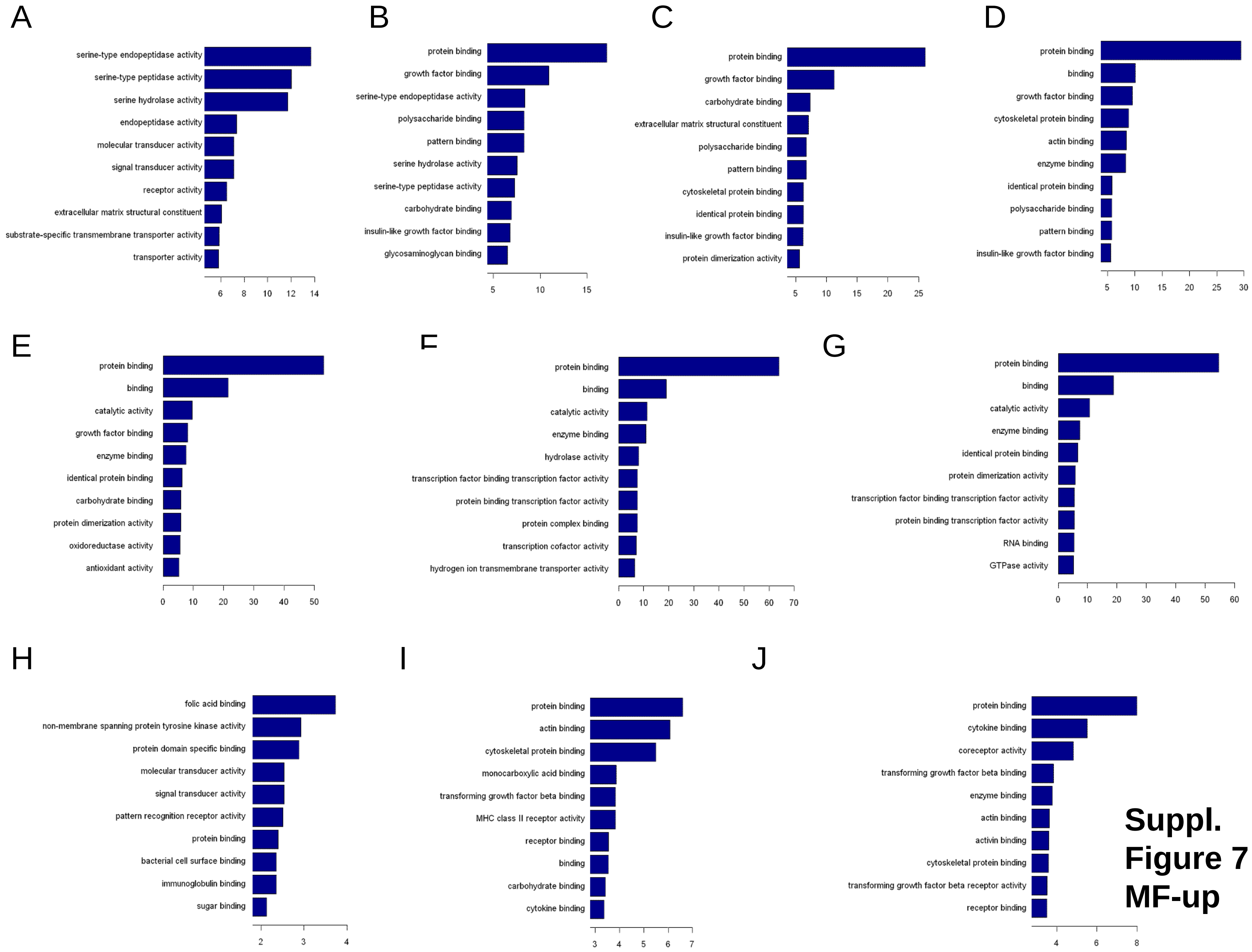


**Suppl. Figure 7 MF-up**

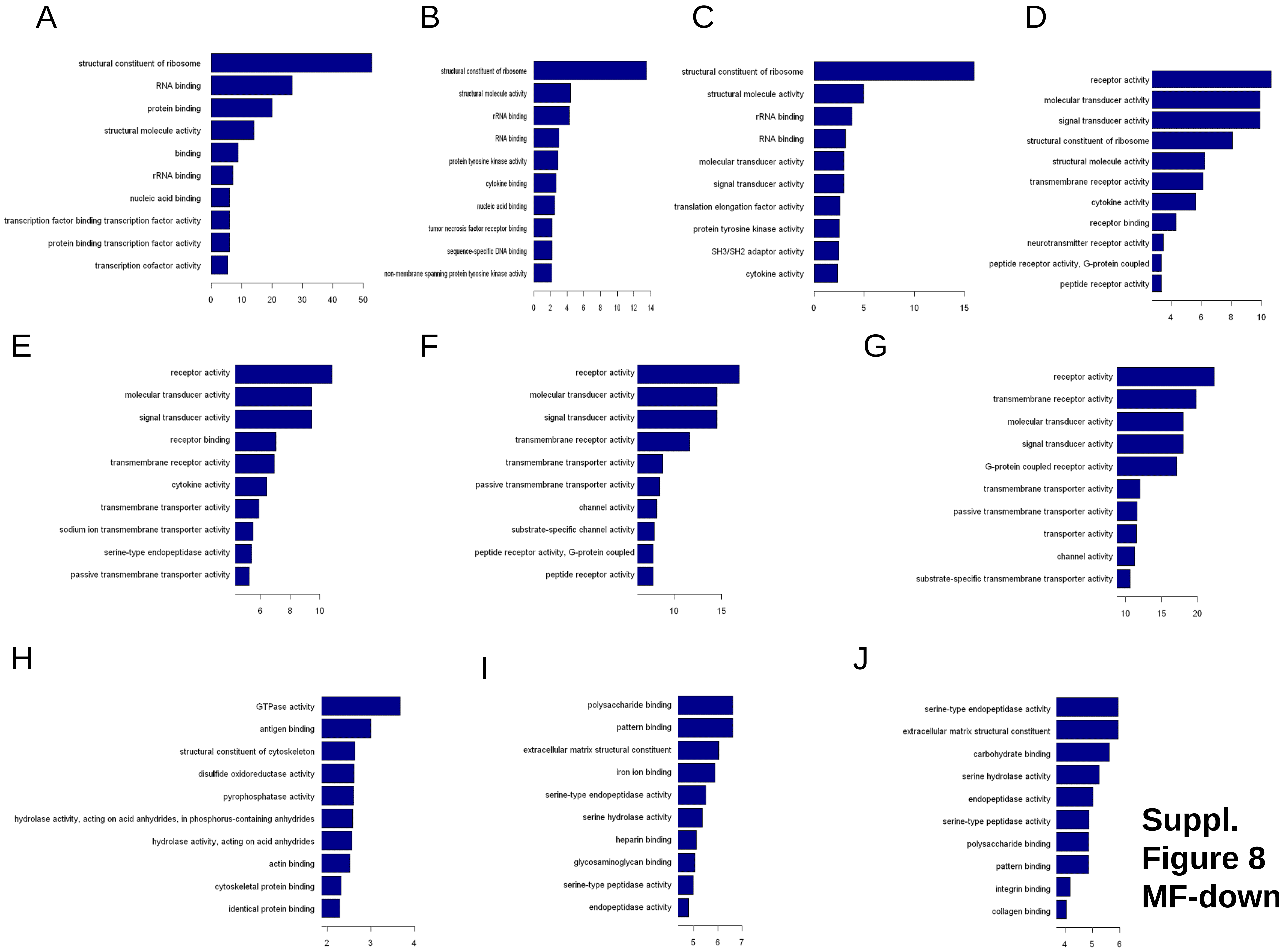


**Suppl. Figure 8 MF-down**
