# Supplementary material for: Dynamic gene expressions of peripheral blood mononuclear cells in patients with acute exacerbation of chronic obstructive pulmonary disease: a preliminary study
Source: Crit Care. 2014 Nov 19;18(6):508. doi: 10.1186/s13054-014-0508-y (PMC4305227; doi:10.1186/s13054-014-0508-y)
Supplement: Additional file 3: — Differentially expressed genes. This file lists 10 comparison pairs with information of fold changes and regulation, normalized intensities or annotations. [file 13054_2014_508_MOESM3_ESM.pdf]

Subject No.	1	2	3	4	5	6
Cough severeness	0	0	0	0	0	0
Sputum	0	0	0	0	0	0
Chest pain	0	0	0	0	0	0
Short breathness	0	0	0	0	0	0
Limitation of activity	0	0	0	0	0	0
Orthopnea at night	0	0	0	0	0	0
Edema of lower limbs	0	0	0	0	0	0
Chill	0	0	0	0	0	0
Fever(℃)	0	0	0	0	0	0
Duration of fever	0	0	0	0	0	0
Appetite	0	0	0	0	0	0
Hymoptysis	0	0	0	0	0	0
Stool and urine	0	0	0	0	0	0
Consciousness	0	0	0	0	0	0
Hypertension	0	0	0	0	0	0
Diabetes mellitus	0	0	0	0	0	0
Chronic obstructive pulmonary disease(COPD)	0	0	0	0	0	0
Temperature(℃)	0	0	0	0	0	0
Heart rate(beat/minute)	0	0	0	0	0	0
Respiratory rate(/minute)	0	0	0	0	0	0
Blood pressure(mmHg)	0	0	0	0	0	0
Nutrition	0	0	0	0	0	0
Enlargement of lymphnodes	0	0	0	0	0	0
Three depression sign	0	0	0	0	0	0
Barrel chest	0	0	0	0	0	0
Chest palpitation	0	0	0	0	0	0
Chest percussion	0	0	0	0	0	0
Rales	0	0	0	0	0	0
Heart examination	0	0	0	0	0	0
Abdominal examination	0	0	0	0	0	0
Hemoglobin(g/L)	0	0	0	0	0	0
WBC($\times 10^9$/L)	0	4	0	0	0	0
Neutrophil percentage(%)	0	0	4	0	0	0
Platelet($\times 10^9$/L)	0	0	0	0	0	0
Albumin(g/L)	0	0	0	0	0	0
ALT(U/L)	0	0	4	0	0	0
AST(U/L)	0	0	0	0	0	0
ALP(U/L)	0	0	0	0	0	0
Gamma-GT	0	0	0	4	0	0
Bilirubin(μmol/L)	0	0	0	0	0	0
Urea (mmol/L)	0	0	0	0	0	0
Creatinine(μmol/L)	0	0	0	0	0	0
Cholesterol(mmol/L)	0	0	0	0	0	0
Triglyceride(mmol/L)	0	0	0	0	0	0
HDL(mmol/L)	0	0	0	0	1	0

LDL(mmol/L)	0	0	0	0	2	0
Na(mmol/L)	0	0	0	0	0	0
K(mmol/L)	0	0	0	0	0	0
Cl(mmol/L)	0	0	0	0	0	0
Ca(mmol/L)	0	0	0	0	0	0
P(mmol/L)	0	0	0	0	0	0
Glycosylated hemoglobin,HbA1c(%)	0	0	0	0	0	0
pH	0	0	0	0	0	0
PaO2(mmHg)	0	0	0	0	0	0
PaCO2(mmHg)	0	0	0	0	0	0
SaO2(%)	0	0	0	0	0	0
Increased numbers of Tumor marker	0	0	0	0	0	0
C-reactive protein,CRP(mg/L)	0	0	0	0	0	0
Prothrombin time prolonged(sec)	0	0	0	0	0	0
Fasting blood glucose(mmol/L)	0	0	0	0	0	0
Lung consolidation	0	0	0	0	0	0
Enlargement of lymph nodes	0	0	0	0	0	0
Pleural effusion	0	0	0	0	0	0
Emphysema	0	0	0	0	0	0
**Total**	0	4	8	4	3	0

Stable COPD

Subject No.	1	2	3	4	5	6
Cough severeness	0	0	0	0	0	1
Sputum	1	2	1	2	2	0
Chest pain	0	0	0	0	0	0
Short breathness	1	0	1	2	0	2
Limitation of activity	1	0	0	4	0	2
Orthopnea at night	0	0	0	0	0	0
Edema of lower limbs	0	0	0	0	0	0
Chill	0	0	0	0	0	0
Fever(℃)	0	0	0	0	0	0
Duration of fever	0	0	0	0	0	0
Appetite	0	0	0	0	0	0
Hymoptysis	0	0	0	0	0	0
Stool and urine	0	0	0	0	0	0
Consciousness	0	0	0	0	0	0
Hypertension	1	0	1	4	4	2
Diabetes mellitus	0	0	0	0	0	0
Chronic obstructive pulmonary disease(COPD)	1	4	2	4	1	1
Temperature(℃)	0	0	0	0	0	0
Heart rate(beat/minute)	0	0	0	4	0	4
Respiratory rate(/minute)	0	0	0	0	0	0
Blood pressure(mmHg)	0	0	1	1	0	0
Nutrition	0	1	1	1	4	1
Enlargement of lymphnodes	0	0	0	0	0	0

Three depression sign	0	0	0	0	0	0
Barrel chest	4	0	4	4	0	4
Chest palpitation	0	0	0	0	0	0
Chest percussion	4	0	4	4	4	4
Rales	0	0	0	0	0	0
Heart examination	0	0	0	0	0	0
Abdominal examination	0	0	0	0	0	0
Hemoglobin(g/L)	0	0	0	0	0	0
WBC($\times 10^9$/L)	0	1	0	0	4	0
Neutrophil percentage(%)	0	4	4	0	0	4
Platelet($\times 10^9$/L)	0	0	0	0	0	0
Albumin(g/L)	0	2	0	2	0	2
ALT(U/L)	0	0	4	0	0	0
AST(U/L)	0	0	0	0	0	0
ALP(U/L)	0	0	0	0	0	0
Gamma-GT	0	0	0	0	4	0
Bilirubin(μmol/L)	0	0	0	0	0	0
Urea (mmol/L)	0	0	0	0	0	0
Creatinine(μmol/L)	0	0	0	0	0	0
Cholesterol(mmol/L)	1	0	1	1	1	0
Triglyceride(mmol/L)	1	0	0	0	0	1
HDL(mmol/L)	0	1	0	1	0	0
LDL(mmol/L)	0	1	0	0	1	0
Na(mmol/L)	1	0	0	1	0	0
K(mmol/L)	0	0	0	0	0	0
Cl(mmol/L)	0	0	0	0	0	0
Ca(mmol/L)	0	4	0	4	0	4
P(mmol/L)	4		0	4	4	4
Glycosylated hemoglobin,HbA1c(%)	0	0	0	0	0	0
pH	0	0	0	0	0	0
PaO2(mmHg)	1	1	2	2	1	1
PaCO2(mmHg)	0	1	1	4	4	4
SaO2(%)	0	0	0	1	0	1
Increased numbers of Tumor marker	0	0	0	0	0	0
C-reactive protein,CRP(mg/L)	1	1	0	1	0	1
Prothrombin time prolonged(sec)	0	0	0	0	0	0
Fasting blood glucose(mmol/L)	0	0	0	0	0	0
Lung consolidation	0	0	0	0	0	0
Enlargement of lymph nodes	4	0	4	0	0	0
Pleural effusion	0	0	0	0	0	0
Emphysema	4	4	4	4	4	4
**Total**	30	27	35	55	38	47

AECOPD-1

Subject No.	1	2	3	4	5	6
Cough severeness	1	2	2	1	4	2
Sputum	2	4	4	2	2	4

Chest pain	0	0	0	0	0	0
Short breathness	2	4	2	4	1	1
Limitation of activity	2	4	2	4	2	2
Orthopnea at night	4	4	0	4	4	0
Edema of lower limbs	4	0	4	0	0	4
Chill	0	4	4	0	4	0
Fever(℃)	4	1	0	2	1	4
Duration of fever	1	1	0	1	1	2
Appetite	1	0	1	1	0	1
Hymoptysis	0	0	0	0	0	0
Stool and urine	4	4	0	0	4	0
Consciousness	1	2	0	1	0	1
Hypertension	1	2	0	0	0	1
Diabetes mellitus	0	0	1	0	0	0
Chronic obstructive pulmonary disease(COPD)	4	4	2	1	1	2
Temperature(℃)	4	1	2	0	1	2
Heart rate(beat/minute)	0	4	0	0	4	0
Respiratory rate(/minute)	0	1	2	2	2	2
Blood pressure(mmHg)	1	2	0	0	0	2
Nutrition	1	1	1	0	4	4
Enlargement of lymphnodes	0	0	0	0	0	0
Three depression sign	0	4	4	4	4	4
Barrel chest	4	4	4	4	4	4
Chest palpitation	0	0	0	0	0	0
Chest percussion	4	4	4	4	4	4
Rales	1	1	2	2	4	4
Heart examination	0	0	0	0	0	0
Abdominal examination	0	0	0	0	0	0
Hemoglobin(g/L)	0	0	0	0	0	0
WBC($\times 10^9$/L)	4	4	1	1	4	4
Neutrophil percentage(%)	4	4	4	4	4	4
Platelet($\times 10^9$/L)	0	0	0	0	0	0
Albumin(g/L)	0	0	2	2	0	2
ALT(U/L)	0	0	0	4	0	0
AST(U/L)	0	0	4	0	0	0
ALP(U/L)	0	0	0	0	0	4
Gamma-GT	4	0	0	4	0	0
Bilirubin(μmol/L)	0	0	0	0	0	0
Urea (mmol/L)	0	0	0	0	0	0
Creatinine(μmol/L)	1	0	1	0	0	1
Cholesterol(mmol/L)	1	0	2	0	1	0
Triglyceride(mmol/L)	0	1	0	1	0	0
HDL(mmol/L)	1	1	0	1	0	1
LDL(mmol/L)	1	0	2	0	1	1
Na(mmol/L)	0	1	2	1	0	1
K(mmol/L)	1	0	1	1	0	0

Cl(mmol/L)	4	0	0	0	4	0
Ca(mmol/L)	4	4	0	4	0	4
P(mmol/L)	4	0	4	0	0	4
Glycosylated hemoglobin,HbA1c(%)	0	0	0	0	0	0
pH	4	0	4	0	0	4
PaO2(mmHg)	2	1	2	1	1	2
PaCO2(mmHg)	4	1	4	0	1	4
SaO2(%)	1	0	1	0	0	1
Increased numbers of Tumor marker	0	0	0	0	0	0
C-reactive protein,CRP(mg/L)	4	1	2	2	4	4
Prothrombin time prolonged(sec)	0	0	0	0	0	0
Fasting blood glucose(mmol/L)	0	0	0	0	0	0
Lung consolidation	1	0	1	2	0	2
Enlargement of lymph nodes	4	0	4	0	4	0
Pleural effusion	1	1	0	1	1	0
Emphysema	4	4	4	4	4	4
**Total**	100	81	86	70	80	97

AECOPD-3

Subject No.	1	2	3	4	5	6
Cough severeness	1	2	2	1	4	2
Sputum	2	2	4	2	2	2
Chest pain	0	0	0	0	0	0
Short breathness	0	2	2	2	1	1
Limitation of activity	2	4	2	4	2	2
Orthopnea at night	4	4	0	4	4	0
Edema of lower limbs	4	0	4	0	0	0
Chill	0	0	0	0	0	0
Fever(℃)	1	1	0	2	1	4
Duration of fever	1	1	0	1	1	2
Appetite	1	0	1	1	0	1
Hymoptysis	0	0	0	0	0	0
Stool and urine	0	4	0	0	4	0
Consciousness	1	1	0	1	0	1
Hypertension	1	2	0	0	0	1
Diabetes mellitus	0	0	1	0	0	0
Chronic obstructive pulmonary disease(COPD)	4	4	2	1	1	2
Temperature(℃)	2	1	1	0	1	2
Heart rate(beat/minute)	0	4	0	0	4	0
Respiratory rate(/minute)	0	1	2	2	2	2
Blood pressure(mmHg)	1	2	0	0	0	2
Nutrition	1	1	1	0	4	4
Enlargement of lymphnodes	0	0	0	0	0	0
Three depression sign	0	0	4	0	4	4
Barrel chest	4	4	4	4	4	4
Chest palpitation	0	0	0	0	0	0

Chest percussion	4	4	4	4	4	4
Rales	1	1	2	2	4	4
Heart examination	0	0	0	0	0	0
Abdominal examination	0	0	0	0	0	0
Hemoglobin(g/L)	0	0	0	0	0	0
WBC($\times 10^9$/L)	1	4	1	1	1	4
Neutrophil percentage(%)	4	4	4	0	4	4
Platelet($\times 10^9$/L)	0	0	0	0	0	0
Albumin(g/L)	0	0	2	2	0	2
ALT(U/L)	0	0	0	0	0	0
AST(U/L)	0	0	4	0	0	0
ALP(U/L)	0	0	0	0	0	4
Gamma-GT	4	0	0	4	0	0
Bilirubin(μmol/L)	0	0	0	0	0	0
Urea (mmol/L)	0	0	0	0	0	0
Creatinine(μmol/L)	1	0	1	0	0	1
Cholesterol(mmol/L)	1	0	2	0	1	0
Triglyceride(mmol/L)	0	1	0	1	0	0
HDL(mmol/L)	1	0	0	1	0	1
LDL(mmol/L)	1	0	0	1	1	1
Na(mmol/L)	1	0	1	1	0	1
K(mmol/L)	0	0	0	0	0	0
Cl(mmol/L)	4	0	0	0	4	0
Ca(mmol/L)	0	4	0	0	0	4
P(mmol/L)	4	0	4	0	0	0
Glycosylated hemoglobin,HbA1c(%)	0	0	0	0	0	0
pH	4	0	4	0	0	0
PaO2(mmHg)	1	1	2	1	1	2
PaCO2(mmHg)	2	1	4	0	1	2
SaO2(%)	1	0	1	0	0	1
Increased numbers of Tumor marker	0	0	0	0	0	0
C-reactive protein,CRP(mg/L)	4	1	1	1	2	4
Prothrombin time prolonged(sec)	0	0	0	0	0	0
Fasting blood glucose(mmol/L)	0	0	0	0	0	0
Lung consolidation	1	0	1	2	0	2
Enlargement of lymph nodes	4	0	4	0	4	0
Pleural effusion	0	1	0	1	1	0
Emphysema	4	4	4	4	4	4
**Total**	78	66	76	51	71	81

AECOPD-10

Subject No.	1	2	3	4	5	6
Cough severeness	1	0	0	0	1	1
Sputum	1	1	2	1	1	0
Chest pain	0	0	0	0	0	0
Short breathness	0	1	1	0	1	1
Limitation of activity	2	2	2	2	2	2

Orthopnea at night	0	4	0	4	0	0
Edema of lower limbs	0	0	0	0	0	0
Chill	0	0	0	0	0	0
Fever(℃)	0	0	0	0	0	0
Duration of fever	0	0	0	0	0	0
Appetite	1	0	1	1	0	1
Hymoptysis	0	0	0	0	0	0
Stool and urine	0	0	0	0	4	0
Consciousness	0	0	0	0	0	0
Hypertension	0	1	0	0	0	0
Diabetes mellitus	0	0	1	0	0	0
Chronic obstructive pulmonary disease(COPD)	4	4	2	1	1	2
Temperature(℃)	0	0	0	0	0	0
Heart rate(beat/minute)	0	4	0	0	0	0
Respiratory rate(/minute)	0	1	1	0	0	0
Blood pressure(mmHg)	0	1	0	0	0	1
Nutrition	1	1	1	0	4	4
Enlargement of lymphnodes	0	0	0	0	0	0
Three depression sign	0	0	0	0	0	0
Barrel chest	4	4	4	4	4	4
Chest palpitation	0	0	0	0	0	0
Chest percussion	4	4	4	4	4	4
Rales	0	1	1	1	0	0
Heart examination	0	0	0	0	0	0
Abdominal examination	0	0	0	0	0	0
Hemoglobin(g/L)	0	0	0	0	0	0
WBC($\times 10^9$/L)	0	1	1	0	1	0
Neutrophil percentage(%)	0	1	1	0	0	0
Platelet($\times 10^9$/L)	0	0	0	0	0	0
Albumin(g/L)	0	0	0	0	0	0
ALT(U/L)	0	0	0	0	0	0
AST(U/L)	0	4	0	0	0	0
ALP(U/L)	0	0	0	4	0	0
Gamma-GT	4	0	0	0	0	0
Bilirubin(μmol/L)	0	0	0	0	0	0
Urea (mmol/L)	0	0	0	0	0	0
Creatinine(μmol/L)	1	0	1	0	0	0
Cholesterol(mmol/L)	1	0	1	0	0	0
Triglyceride(mmol/L)	0	0	0	1	0	0
HDL(mmol/L)	1	0	0	1	0	0
LDL(mmol/L)	0	0	0	0	1	0
Na(mmol/L)	0	0	0	0	0	0
K(mmol/L)	0	0	0	0	0	0
Cl(mmol/L)	4	0	0	0	0	0
Ca(mmol/L)	0	4	0	0	0	4
P(mmol/L)	4	0	0	0	0	0

Glycosylated hemoglobin,HbA1c(%)	0	0	0	0	0	0
pH	0	0	0	0	0	0
PaO2(mmHg)	1	1	1	1	1	1
PaCO2(mmHg)	1	1	1	0	0	1
SaO2(%)	0	0	1	0	0	0
Increased numbers of Tumor marker	0	0	0	0	0	0
C-reactive protein,CRP(mg/L)	0	1	0	1	1	0
Prothrombin time prolonged(sec)	0	0	0	0	0	0
Fasting blood glucose(mmol/L)	0	0	0	0	0	0
Lung consolidation	0	0	1	0	0	0
Enlargement of lymph nodes	4	0	4	0	4	0
Pleural effusion	0	0	0		1	0
Emphysema	4	4	4	4	4	4
**Total**	43	46	36	30	35	30
