# Supplementary material for: Dynamic gene expressions of peripheral blood mononuclear cells in patients with acute exacerbation of chronic obstructive pulmonary disease: a preliminary study
Source: Crit Care. 2014 Nov 19;18(6):508. doi: 10.1186/s13054-014-0508-y (PMC4305227; doi:10.1186/s13054-014-0508-y)
Supplement: Additional file 4: — Co–differentially expressed genes. This file lists COPD-specific and AECOPD-specific genes, as well as dynamically changed genes, in patients with AECOPD. [file 13054_2014_508_MOESM4_ESM.pdf]

SEQ_ID	Fold chang	Log Fold cl	Absolute F	Regulation(	Fold chang	Log Fold cl	Absolute F	Regulation(	Fold chang	Log Fold cl	Absolute F	Regulation(	Fold chang	Log Fold cl	Absolute F	Regulation(
XM_93180	5.053987	2.337422	5.053987	up	8.482903	3.084558	8.482903	up	8.392629	3.0691228	8.392629	up	6.035794	2.5935435	6.035794	up
NM_00442	5.0682173	2.3414783	5.0682173	up	7.104262	2.8286848	7.104262	up	7.5342684	2.9134674	7.5342684	up	13.305146	3.7339125	13.305146	up
XM_92920	5.130059	2.3589754	5.130059	up	11.605559	3.536744	11.605559	up	9.255011	3.2102346	9.255011	up	22.138182	4.468465	22.138182	up
NM_00192	5.1794467	2.372798	5.1794467	up	83.86752	6.3900404	83.86752	up	72.62537	6.1824017	72.62537	up	7.6385717	2.9333029	7.6385717	up
BC015823	5.1999545	2.378499	5.1999545	up	19.592552	4.2922335	19.592552	up	25.329245	4.662732	25.329245	up	7.013664	2.8101683	7.013664	up
NM_13350	5.2028546	2.3793035	5.2028546	up	34.860336	5.1235147	34.860336	up	39.959152	5.320454	39.959152	up	47.89806	5.5818954	47.89806	up
NM_00100	5.2263603	2.3858066	5.2263603	up	22.848629	4.5140357	22.848629	up	16.299974	4.026798	16.299974	up	9.757754	3.286549	9.757754	up
NM_01693	5.302061	2.4065533	5.302061	up	8.708701	3.1224575	8.708701	up	10.48613	3.3904104	10.48613	up	10.172278	3.346571	10.172278	up
NM_00103	5.3622456	2.4228373	5.3622456	up	23.78455	4.571953	23.78455	up	18.137077	4.18087	18.137077	up	20.301058	4.343483	20.301058	up
XM_93461	5.49468	2.4580355	5.49468	up	5.7801957	2.5311184	5.7801957	up	6.847401	2.7755566	6.847401	up	5.1561046	2.3662815	5.1561046	up
NM_00514	5.5001073	2.4594598	5.5001073	up	30.251492	4.9189343	30.251492	up	49.993004	5.6436543	49.993004	up	27.046902	4.7573915	27.046902	up
AY327595	5.547538	2.4718475	5.547538	up	5.9605684	2.57545	5.9605684	up	5.924253	2.5666332	5.924253	up	8.356234	3.0628529	8.356234	up
BC104987	5.5692067	2.4774718	5.5692067	up	12.516432	3.6457515	12.516432	up	5.706553	2.5126195	5.706553	up	5.126108	2.357864	5.126108	up
BC098342	5.7547455	2.5247521	5.7547455	up	6.7692256	2.7589908	6.7692256	up	8.141781	3.0253444	8.141781	up	8.127568	3.0228238	8.127568	up
AY327584	5.844166	2.546997	5.844166	up	8.358311	3.0632114	8.358311	up	9.64278	3.2694492	9.64278	up	11.540425	3.5286245	11.540425	up
BC062415	5.871401	2.5537047	5.871401	up	36.20431	5.1780896	36.20431	up	41.61852	5.3791537	41.61852	up	149.45264	7.2235446	149.45264	up
AK129545	5.896833	2.5599403	5.896833	up	5.3019066	2.4065113	5.3019066	up	5.7366266	2.5202026	5.7366266	up	5.3355713	2.4156427	5.3355713	up
NM_00042	6.105528	2.610116	6.105528	up	18.124804	4.1798935	18.124804	up	23.328793	4.5440397	23.328793	up	24.221064	4.5981903	24.221064	up
BC106728	6.1531105	2.621316	6.1531105	up	42.204933	5.3993397	42.204933	up	24.680038	4.6252728	24.680038	up	6.357094	2.6683674	6.357094	up
AK130055	6.1605263	2.6230536	6.1605263	up	10.420685	3.3813782	10.420685	up	11.64934	3.5421762	11.64934	up	5.5469437	2.471693	5.5469437	up
NM_00727	6.277283	2.6501403	6.277283	up	15.415518	3.9463115	15.415518	up	17.160553	4.101024	17.160553	up	21.037193	4.3948703	21.037193	up
NM_02525	6.333117	2.6629157	6.333117	up	13.862817	3.7931485	13.862817	up	18.080725	4.1763806	18.080725	up	6.9813933	2.803515	6.9813933	up
AK024389	6.3402376	2.664537	6.3402376	up	7.5230184	2.9113116	7.5230184	up	9.958595	3.3159423	9.958595	up	6.2840796	2.6517015	6.2840796	up
NM_00185	6.4326296	2.6854086	6.4326296	up	17.679386	4.1439962	17.679386	up	18.853434	4.2367554	18.853434	up	10.988932	3.4579792	10.988932	up
NM_00202	6.581843	2.7184916	6.581843	up	56.182167	5.8120403	56.182167	up	41.786995	5.384982	41.786995	up	36.949802	5.2074947	36.949802	up
NM_00443	6.601213	2.722731	6.601213	up	32.598763	5.0267453	32.598763	up	38.844357	5.279633	38.844357	up	19.7369	4.3028235	19.7369	up
BX648333	6.6330895	2.729681	6.6330895	up	16.160217	4.0143747	16.160217	up	11.159	3.480136	11.159	up	8.494577	3.0865421	8.494577	up
AK123948	6.710685	2.74646	6.710685	up	10.401051	3.3786573	10.401051	up	12.157213	3.6037407	12.157213	up	27.100206	4.760232	27.100206	up
NM_00277	6.73734	2.7521791	6.73734	up	14.440702	3.852069	14.440702	up	12.587444	3.6539135	12.587444	up	10.137152	3.3415804	10.137152	up
NM_00554	6.7665033	2.7584105	6.7665033	up	13.764404	3.7828703	13.764404	up	19.43269	4.2804136	19.43269	up	10.73359	3.4240608	10.73359	up
NM_05297	6.873843	2.781117	6.873843	up	10.528294	3.3961997	10.528294	up	6.5842514	2.7190194	6.5842514	up	29.611563	4.8880887	29.611563	up
J05581	6.9140854	2.7895384	6.9140854	up	7.026109	2.812726	7.026109	up	10.215507	3.3526888	10.215507	up	13.006427	3.7011528	13.006427	up
X52022	6.941934	2.7953377	6.941934	up	31.376986	4.971635	31.376986	up	32.501713	5.022444	32.501713	up	13.722677	3.77849	13.722677	up
BC015822	7.213853	2.85077	7.213853	up	21.32415	4.4144163	21.32415	up	28.095875	4.8122864	28.095875	up	11.093256	3.471611	11.093256	up
NM_00394	7.380641	2.8837461	7.380641	up	29.264648	4.871087	29.264648	up	5.236775	2.3886786	5.236775	up	21.525925	4.4280033	21.525925	up
NM_00172	7.5334663	2.9133139	7.5334663	up	66.27352	6.0503607	66.27352	up	45.98308	5.523031	45.98308	up	5.4429016	2.444376	5.4429016	up
NM_00229	7.583889	2.9229379	7.583889	up	8.957251	3.163056	8.957251	up	10.556581	3.4000707	10.556581	up	22.685595	4.5037045	22.685595	up
BC009831	7.628482	2.931396	7.628482	up	9.878528	3.304296	9.878528	up	13.042626	3.7051625	13.042626	up	17.200348	4.104366	17.200348	up
BC072389	7.890725	2.9801579	7.890725	up	6.069083	2.6014786	6.069083	up	8.463799	3.0813055	8.463799	up	12.022131	3.5876207	12.022131	up
XM_92721	7.899749	2.9818068	7.899749	up	8.380928	3.06711	8.380928	up	24.931608	4.639904	24.931608	up	13.440564	3.7485218	13.440564	up
BC000006	8.032172	3.0057902	8.032172	up	37.349182	5.223005	37.349182	up	41.10578	5.3612695	41.10578	up	13.974061	3.8046794	13.974061	up
AY327589	8.36829	3.0649328	8.36829	up	7.849091	2.9725256	7.849091	up	7.966337	2.9939165	7.966337	up	11.69715	3.5480852	11.69715	up
NM_00103	8.438055	3.0769105	8.438055	up	9.189777	3.2000299	9.189777	up	8.964015	3.164145	8.964015	up	12.285069	3.618834	12.285069	up
BC093959	8.8662615	3.148326	8.8662615	up	140.3238	7.132616	140.3238	up	165.72356	7.372635	165.72356	up	10.393298	3.3775816	10.393298	up
NM_02478	8.9237995	3.157658	8.9237995	up	17.909302	4.162637	17.909302	up	23.991146	4.58443	23.991146	up	7.9532223	2.9915395	7.9532223	up
AK126468	8.939254	3.1601543	8.939254	up	6.8025656	2.766079	6.8025656	up	7.378802	2.8833866	7.378802	up	15.80658	3.9824533	15.80658	up
NM_00234	9.047701	3.1775513	9.047701	up	22.191559	4.471939	22.191559	up	27.770288	4.79547	27.770288	up	11.365212	3.5065527	11.365212	up
NM_00661	9.319993	3.2203288	9.319993	up	7.4601407	2.8992028	7.4601407	up	8.383851	3.0676131	8.383851	up	9.191339	3.200275	9.191339	up
BC111013	9.430238	3.2372942	9.430238	up	22.608175	4.4987726	22.608175	up	24.254116	4.6001577	24.254116	up	14.119249	3.8195915	14.119249	up
NM_00182	9.472523	3.2437487	9.472523	up	5.5869813	2.482069	5.5869813	up	5.0107565	2.3250284	5.0107565	up	16.107138	4.0096283	16.107138	up
BC032477	9.494841	3.2471437	9.494841	up	13.40431	3.744625	13.40431	up	16.394651	4.0351534	16.394651	up	10.460784	3.386919	10.460784	up
BC003179	9.588577	3.2613168	9.588577	up	14.059861	3.8135104	14.059861	up	14.225332	3.8303905	14.225332	up	19.373093	4.2759824	19.373093	up
BC061909	9.973563	3.318109	9.973563	up	6.46083	2.6917195	6.46083	up	6.7680173	2.7587333	6.7680173	up	8.461257	3.080872	8.461257	up
D12502	10.100639	3.3363748	10.100639	up	83.02904	6.375544	83.02904	up	66.52219	6.0557637	66.52219	up	10.53231	3.39675	10.53231	up
NM_01473	10.222143	3.3536258	10.222143	up	8.048899	3.0087914	8.048899	up	10.263717	3.3594813	10.263717	up	8.8265	3.1418414	8.8265	up
NM_00436	10.430713	3.3827658	10.430713	up	20.968586	4.3901577	20.968586	up	22.446365	4.48841	22.446365	up	10.764642	3.4282284	10.764642	up
NM_02416	10.962207	3.4544663	10.962207	up	6.5814013	2.7183948	6.5814013	up	6.2898936	2.6530356	6.2898936	up	7.636166	2.9328485	7.636166	up
NM_01672	11.840567	3.5656662	11.840567	up	6.290083	2.653079	6.290083	up	5.5327497	2.4679966	5.5327497	up	33.117973	5.0495424	33.117973	up
AF064599	12.095279	3.5963721	12.095279	up	13.629332	3.768643	13.629332	up	16.298649	4.0266805	16.298649	up	11.484472	3.5216126	11.484472	up
AY236492	12.150296	3.6029196	12.150296	up	11.283124	3.4960947	11.283124	up	12.281111	3.618369	12.281111	up	9.964038	3.3167305	9.964038	up
NM_00060	12.482279	3.6418095	12.482279	up	23.908117	4.5794287	23.908117	up	50.70422	5.664034	50.70422	up	9.645567	3.269866	9.645567	up
BC042586	13.140112	3.7159057	13.140112	up	72.34824	6.176886	72.34824	up	92.90301	6.5376534	92.90301	up	17.218151	4.1058583	17.218151	up
AK126554	14.053584	3.8128662	14.053584	up	7.0561166	2.8188744	7.0561166	up	7.434234	2.894184	7.434234	up	11.140398	3.4777288	11.140398	up
BC014473	14.69688	3.877438	14.69688	up	101.19586	6.6610065	101.19586	up	61.015594	5.931106	61.015594	up	11.79183	3.5597157	11.79183	up
NM_02244	16.54608	4.0484176	16.54608	up	6.608495	2.7243218	6.608495	up	5.629602	2.493033	5.629602	up	10.980861	3.4569192	10.980861	up
BC005019	16.667912	4.0590014	16.667912	up	5.4991765	2.4592156	5.4991765	up	5.1835275	2.3739343	5.1835275	up	31.398865	4.9726405	31.398865	up
AY358857	17.011192	4.0884123	17.011192	up	10.270072	3.3603745	10.270072	up	12.770851	3.6747828	12.770851	up	12.221561	3.6113567	12.221561	up
BC028178	18.9325	4.242793	18.9325	up	85.025314	6.4098206	85.025314	up	111.52946	6.801281	111.52946	up	9.489312	3.2463036	9.489312	up
AF348143	19.983717	4.320753	19.983717	up	19.734285	4.3026323	19.734285	up	20.077013	4.3274727	20.077013	up	28.643967	4.8401594	28.643967	up
NM_01238	20.070856	4.32703	20.070856	up	19.080643	4.254038	19.080643	up	21.261656	4.410182	21.261656	up	22.067963	4.4638815	22.067963	up
BC093946	20.154535	4.3330326	20.154535	up	45.88631	5.519992	45.88631	up	53.06496	5.7296877	53.06496	up	24.29677	4.6026926	24.29677	up
NM_00408	20.16457	4.3337507	20.16457	up	68.69194	6.102069	68.69194	up	62.7909	5.9724836	62.7909	up	9.111856	3.187745	9.111856	up
NM_00130	23.001772	4.523673	23.001772	up	34.41174	5.104829	34.41174	up	39.107784	5.289384	39.107784	up	20.699457	4.371521	20.699457	up
NM_00293	26.933065	4.7513065	26.933065	up	12.52757	3.6470346	12.52757	up	15.225223	3.9283915	15.225223	up	37.22396	5.2181597	37.22396	up
BC053636	29.465918	4.8809752	29.465918	up	37.195393	5.217052	37.195393	up	27.96823	4.805717	27.96823	up	11.834404	3.5649152	11.834404	up
BC069423	32.95388	5.0423765	32.95388	up	96.125435	6.5868464	96.125435	up	86.69046	6.4378014	86.69046	up	10.228047	3.3544588	10.228047	up
XM_92834	43.11271	5.4300413	43.11271	up	115.82023	6.8557434	115.82023	up	109.4489	6.7741137	109.4489	up	12.779329	3.6757402	12.779329	up
AK131567	43.54218	5.4443417	43.54218	up	8.036095	3.0064945	8.036095	up	7.952694	2.9914436	7.952694	up	24.606813	4.620986	24.606813	up
AY358483	50.668903	5.6630287	50.668903	up	9.014467	3.1722422	9.014467	up	12.293367	3.6198082	12.293367	up	58.154514	5.8618193	58.154514	up

SEQ_ID	[Con](norm	[Stable](no	[AE-1](norm	[AE-3](norm	[AE-10](no	GENE_NA	SYNONYM	DESCRIPT	NCBI_GEN	CHROMO	STRAND	START	STOP	GO biologi	GO cellula	GO molecu	UniGene	Dbid	TIGRID
XM_93180	189.31242	956.7825	1605.9188	1588.8289	1142.6508	DKFZp547I	MGC10500	hypothetical	283846	chr18	-	11609741	11632796	GO:000815	GO:000557	GO:000367	Hs.552700	gb:AL8341	THC247897
NM_00442	308.31064	1562.5853	2190.3196	2322.8953	4102.118	EFNA1	B61|ECKL	ephrin-A1	1942	chr1	+	153366972	153374010	GO:000726	GO:000588	GO:004687	Hs.516664	ref:NM_004	THC246787
XM_92920	274.7131	1409.2943	3188.199	2542.4727	6081.6484	LOC653723	-	similar to Si	653723	chr19	-	56838244	56844273						
NM_00192	315.6461	1634.8721	26472.457	22923.914	2411.0852	DEFA4	DEF4|HNP-	defensin, alp	1669	chr8	-	6780754	6783196	GO:000680	GO:000557	GO:000524	Hs.591391	ref:NM_001	THC247817
BC015823	1883.8507	9795.938	36909.445	47716.516	13212.695	LTF	GIG12|HLF	lactotransfe	4057	chr3	-	46452502	46481402	GO:000681	GO:000557	GO:000425	Hs.529517	ref:NM_002	THC248581
NM_13350	105.72747	550.08466	3685.6953	4224.7803	5064.1406	DCN	DSPG2|PG4	decorin	1634	chr12	-	90063165	90096460	GO:000988	GO:000557	GO:000551	Hs.156316	ref:NM_001	THC252247
NM_00100	158.76872	829.78253	3627.6477	2587.926	1549.2261	ATP1B1	ATP1B|MG	ATPase, Na	481	chr1	+	167342570	167368584	GO:000681	GO:000589	GO:000539	Hs.291196	ref:NM_001	THC253822
NM_01693	240.79668	1276.7186	2097.0261	2525.0251	2449.451	EFEMP2	FBLN4|UPI	EGF-contai	30008	chr11	-	65390494	65396789	GO:000759	GO:000560	GO:000488	Hs.647231	ref:NM_016	NP1153559
NM_00103	303.98657	1630.0505	7230.1836	5513.4277	6171.249	SCNN1A	ENaCa|ENa	sodium char	6337	chr12	-	6326273	6354976	GO:000681	GO:000562	GO:000521	Hs.591047	ref:NM_001	THC249189
XM_93461	434.13446	2385.43	2509.3823	2972.6929	2238.4426	RGL3	FLJ32585|M	ral guanine	57139	chr19	-	11365744	11390957	GO:000716	GO:000562	GO:000508	Hs.375142	ens:ENST0	THC251173
NM_00514	128.30162	705.67267	3881.3152	6414.1836	3470.1614	HP	MGC11114	haptoglobin	3240	chr16	+	70646008	70652458	GO:000650	GO:000557	GO:000425	Hs.513711	ref:NM_005	THC260533
AY327595	1269.9286	7044.9766	7569.496	7523.378	10611.82	MUC1	CD227|EMA	mucin 1, cel	4582	chr1	-	153425222	153429282		GO:000557	GO:000377	Hs.89603	ref:NM_002	NP078977
BC104987	101.6728	566.2368	1272.5807	580.2012	521.1857	B4GALT5	B4Gal-T5|B	UDP-Gal:be	9334	chr20	-	47685969	47763667	GO:000597	GO:001602	GO:000837	Hs.370487	ref:NM_004	THC246171
BC098342	209.52077	1205.7386	1418.2933	1705.8721	1702.8944	CSAD	CSD|MGC1	cysteine sul	51380	chr12	-	51838540	51859919	GO:0019752(carboxylic		GO:000478	Hs.279815	ref:NM_015	NP075459
AY327584	889.20685	5196.6724	7432.2676	8574.427	10261.826	MUC1	CD227|EMA	mucin 1, cel	4582	chr1	-	153425222	153429282		GO:000557	GO:000377	Hs.89603	ref:NM_002	NP078977
BC062415	202.74951	1190.4237	7340.4067	8438.135	30301.451	MFAP4	-	microfibrilla	4239	chr17	-	19227569	19230746	GO:000715	GO:000152	GO:000367	Hs.296049	ref:NM_002	NP1211437
AK129545	115.4866	681.0051	612.29913	662.5035	616.18695	N/A	N/A	Homo sapie	N/A	chr2	-	239934159	239935779						
NM_00042	127.26968	777.0486	2306.738	2969.048	3082.6072	LTBP2	C14orf141|I	latent transf	4053	chr14	-	74034638	74148787	GO:000007	GO:000557	GO:000550	Hs.512776	ref:NM_000	THC248511
BC106728	460.83588	2835.5742	19449.547	11373.447	2929.577	CEACAM3	CD66D|CEA	carcinoemb	1084	chr19	+	46992423	47007148		GO:0016020(membrane);GO:001602			gb:D90278;	THC248423
AK130055	110.94811	683.4987	1156.1553	1292.4722	615.42285	SPSB1	SSB-1|SSB1	splA/ryanod	80176	chr1	+	9350193	9352175	GO:0007242(intracellular signaling c			Hs.8261	ref:NM_025	THC246305
NM_00727	212.6405	1334.8047	3277.9636	3649.0286	4473.3594	FKBP9	DKFZp586I	FK506 bind	11328	chr7	+	32963576	33013067	GO:000645	GO:000578	GO:000375	Hs.103934	ref:NM_007	THC251319
NM_02525	167.92871	1063.5121	2327.9648	3036.2727	1172.3763	SLC44A4	C6orf29|CT	solute carrie	80736	chr6	-	31939174	31954736		GO:0016020(membrane		Hs.335355	ref:NM_025	THC249205
AK024389	131.5252	833.9011	989.46655	1309.8064	826.5149	FLJ14327	-	hypothetical	79972	chr16	+	80158915	80164449				Hs.656036	gb:AK0243	THC247883
NM_00185	403.43643	2595.157	7132.5083	7606.1616	4433.3354	COL8A1	MGC9568	collagen, ty	1295	chr3	+	100840143	100997848	GO:000681	GO:000557	GO:000519	Hs.654548	ref:NM_001	THC247207
NM_00202	127.4763	839.029	7161.895	5326.8516	4710.224	FN1	CIG|DKFZp	fibronectin 1	2335	chr2	-	215933421	216009036	GO:000695	GO:000557	GO:000520	Hs.203717	ref:NM_054	NP1276420
NM_00443	203.60304	1344.027	6637.207	7908.8286	4018.493	ELF3	EPR-1|ERT	E74-like fac	1999	chr1	+	200243695	200251756	GO:000635	GO:000563	GO:000370	Hs.67928	ref:NM_004	THC255623
BX648333	206.23967	1368.0062	3332.878	2301.4287	1751.9188	KIAA0703	ATP2C2|DK	KIAA0703	9914	chr16	+	82959633	83055293	GO:000681	GO:001602	GO:000016	Hs.6168	ref:NM_014	THC264990
AK123948	132.8199	891.3125	1381.4666	1614.72	3599.447	N/A	N/A	Homo sapie	N/A	chr21	-	41758354	41760885						
NM_00277	815.8948	5496.961	11782.093	10270.03	8270.849	PRSS8	CAP1|PROS	protease, se	5652	chr16	-	31050256	31054652	GO:000650	GO:000557	GO:000425	Hs.75799	ref:NM_002	THC261592
NM_00554	154.20822	1043.4504	2122.5845	2996.6804	1655.2079	ISLR	HsT17563|M	immunoglob	3671	chr15	+	72253139	72256265	GO:000715	GO:001602	GO:000551	Hs.513022	ref:NM_005	THC246995
NM_05297	176.50256	1213.251	1858.2708	1162.1372	5226.5166	HSPA12B	C20orf60|FI	heat shock 7	116835	chr20	+	3661355	3681758			GO:000016	Hs.516854	ref:NM_052	THC247235
J05581	132.32652	914.9168	929.74054	1351.7823	1721.0952	MUC1	CD227|EMA	mucin 1, cel	4582	chr1	-	153424929	153429330		GO:000557	GO:000377	Hs.89603	ref:NM_002	NP078977
X52022	162.05681	1124.9877	5084.8545	5267.1235	2223.8533	COL6A3	DKFZp686I	collagen, ty	1293	chr2	-	237897399	237987559	GO:000681	GO:000557	GO:000486	Hs.233240	ref:NM_057	THC256633
BC015822	1705.3613	12302.227	36365.383	47913.617	18918.012	LTF	GIG12|HLF	lactotransfe	4057	chr3	-	46452500	46481399	GO:000681	GO:000557	GO:000425	Hs.529517	ref:NM_002	THC248581
NM_00394	460.9547	3402.1414	13489.678	2413.916	9922.477	SELENBP1	FLJ13813|L	selenium bi	8991	chr1	-	149603403	149611788			GO:000843	Hs.632460	ref:NM_003	NP1180129
NM_00172	459.68216	3463.0002	30464.756	21137.604	2502.005	BPI	-	bactericidal/	671	chr20	+	36365998	36399319	GO:000695	GO:000588	GO:000828	Hs.529019	ref:NM_001	THC247054
NM_00229	246.25664	1867.5831	2205.7825	2599.6282	5586.4785	LAMB2	LAMS	laminin, bet	3913	chr3	-	49133550	49145603	GO:000715	GO:000560	GO:000519	Hs.439726	ref:NM_002	THC248931
BC009831	179.07822	1366.095	1769.029	2335.6504	3080.2075	RAB25	CATX-8	RAB25, me	57111	chr1	+	154297589	154306917	GO:000726	GO:001602	GO:000016	Hs.632469	ref:NM_020	THC266804
BC072389	108.13709	853.2801	656.293	915.25073	1300.0383	GJA4	CX37	gap junction	2701	chr1	+	35031195	35033933	GO:000681	GO:000588	GO:001528	Hs.296310	ref:NM_002	THC247072
XM_92721	107.24469	847.2061	898.81006	2673.7825	1441.4292	AATK	AATYK|KI	apoptosis-as	9625	chr17	-	76705165	76709894	GO:000646	GO:001602	GO:000016	Hs.514575	ref:NM_001	THC267089
BC000006	179.68878	1443.2913	6711.2295	7386.248	2510.982	ATP1B1	ATP1B|MG	ATPase, Na	481	chr1	+	167342605	167367623	GO:000681	GO:000589	GO:000539	Hs.291196	ref:NM_001	THC253822
AY327589	947.09686	7925.581	7433.8496	7544.893	11078.335	MUC1	CD227|EMA	mucin 1, cel	4582	chr1	-	153425222	153429282		GO:000557	GO:000377	Hs.89603	ref:NM_002	NP078977
NM_00103	225.93361	1906.4402	2076.2795	2025.2721	2775.6099	ALOX15B	-	arachidonat	247	chr17	+	7883082	7893167	GO:000611	GO:000562	GO:000550	Hs.111256	ref:NM_001	NP704909
BC093959	102.31657	907.1655	14357.452	16956.268	1063.4066	DEFA4	DEF4|HNP-	defensin, alp	1669	chr8	-	6780910	6783194	GO:000680	GO:000557	GO:000524	Hs.591391	ref:NM_001	THC247817
NM_02478	116.40898	1038.8104	2084.8037	2792.785	925.8265	TMC5	FLJ13593	transmembr	79838	chr16	+	19375272	19417579		GO:0016021(integral to		Hs.115838	ref:NM_024	NP1276461
AK126468	102.03749	912.139	694.1167	752.9144	1612.8638	ADCY4	-	adenylate cy	196883	chr14	-	23857394	23874117	GO:000617	GO:000562	GO:000028	Hs.443428	ref:NM_139	THC249944
NM_00234	1671.651	15124.599	37096.54	46422.23	18998.668	LTF	GIG12|HLF	lactotransfe	4057	chr3	-	46452499	46481399	GO:000681	GO:000557	GO:000425	Hs.529517	ref:NM_002	THC248581
NM_00661	232.11095	2163.2722	1731.5802	1945.9836	2133.4102	NES	FLJ21841|N	nestin	10763	chr1	-	154905178	154913813	GO:000741	GO:000588	GO:000519	Hs.527971	ref:NM_006	NP286942
BC111013	120.51132	1136.4504	2724.541	2922.8958	1701.5294	ISLR	HsT17563|M	immunoglob	3671	chr15	+	72253229	72256265	GO:000715	GO:001602	GO:000551	Hs.513022	ref:NM_005	THC246995
NM_00182	594.75244	5633.806	3322.8708	2980.1597	9579.76	CKB	B-CK|CKBI	creatine kin	1152	chr14	-	103055748	103058923		GO:000573	GO:000016	Hs.173724	ref:NM_001	THC258659
BC032477	354.02902	3361.4492	4745.515	5804.1826	3703.4211	TMC4	MGC39329	transmembr	147798	chr19	-	59355658	59360262		GO:0016021(integral to		Hs.355126	ref:NM_144	THC246463
BC003179	211.70166	2029.9177	2976.4958	3011.5264	4101.316	MALL	BENE|MGC	mal, T-cell	7851	chr2	-	110199036	110230728	GO:004263	GO:000013	GO:000551	Hs.185055	ref:NM_005	THC278655
BC061909	177.27792	1768.0927	1145.3625	1199.8201	1499.9941	N/A	N/A	Homo sapie	N/A	chr2	-	220134688	220138284						
D12502	216.29684	2184.7366	17958.92	14388.54	2278.1055	CEACAM1	BGP|BGP1|	carcinoemb	634	chr19	-	47705094	47724458	GO:000152	GO:000557	GO:000367	Hs.512682	ref:NM_001	THC248014
NM_01473	201.1827	2056.5183	1619.2991	2064.882	1775.7389	ProSAPiP1	KIAA0552	ProSAPiP1	9762	chr20	-	3091272	3097207		GO:000573	GO:000519	Hs.90232	ref:NM_014	THC246475
NM_00436	774.40466	8077.5923	16238.171	17382.57	8336.188	COL6A3	DKFZp686I	collagen, ty	1293	chr2	-	237897400	237987559	GO:000681	GO:000557	GO:000486	Hs.233240	ref:NM_057	THC256633
NM_02416	178.65079	1958.407	1175.7726	1123.6945	1364.2072	TPSB2	TPS2|TPSB	tryptase bet	64499	chr16	-	1218336	1220186	GO:000650	GO:000557	GO:000425	Hs.592982	ref:NM_024	THC246296
NM_01672	467.1344	5531.136	2938.3142	2584.5376	15470.544	FOLR1	FBP|FOLR|	folate recep	2348	chr11	+	71580820	71584989	GO:000689	GO:000562	GO:000487	Hs.73769	ref:NM_016	NP226704
AF064599	205.8457	2489.7612	2805.5393	3355.0068	2364.0293	NOL3	ARC|MYC|	nucleolar pr	8996	chr16	+	65765405	65767139	GO:000639	GO:000562	GO:000372	Hs.513667	ref:NM_003	THC246332
AY236492	432.08313	5249.938	4875.2476	5306.4604	4305.2925	TMC4	MGC39329	transmembr	147798	chr19	-	59355714	59368756		GO:0016021(integral to		Hs.355126	ref:NM_144	THC246463
NM_00060	105.47439	1316.5608	2521.694	5347.9966	1017.3602	ORM2	AGP-B|AGI	orosomucoi	5005	chr9	+	116131889	116135357	GO:000695	GO:000561	GO:000548	Hs.522356	ref:NM_000	THC256281
BC042586	109.15891	1434.3604	7897.4556	10141.191	1879.5146	COL1A2	OI4	collagen, ty	1278	chr7	+	93862161	93897951	GO:000150	GO:000558	GO:000520	Hs.489142	ref:NM_000	NP1166796
AK126554	207.04286	2909.6943	1460.9186	1539.2051	2306.5398	ST5	DENND2B|	suppression	6764	chr11	-	8671475	8849493			GO:000551	Hs.117715	ref:NM_005	THC246800
BC014473	177.27792	2605.4326	17939.793	10816.718	2090.4312	CEACAM1	BGP|BGP1|	carcinoemb	634	chr19	-	47705097	47724458	GO:000152	GO:000557	GO:000367	Hs.512682	ref:NM_001	THC248014
NM_02244	147.55833	2441.512	975.13855	830.69464	1620.3174	RAB17	FLJ12538	RAB17, me	64284	chr2	-	238147703	238177653	GO:000726	GO:000562	GO:000016	Hs.44278	ref:NM_022	THC261454
BC005019	152.21384	2537.0867	837.0508	789.00464	4779.342	S100A14	BCMP84|S1	S100 calciu	57402	chr1	-	151853911	151855038			GO:000550	Hs.288998	ref:NM_020	THC246163
AY358857	267.5786	4551.8306	2748.0515	3417.2065	3270.2283	MLPH	FLJ12145|M	melanophili	79083	chr2	+	238091410	238127372	GO:000660	GO:001562	GO:000377	Hs.102406	ref:NM_024	THC258978
BC028178	133.502	2527.5269	11351.05	14889.406	1266.8422	COL3A1	EDS4A	collagen, ty	1281	chr2	+	189547356	189585024	GO:000681	GO:000558	GO:000520	Hs.443625	ref:NM_000	THC257712
AF348143	333.22064	6658.987	6575.8706	6690.0747	9544.761	MUC1	CD227|EMA	mucin 1, cel	4582	chr1	-	153425222	153429282		GO:000557	GO:000377	Hs.89603	ref:NM_002	NP078977
NM_01238	255.34796	5125.052	4872.203	5429.12	5635.0093	P8	COM1	p8 protein (	26471	chr16	-	28456162	28457831	GO:000691	GO:000563	GO:000367	Hs.513463	ref:NM_012	THC255444
BC093946	116.12872	2340.5203	5328.7183	6162.366	2821.5527	UNQ473	MGC13830	DMC	284340	chr19	-	47624852	47638891				Hs.445586	ref:NM_198	THC247074
NM_00408	730.6505	14733.253	50189.8	45878.203	6657.583	DEFA1	DEF1|DEFA	defensin, alp	1667	chr8	-	6822580	6825012	GO:000680	GO:0005576(extracellul		Hs.380781	gb:BX4270	THC271059
NM_00130	332.62906	7651.0576	11446.344	13008.386	6885.2407	CLDN4	CPE-R|CPE	claudin 4	1364	chr7	+	72883128	72884951	GO:000940	GO:000588	GO:000488	Hs.647036	ref:NM_001	THC249388
NM_00293	566.8566	15267.187	7101.336	8630.519	21100.648	RNASE1	MGC12408	ribonuclease	6035	chr14	-	20339354	20340876		GO:000557	GO:000372	Hs.78224	ref:NM_198	THC259471
BC053636	216.63985	6383.4917	8058.004	6059.0327	2563.8035	H19	ASM|ASM1	H19, imprin	283120	chr11	-	1972983	1975280				Hs.533566	ref:NR_002	THC252433
BC069423	512.40594	16885.766	49255.246	44420.71	5240.912	DEFA1	DEF1|DEFA	defensin, alp	1667	chr8	-	6822651	6824982	GO:000680	GO:0005576(extracellul		Hs.380781	gb:BX4270	THC271059
XM_92834	420.37894	18123.676	48688.383	46010.01	5372.161	LOC653600	-	similar to N	653600	chr8	-	6841686	6844110						
AK131567	439.6755	19144.43	3533.274	3496.6047	10819.014	N/A	N/A	Homo sapie	N/A	chr19	-	20595976	20636239						
AY358483	116.86469	5921.406	1053.4729	1436.6605	6796.209	MGC45438	-	hypothetical	146556	chr16	-	5034124	5056112				Hs.11782	ref:NM_152	NP814308

COPD Vs Control Down

SEQ_ID	Fold chang	Log Fold cl	Absolute F	Regulation(	Fold chang	Log Fold cl	Absolute F	Regulation(	Fold chang	Log Fold cl	Absolute F	Regulation(	Fold chang	Log Fold cl	Absolute F	Regulation(
M38056	-5.250969	-2.392584	5.2509694	down	-5.87648	-2.554952	5.8764796	down	-5.612449	-2.48863	5.6124487	down	-7.311253	-2.870119	7.3112526	down
AY209188	-5.315821	-2.410293	5.315821	down	-6.728399	-2.750263	6.728399	down	-6.442515	-2.687624	6.442515	down	-11.93453	-3.577069	11.934526	down
BC069511	-5.456451	-2.447963	5.4564505	down	-10.37424	-3.374933	10.374236	down	-14.2999	-3.837933	14.299899	down	-6.926629	-2.792153	6.9266286	down
AJ421515	-5.580614	-2.480424	5.580614	down	-25.3616	-4.664574	25.361597	down	-12.46958	-3.640341	12.469579	down	-11.90723	-3.573766	11.907228	down
AL133666	-5.631684	-2.493567	5.6316843	down	-5.838156	-2.545513	5.8381557	down	-8.181806	-3.032419	8.181806	down	-5.324263	-2.412582	5.324263	down
NM_02015	-5.6994	-2.51081	5.6993995	down	-8.170423	-3.030411	8.170423	down	-9.666873	-3.273049	9.666873	down	-10.42983	-3.382644	10.42983	down
XM_08938	-5.773323	-2.529402	5.7733226	down	-11.65871	-3.543336	11.658708	down	-12.45145	-3.638242	12.451453	down	-12.6978	-3.666507	12.697801	down
NM_00685	-5.998098	-2.584505	5.998098	down	-6.469234	-2.693595	6.469234	down	-10.7175	-3.421897	10.7175	down	-11.06183	-3.467518	11.061828	down

SEQ_ID	[Con](norm	[Stable](no	[AE-1](norm	[AE-3](norm	[AE-10](no	GENE_NA	SYNONYM	DESCRIPT	NCBI_GEN	CHROMO	STRAND	START	STOP	GO biologi	GO cellula	GO molecu	UniGene	Dbid	TIGRID
M38056	1094.3848	208.41576	186.23135	194.9924	149.68498	HLA-DOA	HLA-DNA|	major histoc	3111	chr6	-	33084016	33085337	GO:000695	GO:0005886(plasma me		Hs.631991	ref:NM_002	THC247308
AY209188	1713.7035	322.37793	254.69708	265.99915	143.5921	SAA3P	SAA3	serum amyl	6290	chr11	-	18090594	18094255	GO:000695	GO:000557	GO:000367	Hs.454496	gb:AY2091	NP110391
BC069511	2437.1147	446.64838	234.9199	170.4288	351.84717	UBASH3A	TULA	ubiquitin as	53347	chr21	+	42697094	42740422		GO:0005634(nucleus)		Hs.473912	ref:NM_018	THC247542
AJ421515	5339.434	956.7825	210.53224	428.1968	448.41953	CRTAC1	ASPIC1|CE	cartilage aci	55118	chr10	-	99629401	99761084			GO:000550	Hs.500736	ref:NM_018	THC250302
AL133666	911.46936	161.84666	156.12282	111.40199	171.19164	EPHA6	DKFZp434	EPH recept	285220	chr3	+	98641179	98850111	GO:000646	GO:001602	GO:000016	Hs.653244	gb:AL1336	THC261246
NM_02015	4151.0933	728.33875	508.06345	429.41428	398.002	C21orf7	TAK1L	chromosom	56911	chr21	+	29374743	29470075			GO:000551	Hs.222802	ref:NM_020	THC261828
XM_08938	2206.2725	382.1495	189.23816	177.18994	173.75232	TTC24	-	tetratricope	164118	chr1	+	154817780	154823520						
NM_00685	1866.94	311.25534	288.5875	174.19548	168.7732	IL24	C49A|FISP|	interleukin 2	11009	chr1	+	205137411	205144107	GO:000691	GO:000557	GO:000512	Hs.658964	ref:NM_006	THC247324

AL713701	-6.119443	-2.613401	6.1194434	down	-9.489696	-3.246362	9.489696	down	-9.541381	-3.254198	9.541381	down	-10.00003	-3.321932	10.000026	down	4347.295	710.40686	458.1069	455.62534	434.72836	C21orf7	TAK1L	chromosom	56911	chr21	+	29424868	29470064			GO:000551	Hs.222802	ref:NM_020	THC261828
XM_93159	-6.186644	-2.629157	6.186644	down	-11.41454	-3.512801	11.414543	down	-5.744108	-2.522083	5.7441077	down	-7.402883	-2.888087	7.402883	down	1313.4487	212.30391	115.06802	228.66019	177.42395	LOC643514	-	hypothetical	643514	chr8	-	100022766	100023747						
NM_00615	-6.276377	-2.649932	6.2763767	down	-11.5046	-3.524139	11.504601	down	-7.347457	-2.877245	7.3474565	down	-10.00502	-3.322652	10.005021	down	2227.4153	354.8887	193.61082	303.15457	222.62973	NELL2	NRP2	NEL-like 2	4753	chr12	-	43188331	43556405	GO:000715	GO:000557	GO:000519	Hs.505326	ref:NM_006	THC251221
NM_00234	-6.692009	-2.742439	6.6920085	down	-8.152088	-3.02717	8.152088	down	-7.358062	-2.879326	7.3580623	down	-6.52728	-2.706482	6.52728	down	1991.0325	297.5239	244.23587	270.59195	305.03247	LY9	CD229|SLA	lymphocyte	4063	chr1	+	159032551	159064669	GO:000715	GO:001602	GO:000367	Hs.403857	ref:NM_002	THC260375
XM_93485	-6.890036	-2.784512	6.890036	down	-14.54817	-3.862766	14.54817	down	-12.88771	-3.687923	12.887705	down	-9.41295	-3.234647	9.41295	down	2026.3582	294.0998	139.28612	157.23189	215.27347	LOC129293	FLJ46467	hypothetical	129293	chr2	-	84911269	84961721						
BC062589	-6.941211	-2.795188	6.941211	down	-7.265337	-2.86103	7.2653365	down	-7.124588	-2.832807	7.124588	down	-5.465092	-2.450246	5.465092	down	1664.9404	239.8631	229.16219	233.68936	304.65002	LY9	CD229|SLA	lymphocyte	4063	chr1	+	159050936	159064667	GO:000715	GO:001602	GO:000367	Hs.403857	ref:NM_002	THC260375
XM_93414	-7.021789	-2.811839	7.021789	down	-7.540285	-2.914619	7.5402846	down	-11.22667	-3.488859	11.226674	down	-6.198253	-2.631862	6.198253	down	1131.8488	161.19093	150.1069	100.81781	182.6077	KIAA0748	HSPC257	KIAA0748	9840	chr12	-	53629449	53653930						
BC008567	-7.292638	-2.866441	7.292638	down	-6.276696	-2.650005	6.276696	down	-7.512347	-2.909264	7.5123467	down	-7.71396	-2.947472	7.7139597	down	4777.2285	655.0755	761.1056	635.91693	619.2965	C21orf7	TAK1L	chromosom	56911	chr21	+	29424676	29469749			GO:000551	Hs.222802	ref:NM_020	THC261828
NM_13836	-7.786442	-2.960964	7.786442	down	-6.35867	-2.668725	6.3586698	down	-5.867333	-2.552705	5.867333	down	-5.233062	-2.387655	5.233062	down	3097.3176	397.78345	487.1015	527.89197	591.8749	CCDC45	-	coiled-coil c	90799	chr17	+	59933619	59964526			GO:000551	Hs.569713	ref:NM_138	NP1402281
BC022101	-7.842644	-2.97134	7.842644	down	-44.14891	-5.464306	44.14891	down	-18.08497	-4.17672	18.084974	down	-32.28505	-5.012795	32.285053	down	7126.6973	908.711	161.42409	394.0673	220.74294	UNQ470	-	GAAI470	375704	chr9	-	34511041	34513027				Hs.522085	ref:NM_198	THC261452
BC027920	-7.863331	-2.975141	7.863331	down	-6.237559	-2.640982	6.2375593	down	-5.838262	-2.545539	5.8382616	down	-5.345279	-2.418265	5.345279	down	2220.777	282.42194	356.03302	380.38327	415.46515	LY9	CD229|SLA	lymphocyte	4063	chr1	+	159032651	159064667	GO:000715	GO:001602	GO:000367	Hs.403857	ref:NM_002	THC260375
BC033896	-8.212065	-3.037745	8.212065	down	-8.166988	-3.029804	8.166988	down	-10.57849	-3.403062	10.578494	down	-9.861098	-3.301748	9.861098	down	1317.6743	160.4559	161.3415	124.56162	133.62349	AK5	AK6|MGC3	adenylate ki	26289	chr1	+	77520874	77797007	GO:000613	GO:000573	GO:000016	Hs.559718	ref:NM_174	THC248432
XM_08515	-10.74784	-3.425974	10.747836	down	-8.720418	-3.124397	8.720418	down	-5.104356	-2.351729	5.104356	down	-7.382225	-2.884056	7.3822246	down	1156.867	107.6372	132.66188	226.64308	156.70981	YLPM1	C14orf170|Z	YLP motif c	56252	chr14	+	74299827	74372853	GO:000815	GO:000557	GO:000367	Hs.531111	gb:BC0235	THC275442
NM_01455	-16.08003	-4.007198	16.080032	down	-21.7658	-4.443991	21.7658	down	-32.03665	-5.001651	32.036648	down	-14.88979	-3.896252	14.889792	down	4558.0938	283.46298	209.41539	142.27748	306.12207	TFCP2L1	CRTR1|LBI	transcriptior	29842	chr2	-	121695003	121759245	GO:000012	GO:000563	GO:000370	Hs.156471	ref:NM_014	THC261509
NM_00100	-21.03073	-4.394427	21.030733	down	-27.92748	-4.803614	27.927483	down	-18.68745	-4.223998	18.687447	down	-18.59982	-4.217217	18.599817	down	4235.381	201.39006	151.65637	226.64308	227.71089	SCP2	DKFZp686	sterol carrie	6342	chr1	+	53165535	53232492	GO:000669	GO:000573	GO:000549	Hs.476365	ref:NM_002	THC269380

	(AE-1 vs Con) ∩(AE-1 vs Stable) up								(AE-3 vs Con) ∩(AE-3 vs Stable) up								(AE-10 vs Con) ∩(AE-10 vs Stable) up								Normalized Intensities										
**SEQ_ID**	**Fold chang**	**Log Fold c**	**Absolute F**	**Regulatior**	**Fold chang**	**Log Fold c**	**Absolute F**	**Regulatior**	**Fold chang**	**Log Fold c**	**Absolute F**	**Regulatior**	**Fold chang**	**Log Fold c**	**Absolute F**	**Regulatior**	**Fold chang**	**Log Fold c**	**Absolute F**	**Regulatior**	**Fold chang**	**Log Fold c**	**Absolute F**	**Regulatior**	**[Con PBM**	**[Stable PB**	**[AE-1 PBN**	**[AE-3 PBN**	**[AE-10 8P**	**GENE_NA**	**SYNONYI**	**DESCRIP**	**NCBI_GE**	**CHROMC**	**START**
AB191261	12.0543	3.591476	12.0543	up	6.931292	2.793124	6.931292	up	11.74042	3.553412	11.74042	up	6.750808	2.75506	6.750808	up	10.1716	3.346475	10.1716	up	5.84873	2.548123	5.84873	up	253.8776	441.5218	3060.316	2980.629	2582.342	FN1	CIG|DKFZ	fibronectin	2335	chr2	2.16E+08
AB209814	12.64951	3.66101	12.64951	up	12.97689	3.697873	12.97689	up	20.04907	4.325463	20.04907	up	20.56796	4.362327	20.56796	up	9.831717	3.297443	9.831717	up	10.08617	3.334307	10.08617	up	550.0894	536.2117	6958.362	11028.78	5408.323	ALPL	AP-TNAP|	alkaline ph	249	chr1	21708465
AX772826	6.304344	2.656346	6.304344	up	11.91554	3.574772	11.91554	up	6.757782	2.75655	6.757782	up	12.77256	3.674976	12.77256	up	6.725339	2.749607	6.725339	up	12.71124	3.668033	12.71124	up	226.2718	119.7172	1426.495	1529.095	1521.754	N/A	N/A	Sequence 2	N/A	chr17	31334809
BC004490	27.43201	4.777788	27.43201	up	28.56771	4.836313	28.56771	up	33.53661	5.067665	33.53661	up	34.92505	5.12619	34.92505	up	13.19787	3.722233	13.19787	up	13.74427	3.780758	13.74427	up	709.8009	681.583	19471.26	23804.32	9367.857	FOS	c-fos	v-fos FBJ r	2353	chr14	74815312
BC005008	19.12746	4.257573	19.12746	up	14.66587	3.874391	14.66587	up	12.47856	3.641379	12.47856	up	9.567863	3.258197	9.567863	up	8.446976	3.078435	8.446976	up	6.476671	2.695252	6.476671	up	480.1248	626.1863	9183.566	5991.265	4055.603	CEACAM	CD66c|CE	carcinoeml	4680	chr19	46951346
BC005322	8.366265	3.064584	8.366265	up	21.15465	4.402903	21.15465	up	9.203319	3.202154	9.203319	up	23.2712	4.540474	23.2712	up	10.46855	3.387989	10.46855	up	26.47041	4.726308	26.47041	up	495.6312	196.0128	4146.582	4561.452	5188.537	DCN	DSPG2|PG	decorin	1634	chr12	90063644
BC007628	29.69084	4.891946	29.69084	up	8.770476	3.132655	8.770476	up	34.44355	5.106162	34.44355	up	10.1744	3.346871	10.1744	up	36.13453	5.175306	36.13453	up	10.6739	3.416016	10.6739	up	115.3032	390.3379	3423.449	3971.452	4166.428	KRT19	CK19|K19|	keratin 19	3880	chr17	36933395
BC007695	7.101755	2.828176	7.101755	up	5.740242	2.521112	5.740242	up	9.336472	3.222878	9.336472	up	7.54653	2.915813	7.54653	up	6.785271	2.762406	6.785271	up	5.484432	2.455342	5.484432	up	320.0501	395.962	2272.918	2988.139	2171.627	KLF5	BTEB2|CK	Kruppel-lil	688	chr13	72531154
BC009016	11.60929	3.537208	11.60929	up	7.141369	2.836201	7.141369	up	11.11364	3.474259	11.11364	up	6.836474	2.773253	6.836474	up	9.629347	3.267438	9.629347	up	5.923423	2.566431	5.923423	up	1846.264	3001.359	21433.81	20518.71	17778.32	C1QC	C1QG|C1q	complemer	714	chr1	22842739
BC015492	12.27898	3.618118	12.27898	up	10.29262	3.363538	10.29262	up	13.11475	3.713119	13.11475	up	10.99319	3.458538	10.99319	up	21.56913	4.430896	21.56913	up	18.07991	4.176315	18.07991	up	594.574	709.3201	7300.759	7797.69	12824.44	IFI27	ISG12|P27	interferon,	3429	chr14	93646913
BC030153	16.37814	4.0337	16.37814	up	6.941948	2.795341	6.941948	up	14.19687	3.827501	14.19687	up	6.017407	2.589142	6.017407	up	12.68699	3.665278	12.68699	up	5.377434	2.426918	5.377434	up	544.175	1283.873	8912.576	7725.584	6903.94	C1QA	-	complemer	712	chr1	22835734
BC050368	6.833447	2.772614	6.833447	up	9.455256	3.241117	9.455256	up	6.876368	2.781647	6.876368	up	9.514645	3.25015	9.514645	up	10.84008	3.438304	10.84008	up	14.99913	3.906807	14.99913	up	370.8137	267.9923	2533.936	2549.851	4019.651	MMP19	MMP18|RA	matrix met	4327	chr12	54516478
BC058919	5.687138	2.507703	5.687138	up	9.140419	3.19226	9.140419	up	6.368471	2.670947	6.368471	up	10.23546	3.355505	10.23546	up	31.00942	4.954635	31.00942	up	49.83862	5.639192	49.83862	up	216.2119	134.5263	1229.627	1376.939	6704.605	SEPP1	SELP|SeP	selenoprote	6414	chr5	42835989
BC062415	36.20431	5.17809	36.20431	up	6.166214	2.624385	6.166214	up	41.61852	5.379154	41.61852	up	7.088346	2.825449	7.088346	up	149.4526	7.223545	149.4526	up	25.45434	4.66984	25.45434	up	202.7495	1190.424	7340.407	8438.135	30301.45	MFAP4	-	microfibril	4239	chr17	19227569
BC066954	7.67408	2.939994	7.67408	up	5.816628	2.540183	5.816628	up	9.621324	3.266235	9.621324	up	7.292556	2.866425	7.292556	up	17.81222	4.154796	17.81222	up	13.50091	3.754985	13.50091	up	446.1574	588.6309	3423.847	4292.624	7947.054	RHOB	ARH6|ARI	ras homolo	388	chr2	20510315
BC107587	19.90376	4.314969	19.90376	up	7.56441	2.919228	7.56441	up	35.20491	5.137705	35.20491	up	13.3796	3.741963	13.3796	up	16.07473	4.006723	16.07473	up	6.10919	2.610981	6.10919	up	190.8107	502.0683	3797.85	6717.474	3067.23	HP	MGC1111	haptoglobi	3240	chr16	70646022
BX538018	14.82445	3.889907	14.82445	up	7.284761	2.864882	7.284761	up	19.66299	4.297411	19.66299	up	9.662427	3.272386	9.662427	up	16.0318	4.002865	16.0318	up	7.878055	2.97784	7.878055	up	165.2368	336.256	2449.545	3249.05	2649.044	FN1	CIG|DKFZ	fibronectin	2335	chr2	2.16E+08
CR600380	7.84934	2.972571	7.84934	up	5.112182	2.353939	5.112182	up	7.928878	2.987117	7.928878	up	5.163984	2.368485	5.163984	up	9.908327	3.308641	9.908327	up	6.453175	2.690009	6.453175	up	408.8553	627.7642	3209.244	3241.764	4051.072	SERPING1	C1IN|C1IN	serpin pept	710	chr11	57122255
M18728	28.96551	4.856264	28.96551	up	21.00156	4.392425	21.00156	up	26.19068	4.710981	26.19068	up	18.98966	4.247142	18.98966	up	10.1878	3.34877	10.1878	up	7.386703	2.884931	7.386703	up	167.2268	230.6404	4843.809	4379.782	1703.672	CEACAM	CD66c|CE	carcinoeml	4680	chr19	46951336
NM_0000	9.570893	3.258654	9.570893	up	5.321119	2.41173	5.321119	up	10.40499	3.379204	10.40499	up	5.784852	2.53228	5.784852	up	18.23309	4.188487	18.23309	up	10.13703	3.341563	10.13703	up	588.1798	1057.936	5629.406	6120.005	10724.33	SERPING1	C1IN|C1IN	serpin pept	710	chr11	57121602
NM_0000	11.74595	3.554092	11.74595	up	9.082691	3.18312	9.082691	up	13.97888	3.805177	13.97888	up	10.80933	3.434205	10.80933	up	12.00422	3.585469	12.00422	up	9.282398	3.214498	9.282398	up	271.3211	350.8789	3186.924	3792.765	3256.997	C3	ASP|CPAN	complemer	718	chr19	6628877
NM_0003	28.02783	4.808788	28.02783	up	9.049896	3.177901	9.049896	up	32.85424	5.038008	32.85424	up	10.60829	3.407121	10.60829	up	88.91787	6.474402	88.91787	up	28.71066	4.843514	28.71066	up	176.2925	545.9838	4941.097	5791.957	15675.55	TIMP3	HSMRK22	TIMP meta	7078	chr22	31526801
NM_0004	17.32271	4.114593	17.32271	up	6.38264	2.674153	6.38264	up	25.41245	4.667463	25.41245	up	9.363345	3.227024	9.363345	up	13.87254	3.79416	13.87254	up	5.111408	2.353721	5.111408	up	703.1303	1908.32	12180.12	17868.26	9754.202	ALPL	AP-TNAP|	alkaline ph	249	chr1	21708455
NM_0004	13.40302	3.744486	13.40302	up	9.118811	3.188846	9.118811	up	12.02482	3.587943	12.02482	up	8.181146	3.032303	8.181146	up	9.017483	3.172725	9.017483	up	6.13509	2.617085	6.13509	up	674.2067	990.9629	9036.403	8107.212	6079.647	C1QB	-	complemer	713	chr1	22858473
NM_0010	8.576448	3.10038	8.576448	up	6.381206	2.673829	6.381206	up	10.44151	3.384258	10.44151	up	7.768879	2.957707	7.768879	up	6.787014	2.762777	6.787014	up	5.049798	2.336226	5.049798	up	134.6304	180.9455	1154.651	1405.744	913.7382	ATF3	-	activating t	467	chr1	2.11E+08
NM_0015	12.0101	3.586176	12.0101	up	40.32281	5.333524	40.32281	up	11.2091	3.486598	11.2091	up	37.63352	5.233946	37.63352	up	11.68515	3.546604	11.68515	up	39.23182	5.293953	39.23182	up	497.7946	148.2675	5978.562	5579.828	5816.804	CYR61	CCN1|GIG	cysteine-ric	3491	chr1	85819047
NM_0017	6.552609	2.71207	6.552609	up	5.934189	2.569051	5.934189	up	14.9246	3.89962	14.9246	up	13.51605	3.756601	13.51605	up	6.095377	2.607716	6.095377	up	5.52011	2.464697	5.52011	up	158.8355	175.3883	1040.787	2370.556	968.1626	BCL6	BCL5|BCL	B-cell CLL	604	chr3	1.89E+08
NM_0017	19.70589	4.300555	19.70589	up	16.23278	4.020838	16.23278	up	18.77741	4.230927	18.77741	up	15.46794	3.951209	15.46794	up	6.949381	2.796885	6.949381	up	5.724569	2.517167	5.724569	up	260.6184	316.3795	5135.718	4893.739	1811.137	C1R	-	complemer	715	chr12	7057771
NM_0017	9.425613	3.236587	9.425613	up	9.088198	3.183994	9.088198	up	15.52155	3.956201	15.52155	up	14.96591	3.903608	14.96591	up	33.36822	5.060403	33.36822	up	32.17371	5.007811	32.17371	up	106.2856	110.2316	1001.807	1649.717	3546.561	CDH5	7B4|CD14	cadherin 5,	1003	chr16	64958063
NM_0018	15.00384	3.90726	15.00384	up	5.241824	2.390069	5.241824	up	17.74687	4.149493	17.74687	up	6.200146	2.632302	6.200146	up	50.88115	5.669059	50.88115	up	17.77612	4.151869	17.77612	up	105.9795	303.3484	1590.099	1880.805	5392.359	CNN3	-	calponin 3,	1266	chr1	95135094
NM_0019	35.67524	5.156851	35.67524	up	26.60939	4.733863	26.60939	up	36.88405	5.204925	36.88405	up	27.51101	4.781937	27.51101	up	59.26115	5.889015	59.26115	up	44.2016	5.466027	44.2016	up	103.7402	139.0846	3700.957	3826.358	6147.764	CTGF	CCN2|IGF	connective	1490	chr6	1.32E+08
NM_0019	12.74516	3.671878	12.74516	up	7.871085	2.976563	7.871085	up	14.36053	3.844037	14.36053	up	8.868694	3.148722	8.868694	up	17.31207	4.113707	17.31207	up	10.69149	3.418391	10.69149	up	344.1622	557.2806	4386.403	4942.351	5958.161	DCN	DSPG2|PG	decorin	1634	chr12	90063165
NM_0019	13.25828	3.728822	13.25828	up	12.06289	3.592504	12.06289	up	25.56226	4.675943	25.56226	up	23.25752	4.539625	23.25752	up	5.937971	2.56997	5.937971	up	5.402593	2.433652	5.402593	up	478.353	525.7562	6342.138	12227.78	2840.446	ACSL1	ACS1|FAC	acyl-CoA s	2180	chr4	1.86E+08
NM_0020	56.18217	5.81204	56.18217	up	8.535933	3.093549	8.535933	up	41.787	5.384982	41.787	up	6.348829	2.666491	6.348829	up	36.9498	5.207495	36.9498	up	5.613899	2.489003	5.613899	up	127.4763	839.029	7161.895	5326.852	4710.224	FN1	CIG|DKFZ	fibronectin	2335	chr2	2.16E+08
NM_0022	13.93254	3.800386	13.93254	up	7.365065	2.880698	7.365065	up	15.88699	3.989774	15.88699	up	8.398237	3.070087	8.398237	up	17.93596	4.164783	17.93596	up	9.481368	3.245095	9.481368	up	167.9287	317.6717	2339.673	2667.882	3011.962	KRT19	CK19|K19|	keratin 19	3880	chr17	36933394
NM_0023	6.215687	2.635914	6.215687	up	8.231704	3.041191	8.231704	up	6.546782	2.710786	6.546782	up	8.670187	3.116063	8.670187	up	7.453154	2.897851	7.453154	up	9.870535	3.303128	9.870535	up	799.6087	603.7775	4970.117	5234.863	5959.606	TACSTD2	EGP-1|GA	tumor-asso	4070	chr1	58813230
NM_0024	8.668697	3.115815	8.668697	up	5.451674	2.446699	5.451674	up	9.675421	3.274324	9.675421	up	6.084794	2.605208	6.084794	up	29.83276	4.898826	29.83276	up	18.76158	4.22971	18.76158	up	677.4774	1077.256	5872.846	6554.878	20211.02	MFAP4	-	microfibril	4239	chr17	19227347
NM_0027	6.880107	2.782431	6.880107	up	6.260376	2.646249	6.260376	up	6.451015	2.689526	6.451015	up	5.869934	2.553344	5.869934	up	7.37838	2.883304	7.37838	up	6.713766	2.747122	6.713766	up	123.4909	135.7156	849.6306	796.6415	911.1627	HTRA1	HtrA|L56|(	HtrA serin	5654	chr10	1.24E+08
NM_0039	21.1966	4.405761	21.1966	up	12.6222	3.657891	12.6222	up	19.15176	4.259405	19.15176	up	11.40453	3.511536	11.40453	up	75.80648	6.244249	75.80648	up	45.14142	5.49638	45.14142	up	190.8107	320.4305	4044.538	3654.361	14464.69	GPRC5A	GPCR5A|F	G protein-c	9052	chr12	12935222
NM_0040	6.98992	2.805276	6.98992	up	9.073383	3.181641	9.073383	up	8.840452	3.14412	8.840452	up	11.4755	3.520485	11.4755	up	13.07961	3.709248	13.07961	up	16.97821	4.085612	16.97821	up	593.9545	457.5685	4151.694	5250.827	7768.693	RHOB	ARH6|ARI	ras homolo	388	chr2	20510315
NM_0041	5.530504	2.467411	5.530504	up	5.800321	2.536133	5.800321	up	6.652995	2.734004	6.652995	up	6.977575	2.802726	6.977575	up	7.05802	2.819264	7.05802	up	7.40236	2.887985	7.40236	up	390.2045	372.0531	2158.028	2596.029	2754.071	FKBP5	FKBP51|FI	FK506 bin	2289	chr6	35649344
NM_0044	9.980993	3.319183	9.980993	up	18.15914	4.182624	18.15914	up	9.488791	3.246224	9.488791	up	17.26364	4.109665	17.26364	up	5.623712	2.491523	5.623712	up	10.23163	3.354963	10.23163	up	1337.125	734.9375	13345.83	12687.7	7519.604	DUSP1	CL100|HV	dual specif	1843	chr5	1.72E+08
NM_0046	10.57725	3.402893	10.57725	up	6.315615	2.658923	6.315615	up	8.725389	3.125219	8.725389	up	5.209878	2.381249	5.209878	up	18.55993	4.214119	18.55993	up	11.08202	3.470149	11.08202	up	829.1235	1388.598	8769.849	7234.424	15388.47	TGM2	TG2|TGC	transglutan	7052	chr20	36190276
NM_0051	19.69508	4.299764	19.69508	up	5.324744	2.412712	5.324744	up	27.94098	4.804311	27.94098	up	7.554097	2.917259	7.554097	up	21.56461	4.430594	21.56461	up	5.830186	2.543542	5.830186	up	107.8663	398.9743	2124.436	3013.89	2326.094	ANXA3	ANX3	annexin A3	306	chr4	79694613
NM_0052	22.25604	4.476125	22.25604	up	18.6635	4.222148	18.6635	up	30.14927	4.914051	30.14927	up	25.28262	4.660074	25.28262	up	10.90464	3.446871	10.90464	up	9.144435	3.192894	9.144435	up	357.4784	426.2893	7956.053	10777.71	3898.175	FOS	c-fos	v-fos FBJ r	2353	chr14	74815283
NM_0061	7.321864	2.872211	7.321864	up	6.593782	2.721106	6.593782	up	8.138095	3.024691	8.138095	up	7.328847	2.873586	7.328847	up	8.727266	3.12553	8.727266	up	7.859431	2.974425	7.859431	up	107.0682	118.8906	783.9388	871.3312	934.4127	NNMT	-	nicotinami	4837	chr11	1.14E+08
NM_0064	17.42309	4.122929	17.42309	up	9.464724	3.24256	9.464724	up	18.40173	4.20177	18.40173	up	9.996351	3.321402	9.996351	up	20.67753	4.369992	20.67753	up	11.23263	3.489624	11.23263	up	719.1608	1323.864	12530	13233.81	14870.47	FBLN1	FBLN	fibulin 1	2192	chr22	44277382
NM_0067	21.67992	4.438287	21.67992	up	13.68584	3.774612	13.68584	up	40.12742	5.326517	40.12742	up	25.33117	4.662842	25.33117	up	35.61987	5.15461	35.61987	up	22.48569	4.490935	22.48569	up	166.3222	263.4731	3605.851	6674.08	5924.374	FOSB	G0S3|GOS	FBJ murin	2354	chr19	50663092
NM_0072	5.516048	2.463635	5.516048	up	5.875654	2.55475	5.875654	up	5.954978	2.574096	5.954978	up	6.3432	2.665211	6.3432	up	5.263865	2.396123	5.263865	up	5.607031	2.487237	5.607031	up	119.8374	112.503	661.0288	713.6291	630.8079	SEC63	ERdj2|PRC	SEC63-like	11231	chr6	1.08E+08
NM_0072	21.39357	4.419106	21.39357	up	55.98866	5.807063	55.98866	up	10.86633	3.441793	10.86633	up	28.43803	4.82975	28.43803	up	5.732241	2.519099	5.732241	up	15.00172	3.907056	15.00172	up	319.8407	122.2129	6842.536	3475.494	1833.404	VSIG4	Z39IG	V-set and i	11326	chrX	65158304
NM_1335	19.03901	4.250886	19.03901	up	17.22858	4.106732	17.22858	up	19.62389	4.294539	19.62389	up	17.75784	4.150384	17.75784	up	19.96777	4.319602	19.96777	up	18.06903	4.175447	18.06903	up	208.8525	230.7993	3976.344	4098.497	4170.318	DCN	DSPG2|PG	decorin	1634	chr12	90063165
NM_1335	28.8113	4.848563	28.8113	up	6.110167	2.611212	6.110167	up	38.895	5.281513	38.895	up	8.248672	3.044162	8.248672	up	51.46201	5.685436	51.46201	up	10.91382	3.448085	10.91382	up	105.799	498.8745	3048.206	4115.052	5444.628	DCN	DSPG2|PG	decorin	1634	chr12	90063165
NM_1335	34.86034	5.123515	34.86034	up	6.700233	2.744211	6.700233	up	39.95915	5.320454	39.95915	up	7.680236	2.941151	7.680236	up	47.89806	5.581895	47.89806	up	9.206111	3.202592	9.206111	up	105.7275	550.0847	3685.695	4224.78	5064.141	DCN	DSPG2|PG	decorin	1634	chr12	90063165
NM_1723	23.83608	4.575075	23.83608	up	11.45819	3.518307	11.45819	up	18.87592	4.238475	18.87592	up	9.073797	3.181706	9.073797	up	21.83383	4.448493	21.83383	up	10.49569	3.391725	10.49569	up	777.9804	1618.407	18544	14685.09	16986.29	C1QC	C1QG|C1q	complemer	714	chr1	22842733
U81380	8.305531	3.054072	8.305531	up	12.30203	3.620825	12.30203	up	6.982822	2.80381	6.982822	up	10.34285	3.370563	10.34285	up	5.205516	2.380041	5.205516	up	7.710335	2.946794	7.710335	up	190.3768	128.53	1581.18	1329.367	991.0094	IL13RA1	CD213A1|	interleukin	3597	chrX	1.18E+08
XM_3707	6.651386	2.733655	6.651386	up	7.220107	2.85202	7.220107	up	7.287939	2.865511	7.287939	up	7.911089	2.983876	7.911089	up	5.187938	2.375161	5.187938	up	5.631528	2.493527	5.631528	up	291.0483	268.1227	1935.875	2121.143	1509.941	LOC38793	-	similar to F	387934	chr13	72572488
XM_3734	41.37525	5.370696	41.37525	up	13.54279	3.759453	13.54279	up	46.42712	5.536896	46.42712	up	15.19635	3.925653	15.19635	up	41.33596	5.369326	41.33596	up	13.52993	3.758082	13.52993	up	159.9905	488.7949	6619.646	7427.896	6613.36	LOC38776	-	hypothetica	387763	chr11	43903467
XM_9292	10.22543	3.35409	10.22543	up	13.78548	3.785077	13.78548	up	24.62779	4.622215	24.62779	up	33.2021	5.053203	33.2021	up	6.114021	2.612122	6.114021	up	8.242654	3.043109	8.242654	up	197.3606	146.393	2018.097	4860.555	1206.667	LOC64630	-	similar to N	646309	chr10	36850652

(AE-1 vs Con) ∩(AE-1 vs Stable) up (AE-3 vs Con) ∩(AE-3 vs Stable) up (AE-10 vs Con) ∩(AE-10 vs Stable) up Normalized Intensities

SEQ_ID Fold chang Log Fold c Absolute F Regulatior Fold chang Log Fold c Absolute F Regulatior Fold chang Log Fold c Absolute F Regulatior Fold chang Log Fold c Absolute F Regulatior Fold chang Log Fold c Absolute F Regulatior Fold chang Log Fold c Absolute F Regulatior [Con PBM [Stable PB [AE-1 PBN [AE-3 PBN [AE-10 8P GENE_NA SYNONYI DESCRIP NCBI_GE CHROMC START

BC004490	27.43201	4.777788	27.43201	up	28.56771	4.836313	28.56771	up	33.53661	5.067665	33.53661	up	34.92505	5.12619	34.92505	up	13.19787	3.722233	13.19787	up	13.74427	3.780758	13.74427	up	709.8009	681.583	19471.26	23804.32	9367.857	FOS	c-fos	v-fos FBJ r	2353	chr14	74815312
BC015492	12.27898	3.618118	12.27898	up	10.29262	3.363538	10.29262	up	13.11475	3.713119	13.11475	up	10.99319	3.458538	10.99319	up	21.56913	4.430896	21.56913	up	18.07991	4.176315	18.07991	up	594.574	709.3201	7300.759	7797.69	12824.44	IFI27	ISG12|P27	interferon,	3429	chr14	93646913
NM_00155	12.0101	3.586176	12.0101	up	40.32281	5.333524	40.32281	up	11.2091	3.486598	11.2091	up	37.63352	5.233946	37.63352	up	11.68515	3.546604	11.68515	up	39.23182	5.293953	39.23182	up	497.7946	148.2675	5978.562	5579.828	5816.804	CYR61	CCN1|GIG	cysteine-ric	3491	chr1	85819047
NM_00190	35.67524	5.156851	35.67524	up	26.60939	4.733863	26.60939	up	36.88405	5.204925	36.88405	up	27.51101	4.781937	27.51101	up	59.26115	5.889015	59.26115	up	44.2016	5.466027	44.2016	up	103.7402	139.0846	3700.957	3826.358	6147.764	CTGF	CCN2|IGF	connective	1490	chr6	1.32E+08
NM_00397	21.1966	4.405761	21.1966	up	12.6222	3.657891	12.6222	up	19.15176	4.259405	19.15176	up	11.40453	3.511536	11.40453	up	75.80648	6.244249	75.80648	up	45.14142	5.49638	45.14142	up	190.8107	320.4305	4044.538	3654.361	14464.69	GPRC5A	GPCR5A|F	G protein-c	9052	chr12	12935222
NM_00673	21.67992	4.438287	21.67992	up	13.68584	3.774612	13.68584	up	40.12742	5.326517	40.12742	up	25.33117	4.662842	25.33117	up	35.61987	5.15461	35.61987	up	22.48569	4.490935	22.48569	up	166.3222	263.4731	3605.851	6674.08	5924.374	FOSB	G0S3|GOS	FBJ murine	2354	chr19	50663092
NM_13350	19.03901	4.250886	19.03901	up	17.22858	4.106732	17.22858	up	19.62389	4.294539	19.62389	up	17.75784	4.150384	17.75784	up	19.96777	4.319602	19.96777	up	18.06903	4.175447	18.06903	up	208.8525	230.7993	3976.344	4098.497	4170.318	DCN	DSPG2|PG	decorin	1634	chr12	90063165
XM_37349	41.37525	5.370696	41.37525	up	13.54279	3.759453	13.54279	up	46.42712	5.536896	46.42712	up	15.19635	3.925653	15.19635	up	41.33596	5.369326	41.33596	up	13.52993	3.758082	13.52993	up	159.9905	488.7949	6619.646	7427.896	6613.36	LOC38776	-	hypothetica	387763	chr11	43903467

	(AE-1 vs Con) ∩(AE-1 vs Stable) down								(AE-3 vs Con) ∩(AE-3 vs Stable) down								(AE-10 vs Con) ∩(AE-10 vs Stable) down								Normalized Intensities										
SEQ_ID	Fold chang	Log Fold c	Absolute F	Regulation	Fold chang	Log Fold c	Absolute F	Regulation	Fold chang	Log Fold c	Absolute F	Regulation	Fold chang	Log Fold c	Absolute F	Regulation	Fold chang	Log Fold c	Absolute F	Regulation	Fold chang	Log Fold c	Absolute F	Regulation	[Con PBM	[Stable PB	[AE-1 PBM	[AE-3 PBM	[AE-10 PB	GENE_NA	SYNONY	DESCRIP	NCBI_GE	CHROMO	START
AB042648	-2.27021	-1.18283	2.270213	down	-3.40858	-1.76917	3.408584	down	-2.13113	-1.09162	2.131131	down	-3.19976	-1.67796	3.199761	down	-2.08251	-1.05832	2.082505	down	-3.12675	-1.64466	3.126752	down	611.453	918.0587	269.3372	286.9147	293.6142	MYO18B	DKFZp434	myosin XV	84700	chr22	24495993
AB073385	-4.30153	-2.10485	4.30153	down	-2.41748	-1.2735	2.417481	down	-3.67128	-1.87628	3.671276	down	-2.06328	-1.04494	2.063275	down	-3.64245	-1.86491	3.642451	down	-2.04708	-1.03356	2.047075	down	515.1675	289.5266	119.7638	140.3238	141.4343	C14orf68	HDMCP|H	chromoson	283600	chr14	99859431
AF002256	-2.12843	-1.08979	2.128425	down	-2.07533	-1.05334	2.075332	down	-2.60304	-1.3802	2.603044	down	-2.53811	-1.34375	2.53811	down	-2.90006	-1.53608	2.900059	down	-2.82772	-1.49964	2.827717	down	1617.851	1577.494	760.1166	621.523	557.8685	KIR2DL4	103AS|15.2	killer cell i	3805	chr19	60006922
AF074087	-3.0983	-1.63147	3.098295	down	-2.48749	-1.31469	2.487485	down	-4.79671	-2.26205	4.796712	down	-3.85107	-1.94526	3.851069	down	-2.64845	-1.40515	2.648447	down	-2.12632	-1.08836	2.126322	down	1678.65	1347.715	541.7982	349.9586	633.8245	KIR2DS2	183ACTI|(	killer cell i	3807	chr19_rand	274165
AF290986	-2.26339	-1.17848	2.263388	down	-2.09308	-1.06563	2.09308	down	-5.05448	-2.33756	5.05448	down	-4.67416	-2.22471	4.674155	down	-3.32307	-1.73252	3.323071	down	-3.07303	-1.61966	3.073027	down	2071.184	1915.338	915.0813	409.772	623.2741	SLA2	C20orf156	Src-like-ad	84174	chr20	34675682
AF294278	-3.45213	-1.78749	3.452131	down	-6.52236	-2.70539	6.522363	down	-2.85623	-1.51411	2.856232	down	-5.39649	-2.43202	5.396487	down	-2.09048	-1.06384	2.090481	down	-3.9497	-1.98174	3.949699	down	560.9077	1059.764	162.4816	196.3803	268.3151	PRDM16	KIAA1675	PR domain	63976	chr1	2975620
AF320053	-2.28872	-1.19454	2.288724	down	-2.14865	-1.10343	2.148653	down	-2.64802	-1.40491	2.648016	down	-2.48596	-1.3138	2.485956	down	-3.18266	-1.67023	3.182663	down	-2.98788	-1.57912	2.987882	down	428.5703	402.3416	187.253	161.8458	134.6578	MYCN	MODED|N	v-myc mye	4613	chr2	15998136
AF367022	-3.72848	-1.89859	3.728477	down	-2.44498	-1.28982	2.444979	down	-3.11334	-1.63846	3.113336	down	-2.0416	-1.0297	2.041596	down	-3.06304	-1.61496	3.063039	down	-2.00861	-1.0062	2.008613	down	378.9629	248.4844	101.6305	121.7109	123.7095	KCNIP3	CSEN|DRI	Kv channel	30818	chr2	95326913
AF465821	-2.31799	-1.21287	2.317986	down	-3.33105	-1.73598	3.331046	down	-2.35377	-1.23497	2.353766	down	-3.38246	-1.75807	3.382464	down	-2.21042	-1.14432	2.210419	down	-3.17647	-1.66742	3.176467	down	1668.677	2397.961	719.8823	708.939	754.9144	FAM71B	MGC2698	family with	153745	chr5	1.57E+08
AJ002102	-3.71957	-1.89513	3.719565	down	-3.82965	-1.93721	3.829654	down	-7.40683	-2.88886	7.406831	down	-7.62605	-2.93094	7.626052	down	-4.23646	-2.08286	4.236462	down	-4.36185	-2.12494	4.361849	down	1355.273	1395.385	364.3632	182.9761	319.9067	KIR2DS2	183ACTI|(	killer cell i	3807	chr19_rand	164628
AJ002104	-2.31627	-1.2118	2.316267	down	-2.22194	-1.15182	2.221941	down	-3.72694	-1.89799	3.72694	down	-3.57517	-1.83801	3.575166	down	-2.67726	-1.42076	2.677264	down	-2.56824	-1.36078	2.568236	down	831.2791	797.4266	358.8875	223.046	310.4958	KIR2DS2	183ACTI|(	killer cell i	3807	chr19_rand	164628
AJ421515	-25.3616	-4.66457	25.3616	down	-4.54459	-2.18415	4.544589	down	-12.4696	-3.64034	12.46958	down	-2.23445	-1.15992	2.234446	down	-11.9072	-3.57377	11.90723	down	-2.13368	-1.09334	2.133677	down	5339.434	956.7825	210.5322	428.1968	448.4195	CRTAC1	ASPIC1|CI	cartilage ac	55118	chr10	99629401
AK000262	-3.12871	-1.64557	3.12871	down	-3.00774	-1.58868	3.007741	down	-3.29717	-1.72123	3.297171	down	-3.16969	-1.66434	3.169688	down	-2.291	-1.19598	2.290998	down	-2.20242	-1.13909	2.202419	down	951.8298	915.0281	304.2244	288.6808	415.4652	TMEM104	FLJ00021|	transmemb	54868	chr17	70284233
AK026367	-3.14972	-1.65522	3.149716	down	-2.65195	-1.40705	2.651945	down	-2.91653	-1.54426	2.916535	down	-2.45562	-1.29608	2.455615	down	-3.44457	-1.78432	3.444567	down	-2.9002	-1.53615	2.900199	down	541.7061	456.0966	171.9857	185.7362	157.2639	N/A	N/A	Homo sapi	N/A	chrY	21954251
AK074586	-2.73972	-1.45403	2.739719	down	-2.30367	-1.20393	2.303668	down	-2.75698	-1.46309	2.75698	down	-2.31818	-1.21299	2.318182	down	-2.6511	-1.40659	2.651104	down	-2.22916	-1.1565	2.229157	down	921.0098	774.4226	336.1694	334.0647	347.4061	SCAMP4	FLJ33847|	secretory c	113178	chr19	1856370
AK091878	-6.26776	-2.64795	6.267757	down	-2.13082	-1.09141	2.130824	down	-7.1578	-2.83952	7.157802	down	-2.43341	-1.28298	2.433409	down	-10.5356	-3.39721	10.53564	down	-3.58176	-1.84067	3.581759	down	1612.649	548.246	257.2929	225.2995	153.0661	LOC12387	A-923A4.1	hypothetica	123876	chr16	20394189
AK097866	-2.4492	-1.29231	2.449197	down	-2.57334	-1.36364	2.573343	down	-3.70002	-1.88753	3.700024	down	-3.88757	-1.95887	3.887572	down	-4.09577	-2.03413	4.095767	down	-4.30337	-2.10547	4.303374	down	583.9415	613.5406	238.4216	157.821	142.572	N/A	N/A	Homo sapi	N/A	chr20	59725956
AK122760	-3.22895	-1.69106	3.228946	down	-2.53838	-1.34391	2.53838	down	-3.10721	-1.63562	3.107212	down	-2.44268	-1.28847	2.44268	down	-4.96144	-2.31076	4.961437	down	-3.90035	-1.9636	3.900347	down	743.3167	584.3455	230.2041	239.2231	149.8188	N/A	N/A	Homo sapi	N/A	chr1	54381652
AK124690	-2.04036	-1.02883	2.040363	down	-2.66624	-1.41481	2.666245	down	-2.08072	-1.05708	2.080722	down	-2.71898	-1.44307	2.718984	down	-2.31326	-1.20993	2.313258	down	-3.02285	-1.59591	3.022851	down	281.3968	367.7154	137.9151	135.24	121.6452	N/A	N/A	Homo sapi	N/A	chr11	11248998
AK126846	-2.90564	-1.53886	2.905638	down	-2.45591	-1.29626	2.455913	down	-4.0423	-2.01518	4.042298	down	-3.41664	-1.77258	3.416645	down	-3.72977	-1.89909	3.729767	down	-3.15249	-1.65649	3.152486	down	579.7703	490.0353	199.5328	143.4259	155.4441	N/A	N/A	Homo sapi	N/A	chr3	1.36E+08
AK126967	-2.18319	-1.12644	2.183192	down	-2.6065	-1.38212	2.606503	down	-2.32384	-1.21651	2.323839	down	-2.77442	-1.47219	2.77442	down	-2.2768	-1.18701	2.276796	down	-2.71826	-1.44268	2.718256	down	4518.539	5394.662	2069.694	1944.429	1984.604	N/A	N/A	Homo sapi	N/A	chr17	77555509
AK127390	-2.43517	-1.28402	2.43517	down	-2.34532	-1.22978	2.345319	down	-2.71625	-1.44162	2.716248	down	-2.61603	-1.38738	2.616026	down	-2.30857	-1.207	2.308574	down	-2.22339	-1.15276	2.223394	down	483.9111	466.0561	198.7176	178.1543	209.6147	N/A	N/A	Homo sapi	N/A	chr9	87325324
AK127958	-3.02511	-1.59699	3.025108	down	-4.58614	-2.19728	4.586139	down	-2.2539	-1.17242	2.253902	down	-3.41697	-1.77272	3.416971	down	-2.20193	-1.13877	2.201926	down	-3.33817	-1.73906	3.338174	down	362.6526	549.7902	119.8809	160.8999	164.6979	N/A	N/A	Homo sapi	N/A	chr3	52071149
AK128707	-2.20923	-1.14355	2.209235	down	-2.68065	-1.42258	2.680645	down	-2.00341	-1.00246	2.003407	down	-2.4309	-1.28149	2.430898	down	-2.93205	-1.55191	2.932051	down	-3.5577	-1.83094	3.557697	down	475.7202	577.2303	215.3326	237.4556	162.2483	N/A	N/A	Homo sapi	N/A	chr12	64535993
AK130913	-2.97954	-1.57509	2.979536	down	-2.49586	-1.31953	2.495855	down	-2.91464	-1.54332	2.914638	down	-2.44149	-1.28776	2.441493	down	-3.38279	-1.75821	3.382789	down	-2.83365	-1.50266	2.833647	down	1049.381	879.0302	352.196	360.038	310.2117	N/A	N/A	Homo sapi	N/A	chr8	1.46E+08
AK131481	-2.64532	-1.40344	2.645323	down	-3.95819	-1.98484	3.958193	down	-3.18947	-1.67332	3.189472	down	-4.7724	-2.25472	4.772401	down	-3.68313	-1.88093	3.68313	down	-5.51106	-2.46233	5.511062	down	564.1364	844.1165	213.2581	176.8746	153.1677	C3orf20	DKFZP434	chromoson	84077	chr3	14691651
AK172756	-2.01668	-1.01198	2.016682	down	-2.04966	-1.03538	2.04966	down	-2.32616	-1.21795	2.326163	down	-2.3642	-1.24135	2.364202	down	-3.4448	-1.78442	3.444801	down	-3.50113	-1.80782	3.501133	down	442.1628	449.3935	219.2527	190.0825	128.3566	GLDN	CLOM|CO	gliomedin	342035	chr15	49421168
AX775875	-2.39774	-1.26167	2.397736	down	-2.60005	-1.37854	2.600055	down	-2.23401	-1.15964	2.234014	down	-2.42252	-1.27651	2.422518	down	-2.11244	-1.07891	2.112435	down	-2.29068	-1.19578	2.29068	down	619.4283	671.6951	258.3388	277.2715	293.2295	N/A	N/A	Sequence 1	N/A	chr1	19881321
AY358738	-2.92003	-1.54598	2.920026	down	-2.43294	-1.2827	2.432945	down	-3.2329	-1.69283	3.232904	down	-2.69363	-1.42955	2.693632	down	-3.84847	-1.94428	3.848466	down	-3.20651	-1.68101	3.206513	down	545.7763	454.7368	186.908	168.8192	141.8166	EPHA6	DKFZp434	EPH recep	285220	chr3	98016324
AY366253	-3.38913	-1.76091	3.38913	down	-2.92163	-1.54677	2.921631	down	-5.07321	-2.3429	5.073205	down	-4.3734	-2.12876	4.373404	down	-3.32702	-1.73423	3.327023	down	-2.86809	-1.52009	2.868092	down	2229.991	1922.384	657.9832	439.5625	670.266	KIR3DL1	AMB11|CI	killer cell i	3811	chr19	60019734
AY377973	-2.06891	-1.04887	2.068913	down	-2.38515	-1.25408	2.385155	down	-2.16901	-1.11704	2.169009	down	-2.50055	-1.32225	2.50055	down	-2.65413	-1.40824	2.654132	down	-3.05983	-1.61345	3.059827	down	1124.383	1296.249	543.4654	518.3854	423.6347	ZNFN1A3	AIOLOS	zinc finger	22806	chr17	35175062
AY377979	-2.66975	-1.41671	2.669753	down	-2.33523	-1.22357	2.335231	down	-3.70367	-1.88896	3.703669	down	-3.2396	-1.69581	3.239597	down	-2.93572	-1.55372	2.935723	down	-2.56787	-1.36057	2.567874	down	914.2028	799.6525	342.4298	246.8371	311.4064	ZNFN1A3	AIOLOS	zinc finger	22806	chr17	35175062
BC007600	-4.37971	-2.13084	4.379712	down	-2.95055	-1.56098	2.950551	down	-3.62639	-1.85853	3.626388	down	-2.44305	-1.28868	2.443047	down	-3.52564	-1.81788	3.525639	down	-2.37517	-1.24803	2.375174	down	845.0265	569.2825	192.9411	233.0215	239.6803	HOXA10	HOX1|HO	homeobox	3206	chr7	27177380
BC008600	-2.62394	-1.39174	2.623944	down	-2.84471	-1.50828	2.844708	down	-4.64188	-2.21471	4.64188	down	-5.03242	-2.33125	5.032423	down	-2.13189	-1.09213	2.131887	down	-2.31125	-1.20867	2.311252	down	28004.05	30360.16	10672.5	6032.911	13135.81	CCL5	D17S136E	chemokine	6352	chr17	31223056
BC010446	-3.42817	-1.77744	3.428171	down	-3.55212	-1.82868	3.552124	down	-2.87088	-1.52149	2.87088	down	-2.97468	-1.57274	2.974683	down	-3.29168	-1.71882	3.291681	down	-3.4107	-1.77007	3.410698	down	1063.961	1102.431	310.3581	370.6044	323.2272	C19orf4	BSMAP	chromoson	25789	chr19	18584740
BC016967	-2.62325	-1.39135	2.623246	down	-2.07842	-1.05549	2.07842	down	-3.29674	-1.72104	3.296742	down	-2.61204	-1.38518	2.612037	down	-2.56707	-1.36013	2.567074	down	-2.03391	-1.02426	2.033915	down	629.2777	498.582	239.8851	190.8787	245.1342	RPUSD2	C15orf19|C	RNA pseuc	27079	chr15	38648822
BC021580	-2.52455	-1.33603	2.52455	down	-2.48529	-1.31342	2.485294	down	-2.50137	-1.32272	2.50137	down	-2.46247	-1.30011	2.462474	down	-2.33828	-1.22545	2.338278	down	-2.30192	-1.20284	2.301919	down	511.4845	503.531	202.6042	204.4818	218.744	C9orf90	DKFZp762	chromoson	203245	chr9	1.3E+08
BC022101	-44.1489	-5.46431	44.14891	down	-5.62934	-2.49297	5.62934	down	-18.085	-4.17672	18.08497	down	-2.30598	-1.20538	2.305979	down	-32.2851	-5.01279	32.28505	down	-4.1166	-2.04145	4.116603	down	7126.697	908.711	161.4241	394.0673	220.7429	UNQ470	-	GAAI470	375704	chr9	34511041
BC022407	-3.00703	-1.58834	3.007033	down	-3.66055	-1.87206	3.660546	down	-4.83658	-2.27399	4.836576	down	-5.8877	-2.5577	5.887699	down	-3.11696	-1.64014	3.116962	down	-3.79437	-1.92386	3.794365	down	1477.961	1799.163	491.5013	305.58	474.1671	SH2D1B	EAT2	SH2 domai	117157	chr1	1.61E+08
BC026032	-2.77431	-1.47213	2.774307	down	-2.80865	-1.48988	2.808655	down	-3.33247	-1.73659	3.332465	down	-3.37372	-1.75434	3.373723	down	-3.63613	-1.8624	3.63613	down	-3.68115	-1.88016	3.681148	down	363.688	368.1906	131.0915	109.1348	100.0206	PRRG2	PRGP2	proline ricl	5639	chr19	54776441
BC028735	-2.11309	-1.07935	2.113087	down	-2.30044	-1.20191	2.300439	down	-2.48085	-1.31083	2.480846	down	-2.7008	-1.43339	2.700804	down	-2.50067	-1.32232	2.500671	down	-2.72239	-1.44487	2.722387	down	2992.205	3257.501	1416.035	1206.123	1196.561	LOC40143	-	hypothetica	401431	chr7	1.49E+08
BC030782	-2.46898	-1.30392	2.468983	down	-2.57536	-1.36477	2.575356	down	-3.16884	-1.66396	3.168841	down	-3.30537	-1.72481	3.305366	down	-2.26504	-1.17954	2.265042	down	-2.36263	-1.24039	2.362628	down	765.1513	798.1169	309.9055	241.461	337.8089	N/A	N/A	Homo sapi	N/A	chr20	34019946
BC030994	-2.66049	-1.41169	2.660495	down	-2.60079	-1.37895	2.600785	down	-2.14258	-1.09935	2.142581	down	-2.0945	-1.0666	2.094495	down	-3.17764	-1.66796	3.177644	down	-3.10633	-1.63521	3.106328	down	1109.525	1084.624	417.0371	517.845	349.166	CGB8	-	chorionic g	94115	chr19	54242709
BC032422	-2.76169	-1.46555	2.761692	down	-2.49608	-1.31957	2.496081	down	-3.94784	-1.98106	3.947836	down	-3.56815	-1.83517	3.568146	down	-2.75468	-1.46188	2.754678	down	-2.48974	-1.316	2.489742	down	1727.428	1561.29	625.4963	437.5633	627.0889	KIR2DL3	CD158B2|(	killer cell i	3804	chr19	59941809
BC034459	-3.098	-1.63134	3.098	down	-3.16508	-1.66224	3.16508	down	-2.59374	-1.37503	2.593741	down	-2.6499	-1.40594	2.649902	down	-2.09457	-1.06665	2.094568	down	-2.13992	-1.09756	2.139921	down	501.401	512.2576	161.8467	193.3119	239.3816	N/A	N/A	Homo sapi	N/A	chr1	6191264
BC035371	-2.28106	-1.1897	2.281061	down	-2.08601	-1.06074	2.086006	down	-2.46689	-1.30269	2.466887	down	-2.25594	-1.17373	2.255942	down	-3.2247	-1.68917	3.224703	down	-2.94896	-1.5602	2.948957	down	4185.752	3827.826	1835.002	1696.775	1298.027	HSPC047	-	HSPC047 p	29060	chr7	1.02E+08
BC035680	-5.07945	-2.34467	5.079446	down	-2.41321	-1.27095	2.413209	down	-5.39214	-2.43086	5.392139	down	-2.56177	-1.35714	2.561768	down	-5.42887	-2.44065	5.42887	down	-2.57922	-1.36693	2.579218	down	35102.01	16676.72	6910.598	6509.849	6465.804	TRAJ17	-	T cell recep	28738	chr14	21261799
BC036660	-2.68562	-1.42525	2.685616	down	-2.78786	-1.47916	2.787864	down	-2.85672	-1.51436	2.856724	down	-2.96549	-1.56827	2.965487	down	-3.12395	-1.64337	3.123954	down	-3.24289	-1.69728	3.242891	down	528.4279	548.5465	196.7623	184.9769	169.1536	MAPKBP1	-	mitogen ac	23005	chr15	39853923
BC043281	-3.86529	-1.95058	3.865293	down	-3.86053	-1.9488	3.860531	down	-2.29212	-1.19668	2.292121	down	-2.2893	-1.1949	2.289296	down	-2.99161	-1.58092	2.991606	down	-2.98792	-1.57914	2.98792	down	401.4266	400.932	103.8541	175.1333	134.1843	C10orf62	bA548K23	chromoson	414157	chr10	99338993
BC048769	-16.0564	-4.00508	16.05639	down	-3.81874	-1.9331	3.818741	down	-25.0704	-4.64791	25.07038	down	-5.96257	-2.57593	5.962566	down	-9.90285	-3.30784	9.902846	down	-2.35522	-1.23586	2.355225	down	8966.535	2132.539	558.4404	357.6545	905.4503	TCF7	MGC4773	transcriptic	6932	chr5	1.33E+08
BC051874	-2.26107	-1.177	2.261068	down	-2.20511	-1.14085	2.205106	down	-2.19661	-1.13528	2.196613	down	-2.14225	-1.09912	2.142246	down	-2.05534	-1.03938	2.055343	down	-2.00447	-1.00322	2.004472	down	423.0375	412.5671	187.0963	192.5863	205.8233	CROT	COT	carnitine O	54677	chr7	86812944
BC053323	-2.30254	-1.20322	2.302537	down	-3.26476	-1.70698	3.264756	down	-2.15108	-1.10506	2.151075	down	-3.05	-1.60881	3.049999	down	-2.62215	-1.39075	2.62215	down	-3.71793	-1.8945	3.717933	down	1204.873	1708.383	523.2805	560.1259	459.4981	N4BP3	KIAA0341	Nedd4 binc	23138	chr5	1.77E+08
BC062375	-2.18493	-1.12759	2.184933	down	-2.55275	-1.35205	2.552751	down	-2.42192	-1.27615	2.421917	down	-2.82963	-1.50061	2.82963	down	-2.10534	-1.07405	2.105338	down	-2.45976	-1.29852	2.459756	down	1051.372	1228.363	481.1917	434.1072	499.3839	ARTN	EVN|NBN	artemin	9048	chr1	44172070
BC066595	-3.57574	-1.83824	3.575742	down	-3.21577	-1.68517	3.215773	down	-9.9349	-3.3125	9.934896	down	-8.93475	-3.15943	8.934752	down	-3.57512	-1.83799	3.575125	down	-3.21522	-1.68492	3.215218	down	1823.849	1640.242	510.0617	183.5801	510.1497	SH2D1B	EAT2	SH2 domai	117157	chr1	1.61E+08
BC068078	-4.00424	-2.00153	4.004236	down	-4.36834	-2.12708	4.368339	down	-2.24934	-1.1695	2.249338	down	-2.45387	-1.29506	2.453869	down	-3.01491	-1.59212	3.014911	down	-3.28906	-1.71767	3.289055	down	1122.431	1224.493	280.3109	499.0051	372.2932	NPM2	MGC7865	nucleophos	10361	chr8	21938307
BC069357	-6.61513	-2.72577	6.615128	down	-3.15678	-1.65845	3.156778	down	-5.43648	-2.44267	5.436483	down	-2.59432	-1.37536	2.594322	down	-4.36186	-2.12494	4.361858	down	-2.0815	-1.05763	2.081505	down	2014.785	961.4672	304.5723	370.6044	461.9097	UBASH3A	TULA	ubiquitin a	53347	chr21	42697092
BC069474	-3.48486	-1.8011	3.484858	down	-2.57283	-1.36336	2.572834	down	-3.71827	-1.89463	3.718272	down	-2.74516	-1.45689	2.745162	down	-4.83761	-2.27429	4.837605	down	-3.57155	-1.83655	3.571553	down	1227.445	906.2095	352.2223	330.1116	253.7298	MEOX1	MOX1	mesenchyn	4222	chr17	39074754
BC070329	-7.25367	-2.85871	7.253673	down	-2.41794	-1.27378	2.417935	down	-9.91493	-3.3096	9.914925	down	-3.30504	-1.72467	3.305035	down	-6.30619	-2.65677	6.306187	down	-2.1021	-1.07183	2.102101	down	1851.916	617.3167	255.3074	186.7807	293.6666	TRA@	FLJ22602||	T cell recep	6955	chr14	21661115
BC070387	-10.8614	-3.44114	10.86141	down	-3.4822	-1.8	3.4822	down	-12.5563	-3.65034	12.55628	down	-4.02558	-2.0092	4.025582	down	-6.3201	-2.65995	6.320096	down	-2.02624	-1.01881	2.026242	down	43133.14	13828.62	3971.229	3435.185	6824.761	TRBV3-1	TCRBV3S	T cell recep	28619	chr7	1.42E+08
BC073797	-8.19858	-3.03537	8.198584	down	-4.52566	-2.17813	4.525663	down	-6.6272	-2.7284	6.627199	down	-3.65825	-1.87115	3.65825	down	-4.15062	-2.05333	4.150625	down	-2.29117	-1.19608	2.291168	down	24671.23	13618.65	3009.206	3722.723	5943.979	TRBC1	MGC8881	T cell recep	28639	chr7	1.42E+08
BC073930	-8.72693	-3.12548	8.726934	down	-3.39772	-1.76457	3.397717	down	-8.03112	-3.0056	8.031118	down	-3.12681	-1.64469	3.12681	down	-6.67133	-2.73798	6.671333	down	-2.5974	-1.37707	2.597395	down	2970.693	1156.6	340.4051	369.8978	445.2923	TRBV19	TCRBV17	T cell recep	28568	chr7	1.42E+08
BC075000	-2.05781	-1.04111	2.057808	down	-4.40904	-2.14047	4.409044	down	-2.49046	-1.31641	2.49046	down	-5.33604	-2.41577	5.336042	down	-2.27165	-1.18374	2.27165	down	-4.86722	-2.2831	4.867221	down	360.2301	771.8264	175.0553	144.644	158.5764	PRSS21	ESP-1|TES	protease, se	10942	chr16	2807228
BC093850	-2.11874	-1.0832	2.118737	down	-2.18534	-1.12786	2.185342	down	-3.08455	-1.62506	3.084546	down	-3.18151	-1.66971	3.181513	down	-2.93246	-1.55221	2.932457	down	-3.02464	-1.59676	3.024643	down	3322.706	3427.16	1568.249	1077.211	1133.08	C18orf23	FLJ34218	chromoson	147341	chr18	42168795
BC093855	-3.74845	-1.90629	3.748449	down	-2.09967	-1.07016	2.099668	down	-4.40614	-2.13952	4.40614	down	-2.46807	-1.30338	2.468068	down	-4.46592	-2.15896	4.46592	down	-2.50155	-1.32282	2.501554	down	1105.604	619.2965	294.9498	250.9236	247.5647	DSCR10	-	Down sync	259234	chr21	38502098
BC095539	-2.08309	-1.05872	2.083088	down	-2.48816	-1.31508	2.48816	down	-2.31535	-1.21123	2.315352	down	-2.76559	-1.46759	2.765589	down	-3.36167	-1.74918	3.361674	down	-4.01538	-2.00554	4.015376	down	462.5496	552.4957	222.0499	199.7751	137.595	PRLHR	GPR10|GR	prolactin re	2834	chr10	1.2E+08
BC098107	-2.5834	-1.36927	2.583401	down	-3.12026	-1.64167	3.120262	down	-3.46796	-1.79409	3.467959	down	-4.18864	-2.06648	4.188644	down	-3.44848	-1.78596	3.448478	down	-4.16511	-2.05836	4.165113	down	752.8642	909.3185	291.4237	217.0914	218.3178	KIR3DL1	AMB11|CI	killer cell i	3811	chr19_rand	212785

BC098130	-2.33724	-1.22481	2.337245	down	-2.44875	-1.29205	2.448753	down	-2.26238	-1.17784	2.262383	down	-2.37032	-1.24508	2.37032	down	-4.1829	-2.0645	4.182898	down	-4.38246	-2.13174	4.382462	down	532.2681	557.6624	227.7332	235.2688	127.2487	IL1F9	IL-1F9|IL-	interleukin	56300	chr2	1.13E+08
BC098247	-2.47965	-1.31014	2.479652	down	-2.40119	-1.26375	2.401192	down	-2.59737	-1.37705	2.59737	down	-2.51519	-1.33066	2.515185	down	-3.92019	-1.97093	3.920195	down	-3.79615	-1.92454	3.796154	down	603.6314	584.5316	243.4339	232.401	153.98	TUBAL3	FLJ21665|l	tubulin, alp	79861	chr10	5425232
BC098288	-2.07074	-1.05015	2.070741	down	-2.6921	-1.42873	2.692103	down	-2.95687	-1.56407	2.956875	down	-3.84414	-1.94266	3.844137	down	-2.38064	-1.25135	2.380635	down	-3.09499	-1.62993	3.094987	down	526.4719	684.4491	254.2432	178.0501	221.1476	KIR3DL1	AMB11|CI	killer cell i	3811	chr19_rand	212785
BC100294	-7.32134	-2.87211	7.321339	down	-3.70206	-1.88833	3.702056	down	-7.84078	-2.971	7.840784	down	-3.96471	-1.98722	3.964715	down	-4.98364	-2.3172	4.983643	down	-2.51999	-1.33342	2.519993	down	35228.19	17813.23	4811.714	4492.942	7068.762	TRA@	FLJ22602|l	T cell rece	6955	chr14	21961352
BC100911	-11.2134	-3.48716	11.21345	down	-4.39859	-2.13704	4.398593	down	-15.9694	-3.99724	15.96944	down	-6.26418	-2.64713	6.264182	down	-7.89679	-2.98127	7.896788	down	-3.0976	-1.63115	3.097599	down	4300.045	1686.738	383.4722	269.2671	544.5309	CD8B	CD8B1|LY	CD8b mole	926	chr2	86896190
BC101214	-2.86649	-1.51928	2.866489	down	-2.63548	-1.39806	2.635477	down	-2.77777	-1.47393	2.777772	down	-2.55391	-1.35271	2.55391	down	-2.81758	-1.49446	2.817584	down	-2.59051	-1.37324	2.590514	down	317.7477	292.1403	110.8491	114.3894	112.7731	C17orf54	FLJ40319|l	chromoson	283982	chr17	69258063
BC101465	-4.17825	-2.0629	4.178249	down	-2.56812	-1.36071	2.568124	down	-5.68515	-2.5072	5.685154	down	-3.49433	-1.80502	3.494329	down	-3.46002	-1.79078	3.460017	down	-2.12667	-1.0886	2.126669	down	1024.539	629.7238	245.2077	180.2131	296.1081	PRKCQ	MGC1265	protein kin	5588	chr10	6510125
BC101643	-2.82391	-1.49769	2.823906	down	-2.88472	-1.52843	2.884719	down	-3.71986	-1.89525	3.719855	down	-3.79996	-1.92599	3.799963	down	-2.2628	-1.17811	2.262798	down	-2.31153	-1.20885	2.311528	down	523.2895	534.5586	185.307	140.6747	231.2577	SEMA7A	CD108|CD	semaphorir	8482	chr15	72489974
BC101831	-2.01198	-1.00862	2.011979	down	-3.00766	-1.58864	3.007655	down	-2.03739	-1.02672	2.037392	down	-3.04564	-1.60675	3.045645	down	-2.18842	-1.12989	2.188424	down	-3.27142	-1.70992	3.271418	down	1107.432	1655.471	550.419	543.5534	506.0407	IL1F8	FIL1|FIL1-	interleukin	27177	chr2	1.14E+08
BC101977	-2.28755	-1.1938	2.287546	down	-2.17453	-1.12071	2.174533	down	-4.8036	-2.26412	4.803604	down	-4.56629	-2.19102	4.566288	down	-3.04359	-1.60577	3.043592	down	-2.89323	-1.53268	2.893227	down	864.1406	821.4488	377.7587	179.8942	283.9213	KIR2DS4	CD158I|KI	killer cell i	3809	chr19	60036020
BC103978	-2.64895	-1.40542	2.648946	down	-2.15728	-1.10921	2.157279	down	-3.01223	-1.59083	3.012234	down	-2.45314	-1.29463	2.453138	down	-3.64083	-1.86427	3.640832	down	-2.96506	-1.56806	2.965063	down	999.3358	813.8506	377.2579	331.759	274.48	FGF9	GAF|HBF(	fibroblast g	2254	chr13	21144028
BC107951	-3.0063	-1.58799	3.006296	down	-2.58727	-1.37143	2.58727	down	-4.57748	-2.19455	4.577477	down	-3.93945	-1.978	3.939455	down	-2.63293	-1.39667	2.632931	down	-2.26595	-1.18011	2.265945	down	2190.605	1885.272	728.6725	478.5617	832.0026	KIR3DL2	CD158K|C	killer cell i	3812	chr19	60053717
BC108917	-3.925	-1.97269	3.924997	down	-2.71467	-1.44078	2.714668	down	-6.37935	-2.67341	6.379348	down	-4.41219	-2.14149	4.412185	down	-4.5192	-2.17607	4.519203	down	-3.12564	-1.64415	3.125642	down	1342.62	928.6043	342.0692	210.4636	297.0923	KIR2DS2	183ACTI|(	killer cell i	3807	chr19_rand	181166
BC110073	-3.2862	-1.71642	3.286203	down	-2.56498	-1.35895	2.564976	down	-4.15001	-2.05311	4.150007	down	-3.2392	-1.69564	3.2392	down	-4.20056	-2.07058	4.200555	down	-3.27865	-1.7131	3.278654	down	498.3804	389.0002	151.6584	120.0914	118.6463	LOC28494	-	similar to E	284948	chr2	85511015
BC110354	-10.7694	-3.42886	10.76935	down	-4.10186	-2.03628	4.101862	down	-10.6993	-3.41944	10.6993	down	-4.07518	-2.02686	4.075178	down	-6.36124	-2.66931	6.361243	down	-2.42289	-1.27673	2.422888	down	39120.84	14900.45	3632.608	3656.393	6149.873	TRA@	FLJ22602|l	T cell rece	6955	chr14	21661176
BC112242	-2.0631	-1.04482	2.063103	down	-2.40883	-1.26833	2.408831	down	-2.14362	-1.10005	2.143623	down	-2.50284	-1.32357	2.502844	down	-3.95058	-1.98207	3.950582	down	-4.61261	-2.20558	4.612608	down	1037.653	1211.539	502.9572	484.065	262.6582	NYX	CLRP|CSN	nyctalopin	60506	chrX	41192042
CR604131	-2.94348	-1.55752	2.943481	down	-2.57572	-1.36497	2.575716	down	-2.90229	-1.53719	2.90229	down	-2.53967	-1.34464	2.539672	down	-2.75444	-1.46176	2.754436	down	-2.41029	-1.26921	2.410291	down	450.8565	394.5255	153.1712	155.3451	163.6838	CLUAP1	FLJ13297|l	clusterin as	23059	chr16	3491012
L76667	-2.18598	-1.12828	2.185981	down	-2.33168	-1.22137	2.331675	down	-3.85093	-1.94521	3.850932	down	-4.10759	-2.03829	4.107595	down	-2.70989	-1.43823	2.709887	down	-2.8905	-1.53132	2.890499	down	2368.696	2526.568	1083.585	615.0967	874.094	KIR2DS2	183ACTI|(	killer cell i	3807	chr19_rand	274145
L76668	-2.43553	-1.28424	2.435534	down	-2.6032	-1.38029	2.603202	down	-5.06159	-2.33959	5.061588	down	-5.41004	-2.43564	5.41004	down	-2.54997	-1.35048	2.54997	down	-2.72552	-1.44653	2.725516	down	1803.183	1927.319	740.3646	356.2484	707.1389	KIR2DS2	183ACTI|(	killer cell i	3807	chr19_rand	164617
NM_00023	-2.52466	-1.33609	2.524658	down	-2.78072	-1.47546	2.780721	down	-2.55288	-1.35212	2.552877	down	-2.8118	-1.49149	2.811801	down	-4.76565	-2.25267	4.765649	down	-5.249	-2.39204	5.249003	down	653.0253	719.2582	258.6589	255.7998	137.0276	LIPC	HL|HTGL|	lipase, hep	3990	chr15	56511466
NM_00045	-3.64397	-1.86551	3.643967	down	-2.73834	-1.4533	2.73834	down	-3.56327	-1.8332	3.563267	down	-2.6777	-1.42099	2.677696	down	-4.04158	-2.01492	4.04158	down	-3.03713	-1.60271	3.037134	down	513.1785	385.6393	140.8296	144.0191	126.9747	SLC5A5	NIS	solute carri	6528	chr19	17843781
NM_00048	-2.68381	-1.42428	2.683807	down	-2.14285	-1.09953	2.142852	down	-2.90855	-1.5403	2.908549	down	-2.32229	-1.21555	2.322294	down	-4.27388	-2.09555	4.273881	down	-3.41243	-1.7708	3.412426	down	2997.053	2392.959	1116.717	1030.429	701.2485	AQP2	AQP-CD|M	aquaporin 2	359	chr12	48630795
NM_00093	-2.05958	-1.04235	2.059583	down	-2.67595	-1.42005	2.675953	down	-2.18704	-1.12898	2.187044	down	-2.84156	-1.50668	2.84156	down	-2.35585	-1.23625	2.355851	down	-3.06089	-1.61395	3.060885	down	344.0741	447.0451	167.0601	157.3238	146.0509	POMC	ACTH|CLI	proopiome	5443	chr2	25237291
NM_0010(	-2.30021	-1.20176	2.300208	down	-2.35107	-1.23332	2.351066	down	-2.24048	-1.16381	2.240482	down	-2.29002	-1.19536	2.290019	down	-2.87117	-1.52164	2.871172	down	-2.93465	-1.55319	2.934654	down	434.8384	444.4527	189.0431	194.0826	151.4498	FAM26C	MGC3951	family with	255022	chr10	1.05E+08
NM_0010(	-2.34588	-1.23013	2.345883	down	-2.22671	-1.15492	2.226715	down	-2.51289	-1.32935	2.51289	down	-2.38524	-1.25413	2.385238	down	-2.67055	-1.41714	2.67055	down	-2.53489	-1.34192	2.534889	down	10726.65	10181.74	4572.54	4268.648	4016.642	FLJ45966	-	FLJ45966 p	401120	chr4	8559454
NM_0010(	-2.38127	-1.25173	2.381271	down	-2.61669	-1.38774	2.616692	down	-2.59209	-1.37412	2.59209	down	-2.84835	-1.51013	2.848353	down	-2.07928	-1.05608	2.079277	down	-2.28484	-1.19209	2.284841	down	1051.761	1155.741	441.6803	405.7577	505.83	FCRL6	FLJ16056|l	Fc receptor	343413	chr1	1.58E+08
NM_0010(	-3.17402	-1.66631	3.174016	down	-2.65388	-1.4081	2.65388	down	-2.90378	-1.53793	2.903784	down	-2.42793	-1.27973	2.427932	down	-2.93465	-1.55319	2.934646	down	-2.45374	-1.29498	2.453736	down	1546.051	1292.694	487.096	532.4261	526.827	OR13F1	OR9-6	olfactory re	138805	chr9	1.06E+08
NM_0010(	-3.65606	-1.87029	3.656063	down	-3.13065	-1.64646	3.130652	down	-4.68044	-2.22664	4.680437	down	-4.00781	-2.00282	4.007814	down	-3.67105	-1.87619	3.671048	down	-3.14348	-1.65236	3.143485	down	1076.46	921.7626	294.4315	229.9913	293.2295	OR2W5	OR2W5P|(	olfactory re	441932	chr1	2.46E+08
NM_0010(	-2.09404	-1.06629	2.094045	down	-2.11471	-1.08046	2.114706	down	-3.58655	-1.8426	3.586546	down	-3.62193	-1.85676	3.621934	down	-2.43177	-1.282	2.431766	down	-2.45576	-1.29617	2.45576	down	455.4007	459.894	217.4742	126.9747	187.2716	DNM1	DNM	dynamin 1	1759	chr9	1.3E+08
NM_0010(	-2.31399	-1.21038	2.313992	down	-2.22801	-1.15575	2.228005	down	-2.47392	-1.3068	2.473918	down	-2.38199	-1.25217	2.381989	down	-2.69964	-1.43277	2.69964	down	-2.59932	-1.37814	2.599323	down	483.3897	465.4271	208.8986	195.3943	179.0571	LOC19843	-	bA299N6.3	198437	chr20	62185176
NM_0010(	-2.08275	-1.05849	2.082754	down	-2.72125	-1.44427	2.721249	down	-2.12715	-1.08892	2.127147	down	-2.77925	-1.4747	2.779251	down	-2.19443	-1.13385	2.194434	down	-2.86717	-1.51963	2.867167	down	717.9685	938.071	344.7207	337.5266	327.177	IGFBPL1	IGFBP-RP	insulin-like	347252	chr9	38398990
NM_0010(	-3.10172	-1.63307	3.101719	down	-2.04651	-1.03316	2.046505	down	-3.61285	-1.85314	3.612854	down	-2.38375	-1.25323	2.38375	down	-5.45538	-2.44768	5.455376	down	-3.59944	-1.84777	3.59944	down	801.2945	528.6917	258.3388	221.7899	146.8816	LOC40143	-	hypothetica	401431	chr7	1.49E+08
NM_0010(	-2.80059	-1.48573	2.800587	down	-2.10753	-1.07555	2.107531	down	-3.44362	-1.78392	3.443616	down	-2.59143	-1.37375	2.591431	down	-5.06596	-2.34083	5.065956	down	-3.81229	-1.93066	3.812294	down	660.8292	497.295	235.961	191.8998	130.4451	RXRG	NR2B3|RX	retinoid X	6258	chr1	1.64E+08
NM_00101	-2.73853	-1.4534	2.738528	down	-2.48792	-1.31494	2.487919	down	-2.31844	-1.21316	2.318444	down	-2.10628	-1.0747	2.106278	down	-2.54331	-1.34671	2.54331	down	-2.31057	-1.20825	2.310566	down	950.5635	863.5753	347.1075	410.0007	373.7505	EME2	FLJ00151|g	essential m	197342	chr16	1763229
NM_00101	-3.74643	-1.90551	3.746425	down	-3.30989	-1.72678	3.309887	down	-3.41245	-1.77081	3.412446	down	-3.01482	-1.59207	3.014823	down	-2.49271	-1.31772	2.492713	down	-2.20226	-1.13898	2.202258	down	19695.82	17400.84	5257.231	5771.761	7901.361	IL32	IL-32alpha	interleukin	9235	chr16	3055785
NM_00102	-4.05238	-2.01877	4.052377	down	-3.84136	-1.94162	3.841362	down	-2.78607	-1.47823	2.786073	down	-2.641	-1.40108	2.640998	down	-3.34644	-1.74263	3.34644	down	-3.17218	-1.66548	3.172185	down	1191.803	1129.744	294.0998	427.7716	356.1406	NANOS2	NOS2	nanos hom	339345	chr19	51108311
NM_00103	-2.11696	-1.08199	2.116958	down	-2.23474	-1.1601	2.234736	down	-2.78094	-1.47557	2.780942	down	-2.93566	-1.55369	2.935661	down	-2.32315	-1.21608	2.32315	down	-2.4524	-1.29419	2.452399	down	358.2082	378.1372	169.2089	128.8082	154.1907	C17orf47	FLJ40121	chromoson	284083	chr17	53973946
NM_00103	-4.55539	-2.18758	4.555392	down	-2.51597	-1.33111	2.51597	down	-8.13878	-3.02481	8.138778	down	-4.4951	-2.16835	4.495095	down	-3.88173	-1.9567	3.881729	down	-2.1439	-1.10024	2.143902	down	1028.139	567.8471	225.6971	126.3259	264.8662	TNFRSF25	APO-3|DD	tumor necr	8718	chr1	6446757
NM_00103	-4.27289	-2.09521	4.27289	down	-2.36454	-1.24156	2.364541	down	-6.78873	-2.76314	6.788734	down	-3.75676	-1.90949	3.756764	down	-5.49261	-2.45749	5.492614	down	-3.03951	-1.60384	3.039514	down	774.9844	428.8625	181.3724	114.1574	141.0957	FLJ45825	-	hypothetica	646888	chr6	37583101
NM_00104	-9.60371	-3.26359	9.603709	down	-4.37915	-2.13065	4.379152	down	-7.14504	-2.83694	7.145043	down	-3.25804	-1.704	3.258036	down	-5.32554	-2.41293	5.325543	down	-2.42837	-1.27999	2.42837	down	30496.96	13906.17	3175.539	4268.268	5726.544	LOC64735	MGC1174	similar to T	647353	chr7	1.42E+08
NM_0017(	-3.75783	-1.9099	3.757827	down	-2.07747	-1.05483	2.07747	down	-5.14308	-2.36263	5.143077	down	-2.84329	-1.50756	2.843289	down	-4.44452	-2.15203	4.444521	down	-2.4571	-1.29696	2.457101	down	3810.363	2106.514	1013.98	740.8723	857.3169	CD2	SRBC|T11	CD2 molec	914	chr1	1.17E+08
NM_0017(	-5.48497	-2.45548	5.484969	down	-4.38388	-2.13221	4.383879	down	-7.9518	-2.99128	7.9518	down	-6.3555	-2.66801	6.355501	down	-3.18632	-1.67189	3.186324	down	-2.54668	-1.34862	2.54668	down	5521.072	4412.734	1006.582	694.3173	1732.74	CD8A	CD8|Leu2|	CD8a mole	925	chr2	86865243
NM_0019(	-3.25784	-1.70392	3.257843	down	-3.2756	-1.71176	3.2756	down	-2.41605	-1.27265	2.416046	down	-2.42921	-1.28049	2.429215	down	-2.35471	-1.23555	2.354712	down	-2.36755	-1.24339	2.367546	down	340.099	341.9527	104.3939	140.7668	144.4334	EVX1	-	eve, even-s	2128	chr7	27248945
NM_0021(	-9.0725	-3.1815	9.072495	down	-2.03598	-1.02572	2.035979	down	-10.17	-3.34625	10.17002	down	-2.28228	-1.19047	2.282276	down	-9.68919	-3.27638	9.689188	down	-2.17437	-1.1206	2.174372	down	3425.468	768.7169	377.5663	336.8202	353.5351	GZMK	TRYP2	granzyme K	3003	chr5	54355863
NM_00225	-2.28533	-1.1924	2.285331	down	-2.13719	-1.09572	2.137195	down	-3.96547	-1.98749	3.965472	down	-3.70843	-1.89081	3.708429	down	-2.33218	-1.22168	2.332179	down	-2.18101	-1.12499	2.181007	down	1165.961	1090.383	510.1935	294.0283	499.9447	KIR2DL4	103AS|15.2	killer cell i	3805	chr19	60006877
NM_00237	-5.15049	-2.36471	5.150494	down	-3.40395	-1.76721	3.403951	down	-4.40874	-2.14036	4.408735	down	-2.91372	-1.54286	2.913723	down	-3.46723	-1.79378	3.467227	down	-2.29148	-1.19628	2.291483	down	1975.438	1305.563	383.5435	448.0738	569.7459	MATK	CHK|CTK|	megakaryo	4145	chr19	3728966
NM_0032(	-13.8388	-3.79064	13.83876	down	-4.37133	-2.12807	4.371334	down	-24.3324	-4.60481	24.33239	down	-7.68602	-2.94224	7.686021	down	-7.68505	-2.94205	7.685051	down	-2.42752	-1.27949	2.427524	down	7341.999	2319.162	530.5388	301.7377	955.3611	TCF7	MGC4773	transcriptic	6932	chr5	1.33E+08
NM_0034(	-3.13638	-1.6491	3.136379	down	-2.18609	-1.12835	2.186088	down	-3.87898	-1.95568	3.878983	down	-2.70369	-1.43493	2.703691	down	-4.41007	-2.1408	4.410067	down	-3.07386	-1.62005	3.073862	down	580.3105	404.4824	185.0256	149.6038	131.5877	PAX8	-	paired box	7849	chr2	1.14E+08
NM_00369	-2.65559	-1.40903	2.655594	down	-2.40986	-1.26895	2.409857	down	-3.1907	-1.67387	3.1907	down	-2.89545	-1.53379	2.895447	down	-2.74087	-1.45463	2.740866	down	-2.48724	-1.31454	2.487238	down	1483.905	1346.591	558.7845	465.0719	541.4	LY6D	E48	lymphocyte	8581	chr8	1.44E+08
NM_00412	-4.10606	-2.03776	4.106062	down	-3.0609	-1.61396	3.060902	down	-3.08924	-1.62725	3.089244	down	-2.30291	-1.20346	2.302906	down	-3.96467	-1.9872	3.96467	down	-2.9555	-1.5634	2.9555	down	661.9777	493.4774	161.2196	214.2847	166.9692	GHSR	-	growth hor	2693	chr3	1.74E+08
NM_00417	-2.35864	-1.23796	2.35864	down	-3.37976	-1.75692	3.379758	down	-2.89186	-1.532	2.891858	down	-4.14382	-2.05096	4.14382	down	-2.20238	-1.13907	2.202384	down	-3.15586	-1.65803	3.155855	down	368.0254	527.3535	156.0329	127.2626	167.1032	SLC7A4	CAT-4|CA	solute carri	6545	chr22	19713007
NM_00477	-3.50816	-1.81071	3.508157	down	-2.09522	-1.0671	2.095223	down	-4.80688	-2.2651	4.806884	down	-2.87088	-1.52149	2.870879	down	-4.74081	-2.24513	4.740814	down	-2.83142	-1.50153	2.831419	down	620.9522	370.8595	177.0024	129.1798	130.9801	MMP20	MMP-20	matrix met	9313	chr11	1.02E+08
NM_00482	-4.05477	-2.01962	4.054768	down	-2.57575	-1.36499	2.575752	down	-4.06121	-2.02191	4.061214	down	-2.57985	-1.36729	2.579847	down	-3.53455	-1.82152	3.534545	down	-2.24529	-1.1669	2.245285	down	465.2807	295.565	114.749	114.5669	131.6381	NCR2	CD336|LY	natural cyto	9436	chr6	41411504
NM_0048(	-3.36297	-1.74973	3.362965	down	-2.42533	-1.27818	2.425325	down	-4.41659	-2.14293	4.416593	down	-3.18519	-1.67138	3.185187	down	-2.87334	-1.52273	2.873344	down	-2.07222	-1.05118	2.072217	down	670.8072	483.7771	199.469	151.8834	233.4587	GAL3ST1	CST	galactose-3	9514	chr22	29280623
NM_00493	-10.3033	-3.36503	10.30326	down	-5.28368	-2.40154	5.283679	down	-11.4902	-3.52233	11.49018	down	-5.89235	-2.55884	5.892348	down	-6.55496	-2.71259	6.554957	down	-3.36149	-1.7491	3.361487	down	5385.584	2761.814	522.7066	468.7119	821.6047	CD8B	CD8B1|LY	CD8b mole	926	chr2	86922219
NM_0050(	-2.20778	-1.1426	2.207784	down	-2.3418	-1.22762	2.341805	down	-2.33565	-1.22383	2.335652	down	-2.47744	-1.30885	2.477435	down	-2.19161	-1.13199	2.191605	down	-2.32464	-1.21701	2.324644	down	542.9146	575.8716	245.9094	232.4467	247.7246	SIM1	-	single-mind	6492	chr6	1.01E+08
NM_0051(	-3.39937	-1.76527	3.399372	down	-2.7177	-1.44238	2.717696	down	-3.53139	-1.82024	3.531388	down	-2.82324	-1.49735	2.823238	down	-4.11	-2.03914	4.109998	down	-3.28582	-1.71625	3.28582	down	870.3607	695.8271	256.0357	246.4642	211.7667	CALML3	CLP	calmodulin	810	chr10	5556923
NM_0052(	-2.72715	-1.4474	2.727154	down	-2.76046	-1.46491	2.760464	down	-2.28634	-1.19304	2.286338	down	-2.31426	-1.21055	2.314263	down	-2.69883	-1.43233	2.698826	down	-2.73179	-1.44985	2.731789	down	415.5651	420.6408	152.3805	181.7602	153.98	COX6A2	COX6AH|(	cytochrome	1339	chr16	31346555
NM_00544	-4.45768	-2.15629	4.457676	down	-4.3009	-2.10464	4.300899	down	-5.42047	-2.43842	5.420472	down	-5.22983	-2.38676	5.229832	down	-2.47714	-1.30867	2.477136	down	-2.39001	-1.25702	2.390014	down	1257.384	1213.161	282.0716	231.9694	507.5958	EOMES	TBR2	eomesoder	8320	chr3	27732871
NM_00581	-5.61805	-2.49007	5.618046	down	-3.41066	-1.77005	3.410656	down	-5.23737	-2.38884	5.237372	down	-3.17955	-1.66882	3.179553	down	-4.91226	-2.29639	4.912258	down	-2.98218	-1.57637	2.982179	down	613.4968	372.4473	109.2011	117.1383	124.891	KLRG1	2F1|MAFA	killer cell l	10219	chr12	9033487
NM_00607	-2.24862	-1.16904	2.248617	down	-2.28158	-1.19003	2.281578	down	-2.3262	-1.21798	2.326201	down	-2.3603	-1.23897	2.360299	down	-2.21197	-1.14533	2.211967	down	-2.24439	-1.16632	2.244391	down	3323.517	3372.234	1478.027	1428.732	1502.517	HRBL	RABR	HIV-1 Rev	3268	chr7	99974817
NM_00643	-6.02191	-2.59022	6.021909	down	-6.13902	-2.61801	6.139023	down	-5.82529	-2.54233	5.825288	down	-5.93858	-2.57012	5.938578	down	-2.77144	-1.47064	2.771444	down	-2.82534	-1.49843	2.825343	down	618.0308	630.0502	102.6304	106.0945	222.9996	GNLY	519|D2S69	granulysin	10578	chr2	85774924
NM_00733	-3.40372	-1.76711	3.403722	down	-3.27758	-1.71263	3.277577	down	-3.89225	-1.9606	3.892251	down	-3.748	-1.90612	3.748001	down	-2.31447	-1.21068	2.314473	down	-2.2287	-1.1562	2.228697	down	486.7513	468.7119	143.0056	125.0565	210.3076	KLRD1	CD94	killer cell l	3824	chr12	10351683
NM_01231	-2.44613	-1.2905	2.446128	down	-2.60179	-1.3795	2.601788	down	-3.48539	-1.80132	3.485388	down	-3.70718	-1.89032	3.707181	down	-2.21903	-1.14993	2.219034	down	-2.36024	-1.23894	2.360243	down	1706.647	1815.25	697.6934	489.6577	769.0945	KIR2DS2	183ACTI|(	killer cell i	3807	chr19_rand	274137
NM_01231	-2.90397	-1.53802	2.903967	down	-2.07519	-1.05324	2.07519	down	-5.45725	-2.44817	5.457251	down	-3.89978	-1.96339	3.899781	down	-3.04303	-1.60551	3.043031	down	-2.17457	-1.12073	2.174566	down	1786.761	1276.829	615.283	327.4105	587.165	KIR2DS3	NKAT7	killer cell i	3808	chr19_rand	240823
NM_01231	-2.32922	-1.21985	2.32922	down	-2.54179	-1.34585	2.541794	down	-3.84616	-1.94342	3.846159	down	-4.19718	-2.06942	4.197176	down	-3.35632	-1.74688	3.356319	down	-3.66263	-1.87288	3.66263	down	2100.117	2291.783	901.6397	546.0296	625.7204	KIR2DS4	CD158I|KI	killer cell i	3809	chr19	60035985
NM_0133(	-2.24351	-1.16576	2.243512	down	-2.10469	-1.07361	2.104688	down	-2.53584	-1.34246	2.535836	down	-2.37892	-1.25031	2.378924	down	-2.25541	-1.17339	2.255409	down	-2.11585	-1.08124	2.115849	down	309.0532	289.9296	137.7542	121.8743	137.0276	C12orf24	HSU79274	chromoson	29902	chr12	1.09E+08
NM_0133(	-2.9202	-1.54607	2.920198	down	-2.43347	-1.28301	2.433467	down	-2.54885	-1.34985	2.548852	down	-2.12402	-1.08679	2.124016	down	-2.408	-1.26784	2.408004	down	-2.00664	-1.00478	2.006644	down	1449.144	1207.604	496.2485	568.5476	601.803	ZNF222	-	zinc finger	7673	chr19	49221355
NM_01414	-2.30617	-1.2055	2.306173	down	-2.40895	-1.2684	2.408948	down	-2.5349	-1.34193	2.534898	down	-2.64787	-1.40483	2.647866	down	-2.98653	-1.57847	2.986528	down	-3.11962	-1.64137	3.119623	down	2470.016	2580.093	1071.046	974.4046	827.0527	HSPC047	-	HSPC047 p	29060	chr7	1.02E+08
NM_0142(	-7.62743	-2.9312	7.627431	down	-4.2025	-2.07125	4.202504	down	-7.9135	-2.98432	7.9135	down	-4.36012	-2.12437	4.36012	down	-4.59714	-2.20074	4.597141	down	-2.5329	-1.34079	2.532898	down	3650.003	2011.051	478.5364	461.2376	793.9724	CD5	LEU1|T1	CD5 molec	921	chr11	60626542
NM_01421	-2.11946	-1.0837	2.119458	down	-2.27862	-1.18816	2.278616	down	-3.13163	-1.64691	3.131632	down	-3.3668	-1.75138	3.366798	down	-2.16848	-1.11668	2.168478	down	-2.33132	-1.22115	2.331318	down	1706.873	1835.048	805.3345	545.0426	787.1292	KIR2DL1	47.11|CD1	killer cell i	3802	chr19	59973091
NM_01429	-2.29253	-1.19694	2.29253	down	-2.25972	-1.17614	2.259721	down	-2.2854	-1.19244	2.285397	down	-2.25269	-1.17165	2.25269	down	-2.18585	-1.12819	2.185848	down	-2.15457	-1.1074	2.154566	down	1305.651	1286.966	569.5241	571.3016	597.3202	NPTXR	NPR	neuronal pe	23467	chr22	37544402
NM_01438	-2.60674	-1.38225	2.606744	down	-2.05566	-1.0396	2.055662	down	-2.67008	-1.41688	2.670079	down	-2.10561	-1.07424	2.105608	down	-3.79984	-1.92594	3.799842	down	-2.99653	-1.58329	2.996532	down	2261.698	1783.561	867.6332	847.0527	595.2083	ZBTB32	FAXF|FAZ	zinc finger	27033	chr19	40895669
NM_01451	-2.71457	-1.44072	2.714571	down	-2.30943	-1.20754	2.30943	down	-5.52655	-2.46638	5.526554	down	-4.70173	-2.23319	4.701733	down	-4.12745	-2.04525	4.127445	down	-3.51144	-1.81206	3.511437	down	2004.827	1705.613	738.5428	362.7626	485.7308	KIR2DL3	CD158B2|(	killer cell i	3804	chr19	59941785
NM_01451	-3.4073	-1.76863	3.407302	down	-2.36296	-1.2406	2.362961	down	-6.03001	-2.59216	6.030013	down	-4.18181	-2.06413	4.181809	down	-4.11006	-2.03916	4.110058	down	-2.85032	-1.51112	2.850321	down	2289.074	1587.471	671.8141	379.6134	556.9443	KIR2DS1	CD158H|E	killer cell i	3806	chr19_rand	257787
NM_01462	-2.3773	-1.24932	2.377298	down	-2.42762	-1.27954	2.427622	down	-3.27808	-1.71285	3.278079	down	-3.34747	-1.74307	3.347471	down	-3.01671	-1.59298	3.016711	down	-3.08057	-1.6232	3.080571	down	373.9487	381.8647	157.2999	114.0756	123.9591	HOXC4	HOX3|HO	homeobox	3221	chr12	52696939
NM_01523	-2.12133	-1.08497	2.121333	down	-2.11082	-1.0778	2.110815	down	-2.70885	-1.43768	2.708849	down	-2.69542	-1.43051	2.695418	down	-2.55951	-1.35587	2.559508	down	-2.54682	-1.3487	2.546818	down	308.4501	306.9208	145.4039	113.8676	120.5115	IQSEC3	KIAA1110	IQ motif ar	440073	chr12	56802

NM_01541	-2.12958	-1.09057	2.129575	down	-2.147	-1.10233	2.147005	down	-2.26518	-1.17963	2.26518	down	-2.28372	-1.19139	2.283719	down	-2.38121	-1.25169	2.381205	down	-2.40069	-1.26345	2.400694	down	502.684	506.7982	236.0489	221.9179	211.1049	C20orf28	DKFZP434	chromoson	25876	chr20	3706150
NM_01554	-2.34156	-1.22747	2.341562	down	-2.38093	-1.25153	2.380933	down	-3.07195	-1.61916	3.071951	down	-3.1236	-1.64321	3.123603	down	-2.84638	-1.50913	2.846379	down	-2.89424	-1.53318	2.894237	down	639.7636	650.5204	273.2208	208.2596	224.764	ACOT11	BFIT|BFIT	acyl-CoA t	26027	chr1	54786488
NM_01673	-2.9655	-1.56828	2.965502	down	-2.01628	-1.0117	2.016284	down	-4.02826	-2.01016	4.028264	down	-2.73887	-1.45358	2.738869	down	-3.407	-1.7685	3.407	down	-2.31646	-1.21192	2.316464	down	504.0425	342.7051	169.9687	125.1265	147.9432	PAX5	BSAP	paired box	5079	chr9	36828530
NM_01859	-4.44697	-2.15282	4.446972	down	-2.53747	-1.34339	2.537471	down	-6.99485	-2.80629	6.994845	down	-3.9913	-1.99686	3.991304	down	-4.32221	-2.11177	4.322209	down	-2.46628	-1.30234	2.46628	down	1167.073	665.9392	262.4421	166.8476	270.0177	SLC16A10	PRO0813|	solute carri	117247	chr6	1.12E+08
NM_01906	-2.42952	-1.28067	2.42952	down	-2.40832	-1.26802	2.408316	down	-2.98296	-1.57675	2.982965	down	-2.95693	-1.5641	2.95693	down	-2.2139	-1.14659	2.213903	down	-2.19458	-1.13395	2.194581	down	1060.482	1051.227	436.4987	355.5128	479.0102	SDK2	FLJ10832|	sidekick hc	54549	chr17	68845406
NM_02117	-2.27426	-1.1854	2.274264	down	-2.42557	-1.27832	2.425569	down	-2.15622	-1.1085	2.15622	down	-2.29967	-1.20143	2.299671	down	-2.19937	-1.13709	2.199371	down	-2.34569	-1.23001	2.345692	down	2261.36	2411.806	994.3258	1048.761	1028.185	HAMP	HEPC|HFE	hepcidin ar	57817	chr19	40465249
NM_02193	-2.42164	-1.27598	2.421637	down	-2.32235	-1.21558	2.322347	down	-2.3355	-1.22373	2.335499	down	-2.23974	-1.16333	2.239741	down	-2.71867	-1.4429	2.718671	down	-2.6072	-1.3825	2.607202	down	862.8722	827.4935	356.3177	369.4594	317.3875	PAPPA2	PAPP-A2|	pappalysin	60676	chr1	1.75E+08
NM_03095	-2.21941	-1.15018	2.219414	down	-2.63131	-1.39578	2.631311	down	-3.35448	-1.74609	3.354475	down	-3.97703	-1.99169	3.977025	down	-2.60773	-1.38279	2.607729	down	-3.09169	-1.6284	3.091692	down	449.3061	532.6919	202.4436	133.9423	172.2979	ADAMTS1	ADAM-TS	ADAM me	81794	chr19	8551125
NM_03195	-4.83029	-2.27211	4.830294	down	-4.98916	-2.3188	4.989164	down	-9.83235	-3.29754	9.832348	down	-10.1557	-3.34422	10.15574	down	-3.02997	-1.5993	3.02997	down	-3.12963	-1.64599	3.129626	down	5363.468	5539.874	1110.381	545.4921	1770.139	KSP37	-	Ksp37 prot	83888	chr4	15570963
NM_03221	-2.10301	-1.07245	2.103008	down	-2.4922	-1.31742	2.492202	down	-2.9012	-1.53665	2.901204	down	-3.43812	-1.78162	3.438116	down	-2.7835	-1.4769	2.783502	down	-3.29863	-1.72187	3.298631	down	2996.053	3550.518	1424.651	1032.693	1076.361	SLA2	C20orf156	Src-like-ad	84174	chr20	34674335
NM_03312	-2.25738	-1.17465	2.257376	down	-2.07746	-1.05482	2.077456	down	-2.68768	-1.42636	2.687683	down	-2.47347	-1.30653	2.473467	down	-3.17952	-1.66881	3.179518	down	-2.9261	-1.54898	2.926101	down	737.7606	678.9591	326.8223	274.4969	232.0354	SCRT2	-	scratch hor	85508	chr20	590239
NM_05328	-3.30053	-1.7227	3.300533	down	-2.50813	-1.32661	2.508126	down	-2.96184	-1.56649	2.961838	down	-2.25075	-1.1704	2.250746	down	-2.99302	-1.5816	2.993017	down	-2.27444	-1.18551	2.274439	down	648.2753	492.6343	196.4153	218.876	216.596	ODF3	SHIPPO1|	outer dense	113746	chr11	186760
NM_05824	-2.17617	-1.12179	2.176165	down	-2.82097	-1.49619	2.820969	down	-2.73761	-1.45292	2.73761	down	-3.54877	-1.82732	3.548773	down	-2.61498	-1.3868	2.614978	down	-3.3898	-1.7612	3.389804	down	2573.203	3335.651	1182.448	939.945	984.0247	KRT6C	CK6C|K6C	keratin 6C	140446	chr12	51167243
NM_13345	-2.15468	-1.10747	2.15468	down	-2.19835	-1.13642	2.198352	down	-2.30775	-1.20648	2.307745	down	-2.35452	-1.23543	2.354519	down	-2.52268	-1.33496	2.522683	down	-2.57381	-1.36391	2.573814	down	2357.346	2405.126	1094.059	1021.493	934.4597	EMID1	EMI5|EMU	EMI domai	129080	chr22	27931952
NM_13841	-2.71525	-1.44109	2.715251	down	-3.00317	-1.58648	3.003167	down	-3.78884	-1.92175	3.788835	down	-4.19059	-2.06715	4.19059	down	-2.17034	-1.11792	2.17034	down	-2.40048	-1.26332	2.400475	down	786.2566	869.6285	289.5705	207.5194	362.2735	C10orf65	FLJ37472	chromoson	112817	chr10	99334107
NM_13916	-2.07113	-1.05042	2.07113	down	-2.34791	-1.23138	2.347909	down	-3.34032	-1.73999	3.340319	down	-3.78671	-1.92094	3.786709	down	-2.43279	-1.28261	2.432795	down	-2.75791	-1.46357	2.757906	down	634.874	719.7167	306.5352	190.0639	260.9649	TRUB1	PUS4	TruB pseuc	142940	chr10	1.17E+08
NM_14719	-2.61011	-1.38411	2.610106	down	-2.43978	-1.28675	2.439776	down	-3.58502	-1.84198	3.585018	down	-3.35107	-1.74462	3.351068	down	-2.62155	-1.39042	2.62155	down	-2.45047	-1.29306	2.450473	down	785.127	733.8913	300.8027	219.0022	299.4896	TMIE	DFNB6	transmemb	259236	chr3	46717826
NM_14896	-6.26856	-2.64813	6.268561	down	-3.40161	-1.76622	3.401611	down	-3.95929	-1.98524	3.959291	down	-2.14849	-1.10333	2.148494	down	-3.99133	-1.99687	3.991329	down	-2.16588	-1.11495	2.16588	down	2895.161	1571.048	461.8542	731.2322	725.3627	TNFRSF25	APO-3|DD	tumor necr	8718	chr1	6443797
NM_15237	-2.40262	-1.26461	2.402623	down	-3.09286	-1.62894	3.092862	down	-2.1432	-1.09977	2.143202	down	-2.75891	-1.4641	2.758913	down	-2.47129	-1.30526	2.47129	down	-3.18126	-1.6696	3.181257	down	422.4713	543.8413	175.8376	197.1216	170.9517	KLHDC7A	FLJ38753|	kelch doma	127707	chr1	18680452
NM_15327	-3.18917	-1.67318	3.189173	down	-2.79522	-1.48296	2.795216	down	-2.37894	-1.25032	2.378936	down	-2.08507	-1.06009	2.085067	down	-2.73764	-1.45293	2.737643	down	-2.39946	-1.26271	2.399463	down	1504.348	1318.517	471.7048	632.3618	549.505	RP11-450F	MGC1256	hypothetica	257240	chrX	21583529
NM_15327	-4.48727	-2.16584	4.487271	down	-3.04427	-1.6061	3.044268	down	-3.23554	-1.69401	3.235539	down	-2.19506	-1.13426	2.195064	down	-3.49436	-1.80503	3.494364	down	-2.37066	-1.24529	2.370657	down	1596.226	1082.917	355.7232	493.3417	456.8003	VMD2L2	MGC1268	vitelliform	266675	chr1	45021845
NM_15360	-2.08346	-1.05898	2.083458	down	-2.22303	-1.15253	2.223026	down	-2.43279	-1.28261	2.432785	down	-2.59575	-1.37615	2.595755	down	-2.23732	-1.16177	2.237322	down	-2.3872	-1.25532	2.387197	down	791.2333	844.237	379.7693	325.2376	353.652	MYOCD	MYCD	myocardin	93649	chr17	12510230
NM_15370	-2.00747	-1.00538	2.007469	down	-3.40221	-1.76647	3.402209	down	-2.03574	-1.02555	2.035736	down	-3.45012	-1.78664	3.450115	down	-2.10854	-1.07624	2.108539	down	-3.5735	-1.83734	3.573499	down	300.8586	509.8877	149.8696	147.7886	142.6858	RTP1	MGC3545	receptor tra	132112	chr3	1.88E+08
NM_17208	-2.43712	-1.28517	2.437116	down	-2.31076	-1.20837	2.310762	down	-2.19132	-1.1318	2.191318	down	-2.07771	-1.05499	2.077708	down	-2.88747	-1.52981	2.88747	down	-2.73777	-1.453	2.737768	down	620.2306	588.0744	254.4937	283.04	214.8007	CAMK2B	CAM2|CA	calcium/ca	816	chr7	44225415
NM_17210	-12.7067	-3.66752	12.70671	down	-4.64383	-2.21532	4.643832	down	-23.4509	-4.55157	23.45086	down	-8.57042	-3.09937	8.570421	down	-7.07824	-2.82339	7.078241	down	-2.58684	-1.37119	2.586835	down	3610.962	1319.673	284.1776	153.98	510.1497	CD8B	CD8B1|LY	CD8b mole	926	chr2	86895970
NM_17210	-16.7673	-4.06758	16.76727	down	-6.36995	-2.67128	6.369953	down	-13.0408	-3.70496	13.04076	down	-4.95424	-2.30866	4.954239	down	-6.7835	-2.76203	6.783504	down	-2.57708	-1.36574	2.577081	down	4202.895	1596.697	250.6607	322.2891	619.5758	CD8B	CD8B1|LY	CD8b mole	926	chr2	86896002
NM_17368	-9.51825	-3.2507	9.518249	down	-2.53251	-1.34057	2.532508	down	-12.5777	-3.6528	12.5777	down	-3.34653	-1.74267	3.346531	down	-8.74843	-3.12902	8.748432	down	-2.32768	-1.2189	2.327684	down	1376.339	366.2007	144.6001	109.427	157.3241	ATG9B	APG9L2|N	ATG9 auto	285973	chr7	1.5E+08
NM_17379	-3.13878	-1.6502	3.138778	down	-3.10573	-1.63493	3.105726	down	-2.05662	-1.04027	2.056618	down	-2.03496	-1.025	2.034962	down	-2.40295	-1.2648	2.402946	down	-2.37764	-1.24953	2.377642	down	782.8948	774.6509	249.4267	380.6711	325.8063	VSIG9	DKFZp667	V-set and i	201633	chr3	1.15E+08
NM_17816	-2.36658	-1.24281	2.366584	down	-2.30336	-1.20374	2.303355	down	-2.11103	-1.07795	2.111033	down	-2.05463	-1.03888	2.054632	down	-2.68468	-1.42475	2.68468	down	-2.61295	-1.38568	2.612952	down	428.7724	417.3167	181.1778	203.1102	159.7108	OTOP2	-	otopetrin 2	92736	chr17	70431964
NM_18251	-2.78381	-1.47706	2.783808	down	-2.25768	-1.17484	2.257684	down	-2.66139	-1.41218	2.661394	down	-2.15841	-1.10997	2.158405	down	-4.1911	-2.06733	4.1911	down	-3.399	-1.76511	3.399005	down	567.3245	460.1032	203.7944	213.1681	135.3641	FLJ32252	-	hypothetica	146336	chr16	1054093
NM_18262	-2.56659	-1.35985	2.566588	down	-2.01712	-1.0123	2.01712	down	-3.50351	-1.8088	3.503509	down	-2.75346	-1.46125	2.75346	down	-2.76188	-1.46565	2.761878	down	-2.1706	-1.11809	2.170601	down	1259.605	989.9422	490.7702	359.5267	456.0683	MGC5249	PRO7171|	hypothetica	348378	chr1	52871653
NM_18263	-2.47224	-1.30582	2.472243	down	-2.63948	-1.40025	2.639476	down	-2.26578	-1.18001	2.26578	down	-2.41905	-1.27444	2.419047	down	-2.56969	-1.3616	2.569691	down	-2.74352	-1.45603	2.743516	down	534.835	571.0135	216.336	236.0489	208.132	SLC6A18	FLJ31236|	solute carri	348932	chr5	1278469
NM_19443	-2.90679	-1.53943	2.906791	down	-4.14164	-2.0502	4.141639	down	-3.83712	-1.94002	3.837116	down	-5.46718	-2.4508	5.467181	down	-3.68949	-1.88342	3.689486	down	-5.25684	-2.39419	5.256836	down	478.6788	682.0287	164.676	124.7496	129.7413	LOC28549	MGC1202	hypothetica	285498	chr4	1055268
NM_19807	-2.9212	-1.54656	2.921203	down	-2.28141	-1.18993	2.281415	down	-2.83104	-1.50133	2.831042	down	-2.211	-1.1447	2.211	down	-2.97461	-1.5727	2.974607	down	-2.32312	-1.21606	2.323122	down	340.3131	265.7793	116.4976	120.2077	114.4061	OR2C3	OR2C4|OF	olfactory re	81472	chr1	2.46E+08
NM_19848	-3.60345	-1.84938	3.603452	down	-3.99087	-1.9967	3.99087	down	-2.86676	-1.51942	2.866761	down	-3.17498	-1.66675	3.174975	down	-2.48218	-1.31161	2.482176	down	-2.74904	-1.45893	2.749042	down	1230.02	1362.263	341.345	429.0627	495.5411	LOC28494	-	similar to E	284948	chr2	85515428
NM_19849	-2.11457	-1.08037	2.114571	down	-2.52547	-1.33655	2.52547	down	-2.40972	-1.26886	2.409717	down	-2.87797	-1.52505	2.877967	down	-2.18381	-1.12685	2.18381	down	-2.60816	-1.38303	2.608162	down	1590.295	1899.317	752.0648	659.9509	728.2203	CLEC4G	LP2698|LS	C-type lect	339390	chr19	7699849
NM_19857	-9.81258	-3.29463	9.812576	down	-3.17512	-1.66681	3.175124	down	-6.49024	-2.69827	6.49024	down	-2.10009	-1.07045	2.100092	down	-8.58143	-3.10122	8.58143	down	-2.77675	-1.4734	2.776754	down	5696.005	1843.096	580.4801	877.6263	663.7593	UNQ470	-	GAAI470	375704	chr9	34511039
NM_19917	-2.7228	-1.44509	2.722799	down	-2.2938	-1.19774	2.293802	down	-3.14031	-1.65091	3.140311	down	-2.64553	-1.40356	2.645532	down	-2.46171	-1.29966	2.46171	down	-2.07385	-1.05231	2.073849	down	2325.283	1958.917	854.0046	740.4625	944.5803	BGLAP	BGP|PMF1	bone gamm	632	chr1	1.54E+08
NM_19924	-3.5808	-1.84028	3.580801	down	-2.52147	-1.33427	2.521471	down	-4.4922	-2.16742	4.492203	down	-3.16325	-1.66141	3.163248	down	-3.57957	-1.83979	3.579567	down	-2.5206	-1.33377	2.520602	down	826.1827	581.7681	230.7257	183.9148	230.8052	GPR150	PGR11	G protein-c	285601	chr5	94981735
NM_20163	-15.5828	-3.96189	15.58283	down	-5.55081	-2.4727	5.550806	down	-30.3645	-4.92432	30.36454	down	-10.8162	-3.43513	10.81624	down	-8.92242	-3.15744	8.922422	down	-3.17828	-1.66825	3.178282	down	14506.31	5167.336	930.9164	477.7387	1625.827	TCF7	MGC4773	transcriptic	6932	chr5	1.33E+08
NM_20576	-2.55059	-1.35083	2.550585	down	-3.13509	-1.64851	3.135093	down	-2.43654	-1.28483	2.43654	down	-2.99491	-1.58251	2.994912	down	-2.89772	-1.53492	2.897723	down	-3.56178	-1.8326	3.561783	down	756.8486	930.2927	296.7353	310.6243	261.1874	P117	-	hypothetica	125988	chr19	5629432
NM_20741	-2.21577	-1.14781	2.215766	down	-2.1743	-1.12055	2.174302	down	-3.69683	-1.88629	3.696826	down	-3.62765	-1.85903	3.627647	down	-3.20984	-1.6825	3.209838	down	-3.14977	-1.65525	3.149772	down	756.3405	742.187	341.345	204.5918	235.632	FLJ43860	-	FLJ43860 |	389690	chr8	1.43E+08
NM_20749	-3.11741	-1.64035	3.117411	down	-2.6376	-1.39923	2.637599	down	-3.20973	-1.68245	3.209731	down	-2.71571	-1.44133	2.715709	down	-3.2466	-1.69893	3.246605	down	-2.74691	-1.45781	2.746908	down	1391.962	1177.72	446.5123	433.6695	428.744	FLJ41841	-	FLJ41841 |	401263	chr6	47975303
U33328	-2.00264	-1.0019	2.002641	down	-2.61095	-1.38457	2.610948	down	-3.17368	-1.66615	3.173676	down	-4.13769	-2.04883	4.137689	down	-2.13374	-1.09338	2.133736	down	-2.78186	-1.47605	2.781865	down	1495.004	1949.116	746.5164	471.0639	700.6508	KIR3DL1	AMB11|CI	killer cell i	3811	chr19	59989604
U66145	-6.8314	-2.77218	6.831401	down	-4.92542	-2.30025	4.925422	down	-10.87	-3.44228	10.87003	down	-7.83727	-2.97035	7.837265	down	-2.84203	-1.50692	2.842026	down	-2.04909	-1.03499	2.049093	down	8059.117	5810.602	1179.717	741.4068	2835.694	CD6	TP120	CD6 molec	923	chr11	60495913
U73394	-2.58091	-1.36788	2.580905	down	-2.13369	-1.09335	2.133691	down	-7.51584	-2.90993	7.515841	down	-6.21351	-2.63541	6.213512	down	-2.45172	-1.29379	2.451719	down	-2.02689	-1.01927	2.02689	down	928.7602	767.8266	359.8583	123.5737	378.82	KIR2DL4	103AS|15.2	killer cell i	3805	chr19	60006906
XM_05934	-14.1965	-3.82746	14.19645	down	-3.18627	-1.67187	3.186267	down	-15.0225	-3.90905	15.02247	down	-3.37166	-1.75346	3.371661	down	-10.4573	-3.38644	10.45727	down	-2.34704	-1.23084	2.347043	down	2415.139	542.0565	170.1227	160.7684	230.953	LOC12929	FLJ46467	hypothetica	129293	chr2	84902290
XM_08467	-2.31535	-1.21123	2.315352	down	-2.67413	-1.41907	2.674133	down	-2.14802	-1.103	2.148016	down	-2.48087	-1.31084	2.480867	down	-2.65305	-1.40765	2.653048	down	-3.06416	-1.61549	3.064158	down	915.0729	1056.87	395.2199	426.0085	344.9138	LOC14394	-	similar to C	143941	chr11	1.18E+08
XM_08938	-11.6587	-3.54334	11.65871	down	-2.01941	-1.01393	2.01941	down	-12.4515	-3.63824	12.45145	down	-2.15672	-1.10884	2.156722	down	-12.6978	-3.66651	12.6978	down	-2.19939	-1.13711	2.199392	down	2206.273	382.1495	189.2382	177.1899	173.7523	TTC24	-	tetratricope	164118	chr1	1.55E+08
XM_17185	-3.01438	-1.59186	3.014378	down	-3.13171	-1.64695	3.131711	down	-2.27862	-1.18816	2.278625	down	-2.36732	-1.24325	2.367319	down	-2.59006	-1.37298	2.590056	down	-2.69087	-1.42807	2.690873	down	488.8124	507.8392	162.1603	214.5208	188.7265	LOC25527	-	similar to n	255275	chr17	77490227
XM_29520	-2.72693	-1.44728	2.726926	down	-2.63053	-1.39536	2.630534	down	-2.56464	-1.35876	2.564645	down	-2.47399	-1.30684	2.473989	down	-2.10809	-1.07594	2.108093	down	-2.03358	-1.02402	2.033575	down	435.6244	420.2259	159.7493	169.8576	206.6439	LOC34028	-	hypothetica	340286	chr7	38691474
XM_37377	-3.81408	-1.93134	3.814082	down	-2.1568	-1.10889	2.156798	down	-4.74821	-2.24739	4.748214	down	-2.68503	-1.42494	2.685034	down	-5.04343	-2.3344	5.043426	down	-2.85197	-1.51196	2.85197	down	697.8931	394.6466	182.978	146.9801	138.3768	TTMA	-	hypothetica	388459	chr18	5619965
XM_92606	-3.05183	-1.60967	3.051828	down	-2.79297	-1.4818	2.792966	down	-2.24068	-1.16394	2.240683	down	-2.05062	-1.03606	2.050624	down	-2.32827	-1.21926	2.32827	down	-2.13078	-1.09138	2.130782	down	903.8745	827.2063	296.1748	403.3924	388.2173	LOC64258	-	similar to e	642584	chr8	1.45E+08
XM_92640	-2.84708	-1.50948	2.84708	down	-2.25577	-1.17362	2.255767	down	-3.15837	-1.65918	3.158372	down	-2.50241	-1.32332	2.502406	down	-3.42503	-1.77612	3.425034	down	-2.71369	-1.44025	2.713685	down	530.3814	420.2259	186.2896	167.9287	154.8543	LOC64304	-	hypothetica	643040	chr4	1.79E+08
XM_92662	-2.53337	-1.34106	2.533367	down	-2.23965	-1.16327	2.239651	down	-2.64059	-1.40086	2.640586	down	-2.33444	-1.22308	2.334439	down	-2.91891	-1.54543	2.918911	down	-2.5805	-1.36765	2.580496	down	2135.301	1887.736	842.8708	808.6467	731.5402	LOC64326	-	hypothetica	643266	chr22	47206799
XM_92682	-2.5049	-1.32475	2.504901	down	-2.12745	-1.08913	2.127453	down	-2.68925	-1.4272	2.689251	down	-2.28402	-1.19158	2.284024	down	-3.2307	-1.69184	3.230696	down	-2.74388	-1.45622	2.743882	down	2088.152	1773.502	833.6267	776.4812	646.3476	LOC64350	-	similar to C	643501	chr17	6479625
XM_92715	-2.04922	-1.03507	2.049216	down	-2.58658	-1.37104	2.586577	down	-2.11942	-1.08367	2.119417	down	-2.67519	-1.41964	2.675187	down	-2.39395	-1.25939	2.393948	down	-3.02171	-1.59536	3.021707	down	1471.356	1857.186	718.0093	694.2269	614.6151	LOC65314	-	similar to c	653148	chr15	88256458
XM_92734	-2.21673	-1.14844	2.216735	down	-2.2345	-1.15995	2.234495	down	-2.29642	-1.19939	2.296419	down	-2.31482	-1.2109	2.314818	down	-2.18694	-1.12891	2.18694	down	-2.20446	-1.14043	2.204462	down	250.1993	252.2038	112.8684	108.9519	114.4061	LOC64413	-	hypothetica	644136	chr1	1847365
XM_92785	-2.06943	-1.04923	2.069425	down	-2.91781	-1.54489	2.91781	down	-2.06722	-1.04769	2.067215	down	-2.91469	-1.54334	2.914694	down	-2.03731	-1.02666	2.037307	down	-2.87252	-1.52232	2.872525	down	510.2782	719.4727	246.5797	246.8433	250.467	FLJ36951	-	similar to e	253314	chr5	1.76E+08
XM_92808	-2.34833	-1.23164	2.348335	down	-2.36404	-1.24125	2.364039	down	-2.07254	-1.0514	2.072539	down	-2.0864	-1.06102	2.086399	down	-2.30087	-1.20218	2.300869	down	-2.31626	-1.21179	2.316256	down	1296.087	1304.755	551.9175	625.3619	563.3032	LOC64502	-	hypothetica	645022	chr15	39973725
XM_92824	-2.03681	-1.02631	2.036811	down	-2.05227	-1.03722	2.052269	down	-2.24886	-1.16919	2.248859	down	-2.26593	-1.1801	2.265927	down	-2.25147	-1.17087	2.251467	down	-2.26855	-1.18177	2.268554	down	738.0948	743.6966	362.3777	328.2086	327.8284	LOC64520	-	hypothetica	645205	chr22	18757988
XM_92833	-3.32887	-1.73503	3.328873	down	-2.79174	-1.48117	2.791745	down	-2.7795	-1.47483	2.779503	down	-2.33102	-1.22096	2.331017	down	-2.71512	-1.44102	2.715122	down	-2.27702	-1.18715	2.277025	down	1410.985	1183.316	423.8627	507.6395	519.6765	FAM20C	-	family witl	56975	chr7	288057
XM_92864	-3.91122	-1.96762	3.911221	down	-3.46615	-1.79333	3.46615	down	-3.15391	-1.65714	3.153915	down	-2.79502	-1.48286	2.79502	down	-3.77723	-1.91733	3.777228	down	-3.3474	-1.74304	3.347405	down	1557.114	1379.925	398.1147	493.7084	412.2373	LOC65365	-	similar to N	653651	chr10	75240456
XM_92878	-2.97574	-1.57325	2.975739	down	-2.21574	-1.14779	2.215739	down	-2.86285	-1.51745	2.862852	down	-2.13168	-1.09199	2.131684	down	-3.99053	-1.99658	3.990532	down	-2.97136	-1.57112	2.971355	down	1350.585	1005.647	453.8653	471.7619	338.4473	LOC65366	-	similar to C	653668	chr7	5887046
XM_92889	-2.57284	-1.36336	2.572842	down	-3.23449	-1.69354	3.23449	down	-3.51614	-1.81399	3.516139	down	-4.42037	-2.14417	4.420371	down	-2.59187	-1.37399	2.591869	down	-3.25841	-1.70417	3.258411	down	418.5295	526.1612	162.6721	119.031	161.4779	LOC64592	-	similar to t	645929	chr8	68036083
XM_92894	-2.62814	-1.39404	2.628142	down	-2.10769	-1.07566	2.107693	down	-3.81329	-1.93104	3.813292	down	-3.05815	-1.61266	3.058149	down	-2.71022	-1.43841	2.710216	down	-2.17351	-1.12003	2.173514	down	1770.918	1420.224	673.8288	464.4065	653.423	LOC64597	-	similar to c	645977	chr1	1.52E+08
XM_92916	-2.19334	-1.13313	2.193339	down	-2.1037	-1.07293	2.103697	down	-2.86581	-1.51894	2.865807	down	-2.74868	-1.45874	2.748682	down	-2.34946	-1.23233	2.349464	down	-2.25344	-1.17213	2.253441	down	507.3758	486.6393	231.3258	177.0447	215.9539	LOC64621	-	similar to s	646213	chr10	35204065
XM_92961	-3.35237	-1.74518	3.352372	down	-3.02518	-1.59702	3.025182	down	-2.43761	-1.28547	2.437607	down	-2.1997	-1.13731	2.199698	down	-2.70665	-1.43651	2.706647	down	-2.44248	-1.28835	2.44248	down	547.4598	494.0281	163.3052	224.5891	202.2649	LOC64667	-	hypothetica	646673	chr4	1.51E+08
XM_93012	-2.30638	-1.20563	2.306376	down	-2.03614	-1.02584	2.036143	down	-2.50611	-1.32545	2.506114	down	-2.21248	-1.14566	2.212478	down	-2.52237	-1.33478	2.522373	down	-2.22683	-1.15499	2.226833	down	746.895	659.3831	323.8393	298.0292	296.1081	LOC64710	-	similar to E	647102	chr7	45397597
XM_93038	-3.31954	-1.73098	3.319536	down	-3.27748	-1.71259	3.277479	down	-3.22364	-1.68869	3.223641	down	-3.1828	-1.6703	3.182798	down	-2.18058	-1.12471	2.180578	down	-2.15295	-1.10632	2.152951	down	554.3494	547.3259	166.996	171.9637	254.2213	LOC64731	-	hypothetica	647311	chr14	1.05E+08
XM_93066	-2.81508	-1.49318	2.815083	down	-2.88525	-1.52869	2.885246	down	-2.47644	-1.30827	2.476443	down	-2.53817	-1.34379	2.538166	down	-2.97178	-1.57133	2.971776	down	-3.04584	-1.60684	3.045844	down	1938.795	1987.117	688.7167	782.8948	652.4027	LOC64226	-	hypothetica	642268	chr2	1.12E+08
XM_93077	-2.28412	-1.19164	2.284119	down	-2.63637	-1.39855	2.636367	down	-2.47428	-1.30701	2.474282	down	-2.85586	-1.51392	2.855855	down	-2.0713	-1.05053	2.071297	down	-2.39072	-1.25745	2.390724	down	755.497	872.0065	330.7607	305.3399	364.7459	LOC65304	-	similar to p	653045	chr10	48983044
XM_93083	-3.63753	-1.86296	3.637527	down	-2.06156	-1.04373	2.061557	down	-4.68349	-2.22758	4.683491	down	-2.65435	-1.40836	2.654354	down	-3.76725	-1.91351	3.76725	down	-2.13508	-1.09429	2.135078	down	609.3818	345.3653	167.5264	130.1127	161.7577	FLJ12334	FLJ40608	hypothetica	400946	chr2	19932121
XM_93145	-3.96505	-1.98734	3.965053	down	-4.76092	-2.25124	4.760918	down	-2.71764	-1.44236	2.717643	down	-3.26313	-1.70626	3.263129	down	-2.30576	-1.20524	2.305761	down	-2.76857	-1.46914	2.768574	down	3365.302	4040.786	848.7408	1238.316	1459.519	LOC64327	-	hypothetica	643278	chr17	71629099
XM_93150	-2.33127	-1.22112	2.331275	down	-2.24068	-1.16394	2.240683	down	-2.37116	-1.24559	2.37116	down	-2.27902	-1.18841	2.279019	down	-2.20289	-1.1394	2.202893	down	-2.11729	-1.08222	2.11729	down	593.4753	570.4133	254.5712	250.289	269.4072	LOC64337	-	hypothetica	643377	chr2	1.65E+08
XM_93172	-2.0901	-1.06357	2.090099	down	-2.19989	-1.13743	2.199887	down	-2.37836	-1.24997	2.378364	down	-2.50329	-1.32383	2.503294	down	-2.09775	-1.06885	2.097754	down	-2.20794	-1.1427	2.207945	down	3056.899	3217.471	1462.562	1285.295	1457.225	KIAA0415	-	KIAA0415	9907	chr7	4781825
XM_93220	-4.70753	-2.23497	4.70753	down	-2.91988	-1.54591	2.919882	down	-3.34861	-1.74356	3.348611	down	-2.077	-1.0545	2.077002	down	-4.30155	-2.10486	4.301554	down	-2.66807	-1.4158	2.668072	down	585.3154	363.0464	124.336	174.7935	136.0707	LOC64447	-	hypothetica	644472	chr11	63290928

XM_93227	-2.1246	-1.08719	2.124599	down	-2.28349	-1.19124	2.28349	down	-2.23315	-1.15908	2.233152	down	-2.40016	-1.26313	2.400161	down	-2.07863	-1.05563	2.078631	down	-2.23408	-1.15968	2.234084	down	34935.43	37548.12	16443.31	15644	16806.95	LOC64463	-	hypothetica	644635	chr12	54687343
XM_93254	-3.15358	-1.65699	3.15358	down	-2.45269	-1.29437	2.452693	down	-2.84256	-1.50719	2.842555	down	-2.21079	-1.14456	2.210793	down	-2.80899	-1.49005	2.80899	down	-2.18469	-1.12743	2.184688	down	439.2017	341.5885	139.2708	154.5095	156.3558	LOC65324	-	similar to p	653247	chr12	11435747
XM_93255	-2.75097	-1.45994	2.750975	down	-2.2287	-1.1562	2.2287	down	-2.74143	-1.45493	2.741427	down	-2.22096	-1.15119	2.220965	down	-2.99251	-1.58136	2.992509	down	-2.42438	-1.27762	2.424379	down	507.9933	411.5503	184.6594	185.3025	169.755	LOC64497	-	hypothetica	644978	chr16	65471762
XM_93258	-5.65355	-2.49916	5.65355	down	-3.10746	-1.63574	3.107463	down	-4.50089	-2.17021	4.500889	down	-2.47391	-1.30679	2.473905	down	-4.28369	-2.09886	4.283693	down	-2.35452	-1.23544	2.354524	down	1530.203	841.0731	270.6623	339.9779	357.2157	LOC64502	-	hypothetica	645023	chr17	18205967
XM_93282	-2.13332	-1.0931	2.133324	down	-2.31412	-1.21046	2.314119	down	-2.534	-1.34141	2.533996	down	-2.74875	-1.45877	2.748748	down	-2.22661	-1.15485	2.226613	down	-2.41531	-1.27221	2.415314	down	316.864	343.7177	148.5307	125.0452	142.3076	FBF1	FBF-1|FLJ	Fas (TNFR	85302	chr17	71433523
XM_93283	-2.75469	-1.46189	2.754685	down	-3.24688	-1.69905	3.246882	down	-2.67509	-1.41959	2.675091	down	-3.15307	-1.65676	3.153066	down	-2.52221	-1.33469	2.52221	down	-2.97287	-1.57186	2.972868	down	1391.962	1640.673	505.3073	520.342	551.8821	LOC64534	-	hypothetica	645346	chr16	69029991
XM_93285	-2.27668	-1.18693	2.27668	down	-2.42878	-1.28023	2.428778	down	-2.2188	-1.14978	2.218797	down	-2.36703	-1.24308	2.367027	down	-2.84234	-1.50708	2.842342	down	-3.03223	-1.60038	3.03223	down	334.8268	357.1954	147.068	150.9047	117.7996	LOC64536	-	hypothetica	645365	chr20	56186973
XM_93288	-3.61272	-1.85308	3.612718	down	-2.48047	-1.31061	2.48047	down	-4.93013	-2.30163	4.930132	down	-3.385	-1.75916	3.384999	down	-3.35067	-1.74445	3.350668	down	-2.30055	-1.20198	2.300548	down	727.8488	499.7366	201.4685	147.6327	217.225	LOC64541	-	hypothetica	645411	chr18	75651000
XM_93297	-3.24373	-1.69766	3.243734	down	-3.10246	-1.63341	3.102464	down	-2.87504	-1.52358	2.875037	down	-2.74982	-1.45934	2.749824	down	-2.50003	-1.32194	2.500026	down	-2.39115	-1.2577	2.391146	down	813.7828	778.341	250.8784	283.0512	325.5097	LOC64557	-	hypothetica	645574	chr2	17923032
XM_93319	-2.02118	-1.0152	2.021184	down	-2.53469	-1.34181	2.534692	down	-2.10361	-1.07287	2.10361	down	-2.63806	-1.39948	2.638059	down	-2.05113	-1.03642	2.05113	down	-2.57225	-1.36303	2.572246	down	549.9509	689.673	272.0934	261.432	268.1209	LOC65367	-	similar to b	653675	chr22	21852402
XM_93343	-2.46985	-1.30443	2.469853	down	-2.88042	-1.52628	2.880424	down	-2.24538	-1.16696	2.24538	down	-2.61864	-1.38882	2.618637	down	-2.03797	-1.02713	2.037967	down	-2.37675	-1.24899	2.376745	down	1124.939	1311.941	455.4678	501.0014	551.9905	LOC64613	-	hypothetica	646130	chr11	33806897
XM_93346	-2.26882	-1.18194	2.26882	down	-2.21247	-1.14566	2.212469	down	-2.21684	-1.1485	2.216835	down	-2.16178	-1.11222	2.161776	down	-2.83566	-1.50369	2.835663	down	-2.76523	-1.4674	2.765234	down	946.7883	923.2731	417.3043	427.0901	333.8861	LOC64619	-	hypothetica	646191	chr1	22472748
XM_93347	-3.40279	-1.76672	3.40279	down	-2.19126	-1.13176	2.191264	down	-4.26643	-2.09303	4.266432	down	-2.74742	-1.45808	2.747416	down	-3.38177	-1.75778	3.381773	down	-2.17773	-1.12283	2.17773	down	774.8968	499.0034	227.724	181.6264	229.1392	LOC64622	-	hypothetica	646220	chr18	13207064
XM_93378	-2.35757	-1.2373	2.357569	down	-2.12754	-1.08919	2.127543	down	-2.79193	-1.48126	2.791925	down	-2.51952	-1.33315	2.51952	down	-2.47984	-1.31025	2.479842	down	-2.23789	-1.16214	2.237887	down	900.9075	813.0069	382.1341	322.6832	363.2922	LOC64663	-	hypothetica	646632	chr2	2.22E+08
XM_93388	-6.48942	-2.69809	6.48942	down	-2.45807	-1.29752	2.458066	down	-5.92747	-2.56742	5.927473	down	-2.24521	-1.16685	2.245211	down	-9.19894	-3.20147	9.198939	down	-3.48438	-1.8009	3.484379	down	5483.29	2076.963	844.9583	925.0637	596.0785	LOC64466	-	hypothetica	644663	chr6	1.48E+08
XM_93406	-2.76522	-1.46739	2.765218	down	-3.58676	-1.84268	3.586759	down	-2.39233	-1.25842	2.392331	down	-3.10309	-1.63371	3.103089	down	-2.09806	-1.06905	2.098057	down	-2.72139	-1.44434	2.721386	down	1973.948	2560.404	713.8489	825.1147	940.8457	LOC64452	-	similar to (	644529	chr9	1.27E+08
XM_93412	-2.36012	-1.23886	2.360125	down	-2.01925	-1.01382	2.019253	down	-3.01494	-1.59213	3.014938	down	-2.57949	-1.36709	2.579492	down	-2.38903	-1.25642	2.38903	down	-2.04398	-1.03138	2.043984	down	707.6205	605.4193	299.8234	234.7049	296.1958	GLYCAM	-	glycosylati	644076	chr12	53288243
Y13054	-2.22808	-1.1558	2.228081	down	-2.52031	-1.3336	2.520311	down	-3.0233	-1.59612	3.023295	down	-3.41982	-1.77392	3.419824	down	-3.12407	-1.64343	3.124068	down	-3.53381	-1.82123	3.533814	down	681.6298	771.0309	305.9269	225.4592	218.1866	KIR2DL4	103AS|15.	killer cell i	3805	chr19	60006918

	(AE-1 vs Con) ∩(AE-1 vs Stable) down								(AE-3 vs Con) ∩(AE-3 vs Stable) down								(AE-10 vs Con) ∩(AE-10 vs Stable) down								Normalized Intensities										
**SEQ_ID**	**Fold chang**	**Log Fold c**	**Absolute F**	**Regulation**	**Fold chang**	**Log Fold c**	**Absolute F**	**Regulation**	**Fold chang**	**Log Fold c**	**Absolute F**	**Regulation**	**Fold chang**	**Log Fold c**	**Absolute F**	**Regulation**	**Fold chang**	**Log Fold c**	**Absolute F**	**Regulation**	**Fold chang**	**Log Fold c**	**Absolute F**	**Regulation**	**[Con PBM**	**[Stable PB**	**[AE-1 PBI**	**[AE-3 PBI**	**[AE-10 8P**	**GENE_NA**	**SYNONYI**	**DESCRIP**	**NCBI_GE**	**CHROMC**	**START**
AJ002102	-3.71957	-1.89513	3.719565	down	-3.82965	-1.93721	3.829654	down	-7.40683	-2.88886	7.406831	down	-7.62605	-2.93094	7.626052	down	-4.23646	-2.08286	4.236462	down	-4.36185	-2.12494	4.361849	down	1355.273	1395.385	364.3632	182.9761	319.9067	KIR2DS2	183ACTI|(	killer cell i	3807	chr19_rand	164628
BC022407	-3.00703	-1.58834	3.007033	down	-3.66055	-1.87206	3.660546	down	-4.83658	-2.27399	4.836576	down	-5.8877	-2.5577	5.887699	down	-3.11696	-1.64014	3.116962	down	-3.79437	-1.92386	3.794365	down	1477.961	1799.163	491.5013	305.58	474.1671	SH2D1B	EAT2	SH2 domai	117157	chr1	1.61E+08
BC066595	-3.57574	-1.83824	3.575742	down	-3.21577	-1.68517	3.215773	down	-9.9349	-3.3125	9.934896	down	-8.93475	-3.15943	8.934752	down	-3.57512	-1.83799	3.575125	down	-3.21522	-1.68492	3.215218	down	1823.849	1640.242	510.0617	183.5801	510.1497	SH2D1B	EAT2	SH2 domai	117157	chr1	1.61E+08
BC100911	-11.2134	-3.48716	11.21345	down	-4.39859	-2.13704	4.398593	down	-15.9694	-3.99724	15.96944	down	-6.26418	-2.64713	6.264182	down	-7.89679	-2.98127	7.896788	down	-3.0976	-1.63115	3.097599	down	4300.045	1686.738	383.4722	269.2671	544.5309	CD8B	CD8B1|LY	CD8b mol	926	chr2	86896190
NM_0010(	-3.65606	-1.87029	3.656063	down	-3.13065	-1.64646	3.130652	down	-4.68044	-2.22664	4.680437	down	-4.00781	-2.00282	4.007814	down	-3.67105	-1.87619	3.671048	down	-3.14348	-1.65236	3.143485	down	1076.46	921.7626	294.4315	229.9913	293.2295	OR2W5	OR2W5P|(	olfactory r	441932	chr1	2.46E+08
NM_00493	-10.3033	-3.36503	10.30326	down	-5.28368	-2.40154	5.283679	down	-11.4902	-3.52233	11.49018	down	-5.89235	-2.55884	5.892348	down	-6.55496	-2.71259	6.554957	down	-3.36149	-1.7491	3.361487	down	5385.584	2761.814	522.7066	468.7119	821.6047	CD8B	CD8B1|LY	CD8b mol	926	chr2	86922219
NM_03195	-4.83029	-2.27211	4.830294	down	-4.98916	-2.3188	4.989164	down	-9.83235	-3.29754	9.832348	down	-10.1557	-3.34422	10.15574	down	-3.02997	-1.5993	3.02997	down	-3.12963	-1.64599	3.129626	down	5363.468	5539.874	1110.381	545.4921	1770.139	KSP37	-	Ksp37 prot	83888	chr4	15570963
NM_20163	-15.5828	-3.96189	15.58283	down	-5.55081	-2.4727	5.550806	down	-30.3645	-4.92432	30.36454	down	-10.8162	-3.43513	10.81624	down	-8.92242	-3.15744	8.922422	down	-3.17828	-1.66825	3.178282	down	14506.31	5167.336	930.9164	477.7387	1625.827	TCF7	MGC4773	transcriptic	6932	chr5	1.33E+08

**SEQ_ID**	**Fold chang**	**Log Fold cl**	**Absolute Fc**	**Regulation(**	**Fold chang**	**Log Fold cl**	**Absolute Fc**	**Regulation(**	**[AE-1](nor**	**[AE-3](nor**	**[AE-10](no**	**GENE_NA**	**SYNONYM**	**DESCRIPT**	**NCBI_GEN**	**CHROMO**	**STRAND**	**START**	**STOP**	**GO biologi**	**GO cellular**	**GO molecu**	**UniGene**	**Dbid**	**TIGRID**	**EnsemblID**
XM_49605(	-2.00958	-1.006894	2.0095802	down	-2.026463	-1.018964	2.026463	down	5692.4067	2832.6348	1397.8221	WHDC1L2	-	WAS protei	440253	chr15	+	26782096	26802543							
XM_92857(	-2.017128	-1.012302	2.0171278	down	-2.584037	-1.369627	2.5840372	down	10108.737	5011.451	1939.3881	LOC64555(	-	hypothetical	645550	chr8	+	7411345	7416216							
BC069310	-2.047157	-1.033622	2.047157	down	-2.141696	-1.098754	2.1416964	down	2749.0254	1342.8503	627.0031	GYPB	CD235b|GP	glycophorin	2994	chr4	-	145136760	145159940	GO:000716	GO:000557	GO:000367	Hs.654368	ref:NM_002	NP098195	ENST00000283128
NM_00051	-2.053443	-1.038045	2.0534431	down	-2.672915	-1.418414	2.6729153	down	45653.88	22232.844	8317.826	HBA2	HBA1	hemoglobin	3040	chr16	+	162874	163708	GO:00068	GO:000583	GO:000534	Hs.654744	ref:NM_00(	THC25646	ENST00000251595
XM_93423	-2.054354	-1.038685	2.0543541	down	-3.07149	-1.618939	3.0714905	down	1559.0485	758.8996	247.07861	LOC64508	-	similar to G	645083	chrX	+	49231546	49238845							
NM_00103	-2.056725	-1.040349	2.0567254	down	-2.039601	-1.028287	2.039601	down	779.582	379.0404	185.84047	FLJ46120	-	hypothetical	647008	chr3	-	156438259	156441340							
XM_37307	-2.095933	-1.067593	2.0959332	down	-2.459579	-1.298411	2.4595788	down	2293.8953	1094.4506	444.9748	LOC39176(	-	similar to T	391766	chr5	+	17664894	17665491							
XM_37713(	-2.101305	-1.071285	2.1013045	down	-2.507965	-1.326517	2.5079648	down	16510.705	7857.3594	3132.9624	LOC40165(	-	similar to dc	401650	chr10	+	135327919	135328603							
AK124960	-2.10612	-1.074588	2.1061202	down	-2.040812	-1.029143	2.0408121	down	14118.454	6703.5366	3284.7397	N/A	N/A	Homo sapie	N/A	chr2	-	213849520	213857174							
XM_37787	-2.124965	-1.087439	2.1249655	down	-2.524026	-1.335727	2.524026	down	4191.0063	1972.2703	781.39856	LOC40219	-	similar to T	402199	chr5	-	17586230	17586827							
NM_00181(	-2.126818	-1.088696	2.1268178	down	-7.063597	-2.820403	7.063597	down	8700.114	4090.672	579.12024	CEACAM8	CD66b|CD(	carcinoemb	1088	chr19	-	47776234	47790890	GO:000695	GO:000561	GO:004850	Hs.41	ref:NM_001	THC2475392	
XM_37307	-2.13046	-1.091165	2.1304596	down	-2.067695	-1.048023	2.0676948	down	2356.1104	1105.9165	534.8548	LOC39176	-	similar to T	391763	chr5	-	17641174	17641771							
XM_49585(	-2.143688	-1.100095	2.1436879	down	-2.501972	-1.323066	2.5019721	down	6045.83	2820.294	1127.2284	LOC44001	-	similar to dc	440015	chr3	+	75800771	75801878							
XM_92858	-2.144025	-1.100322	2.1440251	down	-2.066193	-1.046975	2.066193	down	3709.3845	1730.1031	837.33856	LOC645567	-	hypothetical	645567	chr8	+	7418989	7423865							
XM_92799(	-2.164566	-1.114078	2.1645656	down	-2.255348	-1.17335	2.2553484	down	13643.847	6303.2725	2794.811	LOC653541	-	similar to dc	653541	chr4	+	191223373	191224345							
XM_92926	-2.166618	-1.115445	2.1666185	down	-2.307829	-1.206536	2.307829	down	2910.939	1343.5402	582.16626	LOC64632	-	hypothetical	646325	chr4	+	137752667	137756862							
XM_92851	-2.173822	-1.120234	2.1738216	down	-2.148998	-1.103664	2.1489983	down	4225.484	1943.8044	904.5165	LOC64548	-	hypothetical	645489	chr8	+	7391420	7393276							
XM_93086	-2.182976	-1.126296	2.1829756	down	-2.782027	-1.476136	2.7820267	down	3337.4968	1528.875	549.5544	N/A	N/A	PREDICTE	N/A	chr1	-	146671425	146715496							
NM_00147	-2.234589	-1.160009	2.2345887	down	-3.253824	-1.702136	3.2538237	down	1760.2222	787.7164	242.08945	GAGE5	-	G antigen 5	2577	chrX	+	49103600	49110887							
NM_17223	-2.236931	-1.161521	2.2369312	down	-4.114024	-2.04055	4.1140241	down	1180.5848	527.76984	128.28555	REXO1L1	GOR	REX1, RNA	254958	chr8	-	86755784	86762520		GO:000562	GO:000452	Hs.373854	ref:NM_172	THC2690785	
XM_37306	-2.239583	-1.16323	2.2395828	down	-2.955097	-1.563206	2.9550972	down	4800.5845	2143.5173	725.3627	LOC39174	-	similar to T	391749	chr5	-	17589664	17590261							
NM_01661	-2.26201	-1.177606	2.2620105	down	-3.3468	-1.742783	3.3468004	down	31216.213	13800.207	4123.403	SLC25A37	HT015|MFF	solute carrie	51312	chr8	+	23442416	23485681	GO:000681	GO:000573	GO:000538	Hs.122514	gb:AK0939	THC2473820	
XM_49698	-2.272183	-1.184079	2.2721832	down	-4.120853	-2.042943	4.1208533	down	1104.0729	485.9084	117.91451	REXO1L5P	-	REX1, RNA	441361	chr8	-	86861659	86874155							
NM_17587(	-2.296707	-1.199567	2.296707	down	-2.971768	-1.571322	2.971768	down	11404.163	4965.441	1670.871	LOC90925	-	hypothetical	90925	chr14	-	106329757	106330837							
XM_37323	-2.307826	-1.206535	2.3078262	down	-2.571425	-1.362568	2.5714246	down	2567.338	1112.4486	432.61957	LOC39218	-	similar to de	392188	chr8	-	7866592	7868494							
XM_92875	-2.355972	-1.236322	2.3559721	down	-2.177375	-1.12259	2.1773751	down	5594.2944	2374.5164	1090.5408	LOC64574	-	hypothetical	645743	chr8	-	7667630	7669484							
XM_49673	-2.361958	-1.239984	2.3619584	down	-2.051907	-1.036965	2.0519069	down	13457.356	5697.5415	2776.7056	LOC44105(	-	similar to dc	441056	chr4	+	191234862	191237522							
BC015013	-2.365409	-1.242089	2.3654085	down	-3.930527	-1.974723	3.9305269	down	30499.838	12894.11	3280.5042	SLC25A37	HT015|MFF	solute carrie	51312	chr8	+	23442447	23480173	GO:000681	GO:000573	GO:000538	Hs.122514	gb:AK0939	THC2473820	
AY359037	-2.390344	-1.257218	2.3903441	down	-3.990474	-1.99656	3.9904738	down	8413.366	3519.73	882.0331	GFRA3	-	GDNF fami	2676	chr5	-	137615969	137638153	GO:000176	GO:000989	GO:000487	Hs.58042	ref:NM_001	THC24731(	ENST00000274721
XM_49698	-2.40018	-1.263142	2.4001798	down	-4.667398	-2.222619	4.6673983	down	1429.6726	595.6523	127.61977	REXO1L3P	-	REX1, RNA	441359	chr8	-	86743875	86756141							
XM_93512	-2.445644	-1.290214	2.4456441	down	-2.987985	-1.579173	2.9879846	down	1513.8933	619.0162	207.16847	LPAL2	APOA2|AP(	lipoprotein,	80350	chr6	-	160807982	160826893	GO:000815	GO:000557	GO:000367	Hs.654503	ref:NM_145	THC24782	ENST00000335388
NM_01485	-2.448587	-1.291949	2.4485867	down	-2.309609	-1.207649	2.3096092	down	711.00635	290.37418	125.72438	TMCC2	FLJ38497|H	transmembr	9911	chr1	+	203463713	203509093		GO:0016020(membrane		Hs.6360	ref:NM_014	THC24895	ENST00000358024
XM_37324	-2.45129	-1.293541	2.4512896	down	-2.081019	-1.05729	2.0810188	down	599.47595	244.55534	117.51712	LOC392197	-	similar to de	392197	chr8	-	12027334	12029238							
XM_37305	-2.486168	-1.313924	2.4861681	down	-2.322725	-1.215819	2.3227252	down	2568.7163	1033.203	444.8236	LOC39174	-	similar to T	391745	chr5	+	17571520	17572072							
XM_93079	-2.487273	-1.314565	2.4872727	down	-2.492778	-1.317755	2.4927784	down	1798.206	722.96295	290.02295	N/A	N/A	PREDICTE	N/A	chr1	-	146638014	146714022							
XM_92746	-2.503314	-1.323839	2.5033138	down	-2.209409	-1.143661	2.209409	down	3300.607	1318.4951	596.7637	LOC65345	-	similar to de	653455	chr4	+	8950226	8951819							
NM_00101	-2.514404	-1.330216	2.5144039	down	-3.5161	-1.813976	3.5161003	down	1818.9315	723.40466	205.74062	LOC40211(	-	hypothetical	402110	chr4	-	48992527	48995133							
NM_00210(	-2.527428	-1.33767	2.5274276	down	-2.256641	-1.174177	2.2566415	down	570.9863	225.91599	100.1116	GYPB	CD235b|GP	glycophorin	2994	chr4	-	145136706	145159946	GO:000716	GO:000557	GO:000367	Hs.654368	ref:NM_002	NP098195	ENST00000283128
XM_49698	-2.549262	-1.350079	2.5492617	down	-2.01915	-1.013748	2.0191502	down	1443.0928	566.08264	280.35687	REXO1L2P	GOR	REX1, RNA	441362	chr8	-	86873804	86886069							
NM_00277(	-2.553664	-1.352569	2.5536638	down	-2.057966	-1.041219	2.0579656	down	1948.1189	762.8721	370.69235	PRSS2	MGC11118	protease, se	5645	chr7	+	142178777	142182363	GO:000650	GO:000557	GO:000429	Hs.622865	ref:NM_002	THC25222	ENST00000361585
XM_92853	-2.753937	-1.461495	2.7539366	down	-2.038404	-1.02744	2.0384041	down	3861.099	1402.029	687.8072	LOC64550	-	hypothetical	645509	chr8	+	7396047	7400921							
BC030983	-2.842244	-1.50703	2.842244	down	-3.137894	-1.649797	3.1378939	down	18896.701	6648.5146	2118.7825	IGLC1	Constant re	immunoglol	3537	chr22	+	21464983	21578968							
XM_37783(	-2.923624	-1.547758	2.9236244	down	-2.546616	-1.348581	2.546616	down	3439.495	1176.449	461.9656	LOC653442	-	similar to de	653442	chr4	+	8935988	8937581							
XM_37788	-3.13212	-1.64714	3.1321201	down	-2.146362	-1.101893	2.1463621	down	5959.6064	1902.7388	886.4948	LOC402207	-	similar to T	402207	chr5	-	17620570	17621167							
XM_37678	-3.202114	-1.679025	3.202114	down	-2.12725	-1.08899	2.1272504	down	1121.8724	350.35367	164.6979	REXO1L6P	-	REX1, RNA	401467	chr8	-	86885994	86898259							
NM_00034	-5.203593	-2.379508	5.2035926	down	-2.936664	-1.554178	2.9366641	down	4763.0483	915.33844	311.69327	SPTB	HSpTB1	spectrin, bet	6710	chr14	-	64302900	64359619	GO:005101	GO:000562	GO:000377	Hs.417303	ref:NM_001	THC25375	ENST00000389722
NM_00003	-6.397649	-2.677542	6.3976487	down	-6.54387	-2.710144	6.5438701	down	48936.227	7649.0957	1168.8948	ALAS2	ANH1|ASB	aminolevuli	212	chrX	-	55052216	55074136	GO:000166	GO:000573	GO:000387	Hs.522666	ref:NM_00(	THC24645	ENST00000330807
NM_01543	-9.398357	-3.232409	9.3983569	down	-2.452782	-1.294419	2.4527824	down	7424.936	790.0249	322.09335	TRIM58	BIA2|DKFZ	tripartite mc	25893	chr1	+	246087123	246107499		GO:000562	GO:000551	Hs.323858	ref:NM_015	NP073979	ENST00000366481
NM_00011	-9.642423	-3.269396	9.6424229	down	-3.096818	-1.630787	3.0968179	down	5035.36	522.209	168.62761	EPB42	MGC11673	erythrocyte	2038	chr15	-	41276719	41300773	GO:000836	GO:000585	GO:000520	Hs.368642	ref:NM_00(	NP1465858	ENST00000300215
BC099627	-9.930162	-3.311817	9.930162	down	-4.411469	-2.141259	4.411469	down	6234.0137	627.7857	142.30763	EPB42	MGC11673	erythrocyte	2038	chr15	-	41276754	41300394	GO:000836	GO:000585	GO:000520	Hs.368642	ref:NM_00(	NP1465858	ENST00000300215
BC101570	-19.74239	-4.303225	19.742387	down	-2.809396	-1.49026	2.809396	down	5607.074	284.01196	101.0936	SLC4A1	AE1|BND3|	solute carrie	6521	chr17	-	39683305	39700965	GO:000682	GO:000588	GO:000545	Hs.443948	ref:NM_00(	THC24746	ENST00000262418
BC027890	-21.5865	-4.432057	21.586498	down	-5.088833	-2.347335	5.0888333	down	14066.306	651.6252	128.05002	CA1	Car1	carbonic anl	759	chr8	-	86427710	86477586	GO:000673	GO:000573	GO:000408	Hs.23118	ref:NM_001	THC26674	ENST00000256119

SEQ_ID	Fold chang	Log Fold cl	Absolute F	Regulation(	Fold chang	Log Fold cl	Absolute F	Regulation(	[AE-1](nor	[AE-3](nor	[AE-10](no	GENE_NA	SYNONYM	DESCRIPT	NCBI_GEN	CHROMO	STRAND	START	STOP	GO biologi	GO cellular	GO molecu	UniGene	Dbid	TIGRID	EnsemblID
NM_02430	-2.003392	-1.002445	2.003392	down	3.0981957	1.6314283	3.0981957	up	420.3142	209.80128	650.00543	MMP28	MM28|MM	matrix meta	79148	chr17	-	31116988	31146753	GO:000027	GO:000557	GO:000422	Hs.380710	ref:NM_024	THC263577	ENST00000338839
BC002584	-2.007372	-1.005308	2.0073723	down	2.7068733	1.4366273	2.7068733	up	3929.4902	1957.5294	5298.784	CD99	MIC2|MIC2	CD99 mole	4267	chrX	+	2619295	2669348	GO:000715	GO:000573	GO:000551	Hs.654354	ref:NM_002	THC253674	ENST00000381192
AL133657	-2.012777	-1.009187	2.0127768	down	2.1001429	1.0704875	2.1001429	up	279.509	138.86736	291.6413	RPIP8	-	RaP2 intera	10900	chr17	+	39741306	39751563	GO:0007264(small GTP		GO:000508	Hs.500197	ref:NM_006	THC250463	ENST00000225441
BC093698	-2.022516	-1.016151	2.0225164	down	2.4976691	1.3205824	2.4976691	up	850.2233	420.37894	1049.9675	CD28	MGC13829	CD28 mole	940	chr2	+	204279624	204307928	GO:000695	GO:000588	GO:001502	Hs.591629	ref:NM_006	THC265829	ENST00000324106
AY775289	-2.022681	-1.016269	2.022681	down	2.6213261	1.3902968	2.6213261	up	8691.964	4297.249	11264.491	PRMT1	ANM1|HCP	protein argi	3276	chr19	+	54872354	54883516	GO:000170	GO:000563	GO:000551	Hs.20521	ref:NM_198	NP1243329	ENST00000361340
AK131309	-2.030921	-1.022134	2.0309206	down	2.0606008	1.043065	2.0606008	up	501.29095	246.82942	508.6169	ZSCAN2	FLJ20595|Z	zinc finger a	54993	chr15	+	82945254	82967951	GO:000635	GO:000562	GO:000370	Hs.594023	ref:NM_017	THC248218	ENST00000334141
NM_00073	-2.031914	-1.02284	2.0319143	down	3.3050133	1.7246561	3.3050133	up	5883.7183	2895.6528	9570.171	CD247	CD3-ZETA	CD247 mol	919	chr1	-	165666504	165754450	GO:000716	GO:000573	GO:000488	Hs.156445	ref:NM_198	THC260209	ENST00000361719
NM_03195	-2.035559	-1.025425	2.0355588	down	3.245032	1.6982327	3.245032	up	1110.3812	545.49207	1770.1392	KSP37	-	Ksp37 prote	83888	chr4	-	15570963	15573926			GO:001983	Hs.98785	ref:NM_031	THC247394	ENST00000382324
BC060863	-2.060292	-1.042849	2.0602917	down	2.6414516	1.401331	2.6414516	up	363.8402	176.59645	466.47098	ZNF549	FLJ34917	zinc finger p	256051	chr19	+	62730600	62744055	GO:000635	GO:000562	GO:000367	Hs.564295	ref:NM_153	THC247121	ENST00000240719
NM_00054	-2.06493	-1.046093	2.0649302	down	3.3750627	1.7549143	3.3750627	up	821.82684	397.99255	1343.2498	TP53	LFS1|TRP5	tumor prote	7157	chr17	-	7512463	7531642	GO:000628	GO:000562	GO:000073	Hs.654481	ref:NM_000	NP1459124	ENST00000269305
NM_14841	-2.064933	-1.046095	2.0649329	down	2.7812047	1.4757099	2.7812047	up	2680.2505	1297.9843	3609.96	ATXN2L	A2D|A2LG|	ataxin 2-like	11273	chr16	+	28741914	28756059	GO:000815	GO:000557	GO:000367	Hs.460499	ref:NM_145	NP080567	ENST00000336783
XM_93315	-2.065621	-1.046576	2.065621	down	2.2846417	1.1919679	2.2846417	up	930.7989	450.61456	1029.4928	FLJ41352	-	FLJ41352 p	150776	chr2	+	131975414	131992685							
BC080574	-2.071888	-1.050946	2.0718881	down	3.4217343	1.7747278	3.4217343	up	4873.4946	2352.1997	8048.6025	TAPBP	NGS17|TAI	TAP binding	6892	chr6	-	33379395	33389967	GO:000646	GO:000013	GO:001543	Hs.370937	ref:NM_172	NP1186276	ENST00000374562
NM_01626	-2.074857	-1.053012	2.0748569	down	2.3549678	1.2357073	2.3549678	up	708.8237	341.62534	804.51666	LEF1	DKFZp586I	lymphoid er	51176	chr4	-	109188149	109309027	GO:000635	GO:000563	GO:000367	Hs.555947	ref:NM_016	THC260333	ENST00000265165
X13809	-2.084304	-1.059566	2.0843039	down	4.1624232	2.0574237	4.1624232	up	1519.5631	729.05066	3034.6174	POU2F2	40088|OTF2	POU domai	5452	chr19	-	47287071	47328400	GO:000635	GO:000563	GO:000367	Hs.654420	ref:NM_002	THC260435	ENST00000389341
NM_01790	-2.088881	-1.06273	2.0888811	down	2.0822169	1.0581204	2.0822169	up	407.14484	194.91049	405.84592	TPCN1	FLJ20612|K	two pore seg	53373	chr12	+	112143651	112218317	GO:000681	GO:001602	GO:000524	Hs.524763	gb:AB0329	THC260123	ENST00000335509
NM_01642	-2.08948	-1.063144	2.0894797	down	4.3452846	2.1194507	4.3452846	up	416.10718	199.14392	865.337	ZNF219	ZFP219	zinc finger p	51222	chr14	-	20628075	20636639	GO:000635	GO:000562	GO:000370	Hs.250493	ref:NM_016	THC246269	ENST00000360947
NM_00675	-2.089497	-1.063156	2.0894969	down	2.6877284	1.4263874	2.6877284	up	460.51825	220.39671	592.3665	SURF5	MED22|ME	surfeit 5	6837	chr9	-	135197564	135204793	GO:000815	GO:000011	GO:000367	Hs.78354	ref:NM_133	THC249369	ENST00000344469
XM_93181	-2.091201	-1.064332	2.0912008	down	2.3903221	1.257205	2.3903221	up	247.23154	118.22468	282.59506	LOC643795	-	hypothetical	643795	chr10	+	94437622	94439488							
BC031091	-2.092121	-1.064966	2.0921209	down	2.3755395	1.2482552	2.3755395	up	226.1721	108.10661	256.81152	GATAD1	FLJ22489|C	GATA zinc	57798	chr7	+	91914823	91926085	GO:000635	GO:000563	GO:000370	Hs.21145	ref:NM_021	THC253595	ENST00000287957
NM_20704	-2.099672	-1.070164	2.0996715	down	2.0196066	1.0140743	2.0196066	up	886.42035	422.17096	852.61926	ENSA	MGC4319|N	endosulfine	2029	chr1	-	148861222	148868722	GO:0006810(transport);(		GO:000510	Hs.632456	ref:NM_207	THC257165	ENST00000356527
AJ225895	-2.119954	-1.084033	2.1199541	down	2.2547999	1.1729994	2.2547999	up	384.7761	181.50209	409.2509	COLQ	EAD	collagen-lik	8292	chr3	-	15467781	15538255	GO:000150	GO:0005605(basal lamir		Hs.146735	ref:NM_080	NP423138	ENST00000383788
NM_00100	-2.119991	-1.084058	2.1199906	down	2.2064344	1.1417169	2.2064344	up	542.5135	255.90373	564.6348	RP3-377H1	FLJ35429	hypothetical	285830	chr6	-	29810783	29824749	GO:000695	GO:0016020(membrane		Hs.655949	gb:AK0927	THC2560132	
XM_92962	-2.122268	-1.085607	2.1222676	down	2.5775955	1.3660259	2.5775955	up	2495.8945	1176.0508	3031.3833	LOC646675	-	similar to ac	646675	chr22	-	33316141	33316573							
NM_00196	-2.125424	-1.08775	2.1254237	down	2.3511833	1.233387	2.3511833	up	13824.476	6504.339	15292.893	EEF2	EEF-2|EF2	eukaryotic t	1938	chr19	-	3927053	3936446	GO:0006412(translation		GO:000016	Hs.515070	ref:NM_001	THC256721	ENST00000309311
NM_00332	-2.126116	-1.08822	2.1261157	down	3.5561123	1.8303009	3.5561123	up	491.65826	231.24718	822.34094	TULP3	MGC45295	tubby like p	7289	chr12	+	2870293	2920565	GO:000635	GO:000563	GO:000554	Hs.655333	ref:NM_003	NP1151221	ENST00000228245
BC008854	-2.12946	-1.090488	2.1294599	down	2.6980663	1.4319258	2.6980663	up	947.60474	444.9977	1200.6333	C1orf2	COTE1|cote	chromosom	10712	chr1	-	153483622	153491762	GO:000815	GO:001602	GO:000367	Hs.348308	ref:NM_006	NP1167666	ENST00000361361
NM_01660	-2.133663	-1.093332	2.1336631	down	2.078374	1.0554553	2.078374	up	304.25174	142.59596	296.36774	ARMCX3	ALEX3|DK	armadillo re	51566	chrX	+	100764775	100769487		GO:001602	GO:000548	Hs.592225	ref:NM_016	THC276066	ENST00000341189
AF119912	-2.138092	-1.096324	2.138092	down	2.3274452	1.2187472	2.3274452	up	250.73065	117.26841	272.9358	N/A	N/A	Homo sapie	N/A	chr10	+	51764303	51765882							
AF466365	-2.141767	-1.098802	2.1417671	down	2.0642737	1.0456343	2.0642737	up	834.11664	389.45255	803.93665	MGC22014	KIAA0401	hypothetical	200424	chr2	+	74127794	74154538				Hs.516107	gb:AB0078	THC2619304	
NM_02197	-2.146617	-1.102065	2.1466174	down	3.2530579	1.7017965	3.2530579	up	5263.4727	2451.9846	7976.4478	RELA	MGC13177	v-rel reticul	5970	chr11	-	65178392	65186951	GO:000194	GO:000563	GO:000370	Hs.502875	gb:BC0140	NP099839	ENST00000308546
BC002751	-2.1498	-1.104202	2.1497998	down	2.5092716	1.3272686	2.5092716	up	1294.1632	601.99243	1510.5625	TAP2	ABC18|AB	transporter 2	6891	chr6	-	32904244	32913994	GO:000646	GO:000578	GO:000016	Hs.502	ref:NM_000	THC246214	ENST00000374897
NM_00259	-2.158444	-1.109992	2.1584439	down	2.0729733	1.0517015	2.0729733	up	216.4919	100.29999	207.9192	PDE2A	PDE2A1|PE	phosphodies	5138	chr11	-	71964832	72063060	GO:000716	GO:001602	GO:000382	Hs.503163	ref:NM_002	THC246695	ENST00000334456
NM_00101	-2.160076	-1.111082	2.160076	down	4.0407274	2.014615	4.0407274	up	419.90652	194.39433	785.4945	Gcom1	FLJ30973|G	GRINL1A c	145781	chr15	+	55671405	55786445				Hs.437256	ref:NM_001	NP1072819	ENST00000267853
AY847301	-2.171041	-1.118387	2.1710413	down	4.8582104	2.280425	4.8582104	up	278.59573	128.32355	623.4228	P2RX7	MGC20089	purinergic r	5027	chr12	+	120055156	120106987	GO:000681	GO:000588	GO:000487	Hs.507102	ref:NM_002	NP1456785	ENST00000261826
AF237775	-2.181351	-1.125222	2.1813515	down	2.0940508	1.0662965	2.0940508	up	4745.716	2175.5852	4555.786	MKNK2	GPRK7|MN	MAP kinase	2872	chr19	-	1990601	2001886	GO:0006417(regulation		GO:000016	Hs.515032	ref:NM_017	THC249222	ENST00000250896
NM_15344	-2.196353	-1.13511	2.1963528	down	2.0894202	1.0631027	2.0894202	up	804.20667	366.1555	765.0527	KIR3DL3	CD158Z|KI	killer cell in	115653	chr19	+	59927795	59939815				Hs.645224	ref:NM_153	THC247638	ENST00000291860
AK056002	-2.199791	-1.137366	2.1997907	down	2.6988243	1.4323311	2.6988243	up	3130.3147	1423.0057	3840.4424	ATXN7L3	DKFZp761	ataxin 7-like	56970	chr17	-	39625490	39629911				Hs.512651	gb:BC0374	THC254252	ENST00000311910
BC069505	-2.200009	-1.137509	2.2000089	down	2.1848626	1.1275425	2.1848626	up	612.5692	278.43942	608.35187	BMF	FLJ00065	Bcl2 modify	90427	chr15	-	38171210	38185915	GO:000691	GO:001645	GO:000551	Hs.591104	ref:NM_001	THC265102	ENST00000354670
XM_92962	-2.23048	-1.157354	2.2304802	down	2.8150291	1.4931498	2.8150291	up	608.3852	272.75974	767.8266	LOC646677	-	similar to ac	646677	chr22	-	33316623	33316974							
BC002772	-2.23556	-1.160636	2.2355595	down	2.4252579	1.2781382	2.4252579	up	752.693	336.6911	816.56274	NDUFA6	B14|CI-B14	NADH dehy	4700	chr22	-	40811476	40816695	GO:000612	GO:000573	GO:000395	Hs.274416	ref:NM_002	THC257686	ENST00000331989
NM_02084	-2.235713	-1.160735	2.2357131	down	2.4898603	1.3160648	2.4898603	up	561.3698	251.09206	625.18414	PITPNM2	KIAA1457|	phosphatidy	57605	chr12	-	122035654	122085151	GO:000681	GO:000562	GO:000550	Hs.272759	ref:NM_020	THC260171	ENST00000320201
NM_08064	-2.241196	-1.164269	2.2411956	down	2.1490553	1.1037026	2.1490553	up	3981.4727	1776.495	3817.786	APEX1	APE|APE1|	APEX nucle	328	chr14	+	19993129	19995766	GO:000628	GO:000563	GO:000028	Hs.73722	ref:NM_080	THC251727	ENST00000216714
BC001265	-2.244358	-1.166303	2.2443584	down	2.3005953	1.2020072	2.3005953	up	246.72705	109.93211	252.90929	NPAL3	DJ462O23.2	NIPA-like d	57185	chr1	+	24614889	24653900		GO:0016020(membrane		Hs.523442	ref:NM_020	THC255538	ENST00000389489
AY456380	-2.259433	-1.175961	2.2594327	down	2.9507953	1.5611038	2.9507953	up	778.73883	344.66122	1017.0247	MINA	DKFZp762	MYC induc	84864	chr3	-	99146407	99173516				Hs.655935	ref:NM_032	THC278129	ENST00000360258
AY766457	-2.265474	-1.179813	2.2654739	down	2.3233999	1.2162375	2.3233999	up	430.2991	189.93779	441.30145	CCL4L2	AT744.2|CC	chemokine (	388372	chr17	+	31562577	31564393							
NM_00103	-2.269489	-1.182367	2.2694887	down	2.0381366	1.0272508	2.0381366	up	259.9788	114.55391	233.47652	LOC148137	DKFZp434	hypothetical	148137	chr19	+	40940883	40951917				Hs.527982	ref:NM_001	THC247592	ENST00000301165
AB032992	-2.284143	-1.191653	2.2841434	down	2.0946622	1.0667176	2.0946622	up	272.72098	119.39748	250.0974	KIAA1166	HCA127	KIAA1166	55906	chrX	-	64053092	64113138				Hs.28249	ref:NM_018	THC264187	ENST00000374839
BC011603	-2.287182	-1.193571	2.287182	down	3.1308804	1.6465684	3.1308804	up	3895.7407	1703.2928	5332.806	RELA	MGC13177	v-rel reticul	5970	chr11	-	65177648	65186942	GO:000194	GO:000563	GO:000370	Hs.502875	gb:BC0140	NP099839	ENST00000308546
NM_00100	-2.287865	-1.194002	2.2878645	down	2.049805	1.0354867	2.049805	up	384.78754	168.18633	344.74918	FAM116B	MGC33692	family with	414918	chr22	-	49092963	49100010				Hs.530380	ref:NM_001	THC257060	ENST00000380836
BC037906	-2.290941	-1.195941	2.2909414	down	3.6227344	1.857079	3.6227344	up	245.50046	107.16139	388.21725	C4orf19	FLJ11017	chromosom	55286	chr4	+	37131994	37269843				Hs.107527	ref:NM_018	THC247286	ENST00000284437
BC107037	-2.295451	-1.198778	2.2954511	down	5.1888314	2.3754097	5.1888314	up	363.8402	158.50488	822.4551	GIMAP8	DKFZp667I	GTPase, IM	155038	chr7	+	149779067	149806225			GO:000552	Hs.647121	ref:NM_175	THC270777	ENST00000307271
NM_01543	-2.310313	-1.208088	2.3103131	down	3.1247943	1.6437612	3.1247943	up	428.1068	185.3025	579.0322	PLEKHG4	DKFZP434I	pleckstrin h	25894	chr16	+	65868913	65880884	GO:000821	GO:000562	GO:000508	Hs.188781	ref:NM_015	THC246420	ENST00000360461
BC101578	-2.311108	-1.208585	2.3111081	down	2.2515633	1.170927	2.2515633	up	656.293	283.9733	639.38385	PBX1	DKFZp686I	pre-B-cell le	5087	chr1	+	162795631	163082580	GO:000165	GO:000563	GO:000370	Hs.654412	ens:ENST0	THC253175	ENST00000328681
BC101468	-2.332937	-1.222148	2.3329373	down	2.6945988	1.4300705	2.6945988	up	277.36438	118.89063	320.36255	FYCO1	FLJ13335|M	FYVE and c	79443	chr3	-	45938208	46001491	GO:000681	GO:001602	GO:000827	Hs.200227	ref:NM_024	THC260168	ENST00000296137
NM_02183	-2.335092	-1.223479	2.3350918	down	2.4890624	1.3156024	2.4890624	up	1011.7167	433.26636	1078.427	FLJ21839	-	hypothetical	60509	chr2	+	27128052	27146994	GO:000650	GO:000573	GO:000418	Hs.138207	ref:NM_021	THC248677	ENST00000380348
BC028020	-2.338149	-1.225367	2.3381485	down	2.4546395	1.2955111	2.4546395	up	1732.4722	740.959	1818.7872	CENTG1	AGAP2|FLJ	centaurin, g	116986	chr12	-	56406365	56422178	GO:000716	GO:000562	GO:000016	Hs.302435	ens:ENST0	THC267476	ENST00000257897
XM_93374	-2.340467	-1.226796	2.3404666	down	2.1118708	1.0785216	2.1118708	up	1201.2653	513.2589	1083.9365	LOC441253	FLJ41820	nuclear env	441253	chr7	-	74904768	74953505							
XM_49637	-2.347712	-1.231256	2.3477122	down	2.1690417	1.1170578	2.1690417	up	385.12268	164.04169	355.81326	LOC401957	-	hypothetical	401957	chr1	+	106236495	106237113							
AF516753	-2.353884	-1.235043	2.3538838	down	3.4970756	1.806149	3.4970756	up	351.27316	149.23131	521.87317	APITD1	MGC32686	apoptosis-in	378708	chr1	+	10413027	10434302		GO:0000775(chromoson		Hs.412311	ref:NM_199	THC2470575	
NM_00286	-2.366009	-1.242455	2.3660087	down	2.555783	1.3537654	2.555783	up	689.4072	291.37982	744.7036	RAB3A	-	RAB3A, me	5864	chr19	-	18168610	18175839	GO:000726	GO:000802	GO:000016	Hs.27744	ref:NM_002	THC263729	ENST00000222256
BC051858	-2.39463	-1.259803	2.3946302	down	2.1175998	1.08243	2.1175998	up	613.05286	256.0115	542.1299	ADIPOR2	ACDCR2|F	adiponectin	79602	chr12	+	1670507	1765687	GO:000662	GO:001602	GO:000487	Hs.371642	ref:NM_024	THC247426	ENST00000357103
L15409	-2.395416	-1.260276	2.3954156	down	2.4168	1.2730981	2.4168	up	367.54138	153.43533	370.8225	VHL	HRCA1|RC	von Hippel-	7428	chr3	+	10158318	10167603	GO:000012	GO:000563	GO:000813	Hs.517792	ref:NM_000	THC253766	ENST00000256474
NM_00549	-2.413945	-1.271393	2.4139449	down	2.9052185	1.5386467	2.9052185	up	1475.7388	611.33905	1776.0735	AP1M2	HSMU1B|M	adaptor-rela	10053	chr19	-	10544346	10558991	GO:000660	GO:000590	GO:000551	Hs.18894	ref:NM_005	THC278051	ENST00000250244
AK027287	-2.423835	-1.277291	2.4238348	down	2.2171634	1.1487151	2.2171634	up	1824.0947	752.5656	1668.5609	CIZ1	LSFR1|ZNF	CDKN1A ir	25792	chr9	-	129968170	129993672	GO:000007	GO:000562	GO:000367	Hs.212395	ref:NM_012	THC247999	ENST00000372938
BC009903	-2.433969	-1.283311	2.4339691	down	3.7930915	1.9233742	3.7930915	up	3105.2288	1275.7881	4839.181	SF3A2	PRP11|PRP	splicing fact	8175	chr19	+	2187822	2199653	GO:000038	GO:000562	GO:000367	Hs.115232	ref:NM_007	THC246872	ENST00000221494
BC039891	-2.437301	-1.285284	2.4373006	down	4.4501929	2.1538679	4.4501929	up	1703.8008	699.0524	3110.918	ADCY7	FLJ36387|K	adenylate cy	113	chr16	+	48865573	48901927	GO:000617	GO:000588	GO:000028	Hs.513578	ref:NM_001	THC251633	ENST00000254235
BC110543	-2.451295	-1.293544	2.4512945	down	2.135143	1.0943327	2.135143	up	1142.363	466.02438	995.0287	NNT	MGC12650	nicotinamid	23530	chr5	+	43639135	43740750	GO:000609	GO:000573	GO:0003957(NAD(P)+ t		ens:ENST0	THC266610	ENST00000381528
NM_02090	-2.459	-1.298072	2.4590002	down	2.6379826	1.3994351	2.6379826	up	1735.3387	705.70905	1861.6482	KIAA1542	-	CTD-bindin	57661	chr11	+	566485	602220			GO:000551	Hs.325838	ref:NM_020	THC260532	ENST00000264555
NM_00134	-2.474551	-1.307167	2.4745506	down	2.1060889	1.0745663	2.1060889	up	418.18964	168.9962	355.92102	DAB2	DOC-2|DO	disabled hor	1601	chr5	-	39408487	39460703	GO:000090	GO:000590	GO:000802	Hs.481980	ref:NM_001	THC248713	ENST00000339788
AF435977	-2.480516	-1.31064	2.480516	down	2.0879582	1.0620928	2.0879582	up	491.5013	198.14478	413.71802	SON	BASS1|C21	SON DNA b	6651	chr21	+	33837253	33846442	GO:000691	GO:000557	GO:000367	Hs.517262	ref:NM_138	THC248150	ENST00000356577
BC069328	-2.483168	-1.312182	2.4831684	down	2.211922	1.1453005	2.211922	up	414.5149	166.92984	369.23578	BMF	FLJ00065	Bcl2 modify	90427	chr15	-	38171210	38185915	GO:000691	GO:001645	GO:000551	Hs.591104	ref:NM_001	THC265102	ENST00000354670
NM_00253	-2.498331	-1.320965	2.4983313	down	2.1860833	1.1283484	2.1860833	up	2959.2607	1184.4949	2589.4045	ODC1	-	ornithine de	4953	chr2	-	10497958	10505904	GO:000659	GO:000557	GO:000458	Hs.467701	ref:NM_002	THC258113	ENST00000234111
BC104983	-2.508475	-1.326811	2.5084755	down	3.0338337	1.601142	3.0338337	up	1180.9259	470.77435	1428.2511	BMF	FLJ00065	Bcl2 modify	90427	chr15	-	38171252	38185866	GO:000691	GO:001645	GO:000551	Hs.591104	ref:NM_001	THC265102	ENST00000354670
AF028825	-2.515083	-1.330606	2.5150829	down	3.2361087	1.6942601	3.2361087	up	499.42514	198.57204	642.6007	DLG4	PSD95|SAP	discs, large	1742	chr17	-	7046766	7061231	GO:000646	GO:003086	GO:000551	Hs.463928	ref:NM_001	THC246385	ENST00000293813
AK130831	-2.554905	-1.35327	2.554905	down	2.0681215	1.0483209	2.0681215	up	2800.5198	1096.1346	2266.9395	PLCB2	FLJ38135	phospholipa	5330	chr15	-	38367391	38377429	GO:0006644(phospholip		GO:000443	Hs.355888	gb:BC0090	THC2480920	
NM_01500	-2.563541	-1.358138	2.5635412	down	3.3097617	1.7267274	3.3097617	up	730.51483	284.96317	943.1602	GOLGA8A	GM88	golgi autoar	23015	chr15	-	32458560	32487180				Hs.182982	ref:NM_181	THC258366	ENST00000360553
BC008180	-2.574437	-1.364257	2.5744374	down	3.5803074	1.8400835	3.5803074	up	483.84518	187.9421	672.8905	METTL7A	AAM-B|DK	methyltrans	25840	chr12	+	49605082	49611596	GO:0008152(metabolic		GO:000816	Hs.655369	ref:NM_014	NP075556	ENST00000332160
NM_00419	-2.579213	-1.366931	2.579213	down	2.1324757	1.0925293	2.1324757	up	645.54865	250.289	533.7352	P4HA2	4-PH alpha	procollagen	8974	chr5	-	131556201	131590834	GO:001840	GO:000578	GO:000465	Hs.519568	ref:NM_004	THC248397	ENST00000358760
NM_00006	-2.595904	-1.376237	2.5959037	down	3.0290662	1.5988731	3.0290662	up	1247.1844	480.44324	1455.2944	CA2	CA II|CA-II	carbonic anl	760	chr8	+	86563497	86580973	GO:000200	GO:000573	GO:000408	Hs.155097	ref:NM_000	THC246887	ENST00000285379
BC010691	-2.6343	-1.39742	2.6343003	down	2.8380369	1.5048934	2.8380369	up	281.8557	106.99452	303.6544	HCP1	MGC9564	heme carrie	113235	chr17	-	23750177	23757346	GO:000681	GO:001602	GO:000521	Hs.446689	ref:NM_080	THC2496566	
XM_93519	-2.644527	-1.40301	2.6445271	down	2.1392306	1.097092	2.1392306	up	365.7336	138.2983	295.85196	LOC440456	-	similar to pl	440456	chr17	-	60231440	60263716	GO:0007242(intracellula		GO:000827	Hs.659276	gb:BC0323	THC258961	ENST00000316961
XM_92883	-2.688826	-1.426976	2.6888256	down	4.6261629	2.2098161	4.6261629	up	1224.5586	455.42508	2106.8706	LOC645852	-	similar to sp	645852	chr3	-	127873638	127874475							
NM_01749	-2.702915	-1.434516	2.7029147	down	2.5598159	1.3560401	2.5598159	up	1230.6489	455.30438	1165.4954	ATXN2L	A2D|A2LG|	ataxin 2-like	11273	chr16	+	28741914	28756059	GO:000815	GO:000557	GO:000367	Hs.460499	ref:NM_145	NP080567	ENST00000336783
XM_93316	-2.765459	-1.467519	2.7654588	down	2.5648126	1.3588534	2.5648126	up	1002.8563	362.6365	930.09467	FLJ41352	-	FLJ41352 p	150776	chr2	+	131990900	131992952							
BC073805	-2.773347	-1.471628	2.7733471	down	2.3211734	1.2148543	2.3211734	up	4483.9224	1616.7909	3752.852	ZNF282	HUB1	zinc finger p	8427	chr7	+	148523593	148553137	GO:000635	GO:000562	GO:000367	Hs.657701	ref:NM_003	NP085674	ENST00000262085
BC066595	-2.778415	-1.474262	2.7784153	down	2.7788947	1.4745112	2.7788947	up	510.06165	183.58006	510.14966	SH2D1B	EAT2	SH2 domair	117157	chr1	-	160632364	160648526	GO:000724	GO:000557	GO:000367	Hs.350581	ref:NM_053	THC248799	ENST00000367929
XM_92775	-2.79713	-1.483947	2.7971299	down	2.8763856	1.5242571	2.8763856	up	430.4391	153.88599	442.63544	ATP9A	ATPIIA|KIA	ATPase, Cla	10079	chr20	-	49646465	49650249	GO:000681	GO:001602	GO:000016	Hs.592144	ref:NM_006	THC258070	ENST00000338821
NM_18183	-2.820375	-1.495887	2.8203746	down	2.2317131	1.1581515	2.2317131	up	746.56085	264.70273	590.74054	NF2	ACN|BANF	neurofibrom	4771	chr22	+	28329564	28409904	GO:000704	GO:000563	GO:000519	Hs.187898	ref:NM_181	NP1166189	ENST00000338641

BC013694	-2.826939	-1.499241	2.826939	down	3.2857369	1.7162169	3.2857369	up	4980.513	1761.8042	5788.825	PTBP1	HNRNPI|H	polypyrimid	5725	chr19	+	748478	762012	GO:000639	GO:000563	GO:000016	Hs.172550	ref:NM_002	THC250052	ENST00000349038
AK126599	-2.840814	-1.506304	2.8408137	down	4.5866613	2.1974444	4.5866613	up	445.05096	156.6632	718.56104	N/A	N/A	Homo sapie	N/A	chr11	-	65147801	65149679							
AK123224	-2.86621	-1.519144	2.8662097	down	5.590787	2.4830514	5.590787	up	10906.64	3805.2485	21274.334	N/A	N/A	Homo sapie	N/A	chr16	-	29960415	30418376							
NM_01493	-2.881887	-1.527014	2.881887	down	2.765786	1.4676896	2.765786	up	3365.3599	1167.7626	3229.7815	MLXIP	KIAA0867|	MLX intera	22877	chr12	+	121177743	121192312	GO:004544	GO:000563	GO:003052	Hs.437153	ref:NM_014	THC25787	ENST00000377037
BC011971	-2.904925	-1.538501	2.9049247	down	2.4929599	1.3178597	2.4929599	up	596.9862	205.50832	512.324	AGPAT3	LPAAT-GA	1-acylglyce	56894	chr21	+	44109569	44226862	GO:000815	GO:000557	GO:000384	Hs.248785	ref:NM_020	THC24628	ENST00000358148
U73394	-2.912095	-1.542057	2.9120951	down	3.0655391	1.6161408	3.0655391	up	359.85834	123.57369	378.81998	KIR2DL4	103AS|15.2	killer cell in	3805	chr19	+	60006906	60017780	GO:000696	GO:000588	GO:000487	Hs.661219	ref:NM_002	NP365687	ENST00000359085
AY207007	-2.95001	-1.56072	2.9500103	down	4.126347	2.0448651	4.126347	up	584.6982	198.20209	817.8506	Gcom1	FLJ30973|G	GRINL1A c	145781	chr15	+	55671522	55794235				Hs.437256	ref:NM_001	NP1072819	ENST00000267853
BC013009	-2.973775	-1.572296	2.9737755	down	3.1886917	1.6729646	3.1886917	up	582.834	195.99126	624.9557	ZMYM3	DXS6673E|	zinc finger,	9203	chrX	-	70385779	70391148	GO:000727	GO:000563	GO:000367	Hs.522684	ref:NM_005	THC24804	ENST00000314425
NM_00162	-2.975538	-1.573151	2.9755381	down	4.1111969	2.0395585	4.1111969	up	1486.2148	499.47766	2053.451	AKT2	PKBBETA|	v-akt murin	208	chr19	-	45431555	45483036	GO:000646	GO:003002	GO:000016	Hs.631535	ref:NM_001	THC253251	ENST00000311278
AK054965	-2.991778	-1.581003	2.9917784	down	2.565011	1.358965	2.565011	up	475.33234	158.87953	407.52774	N/A	N/A	Homo sapie	N/A	chr19	-	56786847	56789442							
NM_00716	-2.995047	-1.582579	2.9950469	down	4.2882682	2.1003951	4.2882682	up	3777.9417	1261.3965	5409.2065	SF3A2	PRP11|PRP	splicing fact	8175	chr19	+	2187815	2199678	GO:000038	GO:000562	GO:000367	Hs.115232	ref:NM_007	THC246872	ENST00000221494
XM_49695	-3.008958	-1.589264	3.0089578	down	2.9900639	1.5801763	2.9900639	up	1669.7161	554.9151	1659.2316	LOC44132	-	chromosom	441320	chr8	-	7083211	7083563							
AK026042	-3.05236	-1.609925	3.0523603	down	2.3764923	1.2488337	2.3764923	up	763.80505	250.23424	594.67975	NUP210	FLJ22389|G	nucleoporin	23225	chr3	-	13368177	13392851	GO:000660	GO:000563	GO:000551	Hs.475525	ref:NM_024	THC25481	ENST00000388753
BC040603	-3.068948	-1.617744	3.0689485	down	5.0147589	2.3261803	5.0147589	up	825.0798	268.84772	1348.2065	AGPAT3	LPAAT-GA	1-acylglyce	56894	chr21	+	44191238	44227026	GO:000815	GO:000557	GO:000384	Hs.248785	ref:NM_020	THC24628	ENST00000358148
BC012303	-3.082424	-1.624065	3.0824242	down	2.1093137	1.0767737	2.1093137	up	10440.389	3387.0708	7144.395	PDZK1IP1	DD96|MAP	PDZK1 inte	10158	chr1	-	47421852	47428271		GO:0005576(extracellul		Hs.431099	ref:NM_005	THC26125	ENST00000294338
BC014212	-3.131077	-1.646659	3.131077	down	3.9071353	1.9661112	3.9071353	up	3035.137	969.3588	3787.416	FBS1	FBS|FLJ11	fibrosin 1	64319	chr16	+	30584155	30588359				Hs.247186	ref:NM_022	THC25788	ENST00000287468
BC031654	-3.131715	-1.646953	3.1317154	down	3.150161	1.6554256	3.150161	up	553.9955	176.89842	557.2585	FAM53B	KIAA0140|	family with	9679	chr10	-	126345752	126422828	GO:000815	GO:000557	GO:000367	Hs.129195	ref:NM_014	THC24758	ENST00000337318
BC031562	-3.173596	-1.666119	3.1735962	down	5.0906799	2.3478584	5.0906799	up	877.8108	276.59814	1408.0726	AKR7A3	AFAR2	aldo-keto re	22977	chr1	-	19481666	19487809	GO:000608	GO:000582	GO:000403	Hs.6980	ref:NM_012	THC24753	ENST00000361640
BC028309	-3.20266	-1.679271	3.2026604	down	2.2789854	1.1883917	2.2789854	up	8208.072	2562.8918	5840.793	MLXIP	KIAA0867|	MLX intera	22877	chr12	+	121178388	121184663	GO:004544	GO:000563	GO:003052	Hs.437153	ref:NM_014	THC25787	ENST00000377037
AY128689	-3.203304	-1.679561	3.2033039	down	3.7408102	1.9033508	3.7408102	up	473.8027	147.91063	553.3056	CENTG1	AGAP2|FL	centaurin, g	116986	chr12	-	56406601	56418296	GO:000716	GO:000562	GO:000016	Hs.302435	ens:ENST0	THC26747	ENST00000257897
NM_03296	-3.291968	-1.71895	3.291968	down	3.0015638	1.5857143	3.0015638	up	1913.7395	581.336	1744.9171	CCL14	CC-1|CC-3	chemokine (	6358	chr17	-	31334804	31337852							
AK126136	-3.311329	-1.72741	3.3113288	down	4.7013192	2.2330656	4.7013192	up	1367.5659	412.9961	1941.6265	AKNA	KIAA1968|	AT-hook tra	80709	chr9	-	116177898	116200573				Hs.494895	ref:NM_030	NP434202	ENST00000307564
NM_00073	-3.311783	-1.727608	3.3117833	down	2.9045762	1.5383277	2.9045762	up	334.32315	100.94959	293.21576	CHRM1	HM1|M1|M	cholinergic	1128	chr11	-	62432726	62445588	GO:000646	GO:000562	GO:000158	Hs.632119	ref:NM_000	THC270057	ENST00000306960
BC027459	-3.33066	-1.735808	3.3306596	down	2.1970172	1.1355462	2.1970172	up	348.54956	104.64881	229.91524	PIP5K2B	PIP5KIIB|P	phosphatidy	8396	chr17	-	34185753	34209301	GO:000716	GO:000573	GO:000505	Hs.260603	ref:NM_003	THC2586937	
NM_00363	-3.39068	-1.761575	3.39068	down	6.1342341	2.6168832	6.1342341	up	3076.4958	907.33887	5565.829	KCNAB2	AKR6A5|H	potassium v	8514	chr1	+	6008966	6083110	GO:000681	GO:000573	GO:000521	Hs.440497	ref:NM_003	THC24696	ENST00000341524
NM_00687	-3.477512	-1.798056	3.4775125	down	5.5460015	2.471448	5.5460015	up	2827.494	813.07947	4509.34	DSTN	ACTDP|AD	destrin (acti	11034	chr20	+	17498598	17536652	GO:000815	GO:000562	GO:000377	Hs.304192	ref:NM_001	THC24799	ENST00000246069
BC104873	-3.625565	-1.858206	3.6255649	down	3.0362189	1.6022758	3.0362189	up	391.17813	107.89439	327.591	CREBL2	MGC11731	cAMP respo	1389	chr12	+	12656189	12687308	GO:000635	GO:000563	GO:000370	Hs.591156	ref:NM_001	THC250391	ENST00000228865
NM_00617	-3.67933	-1.879443	3.6793304	down	3.2878839	1.7171593	3.2878839	up	18143.514	4931.2	16213.213	NRGN	RC3|hng	neurogranin	4900	chr11	+	124114951	124122309	GO:0007165(signal tran		GO:000551	Hs.524116	ref:NM_006	THC24615	ENST00000284292
BC063552	-3.698201	-1.886824	3.6982009	down	6.9462217	2.7962285	6.9462217	up	615.64136	166.4705	1156.341	AGPAT3	LPAAT-GA	1-acylglyce	56894	chr21	+	44169316	44227410	GO:000815	GO:000557	GO:000384	Hs.248785	ref:NM_020	THC24628	ENST00000358148
BC040943	-3.701461	-1.888095	3.7014614	down	2.2292332	1.1565476	2.2292332	up	3801.9624	1027.1517	2289.7607	WASF2	SCAR2|WA	WAS protei	10163	chr1	-	27606887	27689247	GO:000718	GO:000585	GO:000377	Hs.590909	ref:NM_006	THC254474	ENST00000374019
XM_93295	-3.776419	-1.917019	3.7764192	down	3.1024985	1.6334305	3.1024985	up	2668.7747	706.6945	2192.5186	LOC653307	-	similar to hy	653307	chr5	-	176895536	176914084							
BC002835	-3.816979	-1.932431	3.8169789	down	2.7978133	1.4842997	2.7978133	up	19404.535	5083.7417	14223.36	NRGN	RC3|hng	neurogranin	4900	chr11	+	124115150	124122312	GO:0007165(signal tran		GO:000551	Hs.524116	ref:NM_006	THC24615	ENST00000284292
AF225896	-3.920182	-1.970921	3.9201822	down	3.6298095	1.8598938	3.6298095	up	7033.781	1794.2485	6512.7803	N/A	N/A	Homo sapie	N/A	chr2	-	218376876	218517006							
BC023998	-3.942846	-1.979238	3.9428465	down	3.931881	1.9752197	3.931881	up	1009.1406	255.94214	1006.3341	MTSS1	FLJ44694|K	metastasis s	9788	chr8	-	125634309	125650243	GO:000692	GO:000172	GO:000378	Hs.336994	ref:NM_014	THC248787	ENST00000325064
XM_93151	-3.980976	-1.993122	3.9809755	down	3.071743	1.6190575	3.071743	up	1308.8508	328.7764	1009.9166	UBXD7	KIAA0794	UBX domai	26043	chr3	-	197580692	197643672				Hs.518524	ens:ENST0	THC251277	ENST00000296328
NM_00576	-4.329795	-2.114299	4.3297954	down	2.2114504	1.1449929	2.2114504	up	13880.122	3205.7224	7089.296	PDZK1IP1	DD96|MAP	PDZK1 inte	10158	chr1	-	47421847	47428358		GO:0005576(extracellul		Hs.431099	ref:NM_005	THC26125	ENST00000294338
BC026002	-4.590517	-2.198657	4.5905168	down	2.9709912	1.5709443	2.9709912	up	1009.8599	219.98828	653.58325	GRAP2	GADS|GRA	GRB2-relat	9402	chr22	+	38672801	38697330	GO:000724	GO:000573	GO:000507	Hs.517499	ref:NM_004	THC262474	ENST00000344138
AK127453	-4.679191	-2.226259	4.6791914	down	5.706087	2.5125017	5.706087	up	993.36005	212.2931	1211.3629	N/A	N/A	Homo sapie	N/A	chr19	-	7441415	7444255							
NM_00394	-5.588296	-2.482409	5.5882964	down	4.1105312	2.0393248	4.1105312	up	13489.678	2413.916	9922.477	SELENBP1	FLJ13813|L	selenium bi	8991	chr1	-	149603403	149611788			GO:000843	Hs.632460	ref:NM_003	NP1180129	ENST00000368868
BC090921	-6.185144	-2.628807	6.1851435	down	4.1154795	2.0410605	4.1154795	up	3571.5598	577.4417	2376.4495	MYH9	DFNA17|F	myosin, hea	4627	chr22	-	35051023	35091103	GO:000091	GO:000172	GO:000014	Hs.474751	ref:NM_002	THC24653	ENST00000216181
BC042090	-9.411014	-3.23435	9.4110143	down	2.408731	1.2682733	2.408731	up	1122.5408	119.27947	287.31216	MBNL3	CHCR|FLJ1	muscleblind	55796	chrX	-	131340912	131451676	GO:000727	GO:000563	GO:000367	Hs.105134	ref:NM_133	THC26075	ENST00000370839
BC096105	-20.65973	-4.36875	20.659732	down	2.6642116	1.4137086	2.6642116	up	2289.0737	110.79881	295.19147	EPB41	4.1R|EL1|H	erythrocyte	2035	chr1	+	29186802	29316493	GO:000801	GO:000563	GO:000377	Hs.175437	ref:NM_004	THC247792	ENST00000373800

SEQ_ID	Fold chang	Log Fold c	Absolute F	Regulation(	Fold chang	Log Fold c	Absolute F	Regulation(	[AE-1](nor	[AE-3](nor	[AE-10](no	GENE_NA	SYNONYM	DESCRIPT	NCBI_GEN	CHROMO	STRAND	START	STOP	GO biologi	GO cellula	GO molecu	UniGene	Dbid	TIGRID	EnsemblID
BC108307	2.0019801	1.0014277	2.0019801	up	-2.575332	-1.364758	2.575332	down	257.52875	515.56744	200.19455	NUFIP2	182-FIP|82-	nuclear frag	57532	chr17	-	24614493	24645249		GO:000563	GO:000372	Hs.462598	ref:NM_020	THC24965	ENST00000225388
NM_20717	2.0066398	1.0047817	2.0066398	up	-2.591496	-1.373785	2.5914956	down	507.83917	1019.0503	393.22864	GPR154	GPRA|NPS	G protein-c	387129	chr7	+	34664421	34856115	GO:000716	GO:001602	GO:000158	Hs.652373	ref:NM_207	NP662978	ENST00000359791
BC001420	2.0148197	1.0106508	2.0148197	up	-2.328984	-1.219701	2.3289842	down	455.84674	918.449	394.35605	HN1	ARM2|HN1	hematologic	51155	chr17	-	70643674	70662191		GO:0005634(nucleus)		Hs.532803	ref:NM_001	THC24819	ENST00000356033
AB208963	2.0163825	1.0117693	2.0163825	up	-2.093215	-1.06572	2.093215	down	881.03076	1776.495	848.6921	MAP3K2	MEKK2|MI	mitogen-act	10746	chr2	-	127779992	127813097	GO:000646	GO:000563	GO:000016	Hs.145605	ref:NM_006	THC260171	ENST00000344908
BC015714	2.0178866	1.0128451	2.0178866	up	-2.387038	-1.255221	2.3870377	down	1572.6047	3173.338	1329.4042	PHF21A	BHC80|BM	PHD finger	51317	chr11	-	45911610	46099252	GO:000635	GO:000011	GO:000551	Hs.502458	ref:NM_016	THC24618	ENST00000323180
J03619	2.0187964	1.0134954	2.0187964	up	-2.131546	-1.0919	2.1315455	down	3529.4946	7125.331	3342.8003	FCGR2A	CD32|CD32	Fc fragment	2212	chr1	+	159741884	159755891	GO:000695	GO:000588	GO:000487	Hs.352642	ref:NM_021	THC26080	ENST00000367972
BC058903	2.0189806	1.0136271	2.0189806	up	-2.441867	-1.287985	2.4418672	down	13442.197	27139.535	11114.255	ICAM3	CD50|CDW	intercellular	3385	chr19	-	10305453	10311296	GO:001633	GO:000588	GO:000517	Hs.654563	ref:NM_002	NP1133830	ENST00000160262
NM_00556	2.0191167	1.0137243	2.0191167	up	-2.493922	-1.318417	2.4939224	down	899.6928	1816.5848	728.4047	LIMK2	-	LIM domai	3985	chr22	+	29938249	30006066	GO:000646	GO:000563	GO:000016	Hs.474596	ref:NM_016	THC24796	ENST00000340552
NM_01526	2.0215981	1.0154962	2.0215981	up	-2.251635	-1.170973	2.2516346	down	2159.2734	4365.183	1938.6729	SLC9A8	DKFZp686	solute carrie	23315	chr20	+	47862656	47942179	GO:000681	GO:001602	GO:001529	Hs.444202	ref:NM_015	THC26067	ENST00000361573
BC104796	2.0234377	1.0168084	2.0234377	up	-2.732042	-1.44998	2.7320421	down	935.78314	1893.4989	693.0709	MCEMP1	-	mast cell-ex	199675	chr19	+	7647942	7650069		GO:0016020(membrane		Hs.432413	ref:NM_174	THC247547	ENST00000333598
AJ291579	2.0275672	1.0197497	2.0275672	up	-2.935558	-1.553635	2.9355578	down	915.51636	1856.2709	632.3401	KIF13A	bA500C11.2	kinesin fami	63971	chr6	-	17868565	18095673	GO:000701	GO:000587	GO:000016	Hs.189915	ref:NM_022	THC24908	ENST00000259711
NM_00235	2.0290822	1.0208273	2.0290822	up	-2.718179	-1.44264	2.7181787	down	810.64886	1644.8732	605.138	MAFG	MGC13090	v-maf musc	4097	chr17	-	77469437	77478879	GO:000170	GO:000078	GO:000370	Hs.252229	ref:NM_002	THC248237	ENST00000357736
NM_00182	2.0314965	1.0225429	2.0314965	up	-4.831039	-2.272334	4.8310391	down	497.18362	1010.0268	209.0703	CLC	LGALS10|L	Charcot-Ley	1178	chr19	-	44913734	44920508	GO:0006644(phospholip		GO:000462	Hs.889	ref:NM_001	THC247302	ENST00000221804
NM_03254	2.0330965	1.0236787	2.0330965	up	-2.52732	-1.337608	2.5273199	down	6332.2183	12874.011	5093.938	ABTB1	BPOZ|EF1A	ankyrin rep	80325	chr3	+	128874470	128882453	GO:0006412(translation		GO:000374	Hs.107812	ref:NM_172	NP852599	ENST00000232744
NM_00039	2.0355051	1.0253868	2.0355051	up	-2.254943	-1.173091	2.254943	down	1722.2504	3505.6494	1554.651	CSF2RB	CD131|CDw	colony stim	1439	chr22	+	35648167	35664764	GO:000716	GO:001602	GO:000487	Hs.592192	ref:NM_000	THC24868	ENST00000262825
BC087841	2.0393329	1.0280973	2.0393329	up	-2.528485	-1.338273	2.5284849	down	6338.663	12926.644	5112.407	IGHA1	FLJ14473|F	immunoglol	3493	chr14	-	105244505	105845571		GO:004261	GO:000551	Hs.648398	gb:AK1284	NP852642	ENST00000390547
BC067806	2.0434781	1.0310268	2.0434781	up	-2.542964	-1.346511	2.5429638	down	2361.9597	4826.613	1898.0266	PDLIM7	-	PDZ and LI	9260	chr5	-	176843004	176857208	GO:000150	GO:000585	GO:000551	Hs.533040	ref:NM_005	THC26543	ENST00000355841
AY313927	2.0535184	1.0380979	2.0535184	up	-3.289577	-1.717902	3.2895773	down	168.8652	346.7678	105.41409	ATF3	-	activating tr	467	chr1	+	210854986	210859216	GO:000635	GO:000563	GO:000367	Hs.460	ref:NM_004	NP1205356	ENST00000366987
AK125394	2.0623262	1.0442725	2.0623262	up	-2.163911	-1.113641	2.1639106	down	756.20294	1559.5371	720.7031	N/A	N/A	Homo sapie	N/A	chr2	+	201742306	201744647							
AY212247	2.064181	1.0455695	2.064181	up	-3.022236	-1.595616	3.0222359	down	1693.4055	3495.4954	1156.5925	MAP4K4	FLH21957|I	mitogen-act	9448	chr2	+	101680974	101874215	GO:000646	GO:000557	GO:000016	Hs.431550	ref:NM_145	THC25029	ENST00000324219
XM_92782	2.0724895	1.0513648	2.0724895	up	-3.570217	-1.836012	3.5702174	down	2415.4229	5005.9385	1402.1383	LOC644731	-	similar to Ig	644731	chr9	-	69634182	69636905							
NM_01456	2.0732495	1.0518938	2.0732495	up	-4.09334	-2.033279	4.09334	down	217.6062	451.15195	110.2161	OTX1	FLJ38361|M	orthodenticl	5013	chr2	+	63131468	63137818	GO:000635	GO:000563	GO:000370	Hs.445340	ref:NM_014	THC247932	ENST00000282549
BC007564	2.0828919	1.0585879	2.0828919	up	-2.197048	-1.135567	2.1970483	down	1993.9169	4153.1133	1890.315	ANXA11	ANX11|CA	annexin A1	311	chr10	-	81905333	81955094	GO:000695	GO:000563	GO:000550	Hs.530291	ref:NM_145	THC248592	ENST00000360615
AB011163	2.082914	1.0586033	2.082914	up	-2.546681	-1.348618	2.5466814	down	267.70987	557.61664	218.95815	KIF1B	CMT2|CMT	kinesin fami	23095	chr1	+	10194260	10360582	GO:000701	GO:000573	GO:000016	Hs.97858	ref:NM_015	THC24921	ENST00000263934
AK125892	2.0858338	1.0606242	2.0858338	up	-4.876512	-2.28585	4.8765122	down	655.4519	1367.1637	280.35687	N/A	N/A	Homo sapie	N/A	chr6	-	39957557	39964685							
BC020494	2.0878119	1.0619918	2.0878119	up	-2.429722	-1.280791	2.4297221	down	321.72202	671.69507	276.44934	BLOC1S2	BLOS2|FLJ	biogenesis c	282991	chr10	-	102024452	102035939			GO:004280	Hs.576605	ref:NM_001	THC24674	ENST00000358848
XM_93228	2.0883087	1.062335	2.0883087	up	-2.282384	-1.190542	2.2823843	down	178.27252	372.28806	163.11366	LOC64464	-	hypothetical	644649	chr16	-	58329336	58346796							
NM_03287	2.0885986	1.0625353	2.0885986	up	-2.021843	-1.015671	2.0218429	down	2430.427	5076.1865	2510.673	SYTL1	FLJ14996|J	synaptotagn	84958	chr1	+	27541099	27553008	GO:000688	GO:000562	GO:000521	Hs.469175	ref:NM_032	THC27844	ENST00000318074
XM_93208	2.0896413	1.0632553	2.0896413	up	-2.577906	-1.366199	2.5779056	down	1940.909	4055.8037	1573.2941	LOC64278	-	hypothetical	642788	chr16	+	88751709	88767182							
NM_08059	2.0938544	1.0661611	2.0938544	up	-2.539534	-1.344564	2.5395336	down	577.7682	1209.7625	476.37192	HIST1H2BI	H2B/S|H2B	histone 1, H	85236	chr6	-	27214051	27222598	GO:000633	GO:000078	GO:000367	Hs.437275	ref:NM_080	THC2763449	
NM_05816	2.0985005	1.0693588	2.0985005	up	-2.169613	-1.117438	2.1696131	down	117.55204	246.68301	113.69908	LOH12CR1	LOH1CR12	loss of heter	118426	chr12	+	12401321	12511105				Hs.504805	ref:NM_058	THC26208	ENST00000314565
NM_00090	2.1017865	1.0716161	2.1017865	up	-2.845019	-1.508438	2.8450186	down	2445.0022	5138.8726	1806.2703	NQO2	DHQV|DIA	NAD(P)H d	4835	chr6	+	2945229	2964993	GO:0006118(electron tra		GO:000827	Hs.533050	ref:NM_000	THC26130	ENST00000380455
NM_00515	2.1018894	1.0716867	2.1018894	up	-2.809311	-1.490216	2.8093109	down	592.41077	1245.1819	443.23392	ROD1	DKFZp781	ROD1 regul	9991	chr9	-	114020535	114135733	GO:000639	GO:000563	GO:000016	Hs.269988	ref:NM_005	THC25682	ENST00000374255
NM_14468	2.1021825	1.0718879	2.1021825	up	-2.576412	-1.365363	2.5764117	down	555.50415	1167.7711	453.25485	MGC23280	-	hypothetical	147015	chr17	-	24248927	24254089	GO:0008152(metabolic		GO:001649	Hs.631760	ref:NM_144	THC246331	ENST00000291979
NM_01235	2.1041915	1.073266	2.1041915	up	-2.17142	-1.118639	2.1714196	down	120.59847	253.76227	116.86469	OR10J1	HGMP07J|I	olfactory re	26476	chr1	+	157676172	157677135	GO:000716	GO:000588	GO:000487	Hs.532661	ref:NM_012	THC24843	ENST00000302444
AY862139	2.1049901	1.0738135	2.1049901	up	-4.988689	-2.318661	4.988689	down	1072.0265	2256.6052	452.34433	CMTM2	CKLFSF2|M	CKLF-like	146225	chr16	+	65170851	65179676	GO:000693	GO:000561	GO:000512	Hs.195685	ref:NM_144	NP1400323	ENST00000268595
BC001268	2.1064056	1.0747833	2.1064056	up	-2.112896	-1.079222	2.1128959	down	921.4354	1940.9167	918.605	NMI	-	N-myc (and	9111	chr2	-	151835228	151854396	GO:000636	GO:000573	GO:000371	Hs.54483	ref:NM_004	THC24628	ENST00000243346
XM_93300	2.1071076	1.075264	2.1071076	up	-2.092533	-1.06525	2.092533	down	107.33771	226.1721	108.08532	LOC65364	-	similar to ne	653648	chr9	+	86473546	86475527							
BC008309	2.1081135	1.0759525	2.1081135	up	-2.232444	-1.158624	2.2324436	down	7306.896	15403.766	6899.9575	LITAF	CMT1C|FL	lipopolysac	9516	chr16	-	11549956	11588263	GO:0006350(transcriptio		GO:000370	Hs.459940	ref:NM_004	THC25799	ENST00000339430
NM_01759	2.1174836	1.0823508	2.1174836	up	-2.853666	-1.512817	2.8536662	down	1800.0947	3811.671	1335.7102	KIF21B	FLJ16314	kinesin fami	23046	chr1	-	199205142	199259451	GO:000701	GO:000587	GO:000016	Hs.169182	ref:NM_017	THC24632	ENST00000332129
NM_00060	2.1207952	1.0846053	2.1207952	up	-5.256739	-2.394168	5.2567386	down	2521.694	5347.9966	1017.3602	ORM2	AGP-B|AG	orosomucoi	5005	chr9	+	116131889	116135357	GO:000695	GO:000561	GO:000548	Hs.522356	ref:NM_000	THC256281	ENST00000374100
NM_00115	2.1225229	1.0857801	2.1225229	up	-2.544026	-1.347114	2.5440264	down	2618.3835	5557.579	2184.5603	ANXA11	ANX11|CA	annexin A1	311	chr10	-	81904859	81955308	GO:000695	GO:000563	GO:000550	Hs.530291	ref:NM_145	THC248592	ENST00000360615
NM_03228	2.1256751	1.0879211	2.1256751	up	-2.120586	-1.084463	2.1205862	down	6816.0728	14488.756	6832.4297	ZDHHC18	DKFZp667	zinc finger,	84243	chr1	+	27025787	27054795		GO:001602	GO:000827	Hs.523710	ref:NM_032	THC246142	ENST00000374142
BC001093	2.1288011	1.0900412	2.1288011	up	-2.672774	-1.418338	2.6727739	down	2353.7612	5010.6895	1874.7151	PDLIM7	-	PDZ and LI	9260	chr5	-	176843000	176857190	GO:000150	GO:000585	GO:000551	Hs.533040	ref:NM_005	THC26543	ENST00000355841
NM_01864	2.1300623	1.0908956	2.1300623	up	-2.056888	-1.040463	2.0568883	down	3091.1714	6584.3877	3201.1401	TREM1	TREM-1	triggering re	54210	chr6	-	41351689	41362435	GO:000695	GO:000588	GO:000487	Hs.283022	ref:NM_018	THC26070	ENST00000244709
BC063705	2.1341876	1.093687	2.1341876	up	-3.123608	-1.643213	3.1236077	down	1580.6725	3373.4517	1079.9857	TMEM91	FLJ27310	transmembr	641649	chr19	+	46575036	46581822							

NM_20749	2.135489	1.0945664	2.135489	up	-2.039067	-1.027909	2.0390671	down	200.91867	429.0596	210.41956	FLJ43093	-	FLJ43093 p	401258	chr6	+	36773605	36808938							
NM_02099	2.1402953	1.0978099	2.1402953	up	-2.059895	-1.042571	2.0598954	down	3354.0068	7178.565	3484.9172	HPR	A-259H10.2	haptoglobin	3250	chr16	+	70654625	70668646	GO:000650	GO:000557	GO:000425	Hs.655361	ref:NM_020	THC25577	ENST00000228226
NM_00509	2.1414613	1.0985956	2.1414613	up	-5.81381	-2.539484	5.8138102	down	1350.0963	2891.179	497.29504	PGLYRP1	MGC12689	peptidoglyc	8993	chr19	-	51214280	51218163	GO:000925	GO:000557	GO:000551	Hs.137583	ref:NM_005	THC24797	ENST00000008938
NM_00686	2.1443867	1.1005651	2.1443867	up	-2.053157	-1.037844	2.0531567	down	4238.7275	9089.471	4427.0713	LILRB3	CD85A|HL	leukocyte in	11025	chr19	-	59412548	59418709	GO:000695	GO:000588	GO:000488	Hs.631592	ref:NM_006	NP322453	ENST00000245620
NM_13442	2.1451536	1.101081	2.1451536	up	-2.20434	-1.140347	2.2043398	down	562.6136	1206.8926	547.5075	SLC26A6	DKFZp586	solute carrie	65010	chr3	-	48638159	48647930	GO:000681	GO:001602	GO:000493	Hs.663208	gb:AF1613	THC2603989	
BC009778	2.1478142	1.1028692	2.1478142	up	-7.383833	-2.88437	7.3838329	down	421.1335	904.5165	122.49959	DUSP13	BEDP|FLJ3	dual specifi	51207	chr10	-	76524204	76538860	GO:0006470(protein am		GO:000472	Hs.178170	ref:NM_001	THC24750	ENST00000330673
NM_00229	2.1578525	1.1095963	2.1578525	up	-2.96423	-1.567658	2.9642303	down	854.2378	1843.3192	621.85425	LBR	DHCR14B|	lamin B rec	3930	chr1	-	223655826	223682407		GO:000563	GO:000367	Hs.435166	ref:NM_002	THC246891	ENST00000338179
XM_29084	2.1604016	1.1112995	2.1604016	up	-2.657299	-1.409961	2.6572992	down	3007.003	6496.334	2444.713	LRFN1	KIAA1484|	leucine rich	57622	chr19	-	44489053	44497816			GO:000551	Hs.97860	gb:BC0146	THC2677516	
AY220121	2.1612528	1.1118679	2.1612528	up	-3.254662	-1.702508	3.2546624	down	496.89545	1073.9167	329.96255	SERPINA9	GCET1|MG	serpin pepti	327657	chr14	-	93998812	94012332			GO:000486	Hs.663300	ref:NM_175	NP651814	ENST00000380365
NM_00103	2.170146	1.1177921	2.170146	up	-2.061983	-1.044033	2.0619833	down	116.07991	251.91035	122.16896	FLJ40244	MGC13821	hypothetical	254272	chr17	-	18480440	18485746							
J03925	2.1706842	1.1181498	2.1706842	up	-3.794458	-1.923894	3.7944584	down	2734.1538	5934.9844	1564.119	ITGAM	CD11B|CR	integrin, alp	3684	chr16	+	31178821	31251690	GO:000715	GO:000563	GO:000028	Hs.172631	ref:NM_000	NP1472148	
BC055089	2.1730587	1.1197271	2.1730587	up	-11.01232	-3.461047	11.012325	down	2846.8486	6186.369	561.76776	CAMP	CAP18|FAL	cathelicidin	820	chr3	+	48239965	48241977	GO:004274	GO:0005576(extracellul		Hs.51120	ref:NM_004	THC24730	ENST00000296435
NM_00173	2.1815288	1.1253395	2.1815288	up	-4.683812	-2.227683	4.6838117	down	2162.3586	4717.2476	1007.1386	C5AR1	C5A|C5AR|	complement	728	chr19	+	52504970	52517173	GO:000018	GO:000588	GO:000158	Hs.2161	ref:NM_001	THC24733	ENST00000355085
NM_01805	2.1901158	1.1310071	2.1901158	up	-2.17399	-1.120345	2.1739897	down	1851.4927	4054.9834	1865.2266	XKR8	FLJ10307|R	XK, Kell bl	55113	chr1	+	28159090	28167191		GO:0016020(membrane		Hs.55024	ref:NM_018	THC251654	ENST00000373884
XM_93208	2.1944313	1.1338471	2.1944313	up	-2.038926	-1.027809	2.0389256	down	690.4772	1515.2048	743.13885	LOC339287	DKFZp686J	hypothetical	339287	chr17	+	35532111	35540248				Hs.532786	gb:CR7493	THC262902	ENST00000339569
BC070085	2.1948217	1.1341037	2.1948217	up	-2.022213	-1.015935	2.0222129	down	1716.117	3766.5708	1862.5985	CSF2RB	CD131|CDw	colony stim	1439	chr22	+	35639620	35666423	GO:000716	GO:001602	GO:000487	Hs.592192	ref:NM_000	THC24868	ENST00000262825
BC019931	2.1967273	1.1353558	2.1967273	up	-2.057759	-1.041074	2.0577587	down	4177.437	9176.69	4459.556	FCGR2A	CD32|CD32	Fc fragment	2212	chr1	+	159741843	159754971	GO:000695	GO:000588	GO:000487	Hs.352642	ref:NM_021	THC26080	ENST00000367972
BC015234	2.197923	1.1361408	2.197923	up	-2.450123	-1.292854	2.4501232	down	1460.4705	3210.0017	1310.1389	DGAT2	DKFZp686	diacylglycer	84649	chr11	+	75157436	75189325	GO:000607	GO:000562	GO:000414	Hs.334305	ref:NM_032	NP1242886	ENST00000228027
NM_02391	2.2123421	1.1455745	2.2123421	up	-3.413254	-1.771148	3.4132538	down	762.4728	1686.8507	494.20605	P2RY13	FKSG77|GI	purinergic r	53829	chr3	-	152526785	152529643				Hs.546396	ref:NM_023	THC247465	ENST00000325602
NM_01805	2.2125048	1.1456806	2.2125048	up	-6.475262	-2.694939	6.4752624	down	1322.4946	2926.0256	451.87753	MANSC1	9130403P13	MANSC do	54682	chr12	-	12373484	12394436		GO:001602	GO:000456	Hs.591145	ref:NM_018	THC247163	ENST00000355566
NM_00393	2.2172983	1.1488028	2.2172983	up	-2.141637	-1.098714	2.1416369	down	566.87836	1256.9384	586.90546	SCAP2	MGC10411	src family a	8935	chr7	-	26673211	26870866	GO:000646	GO:000573	GO:000507	Hs.200770	ref:NM_003	THC271637	ENST00000345317
AF116651	2.2211387	1.1512995	2.2211387	up	-2.914255	-1.543127	2.9142548	down	279.509	620.82825	213.03156	N/A	N/A	Homo sapie	N/A	chr15	-	92721689	92722589							
NM_00616	2.2313783	1.1579351	2.2313783	up	-2.337011	-1.224665	2.3370112	down	6427.4346	14342.038	6136.9146	NFE2	NF-E2|p45	nuclear fact	4778	chr12	-	52972161	52975811	GO:000635	GO:000563	GO:000370	Hs.75643	ref:NM_006	THC26020	ENST00000312156
NM_00336	2.2341311	1.1597138	2.2341311	up	-2.187559	-1.129322	2.1875593	down	1571.3608	3510.626	1604.8141	UPP1	UDRPASE|	uridine phos	7378	chr7	+	48095270	48114855	GO:000911	GO:000573	GO:000485	Hs.488240	gb:BC0470	THC26091	ENST00000341253
NM_00288	2.2374268	1.1618405	2.2374268	up	-2.806131	-1.488583	2.8061315	down	914.09766	2045.2266	728.84204	RALB	-	v-ral simian	5899	chr2	+	120726883	120768756	GO:000716	GO:000562	GO:000016	Hs.469820	ref:NM_002	THC26167	ENST00000272519
AK127293	2.2438053	1.1659475	2.2438053	up	-2.474854	-1.307343	2.4748541	down	117.07163	262.68594	106.14199	N/A	N/A	Homo sapie	N/A	chr3	+	196960854	196962472							
BC021987	2.2460588	1.1673957	2.2460588	up	-2.279924	-1.188986	2.2799243	down	1051.6721	2362.1174	1036.0508	NMI	-	N-myc (and	9111	chr2	-	151835233	151847714	GO:000636	GO:000573	GO:000371	Hs.54483	ref:NM_004	THC24628	ENST00000243346
NM_03249	2.2576814	1.1748419	2.2576814	up	-2.894964	-1.533545	2.894964	down	3709.632	8375.167	2893.0125	ARHGAP9	10C|FLJ165	Rho GTPas	64333	chr12	-	56152313	56168864	GO:000716	GO:000562	GO:000509	Hs.437126	ref:NM_032	THC24839	ENST00000356411
BC032708	2.2591131	1.1757565	2.2591131	up	-2.112728	-1.079107	2.1127284	down	814.9827	1841.1381	871.45044	TBL1X	EBI|TBL1	transducin (	6907	chrX	+	9391368	9646902	GO:000635	GO:0005634(nucleus)		Hs.495656	ref:NM_005	THC25149	ENST00000217964
BC063855	2.260991	1.1769553	2.260991	up	-2.006576	-1.004736	2.0065763	down	524.718	1186.3827	591.24725	ATF7IP	FLJ10139|F	activating tr	55729	chr12	+	14409893	14526291	GO:000635	GO:0005634(nucleus)		Hs.591151	ref:NM_018	THC247291	ENST00000261168
NM_02274	2.2621913	1.177721	2.2621913	up	-2.596106	-1.376349	2.5961062	down	1890.7848	4277.317	1647.5894	MOSC1	FLJ22390|R	MOCO sulp	64757	chr1	+	219026893	219054353		GO:000573	GO:001649	Hs.497816	ref:NM_022	THC27452	ENST00000366910
AF096300	2.2627971	1.1781072	2.2627971	up	-2.310502	-1.208206	2.3105017	down	1466.4161	3318.2021	1436.1392	MAP4K4	FLH21957|I	mitogen-act	9448	chr2	+	101680919	101874388	GO:000646	GO:000557	GO:000016	Hs.431550	ref:NM_145	THC25029	ENST00000324219
NM_15268	2.2654327	1.1797867	2.2654327	up	-2.46088	-1.299174	2.4608799	down	239.52283	542.62286	220.49953	FLJ33641	MGC70478	hypothetical	202309	chr5	+	57823086	57827942		GO:0016020(membrane		Hs.547697	ref:NM_152	THC24762	ENST00000318469
BC041387	2.2726445	1.184372	2.2726445	up	-2.719464	-1.443322	2.7194639	down	730.20483	1659.496	610.2291	MCTP2	FLJ11175|F	multiple C2	55784	chr15	+	92575958	92824636	GO:001972	GO:000562	GO:000550	Hs.592017	gb:BC0413	THC24755	ENST00000357742
BC111860	2.2776357	1.187537	2.2776357	up	-2.128098	-1.089565	2.1280983	down	142.79935	325.2449	152.83359	KCNK16	MGC13312	potassium c	83795	chr6	-	39390575	39398294	GO:000681	GO:001602	GO:000521	Hs.287765	ref:NM_032	NP856904	
NM_00170	2.2776571	1.1875506	2.2776571	up	-2.448511	-1.291904	2.4485106	down	1040.7872	2370.5564	968.1626	BCL6	BCL5|BCL6	B-cell CLL/	604	chr3	-	188921858	188946169	GO:000012	GO:000562	GO:000367	Hs.478588	ref:NM_138	THC24665	ENST00000232014
NM_01219	2.2776615	1.1875533	2.2776615	up	-6.351373	-2.667068	6.3513729	down	1379.1171	3141.1619	494.56424	GCA	GCL	grancalcin,	25801	chr2	+	162908895	162925875	GO:000694	GO:000573	GO:000550	Hs.377894	ref:NM_012	THC26213	ENST00000233612
NM_03215	2.2827487	1.1907721	2.2827487	up	-2.027369	-1.019608	2.0273687	down	2158.2288	4926.694	2430.0928	PRAM1	MGC39864	PML-RARA	84106	chr19	-	8460939	8473495	GO:0007229(integrin-m		GO:000828	Hs.465812	ref:NM_032	THC249927	ENST00000255612
NM_01517	2.286854	1.1933643	2.286854	up	-2.722122	-1.444732	2.7221221	down	4665.875	10670.175	3919.8003	XPO6	EXP6|FLJ2	exportin 6	23214	chr16	-	28016800	28099853	GO:000005	GO:000563	GO:000551	Hs.460468	ref:NM_015	THC246994	ENST00000304658
NM_02069	2.2910815	1.1960288	2.2910815	up	-2.355844	-1.236244	2.3558444	down	332.05392	760.7626	322.92566	TMCC3	KIAA1145	transmembr	57458	chr12	-	93486167	93568455		GO:0016020(membrane		Hs.370410	ref:NM_020	THC27076	ENST00000261226
BC060775	2.3022172	1.2030239	2.3022172	up	-3.596035	-1.846407	3.5960347	down	773.33655	1780.3887	495.09775	FLJ32028	-	hypothetical	201799	chr4	-	153767426	153820539		GO:0016020(membrane		Hs.518900	ref:NM_152	THC24711	ENST00000304385
AK131381	2.3047175	1.2045899	2.3047175	up	-2.174648	-1.120782	2.174648	down	406.56042	937.0069	430.8775	CCNJL	FLJ14166	cyclin J-like	79616	chr5	-	159611237	159699177	GO:000007	GO:0005634(nucleus)		Hs.14070	ref:NM_024	THC26069	ENST00000257536
M59819	2.3087902	1.2071371	2.3087902	up	-2.80842	-1.489758	2.8084196	down	4714.894	10885.701	3876.095	CSF3R	CD114|GCS	colony stim	1441	chr1	-	36704281	36721087	GO:000695	GO:000588	GO:000487	Hs.524517	ref:NM_172	NP096413	ENST00000331941
NM_00562	2.3097259	1.2077217	2.3097259	up	-3.393539	-1.762791	3.3935393	down	3165.0732	7310.4517	2154.2263	S100A12	CAAF1|CG	S100 calciu	6283	chr1	-	151612807	151614699	GO:000680	GO:000562	GO:000550	Hs.19413	ref:NM_005	THC247372	ENST00000368737
NM_18270	2.3136668	1.2101811	2.3136668	up	-2.828001	-1.499782	2.8280007	down	3669.9827	8491.117	3002.5159	FAM101B	MGC45871	family with	359845	chr17	-	293026	295959				Hs.591203	ref:NM_182	THC2497865	
NM_17364	2.3137083	1.210207	2.3137083	up	-2.228115	-1.155824	2.228115	down	1734.0349	4012.051	1800.6481	RNF149	DNAPTP2|I	ring finger p	284996	chr2	-	101258984	101291584	GO:000651	GO:001602	GO:000551	Hs.142074	ref:NM_173	THC25350	ENST00000295317
NM_00102	2.3181845	1.2129954	2.3181845	up	-3.035563	-1.601964	3.0355627	down	2330.316	5402.1025	1779.605	SOD2	IPO-B|MNS	superoxide	6648	chr6	-	160020137	160034343	GO:000030	GO:000573	GO:000478	Hs.487046	ref:NM_000	THC25669	ENST00000367054
BC005009	2.3224195	1.2156286	2.3224195	up	-2.850928	-1.511432	2.8509282	down	10585.078	24582.992	8622.803	YPEL3	MGC10500	yippee-like	83719	chr16	-	30011135	30014994				Hs.513491	ref:NM_031	THC250424	ENST00000204279
AK026656	2.3230705	1.2160329	2.3230705	up	-2.840251	-1.506019	2.8402515	down	517.8337	1202.9642	423.54144	N/A	N/A	Homo sapie	N/A	chr5	+	10516282	10518137							
NM_00210	2.3260893	1.2179065	2.3260893	up	-2.931587	-1.551682	2.9315874	down	214.1149	498.05038	169.89102	HAL	HIS|HSTD|	histidine am	3034	chr12	-	94891272	94914202	GO:000654	GO:000573	GO:000439	Hs.190783	ref:NM_002	THC247544	ENST00000261208
XM_92683	2.327342	1.2186832	2.327342	up	-2.525618	-1.336637	2.5256183	down	245.43442	571.20984	226.16634	LOC643518	-	similar to In	643518	chr4	+	81220985	81221300							
BC020823	2.3318756	1.2214908	2.3318756	up	-2.003906	-1.002815	2.0039064	down	4258.8486	9931.105	4955.8726	FCGR2A	CD32|CD32	Fc fragment	2212	chr1	+	159741878	159754973	GO:000695	GO:000588	GO:000487	Hs.352642	ref:NM_021	THC26080	ENST00000367972
AY465895	2.3361201	1.2241144	2.3361201	up	-2.12052	-1.084418	2.1205201	down	294.73697	688.54095	324.7038	HCRP1	-	hepatocellul	387535	chr6	+	42434508	42436487							
NM_01241	2.3431215	1.2284318	2.3431215	up	-5.813484	-2.539403	5.8134838	down	2119.5781	4966.429	854.2948	QPCT	GCT|QC	glutaminyl-	25797	chr2	+	37425256	37453969	GO:0006464(protein mo		GO:000823	Hs.79033	ref:NM_012	THC26029	ENST00000338415
AY358390	2.3445886	1.2293348	2.3445886	up	-2.3625	-1.240314	2.3625001	down	749.45294	1757.1588	743.7709	DPEP3	MBD3	dipeptidase	64180	chr16	-	66567067	66571876	GO:000650	GO:000562	GO:000423	Hs.302028	ref:NM_022	THC24720	ENST00000268793
NM_17352	2.3522815	1.2340607	2.3522815	up	-3.448676	-1.786043	3.4486762	down	241.4643	567.992	164.69856	FLJ38964	-	hypothetical	161253	chr14	+	22422280	22426729	GO:0007264(small GTP		GO:000016	Hs.444911	ref:NM_173	THC247734	ENST00000267396
NM_00087	2.3556917	1.2361508	2.3556917	up	-2.572686	-1.363276	2.5726863	down	5035.36	11861.756	4610.65	IGF2R	CD222|CIM	insulin-like	3482	chr6	+	160310120	160447573	GO:000681	GO:000563	GO:000487	Hs.487062	ref:NM_000	THC24689	ENST00000356956
NM_03338	2.3575533	1.2372904	2.3575533	up	-2.571425	-1.362568	2.5714247	down	4955.4497	11682.737	4543.2935	ATG16L2	FLJ00012|W	ATG16 autc	89849	chr11	+	72203098	72218328				Hs.653186	ref:NM_033	THC249467	ENST00000321297
XM_93118	2.3584348	1.2378297	2.3584348	up	-2.37263	-1.246487	2.3726299	down	184.87648	436.01913	183.77039	LOC642948	-	hypothetical	642948	chr19	-	35408641	35411844							
BC036723	2.3622193	1.2401429	2.3622193	up	-2.082534	-1.05834	2.0825339	down	13762.482	32510	15610.79	FCGR3A	CD16|CD16	Fc fragment	2214	chr1	-	159778174	159786442	GO:000695	GO:000588	GO:000487	Hs.372679	ref:NM_000	THC27327	ENST00000367969
XM_93035	2.3633945	1.2408605	2.3633945	up	-2.252096	-1.171268	2.2520956	down	115.38972	272.71143	121.0923	C14orf144	-	chromosom	145195	chr14	+	103775311	103780535				Hs.679805	gb:BC007749		
NM_00101	2.3658759	1.2423744	2.3658759	up	-3.584915	-1.841939	3.584915	down	1087.6049	2573.1382	717.76825	GNG10	-	guanine nuc	2790	chr9	+	113454835	113472328							
BC017865	2.378367	1.2499714	2.378367	up	-2.974329	-1.572564	2.974329	down	11405.807	27127.195	9120.442	FCGR3A	CD16|CD16	Fc fragment	2214	chr1	-	159779142	159786158	GO:000695	GO:000588	GO:000487	Hs.372679	ref:NM_000	THC27327	ENST00000367969
CR749360	2.3793059	1.2505408	2.3793059	up	-3.678605	-1.879159	3.6786054	down	3375.852	8032.1846	2183.4863	LOC339287	DKFZp686J	hypothetical	339287	chr17	+	35531742	35546566				Hs.532786	gb:CR7493	THC262902	ENST00000339569
NM_00056	2.3797902	1.2508344	2.3797902	up	-2.489357	-1.315773	2.4893567	down	990.4443	2357.0496	946.8509	IL6R	CD126|IL-6	interleukin	3570	chr1	+	152644292	152706812	GO:000695	GO:000557	GO:000487	Hs.591492	ref:NM_000	THC24647	ENST00000344086
NM_00060	2.3876593	1.255597	2.3876593	up	-6.479951	-2.695983	6.4799506	down	3518.053	8399.912	1296.2926	ORM1	AGP-A|AG	orosomucoi	5004	chr9	+	116125156	116128578	GO:000695	GO:000557	GO:000551	Hs.567311	ref:NM_000	THC25587	ENST00000259396
BC035023	2.398004	1.2618341	2.398004	up	-2.085359	-1.060296	2.0853594	down	557.2994	1336.4062	640.85175	OSCAR	MGC33613	osteoclast-a	126014	chr19	-	59289813	59297806			GO:000487	Hs.347655	ref:NM_130	NP957263	ENST00000356532
BC092440	2.401121	1.2637081	2.401121	up	-2.652261	-1.407223	2.652261	down	693.7612	1665.8046	628.06964	SVIL	DKFZp686	supervillin	6840	chr10	-	29786284	29791240	GO:000701	GO:000563	GO:000550	Hs.499209	ref:NM_021	THC25582	ENST00000355867
BC019355	2.401283	1.2638054	2.401283	up	-2.13578	-1.094763	2.1357797	down	1364.355	3276.2024	1533.9608	RNF149	DNAPTP2|I	ring finger p	284996	chr2	-	101259431	101291561	GO:000651	GO:001602	GO:000551	Hs.142074	ref:NM_173	THC25350	ENST00000295317
NM_01693	2.4045406	1.2657613	2.4045406	up	-2.861011	-1.516525	2.8610113	down	974.2945	2342.7307	818.84705	UBN1	VT|VT4	ubinuclein 1	29855	chr16	+	4842216	4872364	GO:000635	GO:000563	GO:000370	Hs.440219	ref:NM_016	THC24658	ENST00000262376
XM_92924	2.4084844	1.2681256	2.4084844	up	-4.028084	-2.010094	4.0280841	down	2018.0969	4860.555	1206.6667	LOC646309	-	similar to N	646309	chr10	-	36850652	36854135							
NM_00486	2.4118459	1.2701377	2.4118459	up	-2.7616	-1.465505	2.7616004	down	6937.447	16732.053	6058.8247	LITAF	CMT1C|FL	lipopolysac	9516	chr16	-	11549356	11587730	GO:0006350(transcriptic		GO:000370	Hs.459940	ref:NM_004	THC25799	ENST00000339430
NM_00660	2.4139347	1.2713866	2.4139347	up	-2.49254	-1.317616	2.4925396	down	184.4401	445.22635	178.62358	RFPL3	-	ret finger pr	10738	chr22	+	31083853	31087148			GO:000551	Hs.558455	ref:NM_006604;ens:EN		ENST00000249007
AK092439	2.4176387	1.2735987	2.4176387	up	-2.74395	-1.456254	2.7439496	down	1414.5602	3419.8955	1246.3405	MOSC1	FLJ22390|R	MOCO sulp	64757	chr1	+	219033153	219054358		GO:000573	GO:001649	Hs.497816	ref:NM_022	THC27452	ENST00000366910
NM_00103	2.4191487	1.2744994	2.4191487	up	-3.85145	-1.945402	3.8514499	down	193.00705	466.91275	121.23039	THEDC1	FLJ11106|M	thioesterase	55301	chr10	+	15125950	15155664	GO:0006633(fatty acid b		GO:000432	Hs.24309	ref:NM_018	THC247432	ENST00000378217
NM_02077	2.4398038	1.2867651	2.4398038	up	-2.281659	-1.190083	2.2816595	down	1207.8112	2946.8223	1291.5259	TBC1D14	KIAA1322	TBC1 doma	57533	chr4	+	6962472	7085739		GO:000562	GO:000509	Hs.518611	ref:NM_020	THC27320	ENST00000382560
BC016281	2.4480575	1.2916374	2.4480575	up	-3.032813	-1.600657	3.0328129	down	1113.5846	2726.1191	898.8748	BCL2A1	BCL2L5|BF	BCL2-relate	597	chr15	-	78040289	78050576	GO:000691	GO:000562	GO:000551	Hs.227817	ref:NM_004	THC24773	ENST00000267953
NM_03225	2.4500956	1.2928381	2.4500956	up	-2.123282	-1.086296	2.1232821	down	2021.9641	4954.0054	2333.1829	CCDC88	BRLZ|DKF	coiled-coil c	283234	chr11	+	63864270	63881582			GO:000551	Hs.98564	ref:NM_032	THC26041	ENST00000356786
NM_00235	2.4539924	1.2951308	2.4539924	up	-2.134337	-1.093788	2.1343373	down	3461.6704	8494.913	3980.1174	LYN	FLJ26625|J	v-yes-1 Yan	4067	chr8	+	56954925	57085685	GO:000646	GO:000563	GO:000016	Hs.491767	ref:NM_002	THC27749	ENST00000276497
NM_00065	2.4618382	1.2997359	2.4618382	up	-3.085388	-1.625452	3.0853881	down	6418.6753	15801.74	5121.4756	SELL	CD62L|LAN	selectin L (l	6402	chr1	-	167926431	167947351	GO:000692	GO:000588	GO:000551	Hs.82848	ref:NM_000	THC24688	ENST00000367783
BC025354	2.4625611	1.3001595	2.4625611	up	-5.123505	-2.357131	5.1235048	down	656.89716	1617.6494	315.73102	CMTM2	CKLFSF2|N	CKLF-like	146225	chr16	+	65170860	65179676	GO:000693	GO:000561	GO:000512	Hs.195685	ref:NM_144	NP1400323	ENST00000268595
NM_03147	2.466838	1.302663	2.466838	up	-2.250526	-1.170262	2.2505263	down	9790.416	24151.37	10731.432	YPEL3	MGC10500	yippee-like	83719	chr16	-	30011135	30015022				Hs.513491	ref:NM_031	THC250424	ENST00000204279
BC003164	2.4709426	1.3050615	2.4709426	up	-2.001151	-1.00083	2.0011508	down	5890.54	14555.186	7273.408	LENG4	BB1	leukocyte re	79143	chr19	-	59368924	59385067	GO:000815	GO:000557	GO:000367	Hs.467279	ref:NM_024	THC26239	ENST00000245615
NM_17077	2.4731681	1.3063603	2.4731681	up	-9.40487	-3.233408	9.4048699	down	8012.1763	19815.459	2106.936	GPR97	GPR-97|PG	G protein-c	222487	chr16	+	56259671	56280791	GO:000716	GO:001602	GO:000487	Hs.383403	ref:NM_170	THC24747	ENST00000333493
BC096063	2.4788081	1.3096466	2.4788081	up	-2.515641	-1.330926	2.5156408	down	5828.3394	14447.335	5743.004	LITAF	CMT1C|FL	lipopolysac	9516	chr16	-	11550739	11587648	GO:0006350(transcriptic		GO:000370	Hs.459940	ref:NM_004	THC25799	ENST00000339430
BC028221	2.4995059	1.3216429	2.4995059	up	-6.374836	-2.672388	6.3748359	down	6275.8486	15686.521	2460.694	IL8RA	C-C|C-C CK	interleukin	3577	chr2	-	218736293	218739920	GO:000693	GO:001602	GO:000158	Hs.194778	ref:NM_000	THC24669	ENST00000295683
BC013300	2.5144553	1.3302459	2.5144553	up	-6.9051	-2.787662	6.9051005	down	822.8091	2068.9167	299.62152	AURKB	AIK2|AIM-	aurora kinas	9212	chr17	-	8048781	8054625	GO:000646	GO:000077	GO:000016	Hs.442658	ref:NM_004	NP1275914	ENST00000316199
BC015220	2.5152783	1.330718	2.5152783	up	-2.556474	-1.354156	2.5564744	down	708.78253	1782.7853	697.3609	VMD2	BEST|BMD	vitelliform r	7439	chr11	+	61473931	61488515	GO:000681	GO:000562	GO:000521	Hs.693826	ref:NM_004	THC24858	ENST00000301774
XM_93196	2.5196474	1.3332219	2.5196474	up	-3.911568	-1.967747	3.9115685	down	191.23543	481.84586	123.18482	LOC643982	-	hypothetical	643982	chr17	-	76929971	76984856							
NM_03229	2.5215128	1.3342896	2.5215128	up	-2.468023	-1.303356	2.4680235	down	691.00684	1742.3826	705.983	CAMKK1	CAMKKA|	calcium/cal	84254	chr17	-	3710357	3743086	GO:000646	GO:000563	GO:000016	Hs.8417	ref:NM_032	THC24644	ENST00000348335
NM_13426	2.5227733	1.3350106	2.5227733	up	-2.441922	-1.288017	2.4419223	down	507.9016	1281.3206	524.718	SLC26A6	DKFZp586	solute carrie	65010	chr3	-	48638159	48647930	GO:000681	GO:001602	GO:000493	Hs.663208	gb:AF1613	THC2603989	

BC104818	2.5415755	1.3457231	2.5415755	up	-7.763201	-2.956652	7.7632015	down	3628.0146	9220.873	1187.7668	ORM1	AGP-A|AG	orosomucoi	5004	chr9	+	116125209	116128503	GO:000695	GO:000557	GO:000551	Hs.567311	ref:NM_000	THC255875	ENST00000259396
NM_00395	2.5460857	1.348281	2.5460857	up	-2.085285	-1.060245	2.085285	down	2099.985	5346.7417	2564.034	SOCS3	CIS3|Cish3|	suppressor c	9021	chr17	-	73864453	73867753	GO:0001558(regulation		GO:000486	Hs.527973	ref:NM_003	THC246186	ENST00000330871
NM_17202	2.5655452	1.3592654	2.5655452	up	-3.165676	-1.662514	3.1656765	down	3695.268	9480.377	2994.7397	ABTB1	BPOZ|EF1A	ankyrin repe	80325	chr3	+	128874470	128882453	GO:0006412(translation)		GO:000374	Hs.107812	ref:NM_172	NP852599	ENST00000232744
NM_01600	2.571005	1.3623324	2.571005	up	-3.497719	-1.806415	3.4977194	down	707.38763	1818.6971	519.96655	ABHD5	CDS|CGI58	abhydrolase	51099	chr3	+	43707378	43735296	GO:000650	GO:000581	GO:000417	Hs.19385	ref:NM_016	THC246405	ENST00000013894
NM_01238	2.572496	1.3631688	2.572496	up	-6.511116	-2.702905	6.5111159	down	5337.828	13731.541	2108.9382	PADI4	PAD|PADI5	peptidyl arg	23569	chr1	+	17507278	17563082	GO:000635	GO:000563	GO:000466	Hs.522969	ref:NM_012	THC248926	ENST00000375448
BC048107	2.5898415	1.3728638	2.5898415	up	-3.374525	-1.754684	3.3745249	down	570.7018	1478.0272	437.99564	ZNF333	KIAA1806	zinc finger p	84449	chr19	+	14661880	14705557	GO:000635	GO:000562	GO:000367	Hs.515215	ens:ENST00000292530;		ENST00000292530
NM_02195	2.5900005	1.3729524	2.5900005	up	-2.87579	-1.523958	2.8757897	down	799.8725	2071.6702	720.38306	HLX1	HB24	H2.0-like ho	3142	chr1	+	219119381	219125018	GO:000635	GO:000563	GO:000370	Hs.74870	ref:NM_021	THC275576	ENST00000366903
BC017773	2.597023	1.3768588	2.597023	up	-2.495384	-1.319262	2.4953836	down	2696.8176	7003.6973	2806.6616	TREM1	TREM-1	triggering re	54210	chr6	-	41351717	41362416	GO:000695	GO:000588	GO:000487	Hs.283022	ref:NM_018	THC260703	ENST00000244709
NM_00063	2.6016518	1.3794279	2.6016518	up	-4.428728	-2.146892	4.4287283	down	3004.8271	7817.5137	1765.1825	NCF4	MGC3810|N	neutrophil c	4689	chr22	+	35586990	35604005	GO:000611	GO:000582	GO:003509	Hs.474781	ref:NM_000	THC246402	ENST00000336951
NM_00634	2.604899	1.3812274	2.604899	up	-3.015082	-1.592197	3.0150822	down	724.9169	1888.3353	626.29645	TACC3	ERIC1|MGC	transforming	10460	chr4	+	1693061	1716696	GO:000007	GO:0005737(cytoplasm)		Hs.104019	ref:NM_006	THC267974	ENST00000313288
BC005051	2.607897	1.3828869	2.607897	up	-2.32974	-1.220169	2.3297402	down	1729.4148	4510.1357	1935.8964	C6orf166	FLJ10342|d	chromosome	55122	chr6	-	88441693	88468382				Hs.485915	ref:NM_018	THC256242	ENST00000257787
NM_00644	2.6113405	1.3847906	2.6113405	up	-2.014276	-1.010262	2.0142765	down	411.9567	1075.7592	534.0673	MTHFS	HsT19268	5,10-methen	10588	chr15	-	77924374	77976398	GO:000939	GO:000582	GO:000028	Hs.459049	ref:NM_006	THC270724	ENST00000258874
NM_01473	2.6123878	1.3853691	2.6123878	up	-2.973147	-1.571991	2.973147	down	2182.723	5702.119	1917.8732	RASSF2	DKFZp781	Ras associat	9770	chr20	-	4708669	4752291	GO:000704	GO:000563	GO:000551	Hs.631504	ref:NM_014	THC246845	ENST00000379400
NM_00100	2.6129408	1.3856744	2.6129408	up	-2.802053	-1.486484	2.8020528	down	132.96442	347.42816	123.99059	DUSP13	BEDP|FLJ3	dual specific	51207	chr10	-	76524195	76538976	GO:0006470(protein am		GO:000472	Hs.178170	ref:NM_001	THC247508	ENST00000330673
NM_14467	2.6234237	1.3914508	2.6234237	up	-6.349291	-2.666595	6.3492907	down	1012.8095	2657.0283	418.4764	CMTM2	CKLFSF2|N	CKLF-like M	146225	chr16	+	65170851	65179679	GO:000693	GO:000561	GO:000512	Hs.195685	ref:NM_144	NP1400323	ENST00000268595
BC093076	2.6259952	1.3928643	2.6259952	up	-3.618542	-1.855409	3.6185423	down	618.0896	1623.1003	448.55087	PPIA	CYPA|CYP	peptidylprol	5478	chr7	+	44802804	44807755	GO:0006457(protein fol		GO:000375	Hs.356331	ref:NM_021	THC256614	ENST00000355968
BC004564	2.6385442	1.3997422	2.6385442	up	-2.366543	-1.242781	2.3665425	down	7778.158	20523.014	8672.151	DOK3	FLJ22570|F	docking pro	79930	chr5	-	176861520	176869992	GO:000726	GO:000573	GO:000515	Hs.153343	ref:NM_024	THC249773	ENST00000357198
BC100942	2.641998	1.4016294	2.641998	up	-4.027571	-2.00991	4.0275712	down	245.59038	648.8493	161.10188	UNQ3033	MGC11916	LAIR hlog	284415	chr19	-	59235925	59258996							
BC029125	2.66337	1.4132528	2.66337	up	-2.783581	-1.476942	2.783581	down	2550.738	6793.559	2440.5825	FPRL1	ALXR|FML	formyl pepti	2358	chr19	+	56963709	56965589	GO:000692	GO:000588	GO:000158	Hs.99855	ref:NM_001	THC247886	ENST00000340023
NM_00178	2.6717969	1.4178104	2.6717969	up	-4.579967	-2.195337	4.5799674	down	2583.0996	6901.5176	1506.8923	CDA	CDD	cytidine dea	978	chr1	+	20771712	20817983	GO:000716	GO:000557	GO:000188	Hs.466910	ref:NM_001	NP1079190	ENST00000375071
NM_00384	2.6988776	1.4323595	2.6988776	up	-4.576495	-2.194243	4.5764955	down	3269.5173	8824.027	1928.1188	TNFRSF10	CD263|DCF	tumor necro	8794	chr8	+	23016378	23030895	GO:000691	GO:000588	GO:000488	Hs.655801	ref:NM_003	THC247160	ENST00000312659
NM_03338	2.7178446	1.442463	2.7178446	up	-2.103944	-1.073096	2.103944	down	2851.0842	7748.8037	3682.9895	SSH2	KIAA1725|	slingshot ho	85464	chr17	-	24977090	25281144	GO:000647	GO:000585	GO:000377	Hs.654754	gb:AB0723	THC2543332	
BC004380	2.7246639	1.4460783	2.7246639	up	-2.33397	-1.222786	2.33397	down	1064.1598	2899.4778	1242.2944	CKLF	C32|CKLF1	chemokine-l	51192	chr16	+	65143995	65157471	GO:000693	GO:000557	GO:000800	Hs.15159	ref:NM_016	THC250318	ENST00000264001
NM_14466	2.7282669	1.4479848	2.7282669	up	-4.300887	-2.104634	4.3008875	down	2145.9644	5854.7637	1361.292	GLT1D1	FLJ31978	glycosyltran	144423	chr12	+	127904033	128035462	GO:0009058(biosyntheti		GO:001674	Hs.655668	ref:NM_144	THC247235	ENST00000281703
NM_01534	2.7386129	1.4534453	2.7386129	up	-2.741129	-1.45477	2.7411287	down	1196.4146	3276.5164	1195.3165	DAAM2	KIAA0381|	dishevelled	23500	chr6	+	39868770	39980619	GO:0016043(cellular co		GO:000377	Hs.652207	ref:NM_015	THC249547	ENST00000274867
NM_00146	2.7393591	1.4538384	2.7393591	up	-2.459629	-1.298441	2.4596293	down	372.80374	1021.2433	415.20212	FYB	ADAP|PRO	FYN bindin	2533	chr5	-	39141113	39255424	GO:000646	GO:000563	GO:000510	Hs.370503	ref:NM_001	THC2472026	
BC104433	2.7554701	1.4622985	2.7554701	up	-3.926568	-1.973269	3.9265685	down	282.30994	777.8966	198.11105	HCG27	FLJ40123|M	HLA compl	253018	chr6	+	31273592	31278775				Hs.659818	ref:NM_181	THC248079	ENST00000376240
NM_00518	2.7557032	1.4624205	2.7557032	up	-2.321941	-1.215331	2.3219408	down	634.65375	1748.9174	753.2136	CBL	C-CBL|CBI	Cas-Br-M (r	867	chr11	+	118582199	118684069	GO:000716	GO:000563	GO:000370	Hs.504096	ref:NM_005	THC247705	ENST00000264033
NM_02246	2.7649468	1.4672517	2.7649468	up	-4.871025	-2.284225	4.8710251	down	6296.272	17408.857	3573.9617	MMP25	MMP20|MT	matrix meta	64386	chr16	+	3036682	3050728	GO:000027	GO:000557	GO:000422	Hs.654979	ref:NM_022	THC247511	ENST00000336577
BC005044	2.7771483	1.4736042	2.7771483	up	-2.463346	-1.300619	2.4633457	down	5410.5044	15025.773	6099.742	NFE2	NF-E2|p45	nuclear fact	4778	chr12	-	52972164	52981058	GO:000635	GO:000563	GO:000370	Hs.75643	ref:NM_006	THC260203	ENST00000312156
AK097259	2.8087315	1.4899187	2.8087315	up	-4.303719	-2.105584	4.3037194	down	791.29706	2222.541	516.4233	N/A	N/A	Homo sapie	N/A	chr5	-	176866807	176869989							
NM_00560	2.8124222	1.4918132	2.8124222	up	-3.764341	-1.912397	3.7643414	down	220.78503	620.94073	164.95335	PYGM	-	phosphoryla	5837	chr11	-	64270605	64284763	GO:0005975(carbohydra		GO:000016	Hs.154084	ref:NM_005	THC246641	ENST00000164139
NM_00383	2.8327772	1.5022172	2.8327772	up	-5.058708	-2.338769	5.0587078	down	1593.9141	4515.2036	892.56067	SIGLEC5	CD170|CD3	sialic acid b	8778	chr19	-	56806995	56831696	GO:000715	GO:001602	GO:000551	Hs.310333	ref:NM_003	THC2472472	
BC027951	2.8689493	1.5205225	2.8689493	up	-3.268792	-1.708758	3.2687924	down	216.57413	621.3402	190.08249	C20orf32	HEFL	chromosome	57091	chr20	+	54420770	54462337	GO:000716	GO:001602	GO:000015	Hs.473144	ref:NM_020	THC248833	ENST00000360314
AJ297858	2.8957028	1.5339135	2.8957028	up	-4.771567	-2.254463	4.7715669	down	1218.1829	3527.4956	739.27405	LRRC4	NAG14	leucine rich	64101	chr7	-	127455797	127458238		GO:001602	GO:000551	Hs.655003	ref:NM_022	THC247080	ENST00000249363
NM_00103	2.9019863	1.5370407	2.9019863	up	-2.075563	-1.053503	2.0755634	down	457.4206	1327.4283	639.55084	PDE4B	DKFZp686I	phosphodies	5142	chr1	+	66570380	66612849	GO:000716	GO:000562	GO:000382	Hs.198072	ref:NM_001	THC263396	ENST00000371045
NM_00530	2.9187834	1.5453672	2.9187834	up	-2.793561	-1.482105	2.7935606	down	507.06195	1480.004	529.79126	FFAR2	FFA2R|GPF	free fatty ac	2867	chr19	+	40632456	40633449	GO:000716	GO:000588	GO:000158	Hs.248056	ref:NM_005	THC260286	ENST00000246549
NM_02208	2.9229869	1.5474433	2.9229869	up	-3.60168	-1.84867	3.60168	down	1933.1611	5650.6045	1568.8802	C1orf24	FLJ38228|N	chromosome	116496	chr1	-	183029674	183210267	GO:000815	GO:000557	GO:000367	Hs.518662	ref:NM_052	THC267749	ENST00000367511
NM_00063	2.9261341	1.5489959	2.9261341	up	-5.766083	-2.527592	5.7660829	down	2359.4067	6903.9404	1197.3363	IL8RA	C-C|C-C CI	interleukin 8	3577	chr2	-	218735812	218739961	GO:000693	GO:001602	GO:000158	Hs.194778	ref:NM_000	THC246694	ENST00000295683
BC036468	2.9370562	1.5543709	2.9370562	up	-2.869891	-1.520996	2.8698914	down	639.61426	1878.583	654.5833	KLHL2	ABP-KELC	kelch-like 2	11275	chr4	+	166348278	166463749	GO:000688	GO:001562	GO:000377	Hs.388668	ref:NM_007	THC246459	ENST00000226725
BC000213	2.9422234	1.5569068	2.9422234	up	-5.610097	-2.488026	5.6100969	down	3781.737	11126.715	1983.3374	RNF24	G1L	ring finger p	11237	chr20	-	3862215	3944192			GO:000551	Hs.589884	ref:NM_007	THC247053	ENST00000358395
AF014794	2.9462441	1.558877	2.9462441	up	-4.837023	-2.274119	4.8370226	down	2570.4539	7573.1846	1565.6707	TNFRSF10	CD263|DCF	tumor necro	8794	chr8	+	23016443	23030893	GO:000691	GO:000588	GO:000488	Hs.655801	ref:NM_003	THC247160	ENST00000312659
BC006096	2.9469415	1.5592184	2.9469415	up	-2.805421	-1.488217	2.8054213	down	1009.2066	2974.0728	1060.1163	NQO2	DHQV|DIA	NAD(P)H d	4835	chr6	+	2951699	2964987	GO:0006118(electron tra		GO:000827	Hs.533050	ref:NM_000	THC261303	ENST00000380455
AF151081	2.9586881	1.5649576	2.9586881	up	-4.544126	-2.184003	4.5441259	down	3570.2026	10563.116	2324.565	RNF24	G1L	ring finger p	11237	chr20	-	3861736	3862693			GO:000551	Hs.589884	ref:NM_007	THC247053	ENST00000358395
NM_05306	2.9611476	1.5661564	2.9611476	up	-2.896369	-1.534245	2.8963691	down	388.0633	1149.1127	396.7425	GNG2	G protein	guanine nuc	54331	chr14	+	51414059	51506263	GO:000716	GO:000583	GO:000487	Hs.693609	ref:NM_053	NP1137195	ENST00000335281
NM_03077	2.971987	1.5714278	2.971987	up	-2.973322	-1.572076	2.9733222	down	155.95593	463.499	155.8859	WNT5B	MGC2648	wingless-typ	81029	chr12	+	1596482	1626638	GO:000722	GO:000557	GO:000487	Hs.306051	ref:NM_030	THC246356	ENST00000310594
NM_15268	2.9801521	1.575386	2.9801521	up	-5.094114	-2.348831	5.0941136	down	956.38666	2850.1777	559.50415	FLJ32028	-	hypothetical	201799	chr4	-	153766720	153820641		GO:0016020(membrane)		Hs.518900	ref:NM_152	THC247115	ENST00000304385
XM_93396	2.988993	1.5796595	2.988993	up	-3.412345	-1.770763	3.4123448	down	1626.4287	4861.384	1424.6462	JMJD3	KIAA0346	jumonji don	23135	chr17	+	7691782	7698839				Hs.223678	ref:NM_001	THC260182	ENST00000254846
NM_01508	3.0059698	1.5878305	3.0059698	up	-2.204291	-1.140315	2.2042914	down	399.307	1200.3048	544.5309	GARNL4	DKFZp686	GTPase acti	23108	chr17	+	2646481	2887783	GO:005105	GO:000562	GO:000509	Hs.499659	ref:NM_015	THC2680829	
BC037961	3.0188321	1.5939905	3.0188321	up	-7.07892	-2.823529	7.0789203	down	1118.336	3376.0686	476.91858	IL8RB	CD182|CDw	interleukin 8	3579	chr2	+	218699026	218710218	GO:000691	GO:000562	GO:000158	Hs.846	ref:NM_001	THC246202	ENST00000318507
BC039196	3.0286679	1.5986834	3.0286679	up	-4.688551	-2.229142	4.6885515	down	2051.6667	6213.817	1325.317	SLC2A3	FLJ90380|G	solute carrie	6515	chr12	-	7963678	7980032	GO:000597	GO:000562	GO:000521	Hs.419240	ref:NM_006	THC272100	ENST00000075120
XM_49621	3.0320249	1.6002816	3.0320249	up	-4.590206	-2.198559	4.5902056	down	413.4971	1253.7335	273.13232	LOC339287	DKFZp686J	hypothetical	339287	chr17	+	35532111	35548144				Hs.532786	gb:CR7493	THC262902	ENST00000339569
NM_00466	3.0338776	1.6011629	3.0338776	up	-3.757306	-1.909698	3.7573057	down	1189.6929	3609.3826	960.63055	VNN2	FOAP-4|GP	vanin 2	8875	chr6	-	133106701	133120726	GO:000680	GO:001602	GO:001681	Hs.293130	ref:NM_004	NP1195731	ENST00000326499
NM_00721	3.036458	1.6023894	3.036458	up	-4.69088	-2.229859	4.69088	down	3281.582	9964.386	2124.204	RNF24	G1L	ring finger p	11237	chr20	-	3861712	3944036			GO:000551	Hs.589884	ref:NM_007	THC247053	ENST00000358395
NM_01484	3.0519001	1.6097077	3.0519001	up	-2.166923	-1.115648	2.1669234	down	195.96852	598.07635	276.00253	KIAA0329	KIAA0297	KIAA0329	9895	chr14	+	101899130	102038570				Hs.195667	ref:NM_014	THC264174	ENST00000359520
NM_00155	3.0706693	1.6185531	3.0706693	up	-7.428702	-2.89311	7.4287023	down	8174.695	25101.785	3379.0269	IL8RB	CD182|CDw	interleukin 8	3579	chr2	+	218698990	218710220	GO:000691	GO:000562	GO:000158	Hs.846	ref:NM_001	THC246202	ENST00000318507
BC072439	3.0820757	1.6239023	3.0820757	up	-6.177821	-2.627098	6.1778211	down	1794.9603	5532.2036	895.4943	PBEF1	MGC11725	pre-B-cell c	10135	chr7	-	105678233	105712647	GO:0007165(signal trans		GO:000451	Hs.489615	ref:NM_005	THC255066	ENST00000222553
XM_93426	3.1002557	1.6323872	3.1002557	up	-2.693138	-1.429288	2.6931376	down	1763.3538	5466.8477	2029.9177	LOC647064	-	hypothetical	647064	chr16	-	30010285	30011513							
NM_00100	3.1042749	1.6342563	3.1042749	up	-2.892558	-1.532346	2.8925579	down	2213.3147	6870.7373	2375.3154	FPRL1	ALXR|FML	formyl pepti	2358	chr19	+	56956264	56965591	GO:000692	GO:000588	GO:000158	Hs.99855	ref:NM_001	THC247886	ENST00000340023
AF116691	3.107599	1.6358004	3.107599	up	-2.192415	-1.132521	2.1924146	down	370.04797	1149.9607	524.5179	MAN2A2	MANA2X	mannosidase	4122	chr15	+	89265834	89266811	GO:000597	GO:000013	GO:000455	Hs.116459	ref:NM_006	THC249069	ENST00000360468
NM_00305	3.1228478	1.6428623	3.1228478	up	-2.547975	-1.349351	2.5479745	down	386.10226	1205.7386	473.21454	SLC22A4	MGC34546	solute carrie	6583	chr5	+	131658043	131707798	GO:000633	GO:000078	GO:000016	Hs.310591	ref:NM_003	THC247214	ENST00000200652
NM_00562	3.1403693	1.6509342	3.1403693	up	-3.221992	-1.687953	3.2219924	down	4232.0503	13290.201	4124.8394	S100A11	MLN70|S10	S100 calciu	6282	chr1	-	150271605	150276135	GO:000716	GO:000172	GO:000550	Hs.417004	ref:NM_005	THC256244	ENST00000271638
CR625664	3.1829668	1.6703721	3.1829668	up	-2.32015	-1.214218	2.3201501	down	1153.9913	3673.116	1583.1372	DPEP2	MBD2	dipeptidase	64174	chr16	-	66578843	66584670	GO:000650	GO:001602	GO:000423	Hs.372633	ref:NM_022	THC249121	ENST00000268795
NM_07862	3.2243143	1.6889924	3.2243143	up	-2.249515	-1.169614	2.2495147	down	285.69254	921.16254	409.4939	VNN3	HSA238982	vanin 3	55350	chr6	-	133085618	133097596	GO:000680	GO:000557	GO:001681	Hs.183656	ref:NM_018	THC248586	ENST00000275223
BC101480	3.2402286	1.6960956	3.2402286	up	-2.556042	-1.353912	2.5560419	down	165.58525	536.53406	209.90816	PDE4B	DKFZp686I	phosphodies	5142	chr1	+	66151515	66611045	GO:000716	GO:000562	GO:000382	Hs.198072	ref:NM_001	THC263396	ENST00000371045
BC039584	3.2450426	1.6982374	3.2450426	up	-4.220895	-2.077549	4.220895	down	3666.3599	11897.494	2818.7136	RNF24	G1L	ring finger p	11237	chr20	-	3861729	3944157			GO:000551	Hs.589884	ref:NM_007	THC247053	ENST00000358395
NM_02216	3.3321427	1.7364502	3.3321427	up	-2.100935	-1.071032	2.100935	down	1142.7172	3807.6968	1812.382	CARD15	ACUG|BLA	caspase recr	64127	chr16	+	49288550	49324488	GO:000181	GO:000562	GO:000016	Hs.592072	ref:NM_022	THC247730	ENST00000300589
NM_00724	3.3505749	1.7444087	3.3505749	up	-2.67406	-1.419032	2.6740605	down	501.13522	1679.0911	627.91815	KLHL2	ABP-KELC	kelch-like 2	11275	chr4	+	166348237	166463749	GO:000688	GO:001562	GO:000377	Hs.388668	ref:NM_007	THC246459	ENST00000226725
XM_93238	3.3935254	1.7627848	3.3935254	up	-2.414058	-1.27146	2.4140581	down	2574.0264	8735.024	3618.3984	LOC65384	-	similar to N	653840	chr7	+	72272601	72287970							
NM_01816	3.4270089	1.7769499	3.4270089	up	-3.768459	-1.913975	3.7684591	down	386.1306	1323.273	351.14432	C12orf35	FLJ10652|F	chromosome	55196	chr12	+	32003619	32037308				Hs.445129	ref:NM_018	THC260118	ENST00000312561
NM_00404	3.4805089	1.7992983	3.4805089	up	-4.83009	-2.27205	4.8300896	down	1074.7164	3740.56	774.4287	BCL2A1	BCL2L5|BF	BCL2-relate	597	chr15	-	78040289	78050698	GO:000691	GO:000562	GO:000551	Hs.227817	ref:NM_004	THC247733	ENST00000267953
NM_02271	3.4847907	1.801072	3.4847907	up	-7.12748	-2.833392	7.1274802	down	1762.3843	6141.5405	861.67065	MMP25	MMP20|MT	matrix meta	64386	chr16	+	3036682	3050728	GO:000027	GO:000557	GO:000422	Hs.654979	ref:NM_022	THC247511	ENST00000336577
AB023202	3.4891219	1.802864	3.4891219	up	-3.607203	-1.850881	3.6072028	down	239.78185	836.6281	231.93265	RPH3A	KIAA0985|	rabphilin 3A	22895	chr12	+	111714055	111821065	GO:000681	GO:000802	GO:000521	Hs.21239	gb:AB0232	THC262768	ENST00000360622
NM_18263	3.5577593	1.8309689	3.5577593	up	-2.812691	-1.491951	2.8126909	down	1503.5809	5349.379	1901.8723	C9orf164	FLJ39737	chromosome	349236	chr9	-	91166335	91169386				Hs.494406	ref:NM_182	THC262384	ENST00000339861
NM_00650	3.5579001	1.831026	3.5579001	up	-2.067942	-1.048196	2.0679419	down	431.94748	1536.826	743.1669	PTPRE	DKFZp313I	protein tyro	5791	chr10	+	129595314	129774155	GO:000647	GO:000562	GO:000487	Hs.127022	ref:NM_006	THC251607	ENST00000254667
NM_17321	3.5824797	1.8409585	3.5824797	up	-8.171946	-3.03068	8.1719457	down	594.4125	2129.4707	260.58307	KRT23	CK23|DKFZ	keratin 23 (l	25984	chr17	-	36332477	36347362		GO:000588	GO:000519	Hs.9029	ref:NM_015	THC246682	ENST00000209718
BC013327	3.5863752	1.8425264	3.5863752	up	-3.567943	-1.835093	3.5679432	down	1359.2509	4874.7837	1366.2728	KCNJ15	KIR1.3|KIR	potassium ir	3772	chr21	+	38550533	38595279	GO:000681	GO:000562	GO:000524	Hs.411299	ref:NM_170	THC247225	ENST00000328656
AK057590	3.6860834	1.8820887	3.6860834	up	-2.793952	-1.482307	2.7939523	down	629.8899	2321.8267	831.01874	AMICA1	AMICA|CR	adhesion mo	120425	chr11	-	117569890	117574604	GO:000715	GO:001602	GO:000551	Hs.16291	ref:NM_153	THC255002	ENST00000292067
NM_02098	3.6881988	1.8829164	3.6881988	up	-3.206238	-1.680881	3.2062377	down	1575.916	5812.2915	1812.8074	AQP9	HsT17287|S	aquaporin 9	366	chr15	+	56217770	56265401	GO:000683	GO:000588	GO:000527	Hs.104624	ref:NM_020	NP509229	ENST00000219919
NM_01806	3.6977609	1.886652	3.6977609	up	-2.589215	-1.372515	2.589215	down	434.68497	1607.3611	620.7909	C6orf166	FLJ10342|d	chromosome	55122	chr6	-	88441692	88468645				Hs.485915	ref:NM_018	THC256242	ENST00000257787
XM_04327	3.6995703	1.8873577	3.6995703	up	-4.786908	-2.259094	4.7869082	down	1525.3821	5643.2583	1178.8942	JMJD3	KIAA0346	jumonji don	23135	chr17	+	7683965	7698839				Hs.223678	ref:NM_001	THC260182	ENST00000254846
NM_03256	3.7295442	1.8989993	3.7295442	up	-3.021639	-1.595331	3.021639	down	552.3058	2059.8489	681.6992	DGAT2	DKFZp686	diacylglycer	84649	chr11	+	75157455	75190225	GO:000607	GO:000562	GO:000414	Hs.334305	ref:NM_032	NP1242886	ENST00000228027
NM_01551	3.8473315	1.9438581	3.8473315	up	-9.536072	-3.253395	9.5360722	down	687.74084	2645.967	277.46927	KRT23	CK23|DKFZ	keratin 23 (l	25984	chr17	-	36332477	36347362		GO:000588	GO:000519	Hs.9029	ref:NM_015	THC246682	ENST00000209718
NM_02235	3.8846656	1.9577904	3.8846656	up	-2.7176	-1.442333	2.7176002	down	418.25543	1624.7825	597.874	DPEP3	MBD3	dipeptidase	64180	chr16	-	66567066	66571867	GO:000650	GO:000562	GO:000423	Hs.302028	ref:NM_022	THC247208	ENST00000268793
NM_00057	3.9121616	1.967966	3.9121616	up	-2.375016	-1.247937	2.3750157	down	1494.0942	5845.138	2461.0945	IL1RN	ICIL-1RA|I	interleukin 1	3557	chr2	+	113591940	113608064	GO:000662	GO:000557	GO:000515	Hs.81134	gb:BC0684	THC2554255	
NM_02035	4.0469664	2.0168409	4.0469664	up	-2.253133	-1.171932	2.2531325	down	175.22131	709.11475	314.72394	C20orf32	HEFL	chromosome	57091	chr20	+	54420770	54467243	GO:000716	GO:001602	GO:000015	Hs.473144	ref:NM_020	THC248833	ENST00000360314
NM_00508	4.0615135	2.0220174	4.0615135	up	-2.766477	-1.46805	2.766477	down	927.5871	3767.4075	1361.8069	TRIM25	EFP|RNF14	tripartite mo	7706	chr17	-	52320268	52346408		GO:000573	GO:000370	Hs.528952	ref:NM_005	THC247350	ENST00000316881
NM_00472	4.0735431	2.0262842	4.0735431	up	-2.81716	-1.494242	2.8171604	down	1152.8286	4696.097	1666.9612	EDG4	EDG-4|LPA	endothelial (	9170	chr19	-	19595476	19600017	GO:000716	GO:000588	GO:000161	Hs.122575	ref:NM_004	THC266878	ENST00000358804
NM_00644	4.080772	2.0288421	4.080772	up	-2.159051	-1.110397	2.1590511	down	475.07504	1938.6729	897.9282	CDC42EP3	BORG2|CE	CDC42 effe	10602	chr2	-	37724246	37752830	GO:000716	GO:001562	GO:000551	Hs.369574	ref:NM_006	THC246875	ENST00000379035
NM_18171	4.1046171	2.0372476	4.1046171	up	-7.28746	-2.865416	7.28746	down	311.82373	1279.917	175.6328	HCG27	FLJ40123|M	HLA compl	253018	chr6	+	31273515	31279723				Hs.659818	ref:NM_181	THC248079	ENST00000376240
NM_14459	4.2868329	2.0999122	4.2868329	up	-3.23623	-1.694314	3.2362295	down	225.84566	968.1626	299.16376	ANKRD22	MGC22805	ankyrin repe	118932	chr10	-	90572281	90609876				Hs.217484	ref:NM_144	THC247572	ENST00000371930

NM_17755	4.312451	2.1085081	4.312451	up	-7.542601	-2.915062	7.5426007	down	1228.3627	5297.254	702.31134	GPR109A	HM74a|HM	G protein-cc	338442	chr12	-	121751792	121753857	GO:00071	GO:001602	GO:000158	Hs.524812	ref:NM_177	THC246151	ENST00000328880
NM_00601	4.4064444	2.139615	4.4064444	up	-5.103446	-2.351472	5.1034465	down	1524.9396	6719.5615	1316.6713	GPR109B	HM74|PUM	G protein-cc	8843	chr12	-	121765255	121767297	GO:00071	GO:000588	GO:000158	Hs.458425	ref:NM_006	THC2461513	
AF249277	4.5972298	2.2007648	4.5972298	up	-5.255012	-2.393694	5.255012	down	493.6314	2269.337	431.8424	MTHFS	HsT19268	5,10-methen	10588	chr15	-	77978301	78003099	GO:000939	GO:000582	GO:000028	Hs.459049	ref:NM_006	THC270724	ENST00000258874
AY234180	5.2228768	2.3848447	5.2228768	up	-4.00758	-2.002731	4.00758	down	307.69843	1607.071	401.00784	BCL2A1	BCL2L5|BF	BCL2-relate	597	chr15	-	78040464	78050524	GO:000691	GO:000562	GO:000551	Hs.227817	ref:NM_004	THC247733	ENST00000267953
AB002384	5.3026741	2.4067201	5.3026741	up	-3.630673	-1.860237	3.630673	down	310.87164	1648.451	454.03455	C6orf32	DIFF40|DIF	chromosom	9750	chr6	-	24912491	25019174				Hs.559459	gb:AB0023	THC251509	ENST00000378027
BC010952	6.0329695	2.5928683	6.0329695	up	-4.374521	-2.129125	4.3745211	down	170.35841	1027.7671	234.94391	PI3	ESI|MGC13	peptidase in	5266	chr20	+	43236956	43238595	GO:000762	GO:000557	GO:000486	Hs.112341	ref:NM_002	THC246161	ENST00000243924
NM_00224	7.0276518	2.8130427	7.0276518	up	-4.838379	-2.274524	4.8383791	down	597.60175	4199.737	868.00494	KCNJ15	KIR1.3|KIR	potassium ir	3772	chr21	+	38550533	38595618	GO:000681	GO:000562	GO:000524	Hs.411299	ref:NM_170	THC247225	ENST00000328656
AF035947	7.5599764	2.9183817	7.5599764	up	-2.435893	-1.284451	2.4358926	down	632.5817	4782.3027	1963.265	CISH	CIS|CIS-1|C	cytokine ind	1154	chr3	-	50618924	50624196	GO:000155	GO:000557	GO:000367	Hs.655334	ref:NM_145	THC246359	ENST00000348721
NM_14507	10.417097	3.3808814	10.417097	up	-2.404833	-1.265937	2.4048333	down	592.09125	6167.872	2564.7815	CISH	CIS|CIS-1|C	cytokine ind	1154	chr3	-	50618923	50624207	GO:000155	GO:000557	GO:000367	Hs.655334	ref:NM_145	THC246359	ENST00000348721
AF132297	10.723215	3.4226657	10.723215	up	-2.769831	-1.469798	2.7698314	down	541.4777	5806.382	2096.2944	CISH	CIS|CIS-1|C	cytokine ind	1154	chr3	-	50618930	50624207	GO:000155	GO:000557	GO:000367	Hs.655334	ref:NM_145	THC246359	ENST00000348721

SEQ_ID	Fold chang	Log Fold ch	Absolute Fc	Regulation(	Fold chang	Log Fold ch	Absolute Fc	Regulation(	[AE-1](nor	[AE-3](nor	[AE-10](no	GENE_NA	SYNONYM	DESCRIPT	NCBI_GEN	CHROMO	STRAND	START	STOP	GO biologi	GO cellular	GO molecu	UniGene	Dbid	TIGRID	EnsemblID
Z15108	2.0241838	1.0173403	2.0241838	up	2.7610363	1.4652099	2.7610363	up	188.72653	382.01718	1054.7633	PRKCZ	PKC2	protein kina	5590	chr1	+	1971947	2106686	GO:000646	GO:000562	GO:0000166(nucleotide		gb:AB0079	THC2527109	
BC037798	2.0484133	1.0345068	2.0484133	up	2.4251858	1.2780953	2.4251858	up	634.3157	1299.3407	3151.1426	CGI-38	-	brain specifi	51673	chr16	-	65981214	65983350				Hs.534458	ref:NM_016	NP211869	ENST00000290942
NM_00103	2.103378	1.0727082	2.103378	up	2.7931822	1.4819097	2.7931822	up	334.29022	703.1387	1963.9945	PRKCZ	PKC2	protein kina	5590	chr1	+	1994945	2106694	GO:000646	GO:000562	GO:0000166(nucleotide		gb:AB0079	THC2527109	
NM_00716	2.1274717	1.0891399	2.1274717	up	3.9825857	1.9937054	3.9825857	up	111.4468	237.09991	944.2707	ABCA8	KIAA0822	ATP-bindin	10351	chr17	-	64375024	64463128	GO:000681	GO:001602	GO:000016	Hs.58351	ref:NM_007	THC246363	ENST00000269080
AK022468	2.2809985	1.1896655	2.2809985	up	3.5298331	1.8196	3.5298331	up	151.83627	346.3383	1222.5164	SORBS1	CAP|DKFZ	sorbin and S	10580	chr10	-	97140403	97311120	GO:000681	GO:000172	GO:000377	Hs.38621	gb:AK0224	THC247915	ENST00000371227
NM_00640	2.2897658	1.1952	2.2897658	up	2.1812388	1.1251477	2.1812388	up	624.15515	1429.1691	3117.3591	NEDD9	CAS-L|CAS	neural precu	4739	chr6	-	11291518	11340887	GO:000007	GO:000563	GO:000551	Hs.37982	ref:NM_006	THC246839	ENST00000379446
NM_02303	2.2961893	1.1992416	2.2961893	up	2.0889467	1.0627757	2.0889467	up	156.49878	359.35083	750.66473	FRY	13CDNA73	furry homol	10129	chr13	+	31503436	31768771	GO:000635	GO:001602	GO:000552	Hs.591225	ref:NM_023	THC256136	ENST00000380250
NM_01673	3.0155165	1.5924051	3.0155165	up	11.694855	3.5478021	11.694855	up	352.9474	1064.3187	12447.053	FOLR1	FBP|FOLR|	folate recept	2348	chr11	+	71578905	71584990	GO:000689	GO:000562	GO:000487	Hs.73769	ref:NM_016	NP226704	ENST00000312293

**EnsemblID**
ENST00000330588
ENST00000368406
ENST00000297435
ENST00000231751
ENST00000052754
ENST00000367816
ENST00000307998
ENST00000228916
ENST00000380456
ENST00000355906
ENST00000368391
ENST00000371711
ENST00000308926
ENST00000368391
ENST00000299610
ENST00000261978
ENST00000344550
ENST00000357898
ENST00000324256
ENST00000375582
5
ENST00000261037
ENST00000357867
ENST00000310044
ENST00000262429
ENST00000317508
ENST00000249842
ENST00000254963
ENST00000368391
ENST00000347401
ENST00000231751
ENST00000368868
ENST00000262865
7
ENST00000361084
ENST00000342280
ENST00000326724
ENST00000367816
ENST00000368391
ENST00000320786
ENST00000297435
ENST00000219821
ENST00000310677
ENST00000231751
ENST00000368223
ENST00000249842
ENST00000348956
ENST00000301187
ENST00000272462
9
ENST00000337576
ENST00000347401
ENST00000338844
ENST00000312293
ENST00000268605
ENST00000301187
ENST00000374100
ENST00000297268
ENST00000357665
9
ENST00000264601
ENST00000344616
ENST00000264605
ENST00000304636
ENST00000368391
ENST00000324873
ENST00000341918
8
ENST00000340958
ENST00000340900
ENST00000252900
8
ENST00000350219

**EnsemblID**
ENST00000229829
ENST00000278207
ENST00000319294
ENST00000370597
5
ENST00000341618
ENST00000294984

ENST00000341618

ENST00000333837
ENST00000368041

ENST00000368041

ENST00000341618
ENST00000269064
ENST00000303992
ENST00000368041
ENST00000344720
ENST00000325680
ENST00000263707
ENST00000371514

Annotations

STOP	STRAND	GO biolog	GO cellula	GO molecu	UniGene	Dbid	TIGRID	EnsemblID
2.16E+08	-	GO:00069	GO:00055	GO:00052	Hs.203717	ref:NM_05	NP127642	ENST00000357867
21777491	+	GO:00015	GO:00160	GO:00002	Hs.75431	ref:NM_00	THC24671	ENST00000374832
31337907	-							
74818404	+	GO:00063	GO:00056	GO:00037	Hs.25647	ref:NM_00	THC25445	ENST00000303562
46967662	+	GO:00071	GO:00058	GO:00485	Hs.466814	gb:BC005008;gb:CB240801		
90096495	-	GO:00098	GO:00055	GO:00055	Hs.156316	ref:NM_00	THC25224	ENST00000052754
36938071	-	GO:00452	GO:00058	GO:00052	Hs.654568	ref:NM_00	THC25532	ENST00000361566
72548080	+	GO:00015	GO:00056	GO:00037	Hs.508234	ref:NM_00	NP284958	ENST00000377687
22847189	+	GO:00068	GO:0005576(extracell		Hs.467753	ref:NM_17	THC26777	ENST00000374637
93652783	+	GO:00069	GO:00057	GO:00036	Hs.532634	ref:NM_00	THC24736	ENST00000298902
22838686	+	GO:00068	GO:0005602(complem		Hs.632379	ref:NM_01	THC26912	ENST00000374642
54522973	-	GO:00002	GO:00055	GO:00042	Hs.591033	ref:NM_00	THC24883	ENST00000322569
42847743	-	GO:00018	GO:00055	GO:00084	Hs.275775	ref:NM_00	THC24989	ENST00000388827
19230746	-	GO:00071	GO:00015	GO:00036	Hs.296049	ref:NM_00	NP121143	ENST00000299610
20512677	+	GO:00015	GO:00056	GO:00001	Hs.502876	ref:NM_00	THC26414	ENST00000272233
70652454	+	GO:00065	GO:00055	GO:00042	Hs.513711	ref:NM_00	THC26053	ENST00000355906
2.16E+08	-	GO:00069	GO:00055	GO:00052	Hs.203717	ref:NM_05	NP127642	ENST00000357867
57138862	+	GO:00069	GO:00055	GO:00048	Hs.384598	ref:NM_00	THC25833	ENST00000278407
46967953	+	GO:00071	GO:00058	GO:00485	Hs.466814	gb:BC005008;gb:CB240801		
57138902	+	GO:00069	GO:00055	GO:00048	Hs.384598	ref:NM_00	THC25833	ENST00000278407
6671660	-	GO:00017	GO:00055	GO:00048	Hs.529053	ref:NM_00	THC24700	ENST00000389151
31589028	+	GO:00071	GO:00055	GO:00036	Hs.644633	ref:NM_00	THC25181	ENST00000266085
21777489	+	GO:00015	GO:00160	GO:00002	Hs.75431	ref:NM_00	THC24671	ENST00000374832
22860714	+	GO:00068	GO:0005602(complem		Hs.8986	ref:NM_00	THC24669	ENST00000314933
2.11E+08	+	GO:00063	GO:00056	GO:00036	Hs.460	ref:NM_00	NP120535	ENST00000366987
85821978	+	GO:00015	GO:00055	GO:00055	Hs.8867	ref:NM_00	NP077848	ENST00000360431
1.89E+08	-	GO:00001	GO:00056	GO:00036	Hs.478588	ref:NM_13	THC24665	ENST00000232014
7136184	-	GO:00065	GO:00055	GO:00038	Hs.524224	ref:NM_00	THC2498629	
64996190	+	GO:00071	GO:00056	GO:00055	Hs.76206	ref:NM_00	NP146631	ENST00000341529
95165289	-	GO:00069	GO:00055	GO:00037	Hs.483454	ref:NM_00	THC25861	ENST00000370206
1.32E+08	-	GO:00015	GO:00055	GO:00051	Hs.591346	ref:NM_00	NP095144	ENST00000367976
90100937	-	GO:00098	GO:00055	GO:00055	Hs.156316	ref:NM_00	THC25224	ENST00000052754
1.86E+08	-	GO:00066	GO:00057	GO:00002	Hs.406678	ref:NM_00	THC25127	ENST00000281455
2.16E+08	-	GO:00069	GO:00055	GO:00052	Hs.203717	ref:NM_05	NP127642	ENST00000357867
36938160	-	GO:00452	GO:00058	GO:00052	Hs.654568	ref:NM_00	THC25532	ENST00000361566
58816033	-	GO:00071	GO:00058	GO:00048	Hs.23582	ref:NM_00	THC24688	ENST00000371225
19231086	-	GO:00071	GO:00015	GO:00036	Hs.296049	ref:NM_00	NP121143	ENST00000299610
1.24E+08	+	GO:00015	GO:00055	GO:00042	Hs.501280	ref:NM_00	NP710487	ENST00000368984
12957867	+	GO:00071	GO:00058	GO:00048	Hs.631733	ref:NM_00	NP120629	ENST00000014914
20512682	+	GO:00015	GO:00056	GO:00001	Hs.502876	ref:NM_00	THC26414	ENST00000272233
35764692	-	GO:00064	GO:00056	GO:00037	Hs.407190	ref:NM_00	THC27122	ENST00000357266
1.72E+08	-	GO:0006470(protein a		GO:00047	Hs.171695	ref:NM_00	THC25283	ENST00000239223
36227114	-	GO:00071	GO:00055	GO:00038	Hs.517033	ref:NM_19	THC2659478	
79752463	+	GO:00071	GO:00057	GO:00055	Hs.480042	ref:NM_00	THC27707	ENST00000264908
74818666	+	GO:00063	GO:00056	GO:00037	Hs.25647	ref:NM_00	THC25445	ENST00000303562
1.14E+08	+			GO:00081	Hs.503911	ref:NM_00	THC26369	ENST00000299964
44375678	+		GO:00055	GO:00052	Hs.24601	ref:NM_00	THC25002	ENST00000262722
50670276	+	GO:00000	GO:00056	GO:00037	Hs.590958	ref:NM_00	THC25060	ENST00000353609
1.08E+08	-	GO:00064	GO:00057	GO:0004872(receptor		ens:ENST0000036900		ENST00000369002
65176610	-		GO:00160	GO:00055	Hs.8904	ref:NM_00	NP121068	ENST00000374737
90096460	-	GO:00098	GO:00055	GO:00055	Hs.156316	ref:NM_00	THC25224	ENST00000052754
90096460	-	GO:00098	GO:00055	GO:00055	Hs.156316	ref:NM_00	THC25224	ENST00000052754
90096460	-	GO:00098	GO:00055	GO:00055	Hs.156316	ref:NM_00	THC25224	ENST00000052754
22847190	+	GO:00068	GO:0005576(extracell		Hs.467753	ref:NM_17	THC26777	ENST00000374637
1.18E+08	+	GO:00071	GO:00058	GO:00048	Hs.496646	ref:NM_00	THC24886	ENST00000371666
72573179	-							
43922003	+				Hs.530443	ens:ENST0000033944		ENST00000339446
36854135	-							

Annotations

STOP	STRAND	GO biolog	GO cellula	GO molecu	UniGene	Dbid	TIGRID	EnsemblID

74818404	+	GO:00063	GO:00056	GO:00037	Hs.25647	ref:NM_00	THC25445	ENST00000303562
93652783	+	GO:00069	GO:00057	GO:00036	Hs.532634	ref:NM_00	THC24736	ENST00000298902
85821978	+	GO:00015	GO:00055	GO:00055	Hs.8867	ref:NM_00	NP077848	ENST00000360431
1.32E+08	-	GO:00015	GO:00055	GO:00051	Hs.591346	ref:NM_00	NP095144	ENST00000367976
12957867	+	GO:00071	GO:00058	GO:00048	Hs.631733	ref:NM_00	NP120629	ENST00000014914
50670276	+	GO:00000	GO:00056	GO:00037	Hs.590958	ref:NM_00	THC25060	ENST00000353609
90096460	-	GO:00098	GO:00055	GO:00055	Hs.156316	ref:NM_00	THC25224	ENST00000052754
43922003	+				Hs.530443	ens:ENST0000033944		ENST00000339446

	Annotations							
**STOP**	**STRAND**	**GO biolog**	**GO cellula**	**GO molec**	**UniGene**	**Dbid**	**TIGRID**	**EnsemblID**
24753732	+		GO:00056	GO:00001	Hs.417959	ref:NM_03	NP500953	ENST00000335473
99866461	+	GO:00068	GO:00057	GO:00052	Hs.108268	ref:NM_20	THC26246	ENST00000361529
60017660	+	GO:00069	GO:00058	GO:00048	Hs.661219	ref:NM_00	NP365687	ENST00000359085
287963	+	GO:00069	GO:00058	GO:00048	Hs.512572	ref:NM_01	NP084034	ENST00000291633
34703152	-	GO:00001	GO:00057	GO:00050	Hs.693639	ref:NM_03	THC24731	ENST00000262866
3340717	+	GO:00063	GO:00056	GO:00036	Hs.99500	ref:NM_02	THC26016	ENST00000270722
15999716	+	GO:00063	GO:00007	GO:00037	Hs.25960	ref:NM_00	THC24782	ENST00000281043
95413524	+	GO:00063	GO:00056	GO:00036	Hs.437376	ref:NM_013434;ref:N		ENST00000295225
1.57E+08	-				Hs.666099	ref:NM_13	THC24723	ENST00000302938
271823	+	GO:00069	GO:00058	GO:00048	Hs.512572	ref:NM_01	NP084034	ENST00000291633
271823	+	GO:00069	GO:00058	GO:00048	Hs.512572	ref:NM_01	NP084034	ENST00000291633
99761084	-			GO:00055	Hs.500736	ref:NM_01	THC25030	ENST00000370597
70347484	+		GO:0016020(membrar		Hs.370262	gb:AK000	THC24752	ENST00000335464
22023120	-							
1872817	+	GO:00150	GO:0016020(membrar		Hs.144980	gb:AK074	THC2482725	
20406034	+	GO:0008152(metaboli		GO:00038	Hs.298252	ref:NM_00	THC24745	ENST00000329697
59728199	-							
54438334	-							
11252804	-							
1.36E+08	-							
77568569	+							
87328126	-							
52074168	-							
64562088	-							
1.46E+08	-							
14789544	+		GO:0005737(cytoplas		Hs.661452	ref:NM_03	THC24911	ENST00000253697
49485509	+	GO:00068	GO:0005737(cytoplas		Hs.526441	ref:NM_18	THC2605336	
19998424	-							
98211660	+	GO:00064	GO:00160	GO:00001	Hs.653244	gb:AL133	THC2612465	
60070260	+	GO:00069	GO:00058	GO:00048	Hs.645228	ref:NM_01	THC24706	ENST00000326321
35273967	-		GO:00056	GO:00036	Hs.444388	ref:NM_01	NP124156	ENST00000293068
35273967	-		GO:00056	GO:00036	Hs.444388	ref:NM_01	NP124156	ENST00000293068
27180439	-	GO:00063	GO:00056	GO:00037	Hs.592166	ref:NM_01	NP140032	ENST00000283921
31231446	-	GO:00068	GO:00055	GO:00048	Hs.514821	ref:NM_00	THC24650	ENST00000293272
18592843	+		GO:0005624(membrar		Hs.329850	ref:NM_01	THC24614	ENST00000262817
38654183	+	GO:0006396(RNA pro		GO:00037	Hs.173311	ref:NM_15	THC24633	ENST00000315616
1.3E+08	-				Hs.373606	ref:NM_19	THC24918	ENST00000373078
34513027	-				Hs.522085	ref:NM_19	THC26145	ENST00000303992
1.61E+08	-	GO:00072	GO:00055	GO:00036	Hs.350581	ref:NM_05	THC24879	ENST00000367929
54786072	+		GO:00055	GO:00051	Hs.35101	ref:NM_00	THC24742	ENST00000246794
1.49E+08	-				Hs.652227	ref:NM_00	THC26012	ENST00000378008
34026289	+							
54244175	-							
59956315	+							
6193546	+							
1.02E+08	-							
22090936	+							
39905106	+				Hs.693637	ref:NM_01	THC2491244	
99340678	+				Hs.662302	ref:NM_00	THC26375	ENST00000370640
1.34E+08	+	GO:00063	GO:00056	GO:00036	Hs.573153	ref:NM_00	THC24965	ENST00000342854
86827305	+	GO:00060	GO:00057	GO:00084	Hs.125039	ref:NM_02	THC24728	ENST00000331536
1.77E+08	+		GO:0005737(cytoplas		Hs.101761	ref:NM_01	THC24757	ENST00000274605
44175498	+	GO:00071	GO:00056	GO:00080	Hs.632404	ref:NM_05	THC26629	ENST00000372354
1.61E+08	-	GO:00072	GO:00055	GO:00036	Hs.350581	ref:NM_05	THC24879	ENST00000367929
21950353	+	GO:00063	GO:00007	GO:00036	Hs.131055	ref:NM_18	NP800281	
42740413	+		GO:0005634(nucleus)		Hs.473912	ref:NM_01	THC24754	ENST00000319294
39094454	-	GO:00063	GO:00055	GO:00036	Hs.438	ref:NM_00	THC24720	ENST00000318579
22090933	+	GO:00069	GO:00058	GO:00048	Hs.74647	gb:BC063	NP1155360	
1.42E+08	+							
1.42E+08	+							
1.42E+08	+							
2811625	+	GO:00065	GO:00056	GO:00042	Hs.72026	ref:NM_00	THC24754	ENST00000005995
42169399	+			GO:00055	Hs.501114	gb:BC093	NP1164839	
38502423	+	GO:00081	GO:00055	GO:00036	Hs.147695	ref:NM_14	THC24843	ENST00000348862
1.2E+08	-	GO:00071	GO:00058	GO:00015	Hs.248119	ref:NM_00	THC24797	ENST00000239032
226904	+	GO:00069	GO:00058	GO:00048	Hs.645228	ref:NM_01	THC24706	ENST00000326321

1.13E+08	+	GO:00069	GO:00055	GO:00051	Hs.211238	ref:NM_01	NP861722	ENST00000259205
5436786	-	GO:00070	GO:00058	GO:00001	Hs.163079	ref:NM_02	THC24788	ENST00000380419
226904	+	GO:00069	GO:00058	GO:00048	Hs.645228	ref:NM_01	THC24706	ENST00000326321
22090933	+	GO:00069	GO:00058	GO:00048	Hs.74647	gb:BC063	NP1155360	
86942549	-	GO:00069	GO:00058	GO:00055	Hs.405667	ref:NM_17	THC24908	ENST00000337868
69336254	-				Hs.464079	ref:NM_18	NP146566	ENST00000321800
6597117	-	GO:00015	GO:00017	GO:00001	Hs.498570	ref:NM_00	THC24717	ENST00000263125
72513344	-	GO:00069	GO:00056	GO:00048	Hs.24640	ref:NM_00	THC24663	ENST00000261918
1.14E+08	-	GO:00069	GO:00055	GO:00051	Hs.278909	ref:NM_17	THC24838	ENST00000327407
60051298	+		GO:00058	GO:00048	Hs.654608	ref:NM_01	THC27529	ENST00000291633
21173793	+	GO:00000	GO:00056	GO:00080	Hs.111	ref:NM_00	THC24806	ENST00000382353
60070101	+	GO:00069	GO:00058	GO:00048	Hs.645228	ref:NM_00	THC24706	ENST00000326321
288060	+	GO:00069	GO:00058	GO:00048	Hs.512572	ref:NM_01	NP084034	ENST00000291633
85517555	+	GO:0007242(intracellular signalin			Hs.209542	gb:BC110	NP145707	ENST00000389938
22090936	+	GO:00069	GO:00058	GO:00048	Hs.74647	gb:BC063	NP1155360	
41219170	+	GO:00076	GO:00055	GO:00036	Hs.302019	ref:NM_02	THC24846	ENST00000378220
3526560	+		GO:00056	GO:00055	Hs.155995	ref:NM_02	THC2474736	
288306	+	GO:00069	GO:00058	GO:00048	Hs.512572	ref:NM_01	NP084034	ENST00000291633
272126	+	GO:00069	GO:00058	GO:00048	Hs.512572	ref:NM_01	NP084034	ENST00000291633
56648364	+	GO:0006487(protein a		GO:00048	Hs.654472	ref:NM_00	THC24729	ENST00000299022
17865897	+	GO:00068	GO:00160	GO:00052	Hs.584804	ref:NM_00	THC24826	ENST00000222248
48638931	+	GO:00068	GO:00056	GO:00052	Hs.130730	ref:NM_00	THC24784	ENST00000199280
25244956	-	GO:00060	GO:00055	GO:00051	Hs.1897	ref:NM_00	THC24810	ENST00000264708
1.05E+08	-		GO:0016020(membrar		Hs.680365	ref:NM_00	THC24851	ENST00000329905
8565237	+							
1.58E+08	+							
1.06E+08	+							
2.46E+08	+							
1.3E+08	+	GO:00068	GO:00058	GO:00001	Hs.522413	ref:NM_00	THC24938	ENST00000341179
62186156	-				Hs.570316	ref:NM_00	THC24855	ENST00000308906
38414444	-	GO:00015	GO:00055	GO:00055	Hs.349705	ref:NM_00	THC24798	ENST00000377694
1.49E+08	-				Hs.652227	ref:NM_00	THC26012	ENST00000378008
1.64E+08	-	GO:00063	GO:00056	GO:00037	Hs.26550	ref:NM_00	THC26061	ENST00000367892
1766240	+				Hs.7247	gb:AK074	THC2504155	
3059669	+	GO:00069	GO:00056	GO:00051	Hs.943	ref:NM_00	THC25889	ENST00000008180
51109876	-							
53976682	-				Hs.646574	ref:NM_00	THC24759	ENST00000321691
6448842	-	GO:00071	GO:00058	GO:00048	Hs.462529	ref:NM_14	NP114549	ENST00000356876
37617144	+							
1.42E+08	+							
1.17E+08	+	GO:00017	GO:00058	GO:00048	Hs.523500	ref:NM_00	THC24719	ENST00000369478
86871578	-	GO:00069	GO:00058	GO:00055	Hs.85258	ref:NM_00	THC24680	ENST00000283635
27252717	+	GO:00063	GO:00056	GO:00037	Hs.369879	ref:NM_00	THC24849	ENST00000222761
54365717	+	GO:00065	GO:00057	GO:00042	Hs.277937	ref:NM_00	THC24765	ENST00000231009
60017783	+	GO:00069	GO:00058	GO:00048	Hs.661219	ref:NM_00	NP365687	ENST00000359085
3752810	-	GO:00000	GO:00056	GO:00001	Hs.631845	ref:NM_13	THC27825	ENST00000310132
1.34E+08	+	GO:00063	GO:00056	GO:00036	Hs.573153	ref:NM_00	THC24965	ENST00000342854
1.14E+08	-	GO:00063	GO:00056	GO:00037	Hs.469728	ref:NM_01	NP207698	ENST00000348715
1.44E+08	-	GO:00071	GO:00056	GO:00055	Hs.415762	ref:NM_00	THC24736	ENST00000301263
1.74E+08	-	GO:00071	GO:00058	GO:00015	Hs.248115	ref:NM_00	THC26039	ENST00000241256
19716846	-	GO:00065	GO:00160	GO:00151	Hs.197116	ref:NM_00	THC24751	ENST00000382932
1.02E+08	-	GO:00002	GO:00055	GO:00042	Hs.591946	ref:NM_00	THC26655	ENST00000260228
41426593	+	GO:00069	GO:00058	GO:00048	Hs.194721	ref:NM_00	THC24879	ENST00000373089
29290876	-	GO:00064	GO:00056	GO:00017	Hs.17958	ref:NM_00	THC24717	ENST00000338911
86942549	-	GO:00069	GO:00058	GO:00055	Hs.405667	ref:NM_17	THC24908	ENST00000337868
1.01E+08	-	GO:00063	GO:00056	GO:00037	Hs.520293	ref:NM_00	THC24793	ENST00000262901
5558225	+			GO:00055	Hs.239600	ref:NM_00	THC24726	ENST00000315238
31347222	-	GO:00061	GO:00057	GO:00041	Hs.250760	ref:NM_005205;gb:BC029818		
27738789	-	GO:00018	GO:00056	GO:00037	Hs.591663	ref:NM_00	THC2631102	
9054610	+	GO:00069	GO:00160	GO:00048	Hs.558446	ref:NM_00	NP387863	ENST00000356986
1E+08	+	GO:00081	GO:00055	GO:00036	Hs.521083	ref:NM_00	THC24727	ENST00000300176
85779380	+	GO:00068	GO:0005615(extracell		Hs.105806	ref:NM_00	THC26417	ENST00000263863
10359227	+	GO:00071	GO:00058	GO:00048	Hs.562457	ref:NM_00	NP492171	ENST00000336164
288431	+	GO:00069	GO:00058	GO:00048	Hs.512572	ref:NM_01	NP084034	ENST00000291633
255356	+							
60051836	+		GO:00058	GO:00048	Hs.654608	ref:NM_01	THC27529	ENST00000291633
1.09E+08	+				Hs.436618	ref:NM_01	THC27877	ENST00000389626
49229100	+	GO:00063	GO:00056	GO:00036	Hs.279840	ref:NM_01	THC26374	ENST00000187879
1.02E+08	-							
60651900	+	GO:00080	GO:00058	GO:00050	Hs.58685	ref:NM_01	THC2463150	
59987590	+							
37569963	-		GO:00160	GO:00048	Hs.91622	ref:NM_01	THC25010	ENST00000333039
40899780	+	GO:00001	GO:00056	GO:00036	Hs.99430	ref:NM_01	THC27609	ENST00000262630
59956316	+							
271801	+	GO:00069	GO:00160	GO:00048	Hs.661101	ref:NM_01	NP083697	ENST00000291633
52735630	+	GO:00063	GO:00056	GO:00037	Hs.549040	ref:NM_01	THC24745	ENST00000303406
150755	+		GO:00056	GO:00050	Hs.536319	gb:BC024	NP437544	ENST00000326643

3710061	-	GO:00081	GO:00055	GO:00036	Hs.72620	ref:NM_01	THC24889	ENST00000356899
54873005	+	GO:00066	GO:00057	GO:00047	Hs.234786	ref:NM_01	THC27076	ENST00000343744
37024476	-	GO:00063	GO:00056	GO:00036	Hs.654464	ref:NM_01	THC24781	ENST00000377859
1.12E+08	+	GO:00068	GO:00160	GO:00052	Hs.591327	ref:NM_01	THC24733	ENST00000368851
68945808	-	GO:00071	GO:00160	GO:00055	Hs.435719	gb:BC066	THC2605019	
40467886	+	GO:00068	GO:00055	GO:00051	Hs.8821	ref:NM_02	THC24780	ENST00000222304
1.75E+08	+	GO:00015	GO:00056	GO:00082	Hs.187284	gb:AF342	THC2490052	
8581588	-	GO:00065	GO:00055	GO:00036	Hs.657508	ref:NM_03	THC24643	ENST00000270328
15573926	-			GO:00198	Hs.98785	ref:NM_03	THC24739	ENST00000382324
34707972	-	GO:00001	GO:00057	GO:00050	Hs.693639	ref:NM_03	THC24731	ENST00000262866
604823	-	GO:00063	GO:00056	GO:00036	Hs.355284	ref:NM_03	THC24776	ENST00000246104
190258	+				Hs.350949	ref:NM_05	THC24833	ENST00000325113
51173240	-							
27985586	+	GO:00068	GO:00055	GO:00055	Hs.289106	ref:NM_13	NP402577	ENST00000334018
99362549	+	GO:00081	GO:00057	GO:00168	Hs.180346	ref:NM_13	THC24750	ENST00000370646
1.17E+08	+	GO:0008033(tRNA pr		GO:00047	Hs.21187	ref:NM_13	NP116008	ENST00000298746
46727205	+	GO:00076	GO:0016020(membrar		Hs.185777	ref:NM_14	THC24802	ENST00000326431
6448842	-	GO:00071	GO:00058	GO:00048	Hs.462529	ref:NM_14	NP114549	ENST00000356876
18682886	+				Hs.406913	ref:NM_15	THC24850	ENST00000325337
21586369	-			GO:00055	Hs.448572	ref:NM_15	THC24789	ENST00000379499
45025964	-	GO:00068	GO:00160	GO:00036	Hs.302513	ref:NM_15	THC24886	ENST00000372207
12607686	+	GO:00063	GO:00056	GO:00036	Hs.567641	ref:NM_15	NP540184	ENST00000343344
1.88E+08	+	GO:00512	GO:0009986(cell surfa		Hs.518480	ref:NM_15	NP541910	ENST00000382104
44331749	-	GO:0006468(protein a		GO:00001	Hs.351887	ref:NM_17	NP118865	ENST00000347193
86942549	-	GO:00069	GO:00058	GO:00055	Hs.405667	ref:NM_17	THC24908	ENST00000337868
86942549	-	GO:00069	GO:00058	GO:00055	Hs.405667	ref:NM_17	THC24908	ENST00000337868
1.5E+08	-	GO:0000045(autophagic vacuole f			Hs.655505	ref:NM_17	THC25198	ENST00000312968
1.16E+08	+				Hs.421750	ref:NM_17	NP116530	ENST00000314309
70441235	+				Hs.352515	ref:NM_17	NP115692	ENST00000331427
1068674	-				Hs.250557	gb:AK056	THC2478752	
52907943	+				Hs.424589	gb:AK128	THC2485408	
1299303	+	GO:00068	GO:00058	GO:00052	Hs.213284	ref:NM_18	NP398860	ENST00000324642
1097350	-			GO:00055	Hs.248290	ref:NM_19	NP146562	ENST00000319237
2.46E+08	-	GO:00071	GO:00160	GO:00048	Hs.23491	ref:NM_19	THC24845	ENST00000366487
85517663	+	GO:0007242(intracellular signalin			Hs.209542	gb:BC110	NP145707	ENST00000389938
7703008	-		GO:00160	GO:00055	Hs.220649	ref:NM_19	THC24791	ENST00000328853
34513037	-				Hs.522085	ref:NM_19	THC26145	ENST00000303992
1.54E+08	+	GO:00071	GO:00055	GO:00055	Hs.654541	ref:NM_19	THC24840	ENST00000368272
94983040	+	GO:00071	GO:00160	GO:00015	Hs.143315	ref:NM_19	THC26031	ENST00000316122
1.34E+08	+	GO:00063	GO:00056	GO:00036	Hs.573153	ref:NM_00	THC24965	ENST00000342854
5631911	-			GO:00055	Hs.356626	ref:NM_20	THC24966	ENST00000309324
1.43E+08	-				Hs.689547	ref:NM_20	THC2480285	
48144348	-							
60034044	+	GO:00069	GO:00058	GO:00048	Hs.645228	ref:NM_01	THC24706	ENST00000326321
60543369	+	GO:00069	GO:00058	GO:00050	Hs.643167	ref:NM_00	NP091718	ENST00000313421
60017780	+	GO:00069	GO:00058	GO:00048	Hs.661219	ref:NM_00	NP365687	ENST00000359085
84961721	-							
1.18E+08	+							
1.55E+08	+							
77498394	-		GO:0016020(membrar		Hs.163451	ens:ENST	THC26147	ENST00000330655
38693156	-				Hs.144075	gb:AK124	THC27852	ENST00000381390
5620855	+							
1.45E+08	-							
1.79E+08	+							
47302669	+							
6483835	-							
88256899	-							
1876799	-							
1.76E+08	+							
39974822	+							
18761119	+							
293088	+				Hs.632006	ref:NM_02	THC2463453	
75242880	-							
5892515	+							
68038530	-							
1.52E+08	-							
35204356	+							
1.51E+08	-							
45398263	-							
1.05E+08	-							
1.12E+08	-							
48997409	-							
19948283	+							
71629939	-							
1.65E+08	-							
4790447	+				Hs.558440	ens:ENST	THC26344	ENST00000348624
63293870	+							

54689234	-							
11439761	-	GO:00071	GO:00055	GO:00015	Hs.654486	ref:NM_006248;gb:K03208;gb:BC044827		
65474181	-							
18207722	+							
71448618	-				Hs.514494	ref:NM_00	THC25330	ENST00000375160
69030324	-							
56189478	+							
75659546	+							
17924078	-							
21853153	+							
33807542	+							
22485140	+							
13210359	-							
2.22E+08	-							
1.48E+08	+							
1.27E+08	+							
53290507	-				Hs.483519	ref:NR_00	NP584779	ENST00000379689
60017389	+	GO:00069	GO:00058	GO:00048	Hs.661219	ref:NM_00	NP365687	ENST00000359085

	Annotations							
**STOP**	**STRAND**	**GO biolog**	**GO cellula**	**GO molec**	**UniGene**	**Dbid**	**TIGRID**	**EnsemblID**
271823	+	GO:00069	GO:00058	GO:00048	Hs.512572	ref:NM_01	NP084034	ENST00000291633
1.61E+08	-	GO:00072	GO:00055	GO:00036	Hs.350581	ref:NM_05	THC24879	ENST00000367929
1.61E+08	-	GO:00072	GO:00055	GO:00036	Hs.350581	ref:NM_05	THC24879	ENST00000367929
86942549	-	GO:00069	GO:00058	GO:00055	Hs.405667	ref:NM_17	THC24908	ENST00000337868
2.46E+08	+							
86942549	-	GO:00069	GO:00058	GO:00055	Hs.405667	ref:NM_17	THC24908	ENST00000337868
15573926	-			GO:00198	Hs.98785	ref:NM_03	THC24739	ENST00000382324
1.34E+08	+	GO:00063	GO:00056	GO:00036	Hs.573153	ref:NM_00	THC24965	ENST00000342854
